# Ikaros sets the threshold for negative B-cell selection by regulation of the signaling strength of the AKT pathway

**DOI:** 10.1186/s12964-024-01732-5

**Published:** 2024-07-12

**Authors:** Patrick A. H. Ehm, Stefan Horn, Konstantin Hoffer, Malte Kriegs, Michael Horn, Susanne Giehler, Marcus Nalaskowski, Christoph Rehbach, Martin A. Horstmann, Manfred Jücker

**Affiliations:** 1 Institute of Biochemistry and Signal Transduction, Center for Experimental Medicine, University Medical Center Hamburg-Eppendorf, Martinistr. 52, Hamburg, 20246 Germany; 2 Department of Pediatric Oncology and Hematology, Research Institute Children’s Cancer Center Hamburg, University Medical Center, Hamburg, 20246 Germany; 3 Research Department Cell and Gene Therapy, Department of Stem Cell Transplantation, University Medical Center Hamburg-Eppendorf, Hamburg, 20246 Germany; 4 UCCH Kinomics Core Facility, University Cancer Center Hamburg (UCCH), University Medical Center Hamburg-Eppendorf, Hamburg, 20246 Germany; 5 Department of Radiotherapy and Radiation Oncology, University Medical Center Hamburg-Eppendorf, Hamburg, 20246 Germany; 6 University Cancer Center Hamburg, University Medical Center Hamburg-Eppendorf, Hamburg, 20246 Germany; 7 Mildred Scheel Cancer Career Center Hamburg, University Medical Center Hamburg-Eppendorf, Hamburg, 20246 Germany; 8 Department of Human Medicine, MSH Medical School Hamburg, Hamburg, Germany

**Keywords:** SHIP1, Inositol 5-phosphatase, PI3K/AKT-signaling, Acute lymphoblastic leukemia, ROS, Targeted hyperactivation

## Abstract

Inhibitory phosphatases, such as the inositol-5-phosphatase SHIP1 could potentially contribute to B-cell acute lymphoblastic leukemia (B-ALL) by raising the threshold for activation of the autoimmunity checkpoint, allowing malignant cells with strong oncogenic B-cell receptor signaling to escape negative selection. Here, we show that SHIP1 is differentially expressed across B-ALL subtypes and that high versus low SHIP1 expression is associated with specific B-ALL subgroups. In particular, we found high SHIP1 expression in both, Philadelphia chromosome (Ph)-positive and ETV6-RUNX1-rearranged B-ALL cells. As demonstrated by targeted knockdown of SHIP1 by RNA interference, proliferation of B-ALL cells in vitro and their tumorigenic spread in vivo depended in part on SHIP1 expression. We investigated the regulation of SHIP1, as an important antagonist of the AKT signaling pathway, by the B-cell-specific transcription factor Ikaros. Targeted restoration of Ikaros and pharmacological inhibition of the antagonistic casein kinase 2, led to a strong reduction in SHIP1 expression and at the same time to a significant inhibition of AKT activation and cell growth. Importantly, the tumor suppressive function of Ikaros was enhanced by a SHIP1-dependent additive effect. Furthermore, our study shows that all three AKT isoforms contribute to the pro-mitogenic and anti-apoptotic signaling in B-ALL cells. Conversely, hyperactivation of a single AKT isoform is sufficient to induce negative selection by increased oxidative stress. In summary, our study demonstrates the regulatory function of Ikaros on SHIP1 expression in B-ALL and highlights the relevance of sustained SHIP1 expression to prevent cells with hyperactivated PI3K/AKT/mTOR signaling from undergoing negative selection.

**Supplementary Information:**

The online version contains supplementary material available at 10.1186/s12964-024-01732-5.

## Introduction

Chromosomal translocations are among the most important genetic aberrations that are causally involved in the development of B-ALL. These translocations can produce chimeric fusion proteins, which often include a kinase or a transcription factor. Among leukemogenic gene fusion events, the Philadelphia chromosome-positive (Ph^+^) BCR-ABL is associated with the worst prognosis of all types of ALL [67].

It is identified in approximately 20% to 30% of adult ALL and approximately 3 to 5% childhood ALL [47, 51]. In contrast to chronic myeloid leukemia (CML) patients, who carry 95% of the Philadelphia chromosome and respond very well to treatment with tyrosine kinase inhibitors, Philadelphia chromosome (Ph)-positive ALL patients often show development of resistance and relapse [31, 37, 61]. An important gene that is also considered to be the cause of recurrences is the IKZF1 gene [55, 63]. In about 83% of the Ph-positive ALL, but not in the CML, Ikaros is functionally impaired [62]. In addition, Ikaros mutations in relapsed patients are identified as enriched [42].

B-cells are also subject to an active negative selection process [5, 68]. Both, weakening of the BCR signal strength below a minimum threshold (non-functional BCR) and hyperactivation above a maximum threshold (autoreactive BCR) can lead to cell death and elimination of B-cell clones in the early stages of B-cell development [65, 68, 77]. The oncogenic activation (e.g., through BCR-ABL or RAS) of BCR-associated signaling cascades in B-cell diseases is the functional equivalent of positive selection during normal B-cell development [9, 22, 34, 71, 85]. Phosphatases often serve as opponents of the BCR signaling and the signaling of activated kinases. Recent studies show that particularly high expression levels of inhibitory phosphatases (such as SHIP1, PTEN and DUSP6) in leukemia allow cells with strong oncogenic B-cell receptor signaling to escape negative selection by attenuating signal strength [9, 14, 71, 72]. Of the B-cell receptor-dependent signaling cascades, the PI3K/AKT signaling pathway takes on a prominent role. The inositol phosphatase SHIP1 possesses important functions as a negative regulator of the PI3K/AKT signaling pathway in hematopoietic cells [11, 81].

Here, we examined the regulation of SHIP1 by the B-cell-specific transcription factor Ikaros and the biological effects after downregulation of SHIP1 expression in ALL cells. In addition, AKT isoforms were studied by AKT isoform-specific RNA interference in ALL cells, especially in the context of the B-cell selection process. In summary, our work aims at improving the understanding of the role of Ikaros/SHIP1 regulation and AKT isoforms in ALL, which could form the basis of a new targeted intervention of ALL in the future.

## Material and methods

### Patient material

Bone marrow and peripheral blood samples were obtained after informed consent of parents or guardians from children diagnosed with B- and T-ALL who had been enrolled in the multi-center CoALL studies -92, -97 and -03 [21, 29]. The trials achieved approval by the ethics committee of the City of Hamburg (No. 2077; 12 August 2003) and institutional review boards of participating trial centers, and they were conducted according to the principles of the Declaration of Helsinki. Material (buffy coat) from anonymized healthy donors was made available as part of the surplus material from the transfusion medicine department of the UKE, according to a statement by the Central Ethics Committee of the BÄK (federal medical association): “The (further) use of human body materials for medical research purposes (20 February 2003)”. CD19-positive B-cells were isolated from fresh peripheral blood mononuclear cells of healthy donors using EasySep Human CD19 positive selection kit (STEMCELL Technologies) according to the manufacturer’s instructions.

### Cell culture and inhibitors

All cells were cultivated at 37°C and 5% CO_2_ according to the DSMZ. The respective medium was mixed with FCS and 1% penicillin / streptomycin (complete medium). Used cells were: SupB15 (B-ALL; DSMZ No. ACC389), Nalm-6 (B-ALL; DSMZ No. ACC128), Sem (B-ALL; DSMZ No. ACC546), Reh (B-ALL; DSMZ No. ACC22), 697 (B-ALL; DSMZ No. ACC42), Call2 (B-ALL; DSMZ No. ACC341), Jurkat (T-ALL; DSMZ No. ACC282), CCRF-CEM (T-ALL; DSMZ No. ACC240), K562 (CML; DSMZ No. ACC10), Nalm-1 (CML in blast crisis; DSMZ No. ACC522), BV173 (CML in blast crisis; DSMZ No. ACC20), Tom-1 (B-ALL; DSMZ No. ACC578). To inhibit specific proteins and signaling pathways, the cells were treated with inhibitors. For inhibition assays, 5 × 10^6^ cells were washed with PBS and resuspended in fresh medium supplemented with imatinib (LC Laboratories), CX4945 (Abcam), MK2206 (Merck) or RAD001 (Sellekchem). Control cells were treated in parallel with corresponding inhibitor solvents. Inhibitor dosis and time of treatment varied as stated in the figure legends.

### Construction of plasmids

The coding sequences of full-length cDNA of human AKT1 (NM 001014431.2), AKT2 (NM 001626.6) and AKT3 (NM 005465.7) were obtained from Sino Biologicals (Pennsylvania, U.S.A.). By PCR amplification, a 5’-FLAG-tag and restriction sites (AKT1 and AKT2: BamHI/BamHI; AKT3: BsrGI/BsrGI) were introduced facilitating subsequent cloning into vector pCW57-GFP-P2A-MCS (Addgene, ID 89181). In addition, the constitutive active AKT isoform (T308D and S473D; DD-mutant) was generated by site-directed mutagenesis using the modified QuikChange [80].

LeGO-iG2-Puro + vector was a kind gift from Kristoffer Riecken and Boris Fehse [82]. Human SHIP1 cDNA was sub-cloned into the LeGO-iG2-Puro + and pCW57-GFP-P2A-MCS vectors using the unique BamHI/BamHI site [15].

Wildtype human Ikaros cDNA was received from PlasmID (Harvard Medical School; ID HSCD00002517). The coding sequence was equipped with a 5’-FLAG-tag and NotI-restriction sites by PCR and cloned into LeGO-iG2-Puro + [19]. The LeGO-iG2-Puro-Ik-wt construct was then used as a template for a QuikChange mutagenesis. The complete sequence from exon 3 to exon 6 was deleted within the Ikaros wildtype sequence to obtain the dominant-negative Ikaros isoform 6 (IK6).

AKT amplification

AKT1_FW: ATGGATCCGCCACCATGGCGGACTACAAAGACGATGACGACAAGAGCGACGTGGCTATTGTGAAG

AKT1_RV: TAGGATCCTCAGGCCGTGCCGCTGGCCGAGTA

AKT2_FW: ATGGATCCGCCACCATGGCGGACTACAAAGACGATGACGACAAGAATGAGGTGTCTGTCATCAAA

AKT2_RV: TAGGATCCTCACTCGCGGATGCTGGCCGAGTA

AKT3_FW: ATTGTACAGCCACCATGGCGGACTACAAAGACGATGACGACAAGAGCGATGTTACCATTGTGAAA

AKT3_RV: TATGTACATCATTCTCGTCCACTTGCAGAGTA

Ikaros amplification

IKZF1-FP : 5´-ATGCGGCCGCGCCACCATGGCGGACTACAAAGACGATGACGACAAGGATGCTGATGAGGGTCAAGAC–3´

IKZF1-RP : 5´- TAGCGGCCGCTCAGCTCATGTGGAAGCGGTGCTC –3´

IK6-isoform

IK6-FP: 5´- TCCAAGAGTGACAGAGTCGTGGGGGACAAGGGCCTGTCCGAC-3´

IK6-RP: 5´- GTCGGACAGGCCCTTGTCCCCCACGACTCTGTCACTCTTGGA-3´

### Lentiviral transduction

Generation of pseudo-typed lentiviral vectors and transduction were performed as previously described [59]. Target cells were seeded at a density of 5 × 10^5^ cells per 6-well plate. Virus supernatant was added. Transduced cells were selected with puromycin or G418 (Sigma-Aldrich, Taufkirchen, Germany) for at least 1 week (unless otherwise stated). Cells with single and double knockdowns of AKT isoforms were generated by using isoform specific shRNAs. Control cells were transduced similarly with non-target control vectors. pLKO.1-puro vector encoding either shRNAs directed against AKT1, AKT2, AKT3, SHIP1 or scrambled shRNA were purchased from Sigma-Aldrich (Taufkirchen, Germany) and used as described previously [17, 19, 26].

### Western blot analysis

Western blot analysis was performed as described previously [15, 16, 64]. Protein lysates were prepared by lysing cells in trichloroacetic acid. Proteins were analysed by SDS–polyacrylamide gel electrophoresis and transferred to nitrocellulose membrane. A loading control using Ponceau S staining of the membrane was performed. Subsequently, the membrane was hybridized with either mouse anti-HSC70 (B-6; Santa Cruz), rabbit anti-phospho-AKT (9018; Cell Signaling), rabbit anti-pan-AKT (11E7; Cell Signaling), mouse anti-phospho-tyrosine (pY-99; Santa Cruz), rabbit anti-phospho-GSK3β-S9 (9336; Cell Signaling), rabbit anti-phospho-S6-ribosomal protein S240/244 (2215; Cell Signaling), mouse anti-SHIP1 (P1C1; Santa Cruz), mouse anti-PTEN (A2B1; Santa Cruz), rabbit anti-phospho-AMPKa-T172 (2535; Cell Signaling), rabbit anti-G6PD (8866; Cell Signaling), rabbit anti-Ikaros (sc-13039; Santa Cruz), rabbit anti-TXNIP (14,715; Cell Signaling), mouse anti-FLAG (F1804; Sigma). Further antibodies used were anti-mouse IgG HRP-conjugated, anti-rabbit IgG HRP-conjugated (both Cell Signaling) and goat anti-rat IgG HRP (Santa Cruz). Subsequently, protein expression was quantified using LAS-3000 Imager from Fuji (Raytest).

### RNA isolation, cDNA synthesis and real-time quantitative PCR (qPCR)

RNA isolation, cDNA synthesis and real-time quantitative PCR (qPCR) were performed as described previously [16]. Total RNA was isolated with the Direct-Zol RNA MiniPrep Kit (ZYMO Research) following the manufacturer's instruction. First strand cDNA was synthesized with the Promega M-MLV Reverse Transcriptase (Promega) following the manufacturer's protocol. Sense and antisense oligonucleotide primers for amplification of mRNAs of human SHIP1 and the housekeeping genes GAPDH were designed as followed:

SHIP1-3’-UTR-FP 5´- GGAAATCAGCTCCTATTCTCCA-3´

SHIP1-3’-UTR-RP 5´- CACACACCACTGGATTTAGCTC-3´

GAPDH-FP: 5´- GAGTCAACGGATTTGGTCGT-3´

GAPDH-RP: 5´- TTGATTTTGGAGGGATCTCG-3´

Oligonucleotide primers were obtained from Eurofins MWG (Ebersberg, Germany). LightCycler Real-Time PCR reactions and data analysis were performed on a LightCycler system, Version 3.5 and LightCycler detection software, according to the instructions of the manufacturer (Roche). The relative expression of the amount of SHIP1 mRNA was determined by normalization to the reference gene GAPDH.

### Live cell imaging

The IncuCyte ZOOM live cell imaging system (Sartorius) was used to determine the proliferation and migration behavior of the examined cells. Cells were each seeded in a cell density of 3 × 10^4^ cells / well in a 96-well plate (Greiner Bio-One, Frickenhausen, Germany) with 200 µl medium, unless otherwise stated, and incubated at 37^◦^C and 5% CO_2_ for at least 48 h. Subsequently, series of phase-contrast images were taken and analysed by creating a confluence mask with the associated IncuCyte Zoom software, according to the manufacturer’s instructions.

### Alamar blue assay

Cells were seeded in a cell density of 3 × 10^4^ cells / well in a 96-well plate (Greiner Bio-One, Frickenhausen, Germany) and incubated at 37°C and 5% CO_2_ for at least 48 h with or without inhibitor treatment. Cell viability and proliferaton were measured using Alamar blue Assay. The Alamar blue assay is widely used to investigate cytotoxicity, cell proliferation and cellular metabolic activity [30, 53]. Therefore, cells were incubated with 5 ng/mL resazurin (Sigma-Aldrich, St. Louis, MO, USA) for 240 min at 37°C in a humidified atmosphere. Fluorescence based absorption was measured at 540 nm on a microplate reader (Tecan, Maennedorf, Switzerland).

### Boyden chamber assay

Analysis of cell migration was performed using a Boyden chamber test (8 μm pore size in a 96-well plate format). Cells were suspended to yield a cell number of 50,000 cells per upper Boyden chamber in serum-free medium. Medium in the lower Boyden chamber was supplemented with 10% heat-inactivated FCS serving as chemo-attractant. After incubation, the non-migrating cells on the upper surface of the inserts were removed and viability of cells in the lower chamber was measured.

### Apoptosis assay

5 × 10^5^ cells were plated in 6-well culture plates two days before analysis. Unstained cells were used as negative controls. After 48 h, cells were carefully washed with PBS, washed in 1 × binding buffer and resuspended in 100 µL 1 × binding buffer supplied with APC-conjugated Annexin V according to the manufacturer’s instructions. Analysis by flow cytometry was performed on a CytoFlex instrument (Beckman Coulter).

### Reactive oxygen species level measurement

For evaluation of intracellular reactive oxygen species (ROS) levels, cells were incubated for 7 min with 1 μM 2′,7′-dichlor-dihydrofluorescein-diacetate (DCFH-DA, Sigma) at 37°C for oxidation of the dye by ROS. After washing with PBS, the cells were incubated for additional 15 min at 37°C in PBS to allow complete deacetylation of the oxidized form of DCFH-DA by intracellular esterases (recover-time) and subsequently the fluorescence was measured.

### Functional kinome profiling

Functional kinome profiling was used to analyze the activity of protein tyrosine kinases (PTK) and serine/threonine kinases (STK), as described previously [4]. Briefly, whole cell lysates were generated using MPER Mammalian Extraction Buffer (Thermo Fisher Scientific, Darmstadt, Germany) with Halt Phosphatase Inhibitor and EDTA-free Halt Protease Inhibitor Cocktail (Pierce Biotechnology, Rockford, IL, USA). The profiling was performed using the PamStation12 (UCCH Kinomics Core Facility, UKE, Hamburg, Germany) and corresponding PTK and STK microarrays (PamChip), according to the manufacturer’s protocols (PamGene, Hertogenbosch, The Netherlands). Lysates containing 5 µg of protein (PTK) or 1 µg (STK) and 400 µM ATP were applied to each array. Phosphorylation of the kinase-specific peptide sequences was detected using either fluorescein-labelled anti-phosphotyrosine antibodies (PTK) or anti-phospho-Ser/Thr antibodies, followed by a secondary polyclonal swine anti-rabbit immunoglobulin-FITC antibody (STK; PamGene International, the Netherlands). Analysis of the intensity was conducted with the Evolve software v. 1.0 (PamGene, The Netherlands) and for further analysis, the intensities were log2-transformed and proceeded using the BioNavigator software v. 6.0 (BN6, PamGene, the Netherlands).

### Animal experiment

Animal experiments were performed in accordance with ARRIVE guidelines and legal regulations (Hamburg TV 68/17) [39]. Non-irradiated, immunodeficient NOD scid gamma mice (NOD.CG-Prkdcscid IL2rgtm1Wji/SzJ; NSG mice) were injected intravenously with 1 × 10^6^ cells each in PBS. For preparation, femurs were cut open at the ends and the bone marrow was isolated by centrifugation (8000 × g for 30 s). Splenocytes and hepatocytes were separated through a 70 µm sieve (BD Falcon) and taken up in PBS. The relative proportion of human SHIP1-expressing cells in spleen and liver was analysed by flow cytometry after staining with an FITC-conjugated anti-hCD45 antibody (2D1; BioLegend) and an APC-conjugated anti-hSHIP1 antibody (P1C1-A5; BioLegend). The in vivo imaging was carried out at the core facility “In vivo Optical Imaging” of the University Cancer Center Hamburg (UCCH) on an IVIS Spectrum in vivo imaging system (PerkinElmer) [73]. For imaging of the NSG mice, the animals were first anesthetized with isoflurane before injecting intraperitoneally 100 µg/mouse of coelenterazine substrate (SYNCHEM). The image was recorded 15 min later and analysed using the Living Image software (version 4.3.1).

### Gene expression and bioinformatic analysis

RNA-seq data of B-ALL patient samples were taken from a publicly available dataset of St. Jude Research Hospital http://www.stjuderesearch.org/data [27]. Gene expression analysis was carried out on VST data. Microarray data of the mixed leukemia dataset of the MILE study was taken from GEO (accession: GSE13204). Patient samples were ordered according to their molecular subgroup affiliation. GSEA (v4.0.3) software [78] was used for gene set enrichment analysis using the MSigDB Hallmark [49] gene sets (significance FDR q-val: * ≤ 0.25; ** ≤ 0.05; *** ≤ 0.01). For input, gene lists were obtained by forming the tercile and dividing the strength of the gene expression of the gene of interest into high, intermediate and low. Gene set enrichment of high and low expressed genes was subsequently received as a ranked list with overrepresented genes (top or bottom) from the expression dataset. The genes were selected for further analysis according to a score of > 0.45 and < -0.45. Data from gene ontology (GO), Kyoto Encyclopedia of Genes and Genomes (KEGG) and Reactome Pathways enrichment analysis were obtained using the g:profiler and StringDB database [69, 74]. Gene set lists for oncogene/driver gene/tumor suppressor gene assignment, transcription factor gene assignment and cell surface marker gene assignment are accessible at the following reference [2, 45, 48]. The annotation of the kinome tree genes was performed using the kinome render tool [8].

### Statistical analysis

The statistical significance (* *p* ≤ 0.05; ** *p* ≤ 0.01; *** *p* ≤ 0.001) was determined by the Student’s t-test, unless otherwise stated. Each experiment was performed at least in triplicate. All figures show mean values, and error bars represent SD, unless indicated otherwise. The statistical analysis data were analysed using GraphPad Prism (GraphPad Software Inc., San Diego, CA, USA). Gene expression data were analysed using one-way ANOVA-tests and Benjamini–Hochberg correction for multiple testing.

## Results

### Differential expression of SHIP1 in B-ALL

SHIP1 serves as important antagonist of the PI3K/AKT/mTOR signaling pathway in hematopoietic cells. By analyzing the INPP5D expression from the leukemia dataset of Kohlmann et al. [41], GSE13204 we observed that the SHIP1 mRNA expression was differentially expressed across all B-ALL subtypes and increased in the group of ETV6-RUNX1-positive B-ALL and in the group of BCR-ABL-positive B-ALL, in comparison to other translocations within the B-ALL and compared to healthy hematopoietic cells (Fig. S1A). Mutations of the INPP5D gene (SHIP1) in hematopoietic cells (126 mutations in 8122 samples examined; 1.55% of cases) were rare [24], COSMIC status: 06/25/24]. Four mutations of the INPP5D gene in patients with acute lymphoblastic B-cell leukemia were identified in the COSMIC database. However, a detailed analysis of the dataset from Chouvarine and coworkers [10] showed that several SHIP1 fusion proteins were found in the B-other cohort (Fig. S1B). These fusion proteins were identified, particularly in the Ph-like subtype.

**Fig. 1:**
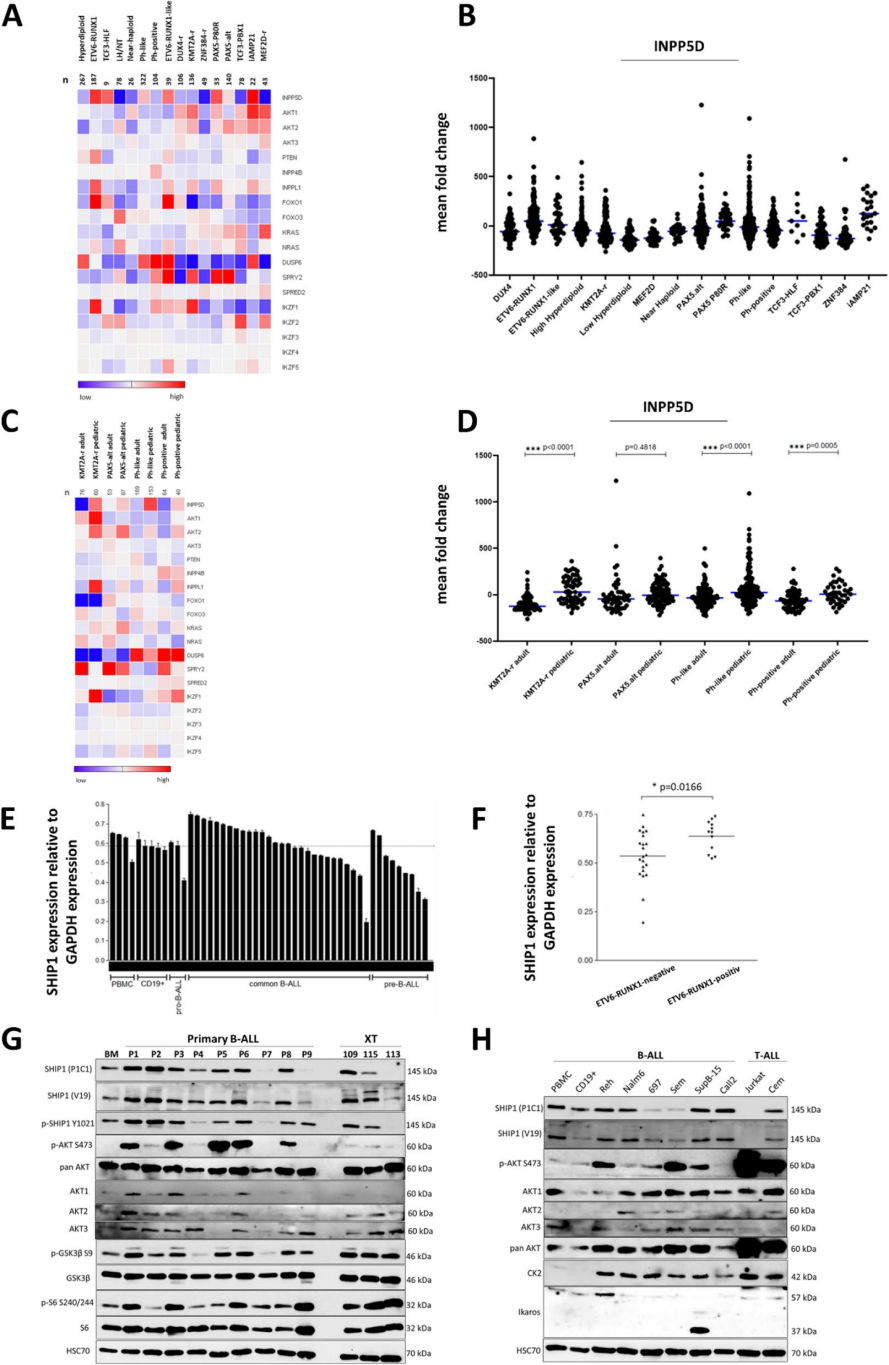
Differential gene expression of INPP5D in B-ALL subtypes. **A** Gene expression data of INPP5D (SHIP1) and other regulators of AKT and RAS signaling as well as Ikaros transcription factors in B-ALL subtypes were shown in a heatmap. Taken from the publicly available dataset of [http://www.stjuderesearch.org/data]. **B** Dot plot analysis of INPP5D gene expression in individual B-ALL patients. **C** Gene expression data of INPP5D and other regulators of AKT and RAS signaling as well as Ikaros transcription factors in selected B-ALL subtypes across age. **D** Dot plot analysis of INPP5D gene expression in selected B-ALL subtypes in pediatric versus adult patients. **E** Relative gene expression of SHIP1 at the mRNA level in B-ALL patient samples, were divided into pro-B-ALL, common B-ALL and pre-B-ALL. PBMCs and purified CD19-positive cells from healthy donors served as controls. The mean relative gene expression in the healthy CD19-positive cells was illustrated as a dashed line. **F** Relative INPP5D gene expression in ETV6-RUNX1-negative (*n* = 22) versus -positive B-ALL patients (*n* = 12). The horizontal line shows the mean expression level in each cohort. **G** Expression of SHIP1 protein level and concomitant phosphorylation status of the AKT signaling pathway in nine B-ALL patient samples (P1-P9) and three B-ALL samples isolated from xenotransplanted mice (XT), in comparison to bone marrow cells from a healthy donor (BM). **H** Analysis of SHIP1 protein expression and activation of the AKT signaling pathway in six human B-ALL (Reh, Nalm-6, 697, Sem, SupB15 and Call2) and two human T-ALL cell lines (Jurkat and CEM)

We analysed the gene expression of INPP5D and additional important phosphatases and kinases of the AKT and RAS signaling pathway in different subtypes of B-ALL using transcriptome data of 1,639 B-ALL patient samples (Fig. 1) [http://www.stjuderesearch.org/data]. The available B-ALL patient samples were arranged according to their subgroup affiliation (Fig. 1A). INPP5D was differentially expressed across all B-ALL subtypes (Fig. 1B). We noticed a higher expression in the ETV6-RUNX1, iAMP21, PAX5-P80R, TCF3-HLF and ETV6-RUNX1-like subgroup. In contrast, we saw a lower expression of the INPP5D gene expression in the MEF2D-r, LH/NT, ZNF384-r, TCF3-PBX1 and near-haploid subgroup. Furthermore, we noticed a different gene expression pattern of the INPP5D gene across age in different B-ALL subtypes (Fig. 1C). The INPP5D gene was significantly higher expressed in the pediatric cohort of the KMT2A, Ph-positive and Ph-like subgroups than in the adult cohorts of these subgroups (Fig. 1D). This suggests an age-specific expression of SHIP1.

In addition, B-ALL patient samples (*n* = 40) were used to analyze SHIP1 expression at the RNA level by quantitative real-time PCR (RT-qPCR) (Fig. 1E). Healthy B-cells (CD19-positive; *n* = 5, PBMC; *n* = 4) were used as control. The distribution of SHIP1 expression in the primary B-ALL cells was heterogeneous compared to the healthy CD19-positive cells. In detail, 12 samples of the examined B-ALL patient samples showed an ETV6-RUNX1 fusion. Here, a significant difference in the SHIP1 expression in the group of the ETV6-RUNX1-positive B-ALL cells (*n* = 12) compared to the SHIP1 expression in the group of the ETV6-RUNX1-negative (*n* = 22) B-ALL cells were observed (Fig. 1F). For complementation to the gene expression data, we investigated protein expression of SHIP1 from selected B-ALL cases (Fig. 1G). Six of nine samples (66.7%) showed a significantly stronger expression of SHIP1 than the cells of the healthy bone marrow. Primary B-ALL patient samples were also examined for the expression of the three AKT isoforms using isoform-specific antibodies. A phosphorylation of AKT at the serine residue 473 was seen in 10 of 12 (83.3%) primary ALL and patient-derived samples. Furthermore, SHIP1 protein expression in B-ALL and T-ALL cell lines was examined (Fig. 1H). Reh (ETV6-RUNX1), Nalm-6 (DUX4-r), SupB15 (BCR-ABL) and MHH-CALL2 cells showed an increased SHIP1 expression. In summary, SHIP1 was expressed heterogeneously in B-ALL with clear subgroup-dependent expression differences.

### BCR-ABL inhibition by imatinib increased SHIP1 expression in Ph-positive CML and B-ALL cells

**Fig. 2:**
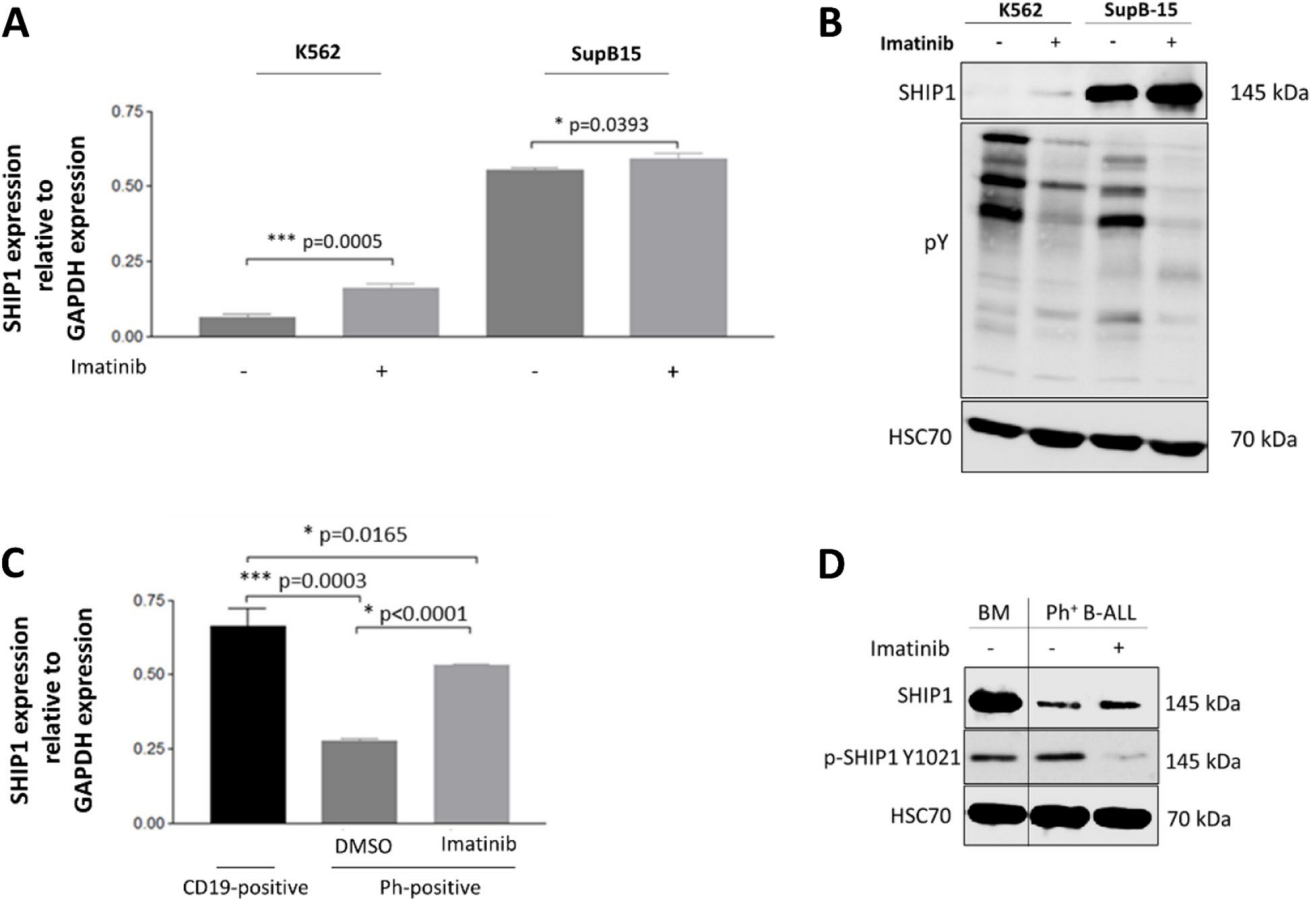
Different expression of SHIP1 in BCR-ABL-positive CML and B-ALL cells. Expression of SHIP1 in BCR-ABL-positive leukemia cell lines was analysed (**A**) at the mRNA level by RT-qPCR and (**B**) at the protein level by Western Blot in K562 and SupB15 cells before and after treatment with 1 µM imatinib for 48 h. **C** Relative gene expression and (**D**) protein level of SHIP1 in a BCR-ABL-positive B-ALL patient sample with and without treatment with 5 μM imatinib or DMSO for 5 h

In patients with chronic myeloid leukemia (CML), SHIP1 had been shown to be phosphorylated by BCR-ABL and subsequently degraded [70]. However, little is known about the expression and regulation of SHIP1 in Ph-positive B-ALL cells. Therefore, both the BCR-ABL-positive B-ALL cell line SupB15 and the BCR-ABL-positive CML cell line K562 were examined. SupB15 and K562 cells were either treated with the BCR-ABL inhibitor imatinib or with DMSO and analysed by RT-qPCR (Fig. 2A) and Western blot (Fig. 2B). Strikingly, the two BCR-ABL-positive cell lines differed greatly in their endogenous SHIP1 expression status. SupB15 cells showed a strong SHIP1 expression, whereas the CML cell line K562 expressed low amounts of SHIP1 mRNA and protein. Nevertheless, treatment with imatinib led to an increase in SHIP1 mRNA and protein levels in both cell lines (Fig. 2A and B). This result was confirmed for a primary Ph-positive B-ALL patient-derived xenograft (Fig. 2C and D).

### The proliferation of Ph-positive B-ALL cells *in vitro* and their tumorigenic spread in vivo depended in part on SHIP1 expression strength

**Fig. 3:**
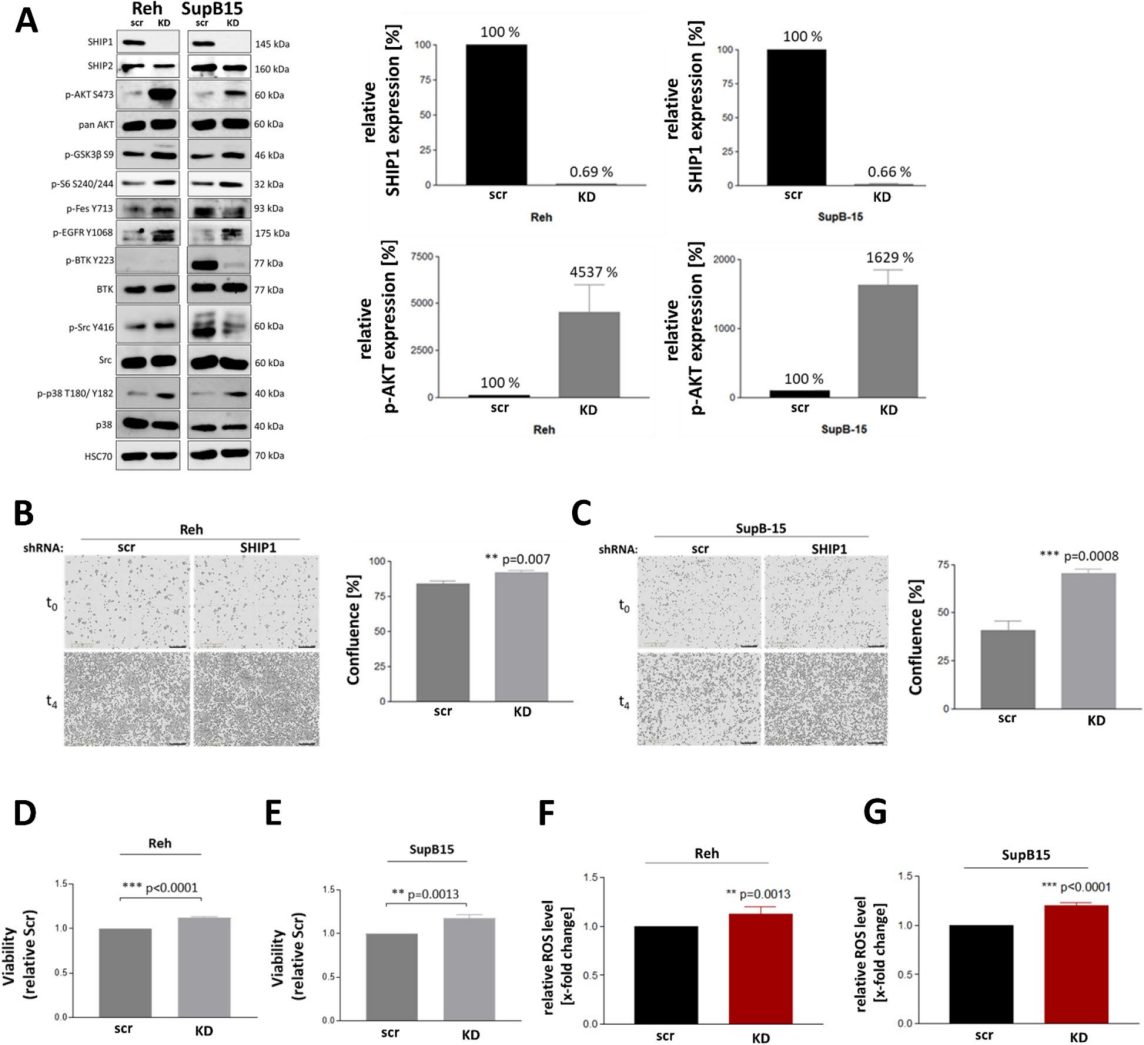
High SHIP1 expression attenuated AKT signaling and proliferation of B-ALL cells in vitro. **A** Effects of SHIP1-knockdown on signal transduction mediated by AKT and other kinases in ETV6-RUNX1-positive Reh and Ph-positive SupB15 B-ALL cell lines. Quantification of SHIP1 expression levels and phosphorylation of AKT at Ser473 (right panel). Increased proliferation of Reh (**B**) and SupB15 cells (**C**) after SHIP1-knockdown. Cell proliferation was monitored by live cell imaging over four days after seeding. Representative images of cell confluency were shown. In addition, viability and proliferation of Reh (**D**) and SupB15 cells (**E**) were determined using an Almar blue assay. Analysis of intracellular reactive oxygen species (ROS) levels in Reh (**F**) and SupB15 (**G**) after SHIP1-knockdown

To better understand the role of SHIP1 in B-ALL cells, expression of SHIP1 in the ETV6-RUNX1-positive Reh cells and the Ph-positive SupB15 cells was downregulated by targeted expression of SHIP1-specific shRNAs. SHIP1-knockdown was confirmed by Western Blot. As shown in Fig. 3A and B, expression of the SHIP1-specific, but not of the control shRNA resulted in strong reduction of SHIP1 protein expression in both ALL cell lines and a concomitant increase in the phosphorylation of AKT by a factor of 45 (Reh) and 16 (SupB15), respectively.

To identify kinases whose activity profile was differentially regulated after knockdown of SHIP1 protein expression in SupB15 B-ALL cells, a functional kinome profiling (Fig. S2A) was used and the results were validated by Western Blot (Fig. 3A). A kinome tree visualization [54, 58] of the differential kinase activity profile after SHIP1-knockdown showed that the family of tyrosine kinases (TK) and calcium/calmodulin-dependent protein kinases (CAMK) were predominantly affected (Fig. S2B). The Tec-kinase BTK was prominently displayed. The differentially regulated kinases were consequently grouped according to their classification in known signaling pathways (PI3K/AKT: KEGG hsa04151, RTK: gene set of [2]. The SHIP1-knockdown in SupB15 cells led to a strong decrease (> 2 log2FC) in the activity of TEC kinases (BTK, TEC), FES, ABL1, the Src kinase FGR and cell surface receptors (KDR, EGFR) (Fig. S2C–E), which were confirmed as significantly regulated by volcano plot visualization (Fig. S2F). These results were confirmed in Western blot analysis for BTK, FES and the Src-kinase family as well as AKT, S6 and GSK3β (Fig. 3A).

In addition, we analysed the impact of the SHIP1-knockdown on cell proliferation and viability. In both ALL cell lines, SHIP1-knockdown resulted in a significant increase in cell proliferation and viability, as measured by live-cell-imaging and Alamar blue assay, respectively (Fig. 3B/C and D/E). The analysis of intracellular reactive oxygen species (ROS) levels revealed significantly elevated levels both, in Reh and SupB15 cells after specific SHIP1-knockdown (Fig. 3F and G). These data indicated increased proliferation and cell stress in SHIP1-knockdown ALL cells.

Next, we analysed the gene set enrichment for the INPP5D high and low expression phenotype using the transcriptome data of Ph-positive and ETV6-RUNX1 patient samples [http://www.stjuderesearch.org/data], as described in the method section. Gene sets with a FDR q-val ≤ 0.25 were each considered significant and presented in a bar chart with the Normalized Enrichment Score (NES) value (Fig. S3A and S3B). Interestingly, no gene set was significantly enriched for the INPP5D high expression phenotype in the Ph-positive cohort. Among the gene sets significantly enriched for the INPP5D low expression phenotype between the Ph-positive and the ETV6-RUNX1 subgroup, most genes were found in both HALLMARK datasets (oxidative phosphorylation, MYC targets, DNA-repair, adipogenesis, MTORC1 signaling, reactive oxygen species pathway and unfolded protein response). This suggested a common mechanism for the influence on the energy metabolism and cellular stress response in both subgroups. The gene expression analysis of kinome-specific genes according to the classification of INPP5D with high, intermediate or low expression in transcriptome data from B-ALL patient samples with Ph-positive (Fig. S3C) subtype showed that the genes ABL1, FES, TEC and BTK, together with INPP5D, exhibited a gradual change in gene expression from the low to high expression classification.

In the next step, we compared the significantly enriched genes (for INPP5D gene expression) of the ETV6-RUNX1-positive and the Ph-positive subtypes (Fig. S3D; Table S1-S10). Subsequently, gene ontology (GO) analysis of molecular function of common INPP5D enriched genes in the Ph-positive and in the ETV6-RUNX1 subtype was carried out using the g:profiler tool. The analysis of the overlapping genes using the dataset of [48] showed that 16.5% of them had a putative oncogenic function, 10.9% had a putative driver function and 7.8% had a putative tumor suppressor function. Since these genes showed a particular transcriptional focus, they were further subdivided into transcription factor genes (Fig. S3E) and cell surface marker genes (Fig. S3F) using the dataset of [2, 45]. Transferring the results to all subgroups showed the strongest INPP5D correlation for the genes *ZNF496* (score: 0.7), *MBD1* (score: 0.55), *STAT5A* (score: 0.59), *MLXIP* (score: 0.56), *L3MBTL1* (score: 0.57), *SRCAP* (score: 0.53) and *AHDC1* (score: 0.5) for transcription factor genes as well as *CLCN6* (score: 0.68), *STIM1* (score: 0.59) and *GPR107* (score: 0.54) for cell surface marker genes.

**Fig. 4:**
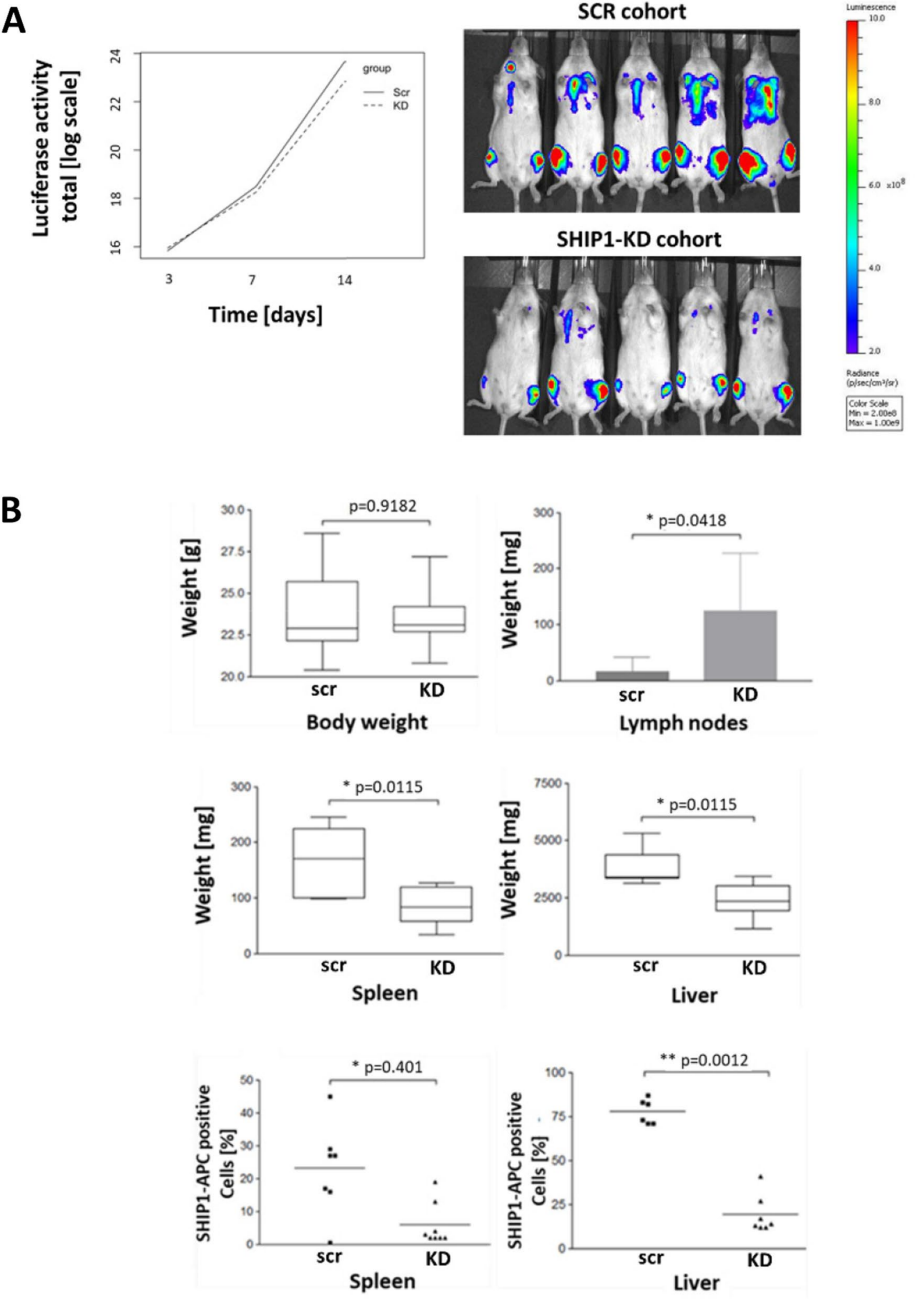
High SHIP1 expression altered tumorigenic spread of B-ALL cells in vivo. **A** Reh cells expressing luciferase in conjunction with either the SHIP1-specific or the scrambled shRNA were injected into NOD-scid-gamma mice (NSG). Initial cell engraftment was monitored by in vivo imaging of luciferase activity on day 3, 7 and 14 post transplantation (p.t.). A representative analysis of mice with unmodified SHIP1 expression (SCR) and with SHIP1-knockdown (KD) at day 14 p.t. was shown. **B** At the end of the experiment, the total body weights and the weights of various organs as well as the relative proportion of human SHIP1-expressing cells in spleen and liver were quantified. Flow cytometry staining was performed with an FITC-conjugated anti-hCD45 and an APC-conjugated anti-hSHIP1 antibody

To investigate the effect of a reduced SHIP1 expression in B-ALL cells in vivo, ETV6-RUNX1-positive Reh cells either with or without SHIP1-knockdown were intravenously injected into NOD-scid-gamma mice (NSG). The growth of the luciferase-expressing cells was monitored by in vivo imaging. In all animals, malignant cell growth was observed after application (Fig. 4A). In accordance with termination criteria, the animal experiment was ended individually and hematopoietic cells from femur, spleen and liver were analyzed. Enlarged lymph nodes were observed specifically in the SHIP1-knockdown cohort in 7 out of 9 cases (77.8%), which indicated a preferential spread of SHIP1-knockdown cells into this lymphoid tissue. In contrast, a significantly higher liver (1.5 times) and spleen weight (2 times) was observed in the cohort with unmodified SHIP1 expression (Fig. 4B). The number of bone marrow cells isolated from the femur was too small at the time of processing, so that they could not be analysed. As shown by flow cytometry analysis, however, SHIP1 expression was strongly decreased in leukemic cells from spleen and liver when isolated from the knockdown mice (Fig. 4B and Fig. S4). These data showed that in a xenotransplantation model, Reh cells with reduced SHIP1 expression had a similar capacity to engraft and expand as their control cells with unaltered SHIP1 expression, but led to a differential spread of cells to various hematopoietic and non-hematopoietic organs in end-stage disease.

### The transcription factor Ikaros regulated SHIP1 expression in ALL cells

**Fig. 5:**
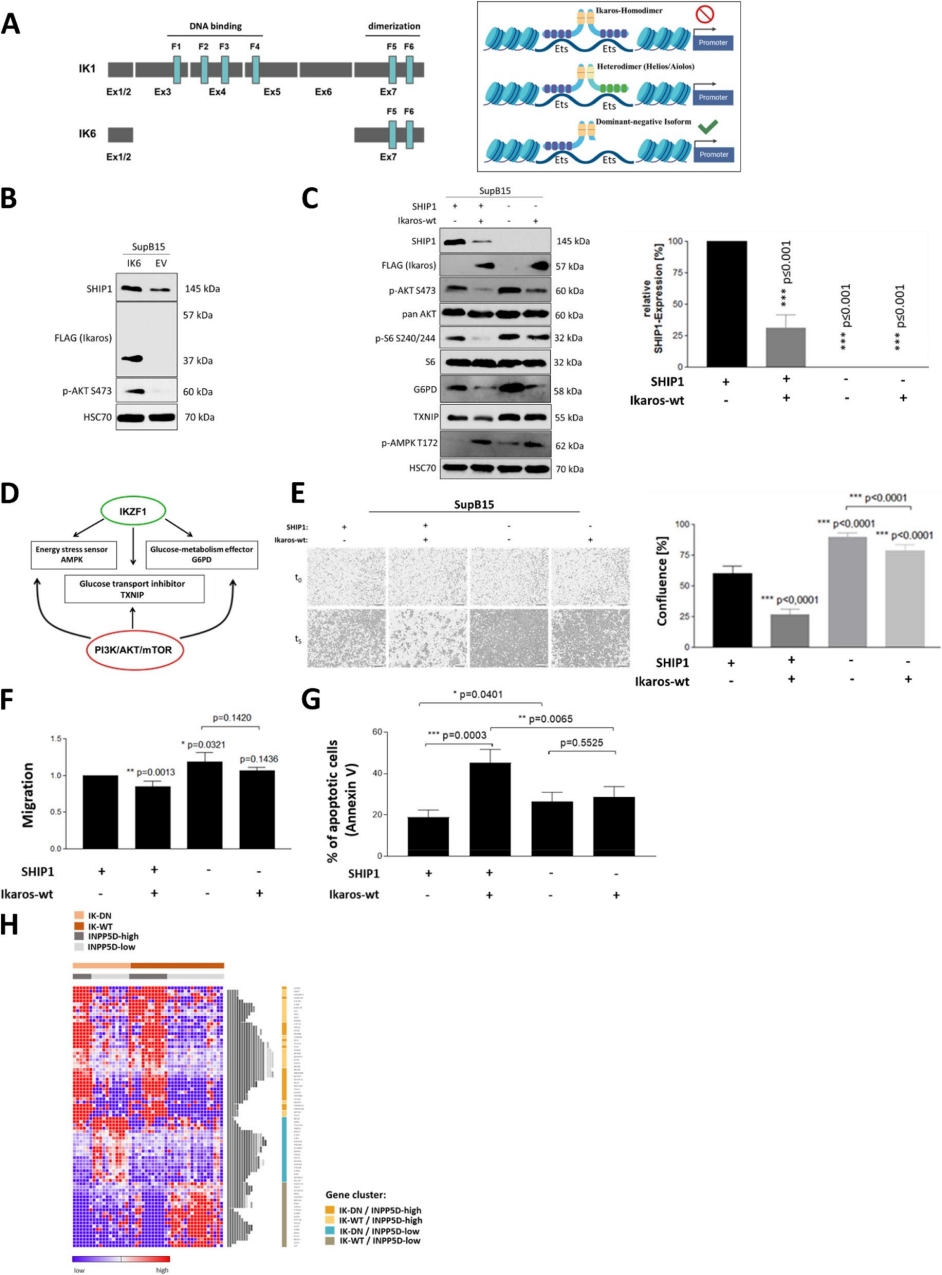
Ikaros regulated SHIP1 expression in B-ALL cells. **A** Schematic overview of Ikaros isoforms 1 (IK1, full length) and 6 (IK6). Zinc finger domains F1-F4 are responsible for DNA binding, whereas the two C-terminal zinc finger domains F5 and F6 mediate dimerization (left). Model for the transcriptional regulation of SHIP1 by Ikaros (right). **B** Increased protein expression of SHIP1 after ectopic expression of the dominant negative IK6 in SupB15 cells, compared to control cells transduced with the empty vector (EV). **C** Ectopic expression of wildtype Ikaros resulted in reduced SHIP1 protein levels in SupB15 cells (left panel). SHIP1 levels were analysed in cells with and without SHIP1-knockdown and Ikaros-WT expression, respectively (right panel). **D** Model of the contrary regulation of the energy metabolism by Ikaros and PI3K/AKT/mTOR signaling (red: energy increase, green: energy deprivation). **E** Live cell imaging analysis after restoration of Ikaros-WT expression in combination with SHIP1-knockdown in SupB15 B-ALL cells. Cell proliferation was monitored over five days using a live cell imaging analysis platform. Unless otherwise stated, statistical significance refers to cells transduced with the scrambled shRNA and with the empty vector control (left bar). **F** Effects of Ikaros-WT restoration and SHIP1 expression on cell migration in boyden chamber analyses were shown. **G** Apoptosis induction in SupB15 cells with and without SHIP1-knockdown and Ikaros-WT expression was detected by Annexin-V-APC staining. **H** Hierarchical clustering (Euclidean distance) of significantly upregulated genes (*n* = 80; columns) for SHIP1-high / Ikaros-DN (dominant negative), SHIP1-high / Ikaros-WT, SHIP1-low / Ikaros-DN and SHIP1-low / Ikaros-WT expressing cells was carried out by gene set enrichment analysis using the transcriptome data of Ph-positive patient samples (*n* = 46; rows). Taken from the dataset of [http://www.stjuderesearch.org/data]

Dominant-negative isoforms of the transcription factor Ikaros (IKZF1) had been found in high degree in Ph-positive B-ALL cases [62] (Fig. 5A). Patients with an IKZF1 deletion were classified to high-risk groups [60]. The SupB15 cell line also expressed the dominant-negative Ikaros isoform 6 (IK6) [62]. It was therefore examined whether Ikaros and its dominant-negative isoform 6 had an influence on the expression of SHIP1 in B-ALL cells and on the cell growth. In the model for the transcriptional regulation of SHIP1 by Ikaros in B-ALL cells, SHIP1 possessed two Ets binding sites for binding Ikaros and Ikaros family members. Ikaros homodimers led to the repression of the INPP5D expression, while Ikaros dominant-negative isoforms were no longer able to maintain the inhibition of transcription.

After transduction of SupB15 cells with a lentiviral vector expressing the Ikaros 6 isoform, the cells showed a stronger SHIP1 expression and a higher phosphorylation of AKT-S473 in comparison to control cells (Fig. 5B). Subsequently, SupB15-SHIP1-knockdown and scrambled control cells were transduced again either with the empty vector or with the Ikaros-WT vector resulting in Ph-positive SupB15 cells with and without SHIP1-knockdown and Ikaros-WT expression. The restoration of the Ikaros-WT expression led to a significant reduction in the endogenous SHIP1 protein expression (Fig. 5C). Ikaros also reduced the phosphorylation of AKT at the serine residue 473 both in cells both with and without SHIP1-knockdown. However, SHIP1-knockdown cells had an increased expression of phosphorylated AKT-S473 compared to cells with high SHIP1 expression. After restoration of Ikaros-WT expression, a decrease of G6PD expression and an upregulation of phospho-AMPK-T172 was observed. Ikaros and the PI3K/AKT/mTOR signaling pathway had opposite functions in the regulation of the energy stress sensor AMPK as well as the glucose-metabolism effector G6PD and the glucose transport inhibitor TXNIP (Fig. 5D).

The growth behavior of Ph-positive SupB15 cells with and without SHIP1-knockdown and Ikaros-WT expression were monitored using live cell imaging (Fig. 5E). The simultaneous expression of SHIP1 and Ikaros-WT led to a strong reduction (56%) in the growth of SupB15 cells compared to double-transduced cells with the scrambled shRNA and with the empty vector control. SHIP1-knockdown cells showed significantly faster proliferation than SHIP1 expressing cells. In agreement with the reduced proliferation of SHIP1 and Ikaros-WT double positive SupB15 cells, these cells also showed a decreased migration and a high rate of apoptosis (Fig. 5F-G and Fig. S5), which indicated that both proteins, despite their highly different properties, were both fundamentally involved in the control of basic cellular processes.

To identify significantly upregulated genes of the different subtypes with and without SHIP1-knockdown and Ikaros-WT expression, a gene set enrichment analysis was performed using the transcriptome data of Ph-positive patient samples from the dataset of [http://www.stjuderesearch.org/data]. Therefore, the strength of the gene expression of INPP5D was divided into high and low. In addition, Ikaros status was divided into wildtype and dominant negative isoform (DN). Hierarchical clustering of top variably expressed protein coding genes led to three gene cluster dividing SHIP1-low/Ikaros-DN (cluster 1), SHIP1-low/Ikaros-WT (cluster 2) and SHIP1-high/Ikaros-DN as well as SHIP1-high/Ikaros-WT (cluster 3) (Fig. 5H; Table S11). Subsequently, gene ontology (GO) analysis was carried out. 50 out of 80 (62.5%) significantly upregulated genes were connected to cellular metabolic processes (FDR: 0.0015; GO:0044237). In summary, these data underlined the tumor suppressor function of SHIP1 and Ikaros in ALL cells.

In a protein kinase profiling assay the change in the activity of tyrosine and serine/threonine kinases was examined after the restoration of wildtype Ikaros expression in SupB15 cells (Fig. S6). The influence of Ikaros-WT reconstitution on the NTRK and Src-kinase subfamilies were particularly recognizable (Fig. S6A). The reconstitution of Ikaros-WT expression in the cell line SupB15 led to an increase (> 1 log2FC) in the activity of PRKCZ (PKC), JAK2, CHEK1 (CHK1) and PRKAA1 (AMPKa1) (Fig. S6B-D). In contrast, an increase (> 2 log2FC) in the activity of the Src kinase FGR was seen without Ikaros-WT expression. Among others, these genes were confirmed as significantly regulated by volcano plot visualization (Fig. S6E). The gene expression analysis of kinome-specific genes according to the classification of IKZF1 with high, intermediate or low expression in transcriptome data from B-ALL patient samples with Ph-positive (Fig. S6F) subtype showed that the genes PTK2B, CDK9, FGR, CSK, GSK3B, CHEK1 and CSNK2A2, together with IKZF1, exhibited a gradual change in gene expression from the low to high expression classification.

Moreover, the expression of SHIP1 was analysed in Ph-positive B-ALL cell lines (TOM-1 and SupB15) and Ph-positive CML cell lines in the blast crisis (Nalm-1 and BV173) (Fig. S7A). Nalm-1 and TOM-1 cells expressed the Ikaros isoform 1, while BV173 and SupB15 cells expressed the Ikaros isoform 6 [62]. A strong SHIP1 expression was also seen in the BV173 cell line. The Ikaros-WT restoration experiments were then repeated for this cell line. Here, restoration of Ikaros-WT expression led to an increase in the endogenous SHIP1 expression (Fig. S7B). In addition, the cellular proliferation after SHIP1-knockdown and/or ectopic restoration of Ikaros expression was analysed (Fig. S7C). As already observed for the SupB15 cells, the simultaneous expression of SHIP1 and Ikaros-WT led to a significant reduction in the proliferation of BV173 cells. The tumor suppressor function of Ikaros seemed again partially enhanced by an SHIP1-dependent, additive effect in this CML cell line. In comparison to the B-ALL cell line, SHIP1 was not downregulated here, but instead expressed to an increased extent.

### The role of casein kinase 2 on SHIP1 expression and AKT signaling in B-ALL cells

**Fig. 6:**
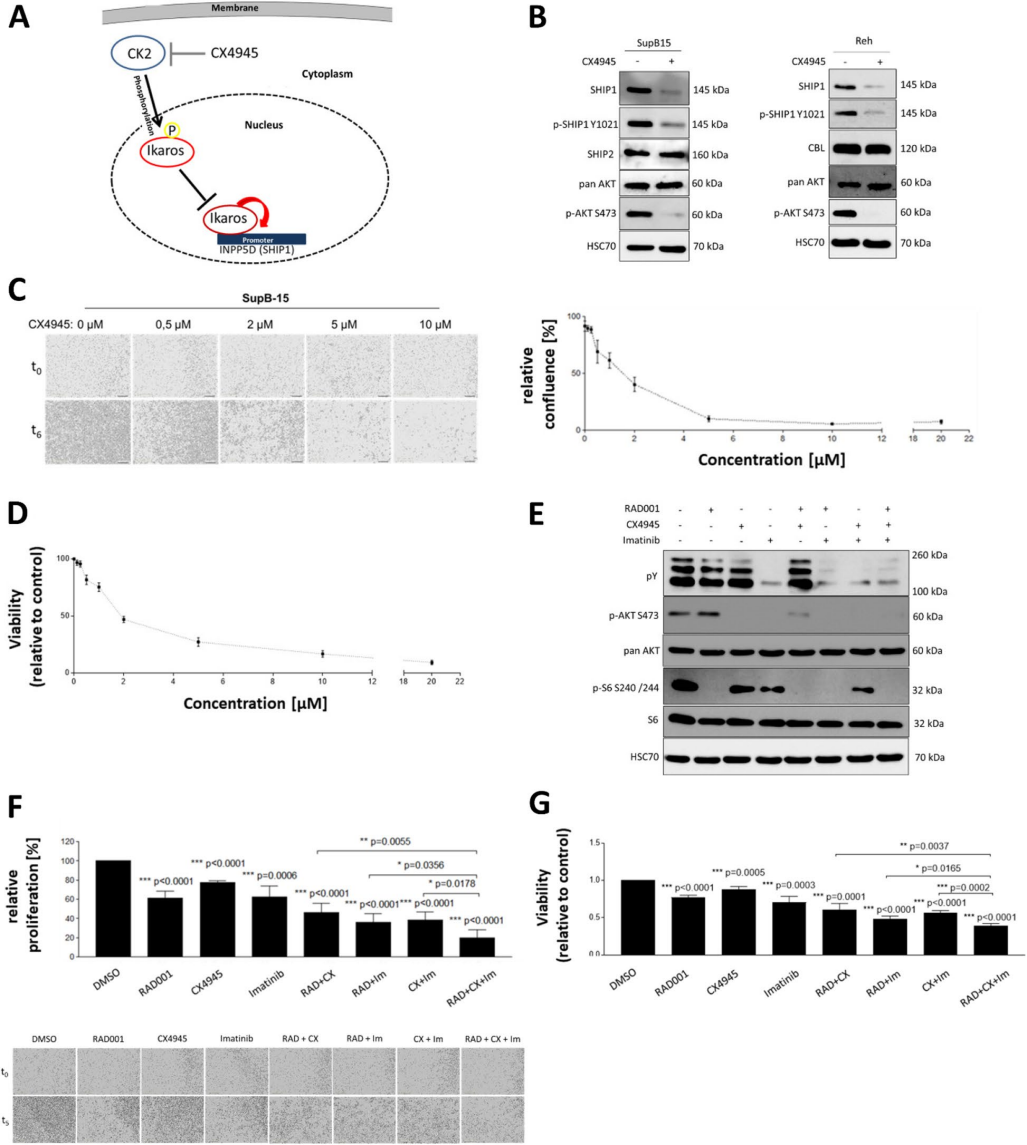
The role of casein kinase 2 on SHIP1 expression and AKT activation in B-ALL cells. **A** Model of how CK2 regulated the transcription factor Ikaros in B-ALL cells according to Dovat et al. [12, 76]. **B** Analysis of the effects of CK2 inhibition on SHIP1 protein levels and AKT S473 phosporylation in B-ALL cells. SupB15 and Reh cells were each treated with 10 μM CX4945 or DMSO for 24 h. **C** Proliferation of SupB15 cells following treatment with increasing concentrations of CX4945. Representative images before and after treatment for six days were shown. The relative confluence of the cells was shown. **D** In addition, cell viability/proliferation was determined using an Alamar blue assay. **E** Combined treatment of SupB15 cells with CK2-, mTOR- and BCR-ABL-inhibitors and its effects on AKT/S6 activation and cell proliferation. Cells were treated with 500 nM CX4945, 0.8 nM RAD001, 250 nM imatinib or combinations thereof. For Western Blot experiments, inhibitors were applied for 48 h. **F** Anti-mitogenic effects were evaluated by live cell imaging after treating cells for five days. Representative live cell images were shown below. The statistical significance always relates to the DMSO-treated cells. **G** In addition, cell viability/proliferation was determined using an Alamar blue assay

The casein kinase 2 (CK2) had been described as a negative regulator of Ikaros activity in B-cells (Fig. 6A) and prevented the binding of Ikaros to the DNA [75]. To address the functional role of CK2 in Ikaros and SHIP1 regulation in B-ALL, SupB15 and Reh cells were treated with the CK2 inhibitor CX4945 (Fig. 6B). The treatment with CX4945 led to a strong reduction in SHIP1 expression (> 90%) in both cell lines. In comparison to the DMSO-treated cells, the inhibition of CK2 also drastically reduced the phosphorylation of AKT at serine 473. In contrast, the overall expression of pan AKT was not affected. The cells showed a significant effect on growth and viability at a concentration of 0.5 µM and an IC50 value of 1.53 µM and 1.83 µM, respectively (Fig. 6C and D).

Constitutively activated receptor tyrosine kinases (RTK) or oncogenic tyrosine kinases (e.g. BCR-ABL), which mimic activated RTK signaling, lead to a constitutive activation of the PI3K/AKT/mTOR signaling pathway. Since previous data showed that the CK2 inhibitor resulted in a similar protein expression profile as seen in the SupB15 cells after restoration of Ikaros-WT expression, we used it for a combinatorial therapy approach. Due to the increased phosphorylation of AKT in SupB15 cells, the specific inhibitors against CK2 (CX4945) and mTOR (RAD001) in combination with the BCR-ABL inhibitor imatinib were used to investigate a potential combinatorial strategy to downregulate activated AKT signaling in Ph-positive B-ALL (Fig. 6E). RAD001 inhibition led to a weak increase in the phosphorylation of AKT, but was the only inhibitor used that had the capacity to decrease phosphorylation of S6. The inhibition with CX4945 led to a decrease in AKT phosphorylation, but showed only a small effect on reducing the phosphorylation of S6. Imatinib treatment had a small inhibitory effect on S6 phosphorylation, but was able to block AKT phosphorylation and to generally reduce the level of tyrosine-phosphorylated proteins. In consequence, triple inhibition had a strong negative effect on the phosphorylation of both, AKT and S6 as well as on the cellular content of phospho-tyrosines. Moreover, the growth of the cells was analysed by live cell imaging (Fig. 6F). All inhibitor treatments resulted in a significant reduction of cell proliferation. The inhibitor combinations showed significantly stronger anti-proliferative effects than the individual inhibitors alone. The strongest inhibitory effect was observed with the triple combination of RAD001, CX4945 and imatinib. The results were validated using an Alamar blue viability/proliferation assay (Fig. 6G).

### All three isoforms contributed to AKT signaling and hyperactivation-induced negative selection in B-ALL cells

**Fig. 7:**
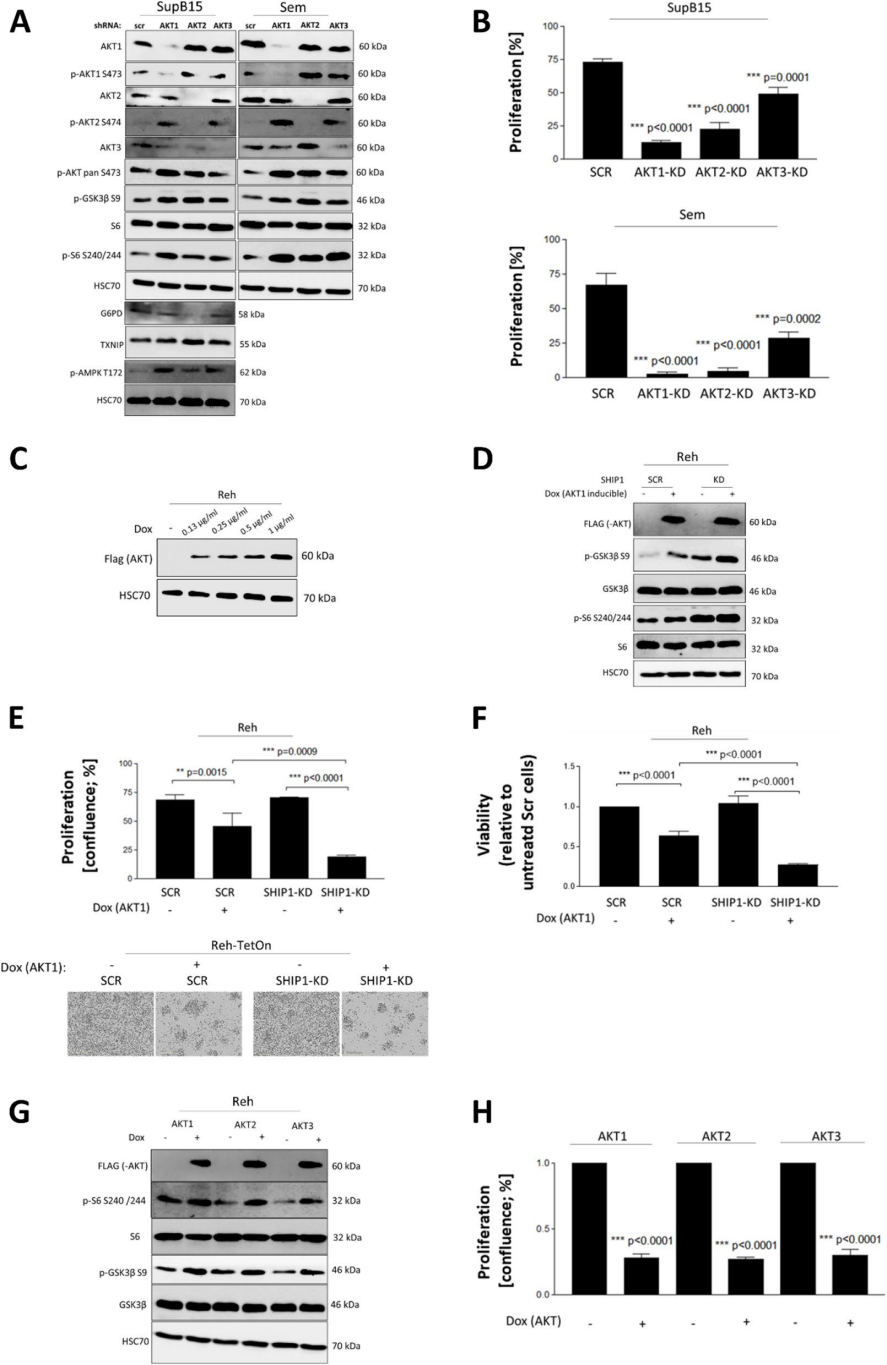
Functional role of individual AKT isoforms on the signal transduction of B-ALL cells. **A** The AKT signaling pathway was analysed at the protein level after shRNA-mediated knockdown of AKT isoforms 1, 2 and 3 in B-ALL cell lines SupB15 and Sem. **B** AKT knockdown cells were analysed for cell proliferation. **C** Doxycycline-inducible expression of a constitutively active mutant of AKT1 (T308D/S473D, AKT1-DD) in Reh cells. Establishment of the inducible expression system by applying increasing doxycycline concentrations and detection of induced expression of the AKT1-DD mutant with an anti-FLAG antibody. **D** Biochemical and (**E**) biological effects of the inducible AKT1 hyperactivation in SHIP1-negative (KD) or positive (scr) Reh cells were evaluated by Western Blot and proliferation assay, respectively. **F** In addition, cell viability/proliferation was determined using an Alamar blue assay. **G** Doxycycline-inducible expression of constitutively active DD mutants of all three AKT isoforms (AKT 1, 2 and 3) in Reh cells following treatment with 1 µg/mL Doxycycline. The impact of AKT hyperactivation was analysed by Western Blot and (**H**) in proliferation assays

The PI3K/AKT/mTOR signaling pathway was found to be constitutively activated in many solid cancers and the majority of hematologic malignancies, including ALL. The kinase AKT is an essential downstream target of PI3K and itself has the capacity to promote growth and survival of tumorigenic cells. Three AKT isoforms (AKT1, AKT2 and AKT3) were identified in mammals, some of which were thought to have differential or even unique functions [17, 79]. We used the transcriptome data of Ph-positive patient samples [http://www.stjuderesearch.org/data] and divided the gene expression of AKT1, 2 and 3 into high and low by forming the tercile of the gene set. Indeed, we identified specific and overlapping genes between the three isoforms (Fig. S8; Table S12-S18). Interestingly, isoform-specific AKT-knockdown cells, which were established through RNA interference using isoform-specific shRNAs, showed different effects on the phosphorylation of the AKT-dependent signaling proteins GSK3β and S6 as well as proteins of the energy metabolism (G6PD, TXNIP and AMPK) (Fig. 7A and B). In addition, the knockdown of AKT isoforms led to an increased phosphorylation of the remaining AKT isoforms. The knockdown of each AKT isoform alone resulted in a significant reduction in cell proliferation in Sem and SupB15 cells. Interestingly, inhibition of proliferation was much more pronounced after knockdown of either, AKT1 or AKT2 suggesting that both AKT1 and AKT2, but not AKT3 were primarily involved in the transduction of pro-mitogenic signals in the two ALL cell lines (Fig. 7B).

Moreover, we established an AKT isoform-specific doxycycline-inducible expression system (TetOn system) in Reh cells (Fig. 7C). To test, whether cells with targeted knockdown of SHIP1 react more sensitive to an hyperactivation of the AKT signaling pathway, Reh cells were transduced with doxycycline-inducible constitutive active AKT (T308D/S473D; DD) isoforms. The induced expression of mutant AKT1 was detected by a FLAG-tag in the Western blot (Fig. 7D). Hyperactivation of the AKT signaling pathway after induced expression of the AKT1-DD mutant was demonstrated by an increase in the phosphorylation of both, GSK3β and S6. Importantly, expression of the AKT1-DD mutant in SHIP1-KD Reh cells led to an even stronger activation of the AKT cascade than either expression of AKT1-DD or knockdown of SHIP1 alone (compare Fig. 7D, lane 4 with lanes 2 and 3). Moreover, hyperactivated AKT1 signaling in Reh cells reversed the pro-mitogenic and anti-apoptotic AKT signaling to the negative (Fig. 7E and F). Next, we analysed the influence of all three constitutively active AKT-isoforms on the signal transduction and negative selection of Reh cells. As shown in Fig. 7G-H, each single AKT isoform inhibited cell proliferation and induced cell death by hyperactivation of the AKT signaling.

## Discussion

Acute lymphoblastic leukemia (ALL) is the most common cancer and the most common cause of cancer-related death in childhood. Despite significantly improved chances of cure, the prognosis remains poor, especially for patients in a high-risk group (e.g., Ph-positive B-ALL) and patients with relapse. For instance, up to 20% of patients with ETV6-RUNX1 had been shown to relapse [43]. In addition, the effects of intensive chemotherapy often lead to the occurrence of secondary tumors.

One major problem in the therapy is the strong heterogeneity of childhood ALL. Up to now over 20 subtypes had been described based on cytogenetic analysis, immunophenotyping and gene expression profiles [3]. The strong differences between individual ALL subtypes make a general therapeutic strategy more difficult and had led to subtype-specific treatment approaches [46]. We showed that the inositol-5-phosphatase SHIP1 was differentially expressed across all ALL subtypes. SHIP1 protein was highly upregulated in pediatric ETV6-RUNX1 and pediatric BCR-ABL positive B-ALL cells, while it had been shown to be strongly down-regulated in B-ALL cells with KMT2A-rearrangement and T-ALL cells [9, 18, 20, 52]. As previously shown, SHIP1 expression had been highly downregulated in cells with CML and myeloproliferative neoplasm (MPN) [25, 70]. The protein expression of SHIP1 was also significantly increased in 8 out of 12 (~ 67%) primary and patient-derived B-ALL samples analysed (Fig. 1). We must note that the selection of CD19-positive cells may only reflect a subset of the B-cell population, as exemplified by the emergence of tumor escape variants with B-cell antigen loss (e.g. CD19) in relapsed disease [57]. However, samples with relapsed disease were not used here. At the molecular level, CD19 is involved in the activation of PI3K signaling. In this context, selection of CD19-positive cells could alter intracellular signaling.

**Fig. 8:**
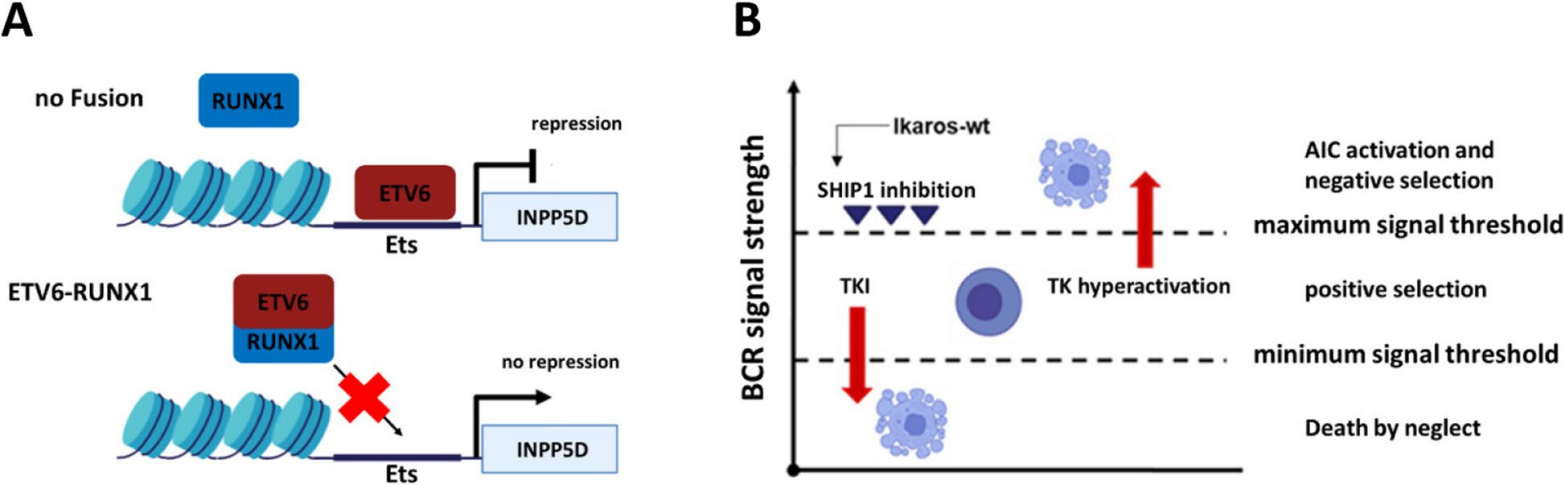
Model of SHIP1 regulation in ETV6-RUNX1-positive B-ALL cells and implication of SHIP1 in clonal B-cell-selection. **A** ETV6 binds to the Ets binding site of SHIP1 and represses its expression. After fusion to RUNX1 in ETV6-RUNX1, ETV6 can no longer bind to the SHIP1 promoter. As a result, transcription of SHIP1 is not further suppressed. **B** Implication of SHIP1 in the model of AIC activation and negative selection of B-cell clones adopted from [9]. We introduced into this model the negative regulation of SHIP1 expression by Ikaros in B-ALL cells. According to this, the Ikaros status (WT versus DN) is an important factor for AIC activation, at least partially by dynamic regulating SHIP1 expression. Emphasize that the thresholds for AIC activation can be cell-specific, depending on the individual activity of the kinome and phosphatome of the cell

Moreover, our study revealed that the group of ETV6-RUNX1 positive B-ALL had a significantly higher amount of SHIP1 mRNA compared to the group of ETV6-RUNX1-negative B-ALL (Fig. 1F). In agreement with our data, the dataset of Kohlmann et al. showed markedly increased SHIP1 expression in ETV6-RUNX1-positive B-ALL and in BCR-ABL-positive B-ALL, when compared to B-ALL with other translocations than BRC-ABL as well as in comparison to healthy hematopoietic cells [41] (Fig. S1A). ETV6 belongs to the Ets family of transcription factors [84]. Direct regulation of SHIP1 mRNA expression by ETV6 has not yet been demonstrated. However, one reason why SHIP1 expression was increased, particularly in the ETV6-RUNX1-ALL, could be that the fusion with RUNX1 prevented the ETV6 transcription factor from binding to the Ets binding site in the INPP5D locus, thus abolishing the ETV6-mediated repression of SHIP1 expression (Fig. 8A). This hypothesis was supported by the finding that FLI1, another member of the Ets transcription factor family, had been shown to bind directly to the Ets binding site within the INPP5D promoter, thus directly blocking SHIP1 transcription [44].

Mutations of the INPP5D gene in patients with acute lymphoblastic B-cell leukemia were rare. However, ALL patients with a SHIP1-ABL1 fusion protein had been described [36, 66]. As a result, the fusion protein could both act as an activated tyrosine kinase and influence the normal SHIP1 function in a dominant-negative manner. A detailed analysis of the dataset from Chouvarine and coworkers [10] showed that several SHIP1 fusion proteins were found in the B-other cohort (Figure S1B). These fusion proteins should be examined in more detail in future. An INPP5D-ATG16L1 fusion transcript had been observed before [23]. We therefore suggested here that the regulation of SHIP1 expression is an important mechanism in leukemic cells to change the cellular phenotype. It seemed to be a more common event than INPP5D gene mutation. This finding was supported by the strong differences in the expression of SHIP1 between Ph-positive B-ALL and CML, which had been previously shown by Chen et al. [9]. The differences could not be explained by the expression of BCR-ABL alone, since inhibitor treatment with imatinib led to upregulation of SHIP1 in both cell lines. While more than 95% of patients with chronic myeloid leukemia had achieved long-term disease-free survival after treatment with tyrosine kinase inhibitors [13], patients with Ph-positive B-ALL had relapsed very quickly within a short period of time after initial remission [35]. Moreover, deep molecular profiling recently revealed distinct transcriptomic subtypes of BCR-ABL-positive lymphoblastic leukemia [40]. Taken together, this had suggested that a multilineage and a lymphoid gene expression profile of the BCR-ABL subtype could be observed. In contrast to CML, loss of Ikaros by deletion or mutation had been found in 80% of BCR-ABL-positive B-ALL cases and 29% of BCR-ABL-negative B-ALL high-risk patients [62, 63]. Recently, it had been shown that the transcription of SHIP1 was strongly repressed by Ikaros in the chicken DT40 B-cell line [1]. In contrast, Song et al. had shown that Ikaros upregulated SHIP1 transcription in human Nalm-6 B-ALL cells (DUX4-r) [76]. It had been shown that Ikaros itself became directly inactivated by a post-translational modification induced by CK2-dependent phosphorylation. Inhibition of the constitutively activated PI3K/AKT/mTOR signaling pathway with a triple combination of small molecule inhibitors targeting mTOR, BCR-ABL and CK2 resulted in a highly significant and potent inhibition of cellular growth of SupB15 B-ALL cells (Fig. 6).

Indeed, SHIP1 had an antagonistic influence on the B-cell receptor downstream signal strength, as demonstrated by the reduced activation of the AKT signaling cascade in B-ALL cells with high SHIP1 expression. One strong reason for the increased SHIP1 expression detected in some malignant B-ALL cells could be that the cells had a survival advantage in clonal B-cell selection due to the functional implication of SHIP1 to downregulate overactivated BCR signaling events [9]. A more detailed analysis showed that the restoration of Ikaros wildtype expression reduced the strong SHIP1 expression to a moderate level, thereby lowering the AIC threshold to escape stringent negative selection processes and led to a significant reduction in cell growth and increased apoptosis (Fig. 5). Hence, it was shown in this study for the first time that Ikaros tumor suppressor function was particularly enhanced by SHIP1. We therefore concluded that the Ikaros status in B-ALL was an important factor for AIC activation, in part through SHIP1 regulation (Fig. 8B).

In line with this, our gene expression analysis revealed that INPP5D exhibited a substantial influence on the energy metabolism and cellular stress response in B-ALL cells (Fig. 5 and Fig. S3). Compared to myeloid cells, the AMP / ATP ratios in B-cells had been shown to be significantly higher [6, 7]. In addition, oncogenic kinases consumed ATP and increased the energy requirement of pre-malignant B-cells. Thus, ATP restriction served as a safeguard against the elimination of these cells. IKZF1 had been shown to regulate genes associated with glucose transport (e.g. NR3C1, AMPK and TXNIP) and limited the glucose and energy supply in B-cells to values that were not sufficient for malignant transformation [6, 50, 56, 83]. Other groups had demonstrated that phosphatases could also stabilize energy reserve and, thus prevented negative selection of the cell [9, 71, 72]. Expression of the dominant-negative Ikaros isoform can fundamentally alter the B-cell-specific framework and result in a different gene expression profile, as shown by Iacobucci and colleagues [33]. Terminal differentiation to plasma cells had been shown to result in fundamental changes in key characteristics of the B-cell lineage, including Ikaros expression, and may serve as a mechanism to escape negative selection [32, 38].

In the present model, the B-cell-specific negative selection against cells with hyperactive tyrosine kinase signaling could be undermined by the dominant-negative Ikaros isoform and could raise the threshold being able to induce cell-intrinsic apoptosis. In a recent study, it had been shown that the dominant-negative Ikaros isoform had protected ALL cells from apoptosis by manipulating the AKT/FoxO1 axis [28]. In summary, our and other’s results pointed to a regulatory window for the expression level of SHIP1, which depends partly on Ikaros and must neither be undercut (e.g., in T-ALL and CML) nor exceeded (e.g. in Ph-positive B-ALL and ETV6-RUNX1-positive B-ALL). The regulatory function of Ikaros and CK2 on SHIP1 expression in B-ALL highlights the relevance of the AKT pathway as a therapeutic target for the treatment of this disease.

### Supplementary Information

Additional file 1: Figure S1. Differential INPP5D expression in B-ALL subtypes. (A) Analysis of INPP5D expression across leukemia subtypes from the gene expression microarray data GSE13204 [41]. INPP5D mRNA expression was compared between different subtypes and to healthy and non-malignant bone marrow controls. (B) Occurrence of INPP5D gene fusions in B-other subtypes. INPP5D gene fusions were listed on the right side. Data were taken from the publicly available dataset of [10]. (C) Establishment of SHIP1-specific knockdown in B-ALL cell lines Reh and SupB15. Two different SHIP1-shRNAs were tested for their knockdown efficiency. Figure S2. Effects of SHIP1-knockdown on the activity profile of cellular kinases in SupB15 cells. (A) Schematic representation of functional kinome profiling on whole cell extracts. (B) Analysis of the influence of SHIP1-knockdown on the activity profile of tyrosine and serine/threonine kinases in BCR-ABL-positive SupB15 cells by kinome tree visualization. The influence on the family of tyrosine kinases (TK) and calcium/calmodulin-dependent protein kinases (CAMK) was shown enlarged. The Tec-kinase BTK was particularly recognizable here. Branch and node color showed the kinase statistic of each kinase after knockdown of SHIP1. Blue indicated a negative regulation and red indicated a positive regulation after SHIP1-knockdown. The node size visualized the specificity score of each kinase after knockdown of SHIP1. (C-E) showed the highest impact of the SHIP1-knockdown (“top list”) on the activity profile of tyrosine and serine/threonine kinases involved in overall (C), receptor tyrosine kinase (RTK, D) and PI3K/AKT signaling. The length of the individual bars corresponded to the strength of the change in phosphorylation. (F) Volcano plot visualization of the effect of SHIP1-knockdown on the activity profile of tyrosine and serine/threonine kinases in SupB15 cells. Significantly regulated kinases were shown in red (dots). Figure S3. Gene set enrichment analysis for INPP5D-high and -low expression in Ph-positive and ETV6-RUNX1 B-ALL subtypes. The strength of INPP5D gene expression was divided into high and low and a subsequent gene set enrichment analysis was carried out in patient samples with Ph- (A) and ETV6-RUNX1-positive (B) B-ALL subtypes. Gene sets with a FDR q-val ≤ 0.25 were considered significant and presented in a bar chart with the Normalized Enrichment Score (NES) value. Identical genes were marked with an asterisk. (C) Gene expression analysis of kinome-specific genes according to the classification of INPP5D with low, intermediate or high gene expression by transcriptome data from B-ALL patient samples with a Ph-positive subtype. In the heatmap analysis, high and low expressed genes were shown in red and blue, respectively. Genes that showed a gradual change in their gene expression pattern from low to high together with INPP5D were marked with an asterisk. (D) Venn diagram of significantly enriched genes for INPP5D expression in the Ph- (blue) and ETV6-RUNX1-positive (red) subtype. Both subtypes were compared for similarities and differences in gene sets. Gene set enrichment analysis of high versus low INPP5D expressing samples was carried out. Gene ontology (GO) analysis of genes correlated with INPP5D in the Ph-positive and ETV6-RUNX1 subtype was performed. Identical genes were marked with an asterisk. Shared genes were further subdivided according to their function as driver, oncogene or tumor suppressor. The genes were further subdivided into groups of transcription factors (E) and surface markers (F). Common genes were listed below in grey rectangle. Gene expression data were taken from the publicly available dataset of [http://www.stjuderesearch.org/data]. Figure S4. Representative flow cytometry gating strategy for analysis of SHIP1-positive cell populations. The intact lymphocyte gate was analyzed depending on distinguished FSC vs. SSC properties. The cell doublets were excluded. The relative proportion of human SHIP1-expressing cells in spleen and liver was analysed after staining with an FITC-conjugated anti-hCD45 antibody and an APC-conjugated anti-hSHIP1 antibody for the scrambled control (above) and the SHIP1-knockdown cohort (below). Figure S5: Representative flow cytometry gating strategy for analysis of apoptosis of genetically modified Ph-positive SupB15 cells. (A)The lymphocyte gate was analyzed depending on distinguished FSC vs. SSC properties. The cell doublets were excluded. Apoptotic cells were identified by Annexin V staining. Figure S6. Kinase activity profiles after reconstitution of Ikaros-WT in SupB15 cells. (A) Kinome tree visualization of the differential kinase activity profiles after reconstitution of Ikaros-WT expression. The impact on the family of tyrosine kinases (TK) and calcium/calmodulin-dependent protein kinases (CAMK) was shown enlarged. The NTRK and Src-kinase subfamilies were particularly recognizable. Branch and node color showed the statistic of each kinase after reconstitution of Ikaros-WT. Blue indicated a negative and red indicated a positive change after Ikaros-WT reconstitution. The node size visualized the specificity score. (B–D) showed the highest impact (“top list”) on the activity profile of tyrosine and serine/threonine kinases involved in overall (B), RTK (C) and PI3K/AKT signaling (D) after reconstitution of Ikaros-WT. The length of the individual bars corresponded to the strength of the change in phosphorylation. The length of the individual bars corresponded to the strength of the change in phosphorylation. (E) Volcano plot visualization of the kinome activity profiling data. Significantly regulated kinases were shown in red (dots). (F) Gene expression analysis of kinome-specific genes after categorizing IKZF1 expression into low, intermediate or low expressing groups. Data were taken from transcriptome analysis of Ph-positive B-ALL patient samples [http://www.stjuderesearch.org/data]. High and low expressed genes were shown in the heatmap analysis in red and blue, respectively. Genes displaying a gradual change in their expression from low to high together with IKZF1 were marked with an asterisk. Figure S7: SHIP1 expression was upregulated by Ikaros in the Ph-positive CML cell line BV173 cells. (A) Expression of SHIP1 and AKT in human Ph-positive cell lines. Two ALL (TOM-1 and SupB15) and two CML cell lines in blast crisis (Nalm-1 and BV173) were analysed. (B) To investigate the influence of SHIP1 and Ikaros-WT on the signal transduction of Ph-positive CML cells, BV173 control and BV173-SHIP1-knockdown cells were transduced with lentiviral particles containing the empty vector and the Ikaros-WT vector, respectively. The different cells were analysed at the protein level and the SHIP1 expression was quantified relative to the double transduced cells with the scrambled shRNA and with the empty vector control (right panel). (C) Effects of SHIP1-knockdown and ectopic expression of Ikaros-WT on cell proliferation. Representative images were shown (left). The confluence was determined at day five after seeding. Figure S8. Venn diagram of AKT isoform-associated gene expression profiles in Ph-positive B-ALL patients. The strength of the gene expression of AKT1, 2 and 3 was categorized separately into high and low, followed by a gene set enrichment analysis. AKT isoform-specific and overlapping genes were identified and assigned to the three isoforms. Data were extracted from the publicly available dataset of [http://www.stjuderesearch.org/data].

Additional file 2.

## Data Availability

No datasets were generated or analysed during the current study.

## Abbreviations

PtdIns(3, 4, 5)P_3_: Phosphatidylinositol-(3, 4, 5)-trisphosphate
PtdIns(3, 4)P_2_: Phosphatidylinositol-(3, 4)-bisphosphate
SHIP1: Src homology 2 domain-containing inositol phosphatase 1
Ras / Raf / Mek / Erk: Rat sarcoma / rapidly accelerated fibrosarcoma / mitogen-activated protein kinase /extracellular signal-related kinase
Grb2 / Sos: Growth factor receptor-bound protein 2 / son of sevenless
JAK / STAT: Janus kinase / signal transducers and activators of transcription
PI3-kinase: Phosphoinositide 3-kinase/PI3K
AKT: Protein kinase B/PKB
mTOR: Mechanistic target of rapamycin
PTEN: Phosphatase and tensin homolog
AIC: Autoimmunity checkpoints
ROS: Reactive oxygen species
BCR: B-cell receptor
