# Supplementary material for: Ikaros sets the threshold for negative B-cell selection by regulation of the signaling strength of the AKT pathway
Source: Cell Commun Signal. 2024 Jul 12;22:360. doi: 10.1186/s12964-024-01732-5 (PMC11241878; doi:10.1186/s12964-024-01732-5)
Supplement: Supplementary file 1 — Additional file 1: Figure S1. Differential INPP5D expression in B-ALL subtypes. (A) Analysis of INPP5D expression across leukemia subtypes from the gene expression microarray data GSE13204 [41]. INPP5D mRNA expression was compared between different subtypes and to healthy and non-malignant bone marrow controls. (B) Occurrence of INPP5D gene fusions in B-other subtypes. INPP5D gene fusions were listed on the right side. Data were taken from the publicly available dataset of [10]. (C) Establishment of SHIP1-specific knockdown in B-ALL cell lines Reh and SupB15. Two different SHIP1-shRNAs were tested for their knockdown efficiency. Figure S2. Effects of SHIP1-knockdown on the activity profile of cellular kinases in SupB15 cells. (A) Schematic representation of functional kinome profiling on whole cell extracts. (B) Analysis of the influence of SHIP1-knockdown on the activity profile of tyrosine and serine/threonine kinases in BCR-ABL-positive SupB15 cells by kinome tree visualization. The influence on the family of tyrosine kinases (TK) and calcium/calmodulin-dependent protein kinases (CAMK) was shown enlarged. The Tec-kinase BTK was particularly recognizable here. Branch and node color showed the kinase statistic of each kinase after knockdown of SHIP1. Blue indicated a negative regulation and red indicated a positive regulation after SHIP1-knockdown. The node size visualized the specificity score of each kinase after knockdown of SHIP1. (C-E) showed the highest impact of the SHIP1-knockdown (“top list”) on the activity profile of tyrosine and serine/threonine kinases involved in overall (C), receptor tyrosine kinase (RTK, D) and PI3K/AKT signaling. The length of the individual bars corresponded to the strength of the change in phosphorylation. (F) Volcano plot visualization of the effect of SHIP1-knockdown on the activity profile of tyrosine and serine/threonine kinases in SupB15 cells. Significantly regulated kinases were shown in red (dots). Figure S3. Gene set enr [file 12964_2024_1732_MOESM1_ESM.pdf]

Figure 1G

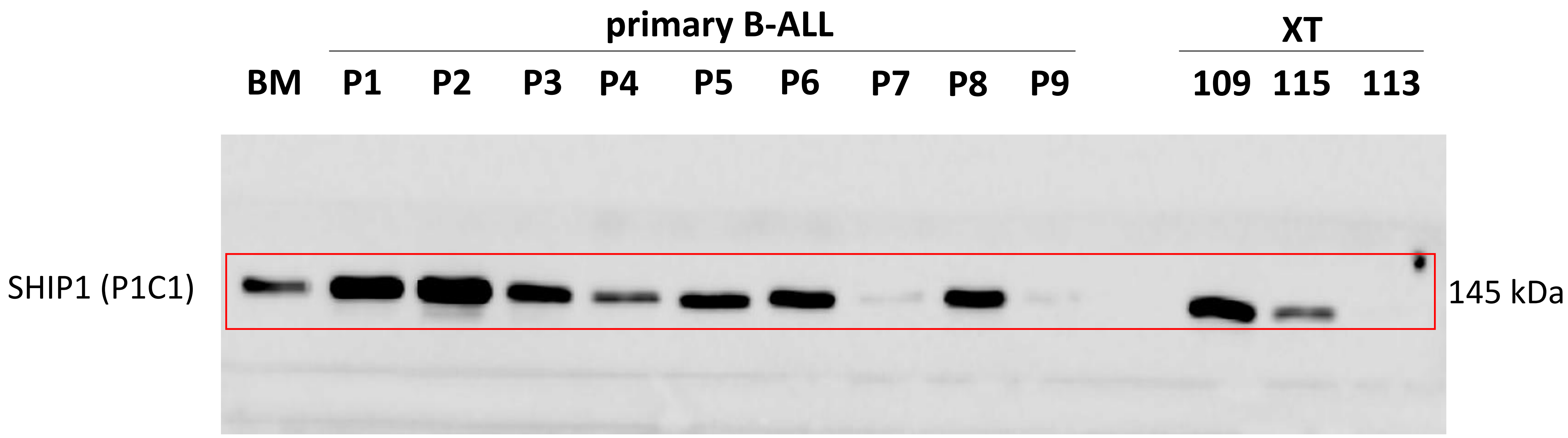
primary B-ALL
XT
BM
P1
P2
P3
P4
P5
P6
P7
P8
P9
109
115
113
SHIP1 (P1C1)
145 kDa

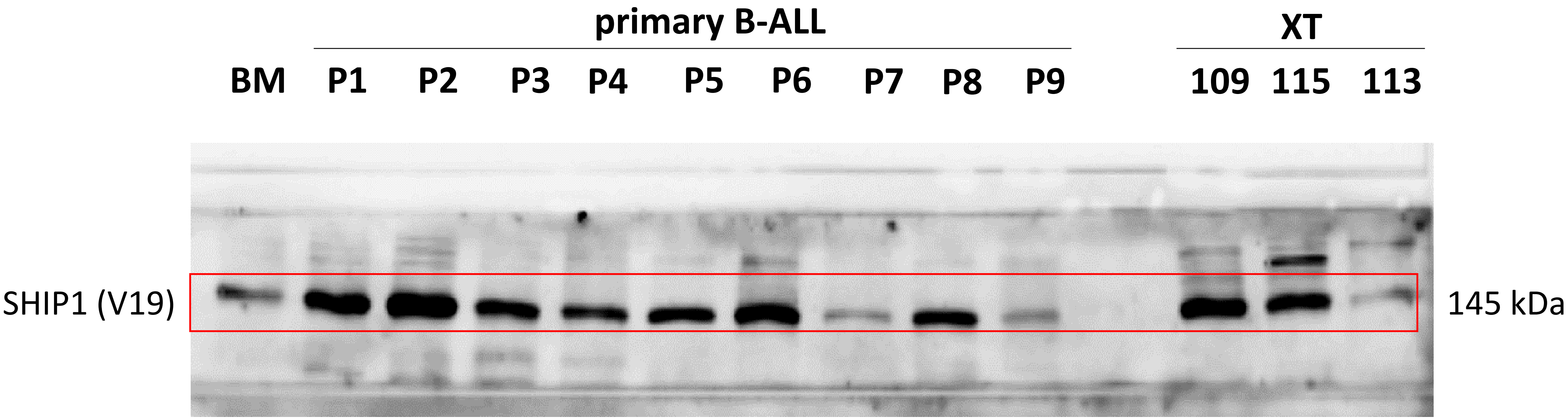
primary B-ALL
XT
BM
P1
P2
P3
P4
P5
P6
P7
P8
P9
109
115
113
SHIP1 (V19)
145 kDa

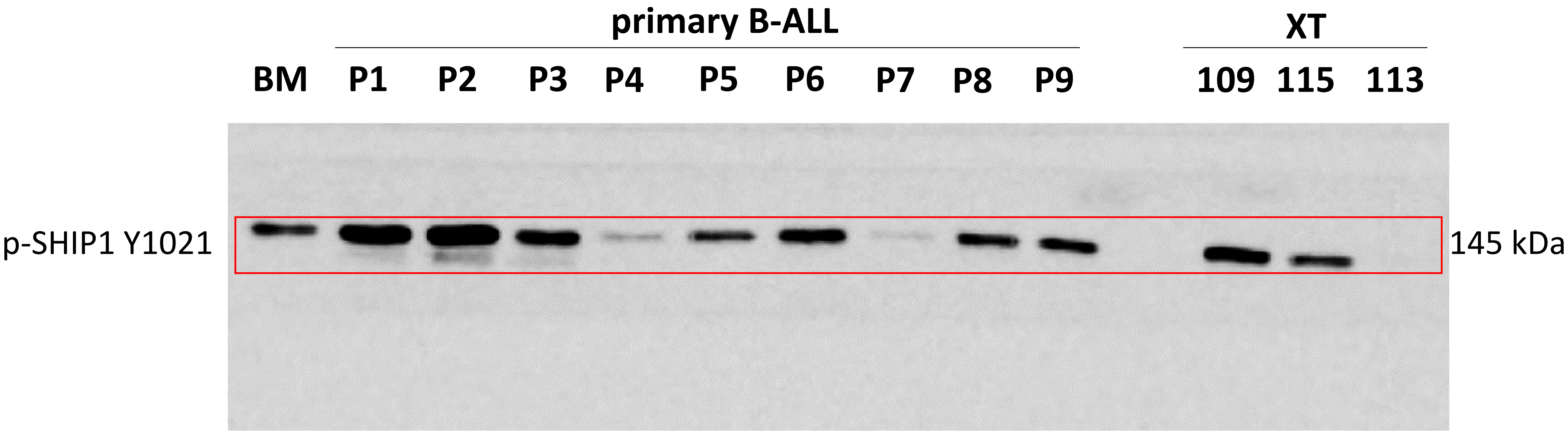
primary B-ALL
XT
BM
P1
P2
P3
P4
P5
P6
P7
P8
P9
109
115
113
p-SHIP1 Y1021
145 kDa

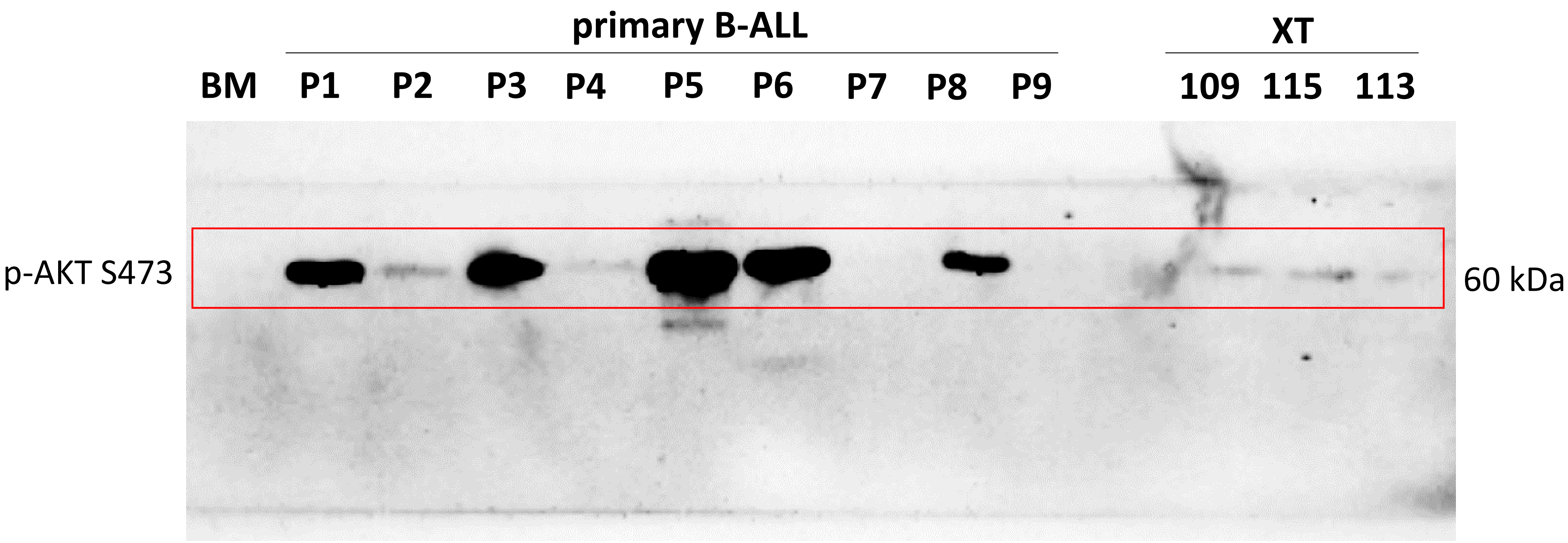
primary B-ALL
XT
BM
P1
P2
P3
P4
P5
P6
P7
P8
P9
109
115
113
p-AKT S473
60 kDa

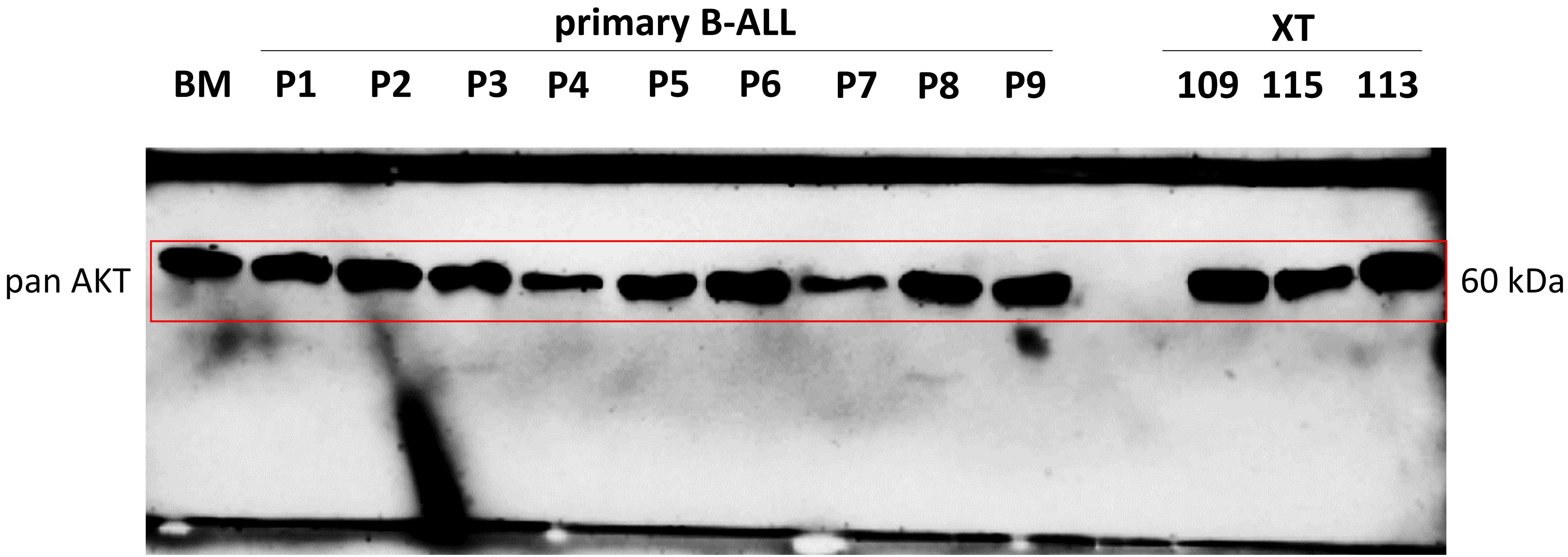
primary B-ALL
XT
BM
P1
P2
P3
P4
P5
P6
P7
P8
P9
109
115
113
pan AKT
60 kDa

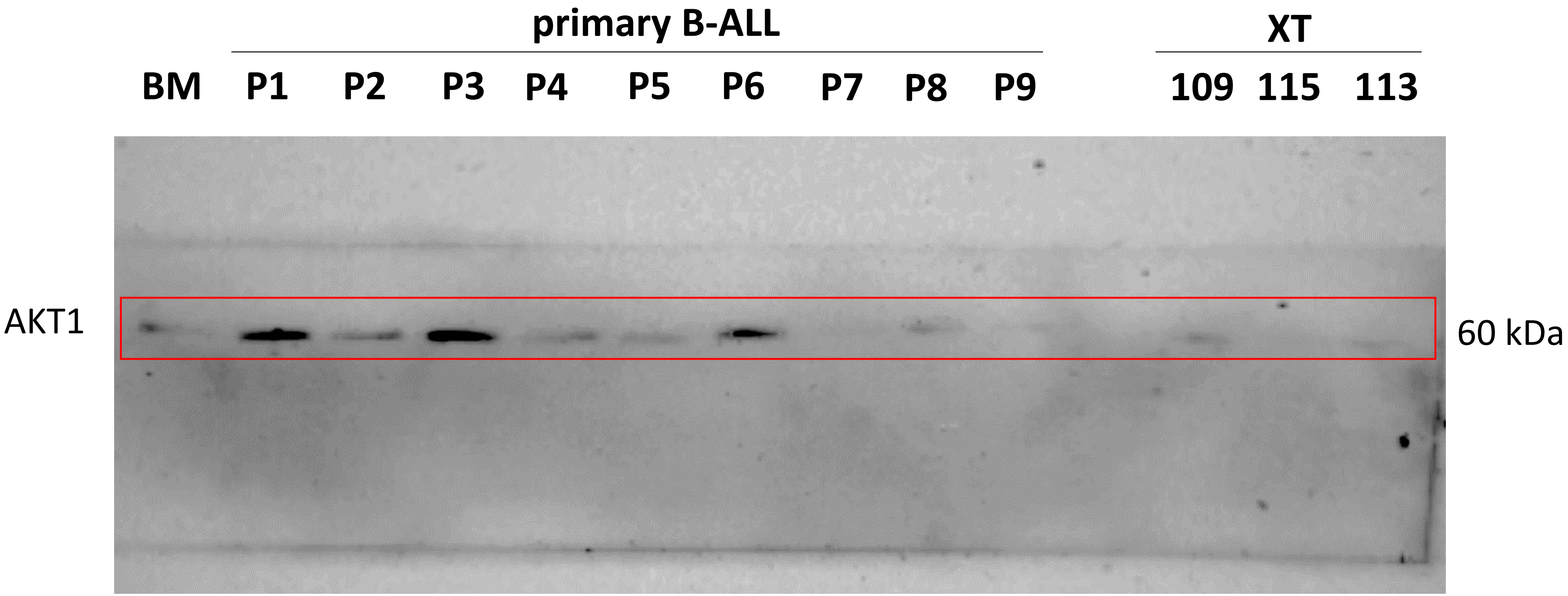
primary B-ALL
XT
BM
P1
P2
P3
P4
P5
P6
P7
P8
P9
109
115
113
AKT1
60 kDa

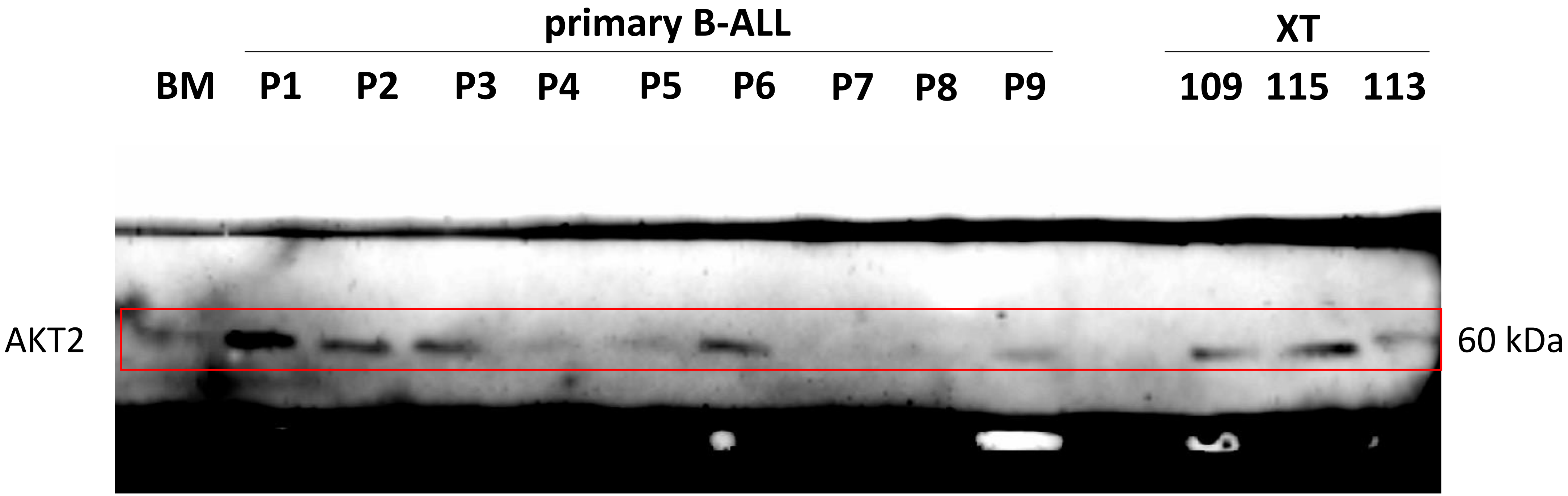
primary B-ALL
XT
BM
P1
P2
P3
P4
P5
P6
P7
P8
P9
109
115
113
AKT2
60 kDa

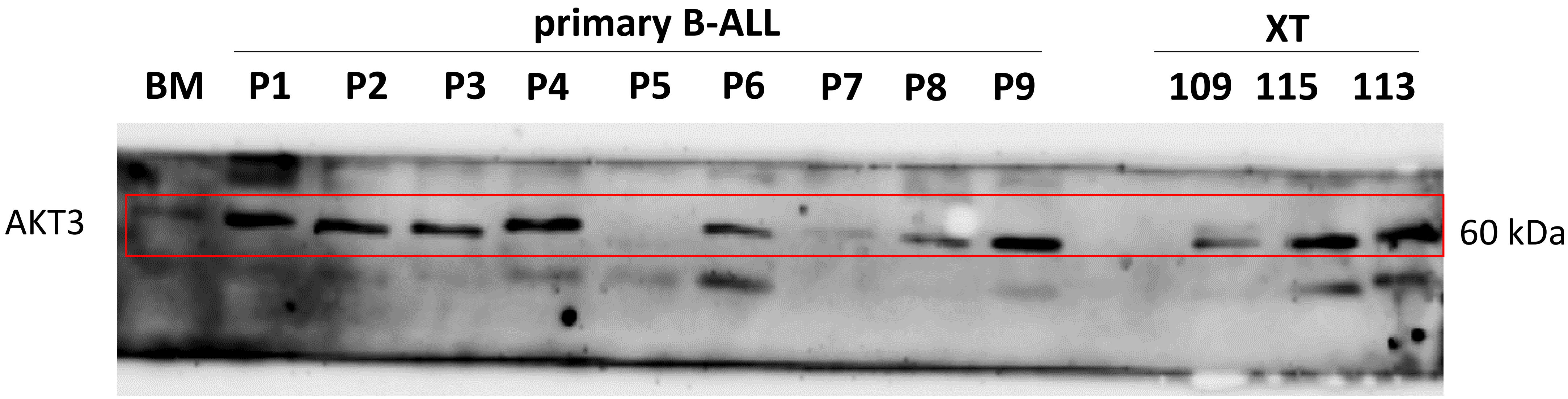
primary B-ALL
XT
BM
P1
P2
P3
P4
P5
P6
P7
P8
P9
109
115
113
AKT3
60 kDa

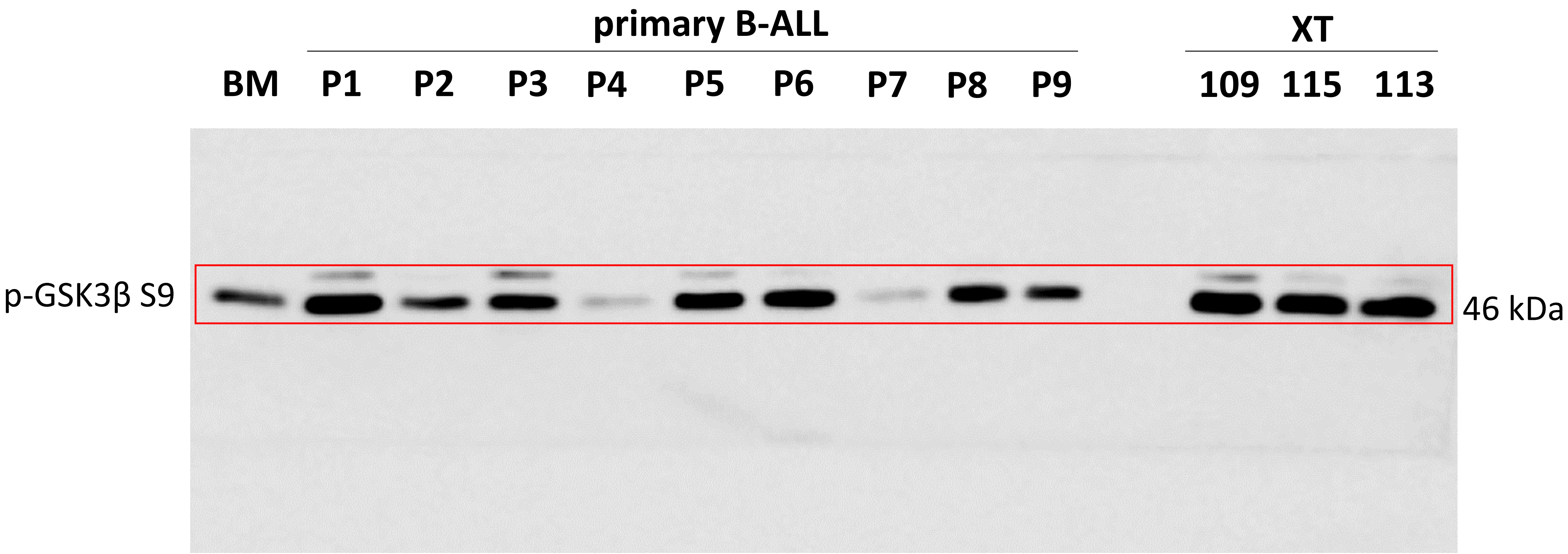
primary B-ALL
XT
BM
P1
P2
P3
P4
P5
P6
P7
P8
P9
109
115
113
p-GSK3β S9
46 kDa

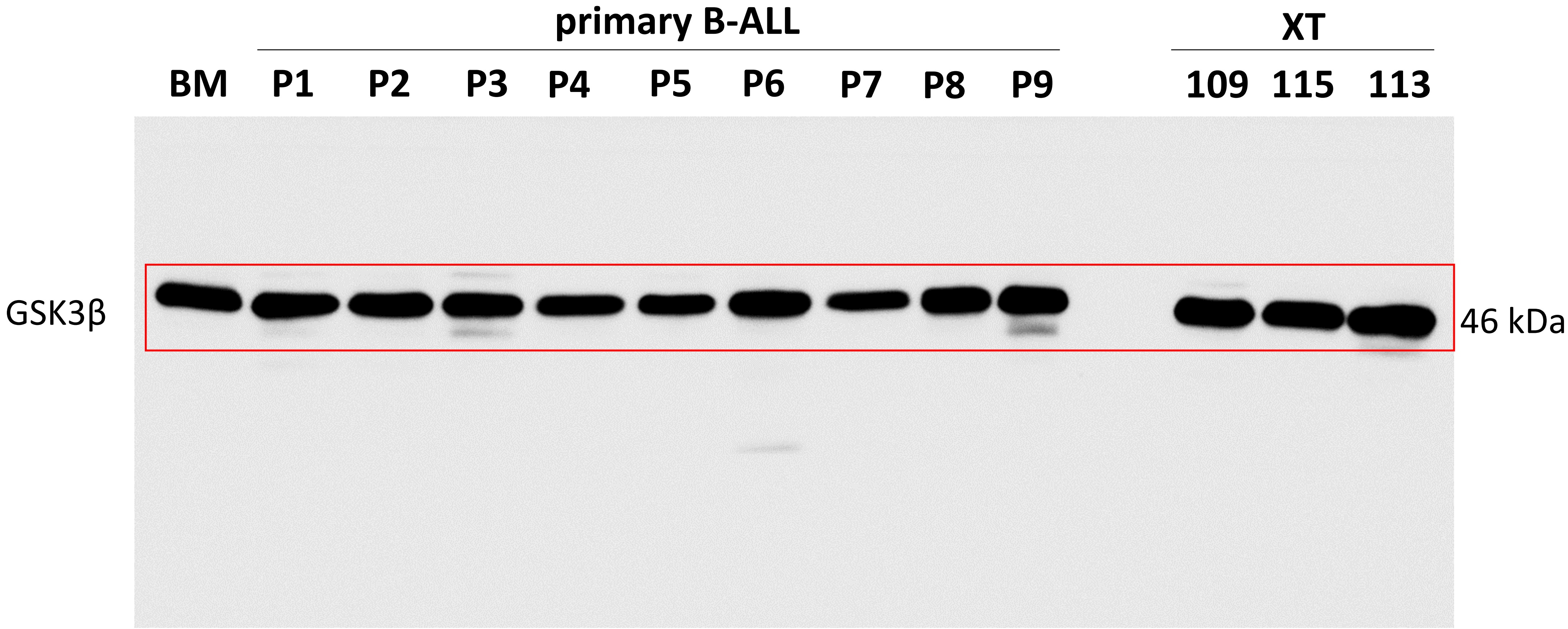
primary B-ALL
XT
BM
P1
P2
P3
P4
P5
P6
P7
P8
P9
109
115
113
GSK3β
46 kDa

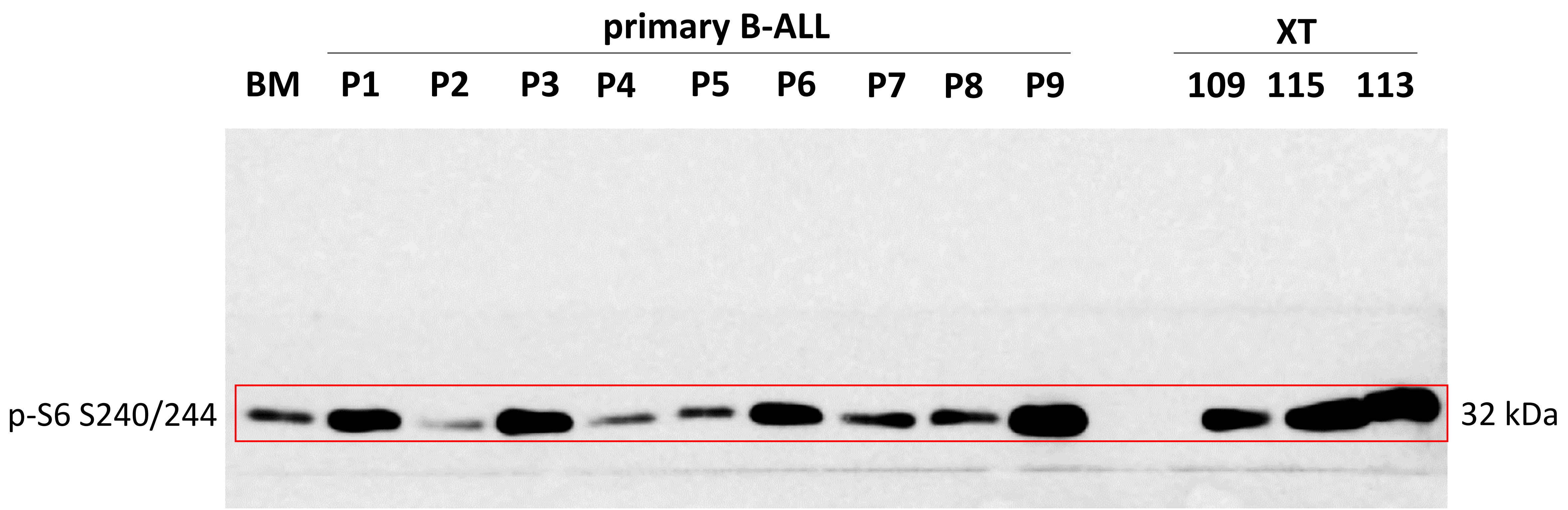
primary B-ALL
XT
BM
P1
P2
P3
P4
P5
P6
P7
P8
P9
109
115
113
p-S6 S240/244
32 kDa

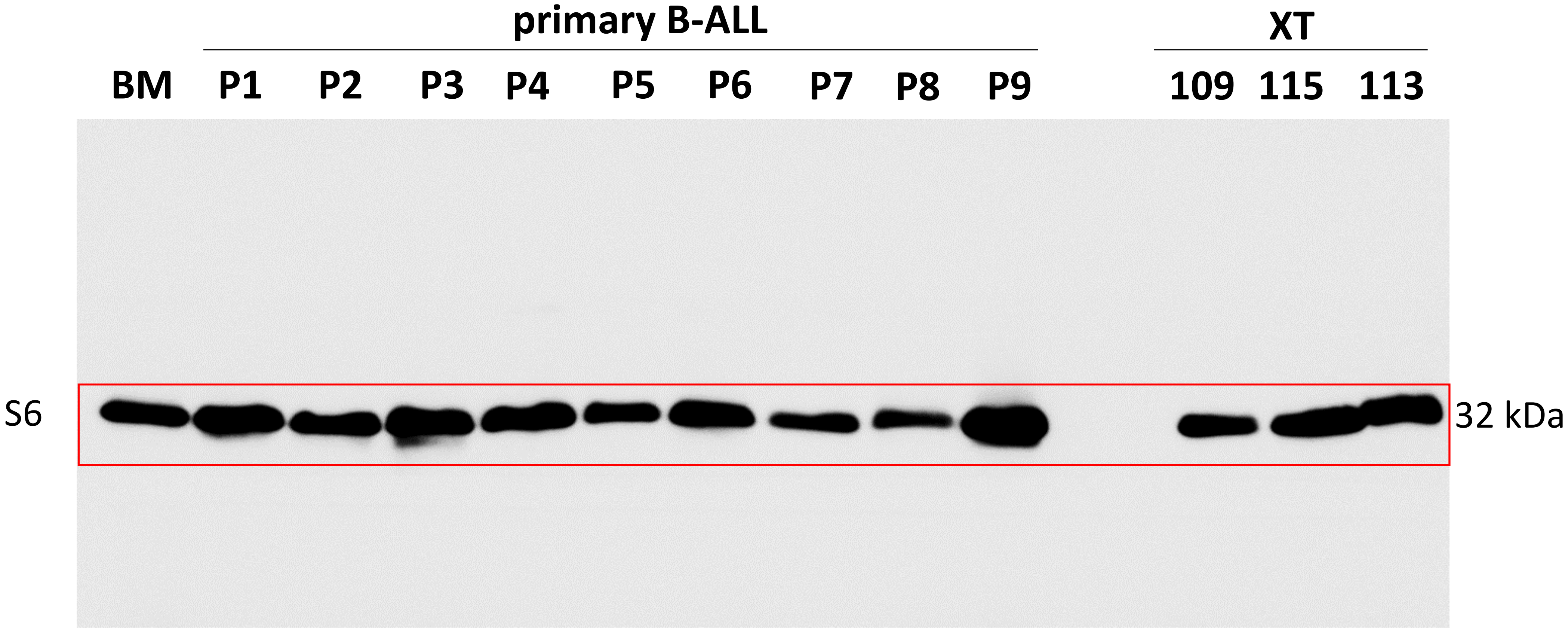
primary B-ALL
XT
BM
P1
P2
P3
P4
P5
P6
P7
P8
P9
109
115
113
S6
32 kDa

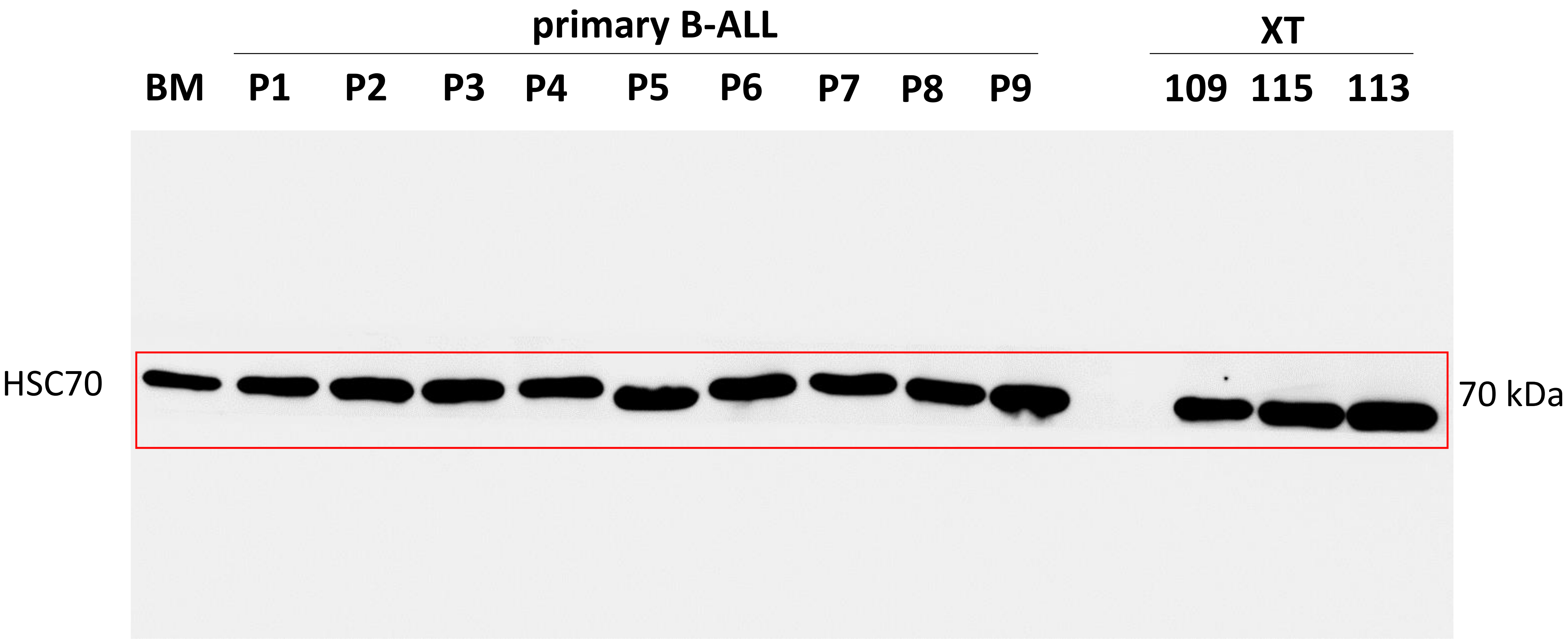
primary B-ALL
XT
BM
P1
P2
P3
P4
P5
P6
P7
P8
P9
109
115
113
HSC70
70 kDa

Figure 1H

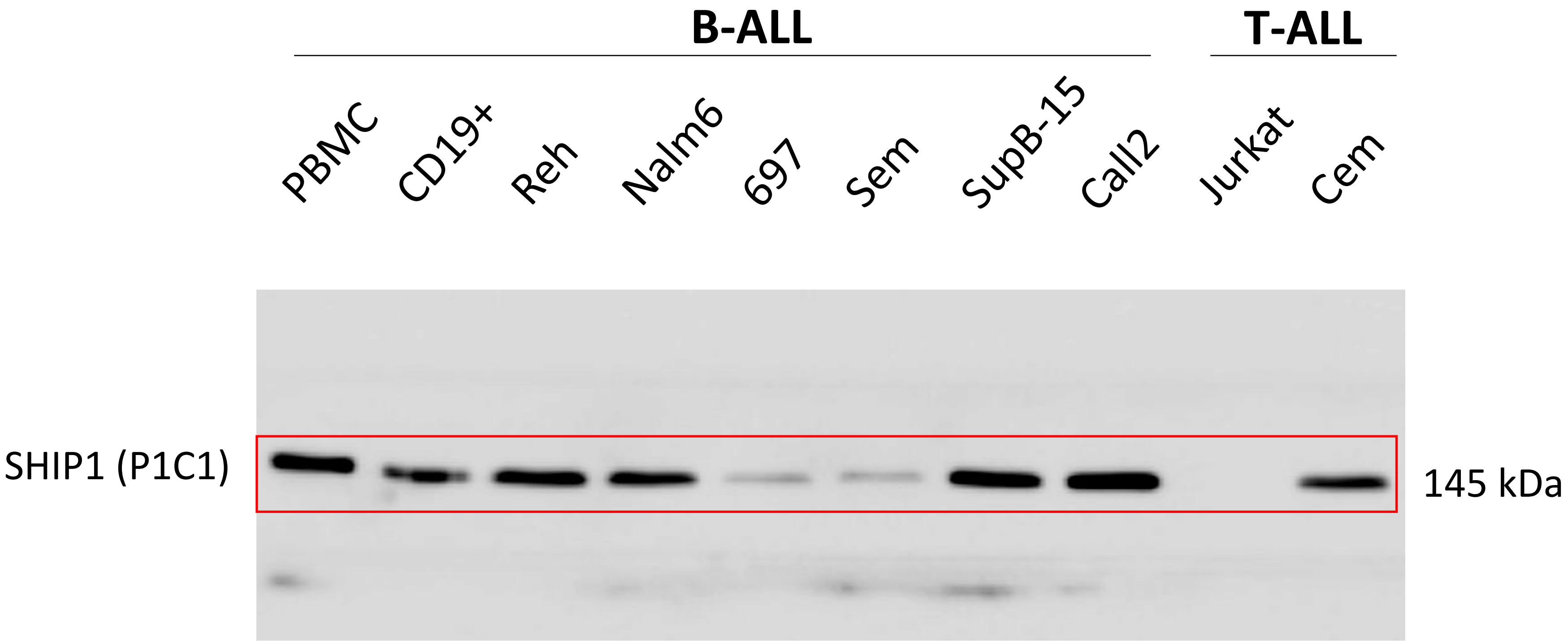
B-ALL
T-ALL
PBMC
CD19+
Reh
Nalm6
697
Sem
SupB-15
Call2
Jurkat
Cem
SHIP1 (P1C1)
145 kDa

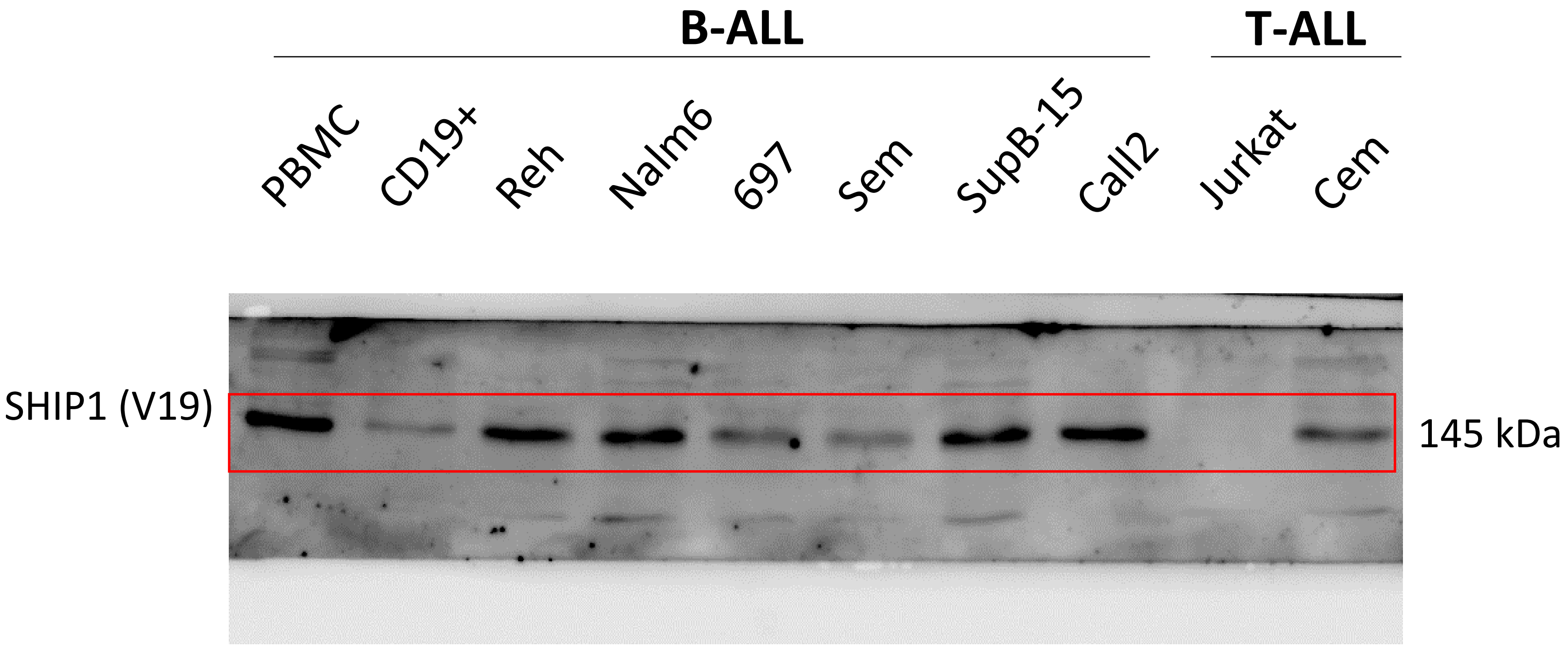
B-ALL
T-ALL
PBMC
CD19+
Reh
Nalm6
697
Sem
SupB-15
Call2
Jurkat
Cem
SHIP1 (V19)
145 kDa

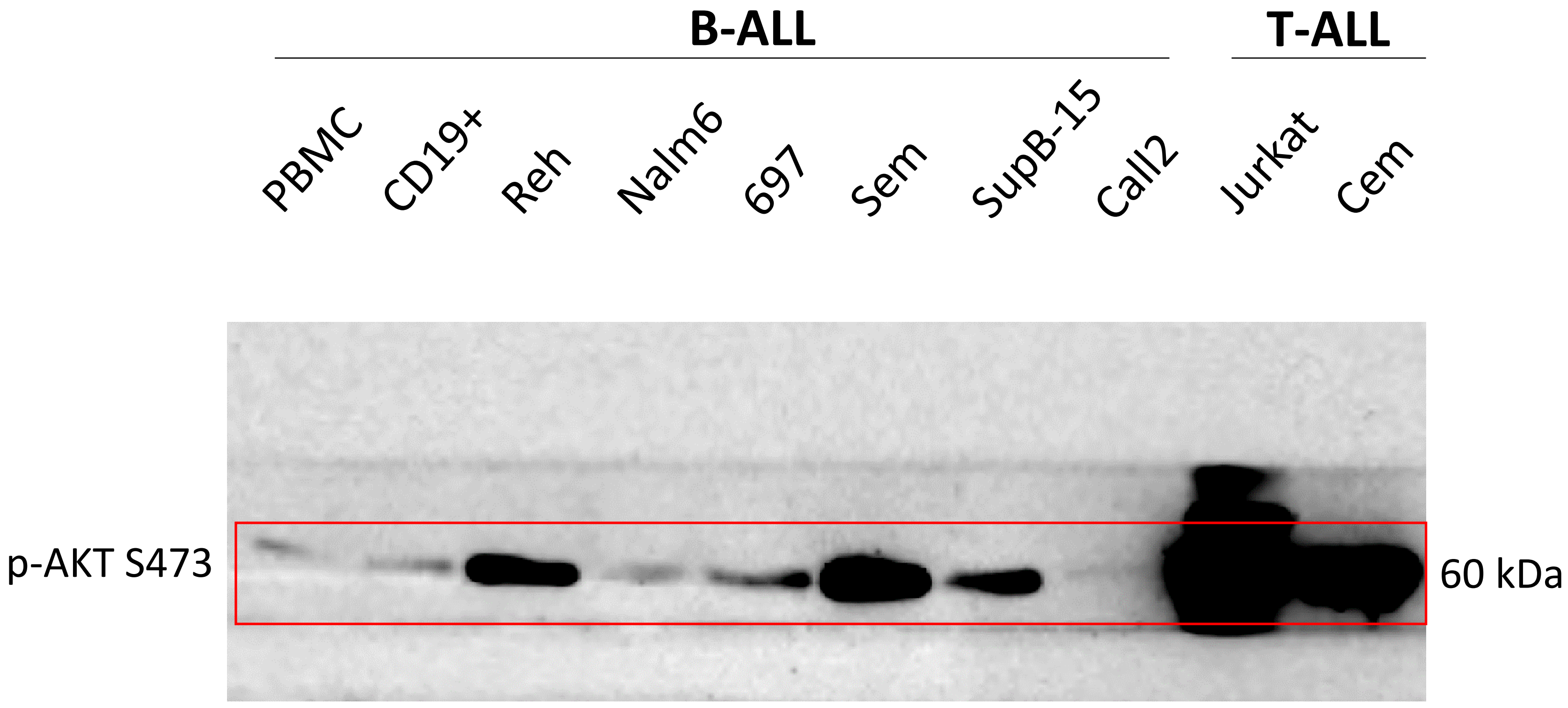
B-ALL
T-ALL
PBMC
CD19+
Reh
Nalm6
697
Sem
SupB-15
Call2
Jurkat
Cem
p-AKT S473
60 kDa

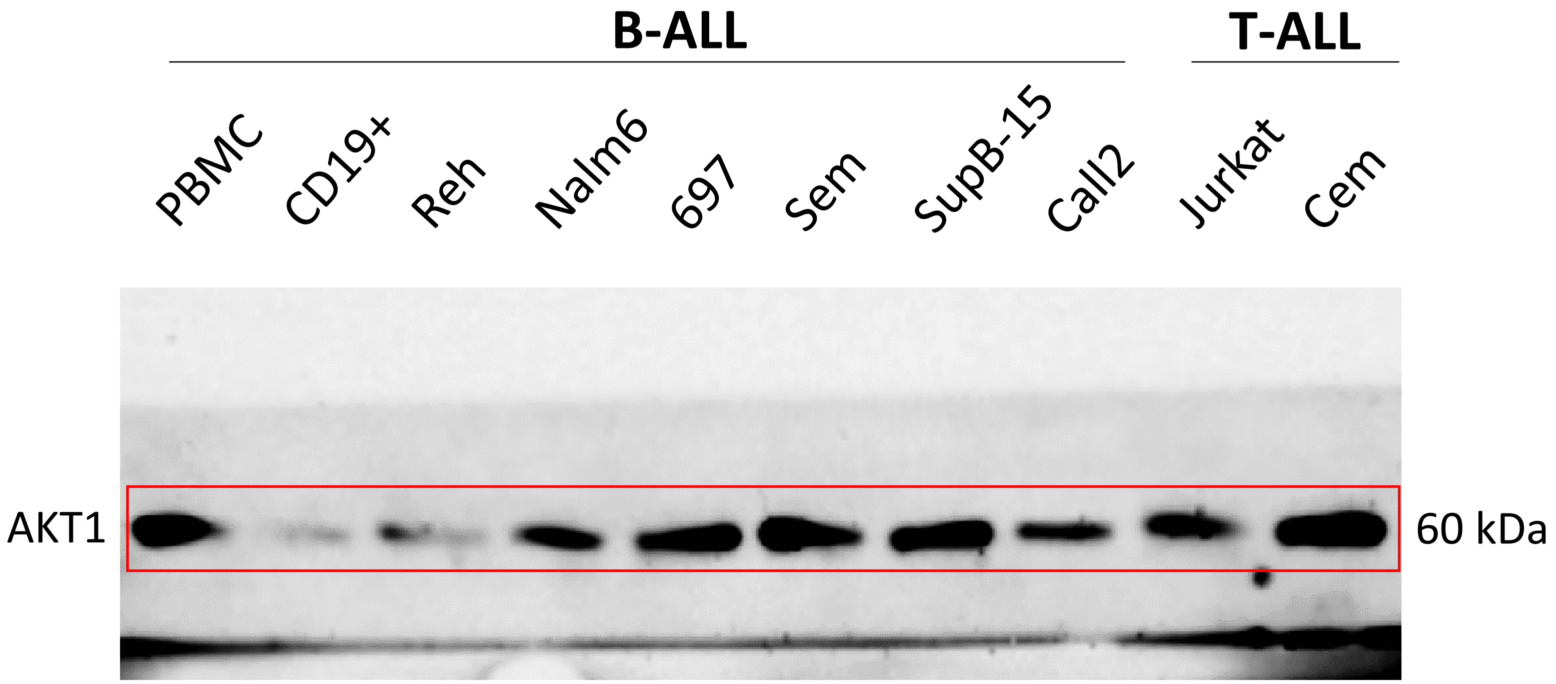
B-ALL
T-ALL
PBMC
CD19+
Reh
Nalm6
697
Sem
SupB-15
Call2
Jurkat
Cem
AKT1
60 kDa

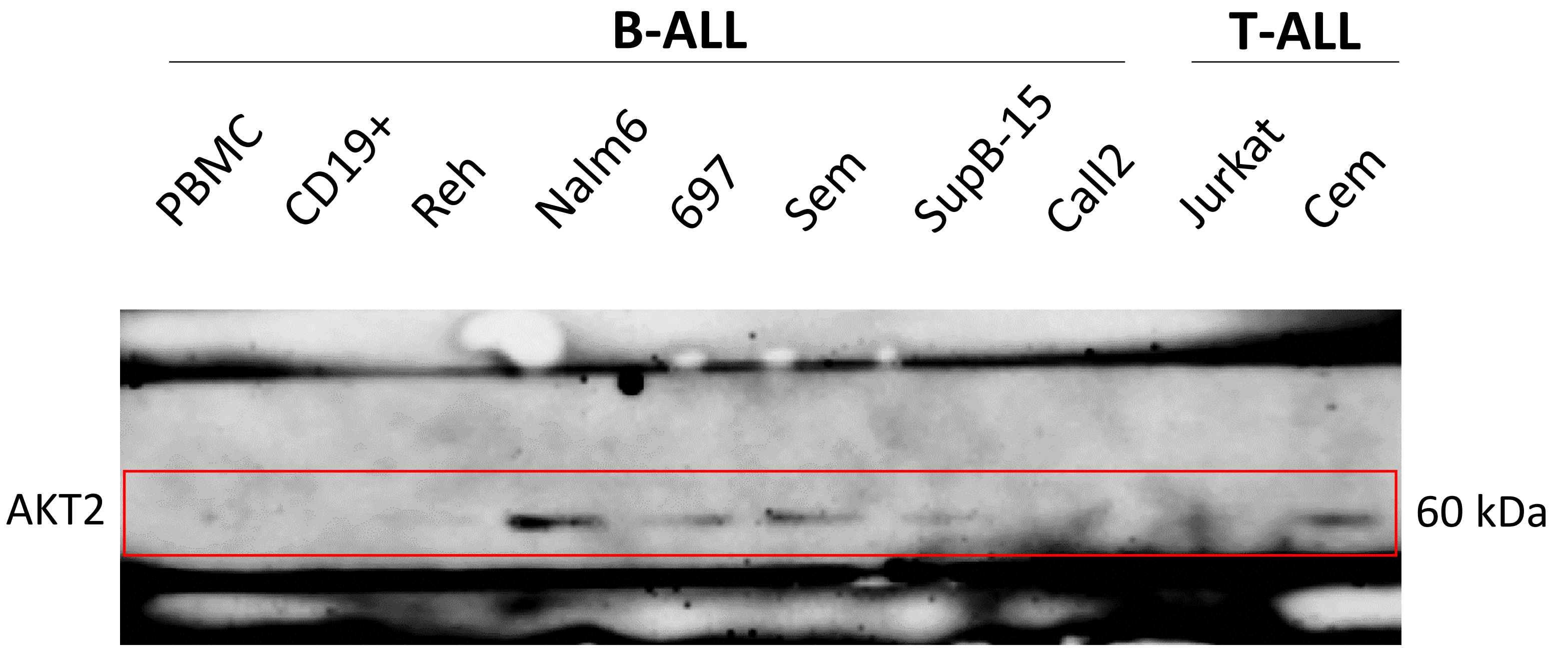
B-ALL
T-ALL
PBMC
CD19+
Reh
Nalm6
697
Sem
SupB-15
Call2
Jurkat
Cem
AKT2
60 kDa

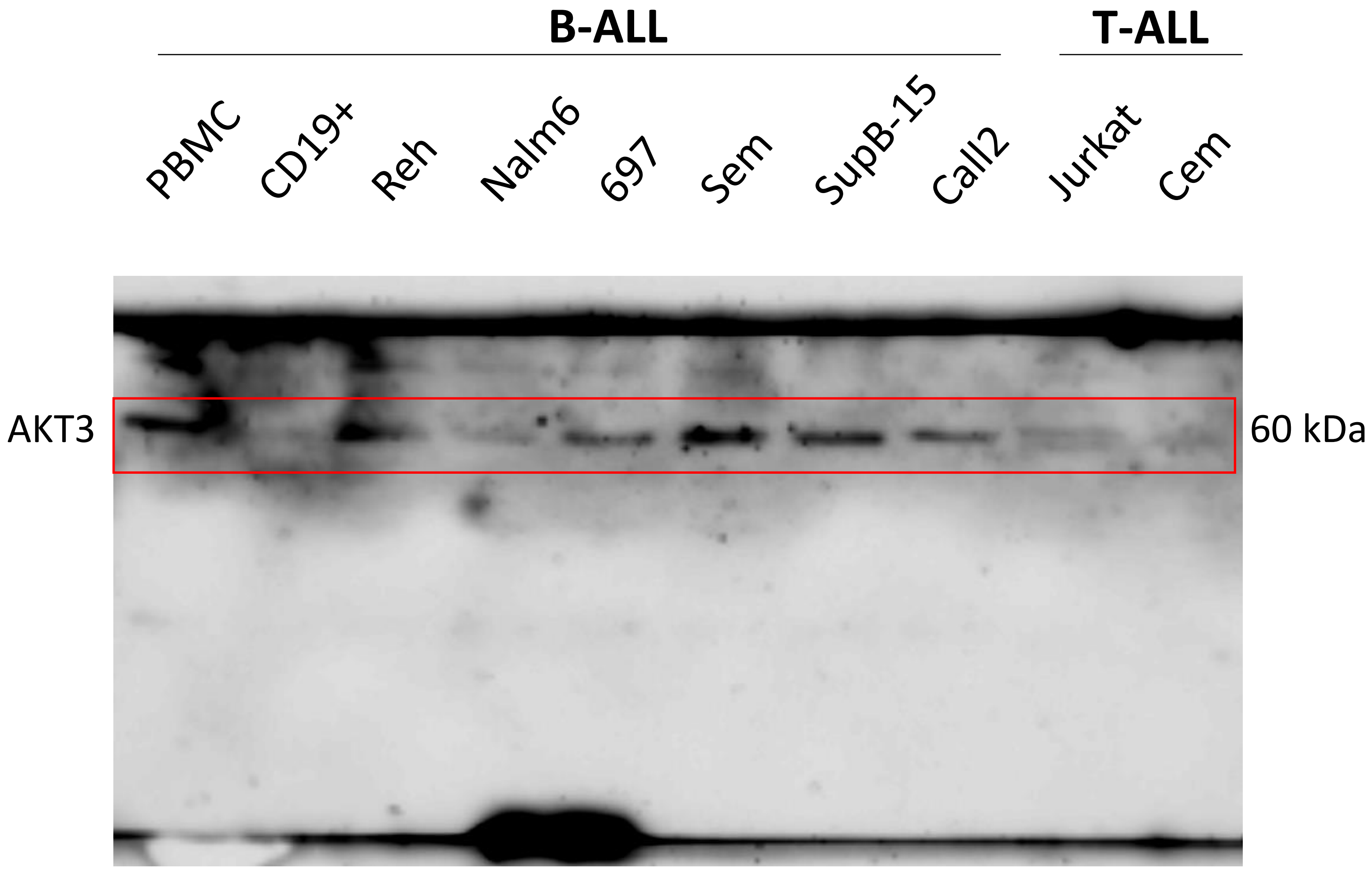
B-ALL
T-ALL
PBMC
CD19+
Reh
Nalm6
697
Sem
SupB-15
Call2
Jurkat
Cem
AKT3
60 kDa

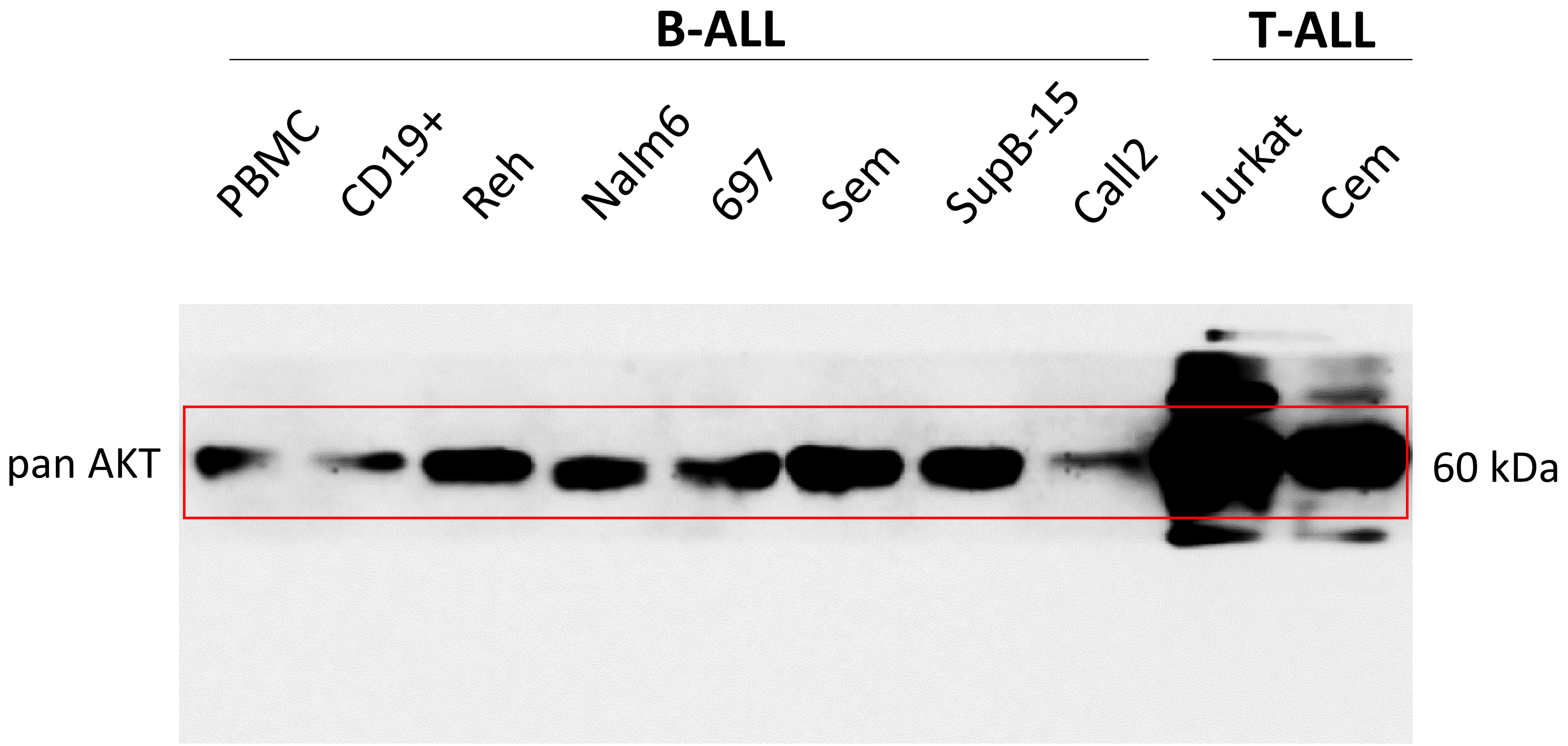
B-ALL
T-ALL
PBMC
CD19+
Reh
Nalm6
697
Sem
SupB-15
Call2
Jurkat
Cem
pan AKT
60 kDa

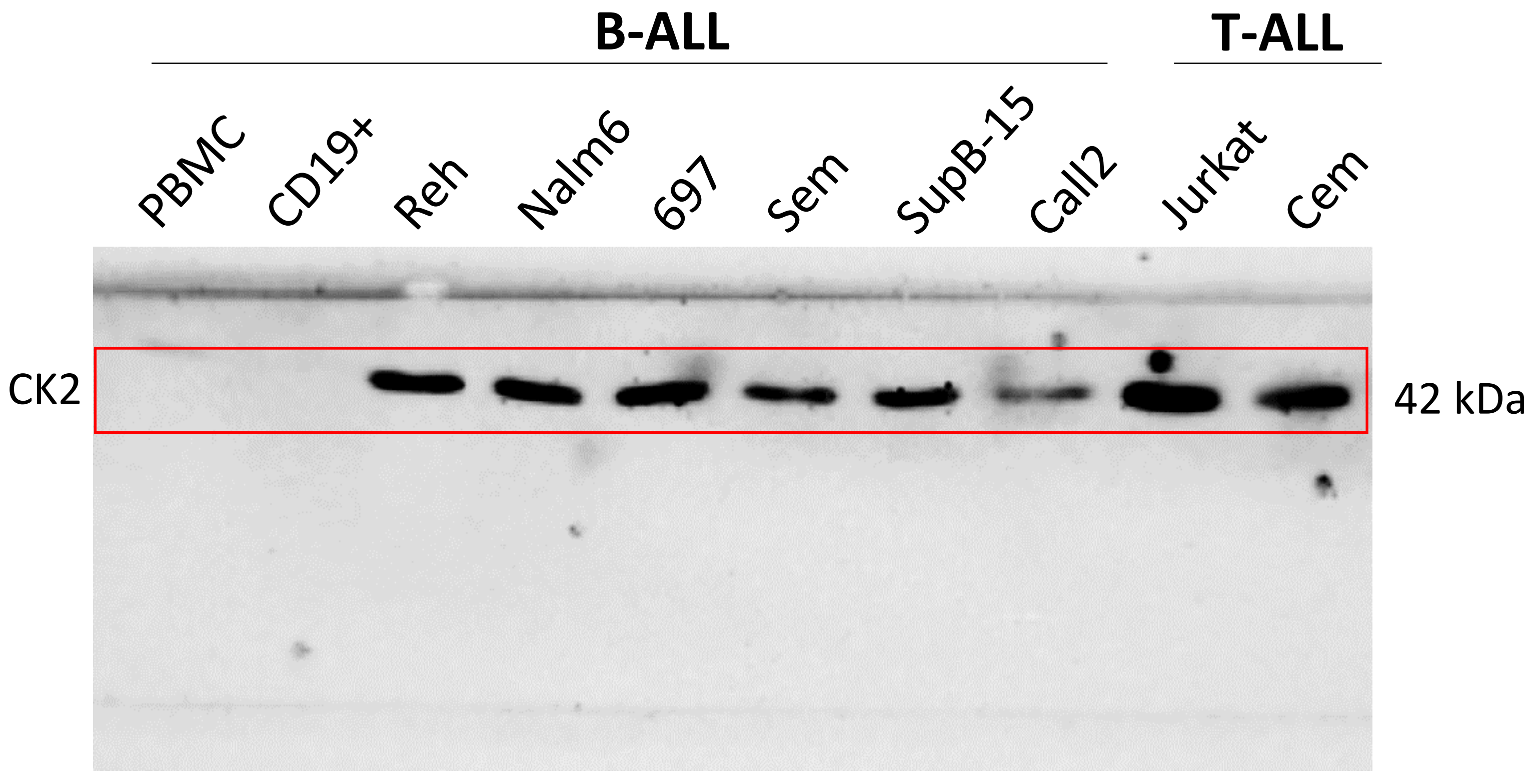
B-ALL
T-ALL
PBMC
CD19+
Reh
Nalm6
697
Sem
SupB-15
Call2
Jurkat
Cem
CK2
42 kDa

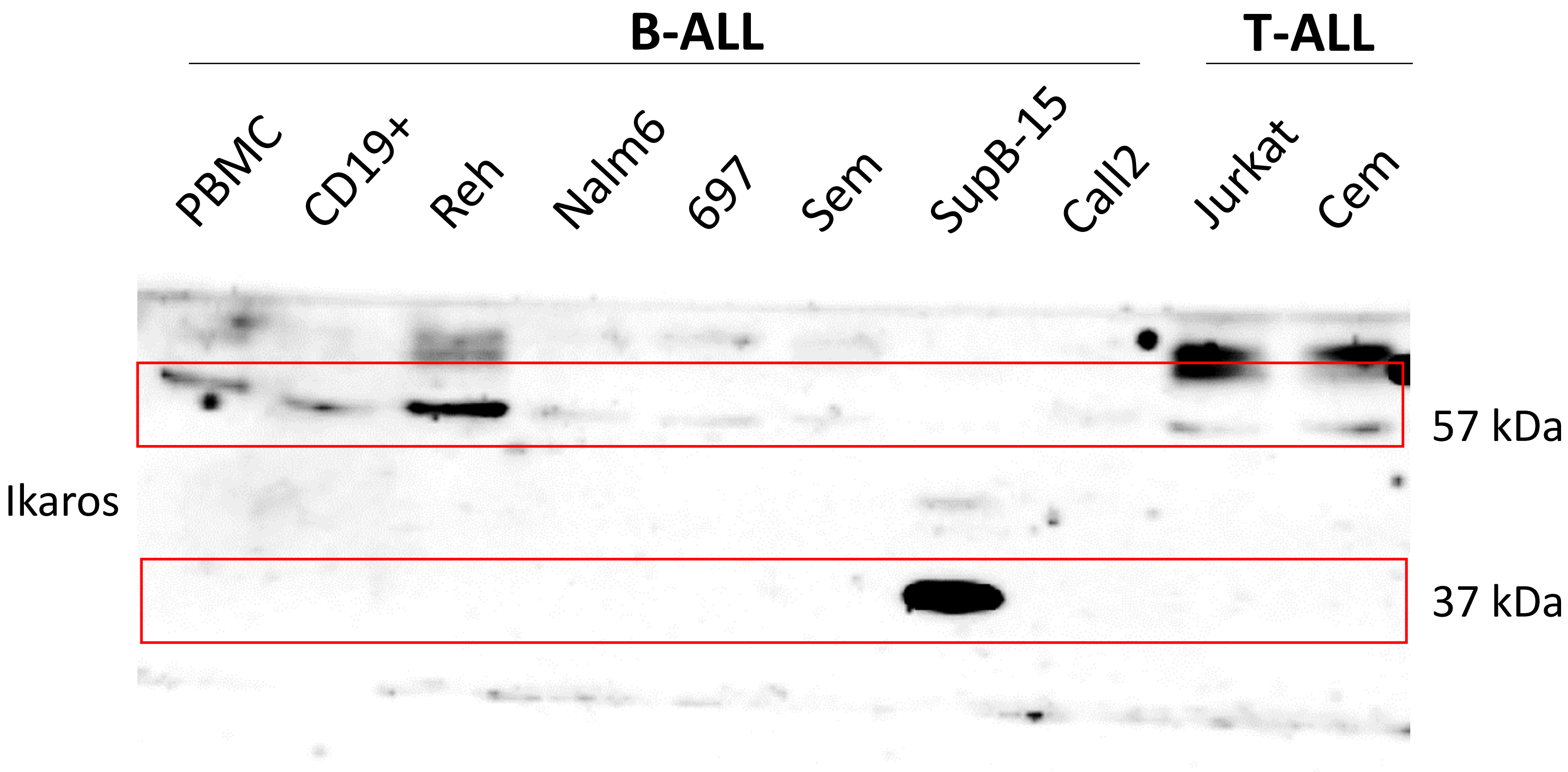
B-ALL
T-ALL
PBMC
CD19+
Reh
Nalm6
697
Sem
SupB-15
Call2
Jurkat
Cem
Ikaros
57 kDa
37 kDa

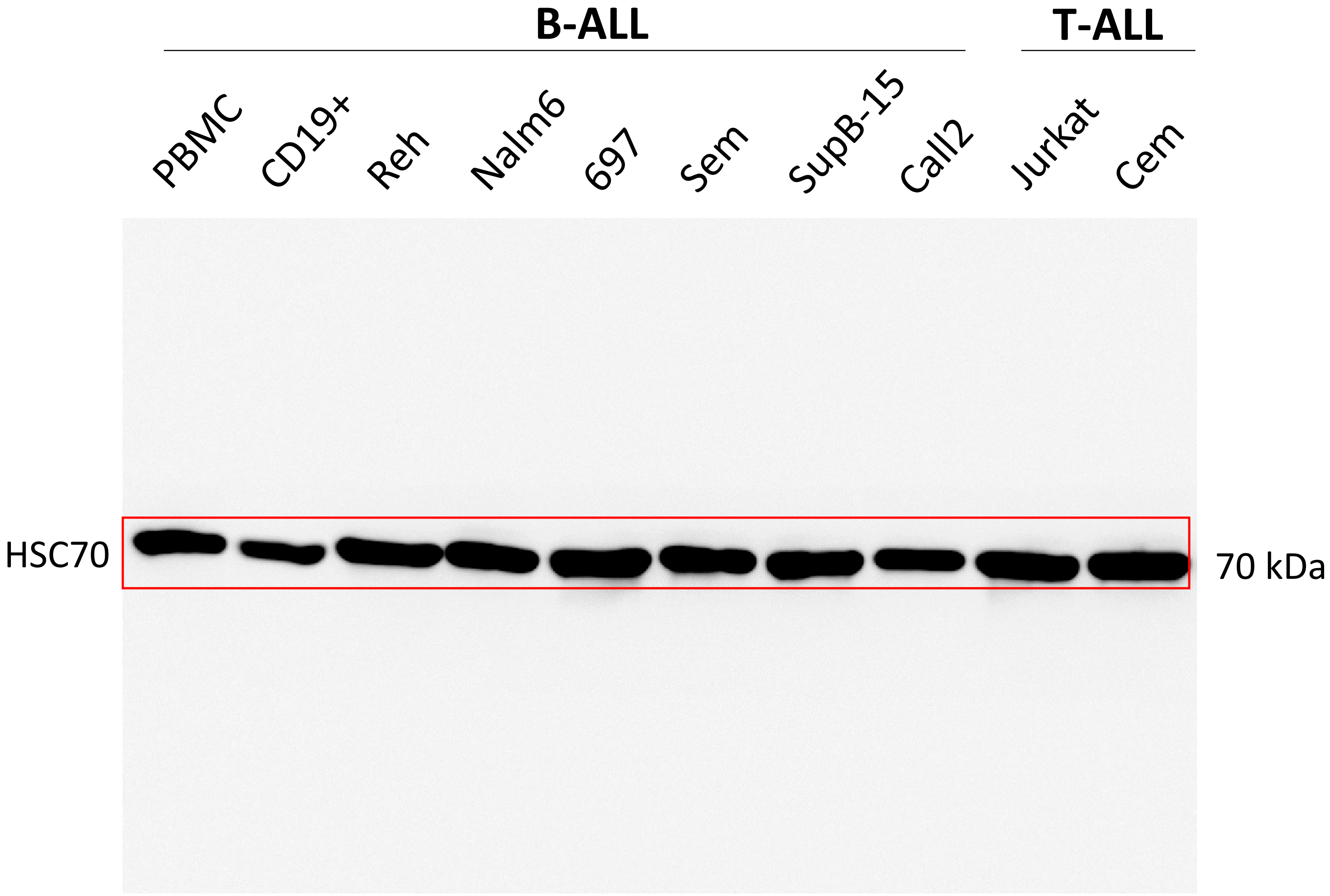

B-ALL
T-ALL
PBMC
CD19+
Reh
Nalm6
697
Sem
SupB-15
Call2
Jurkat
Cem
HSC70
70 kDa

Figure 2B

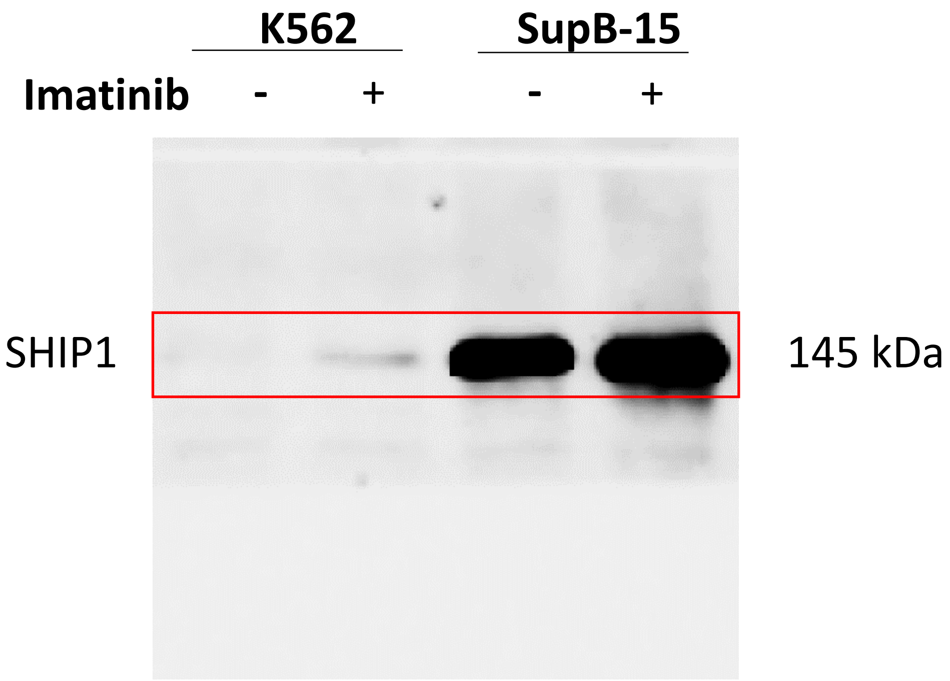
K562
SupB-15
Imatinib
-
+
-
+
SHIP1
145 kDa

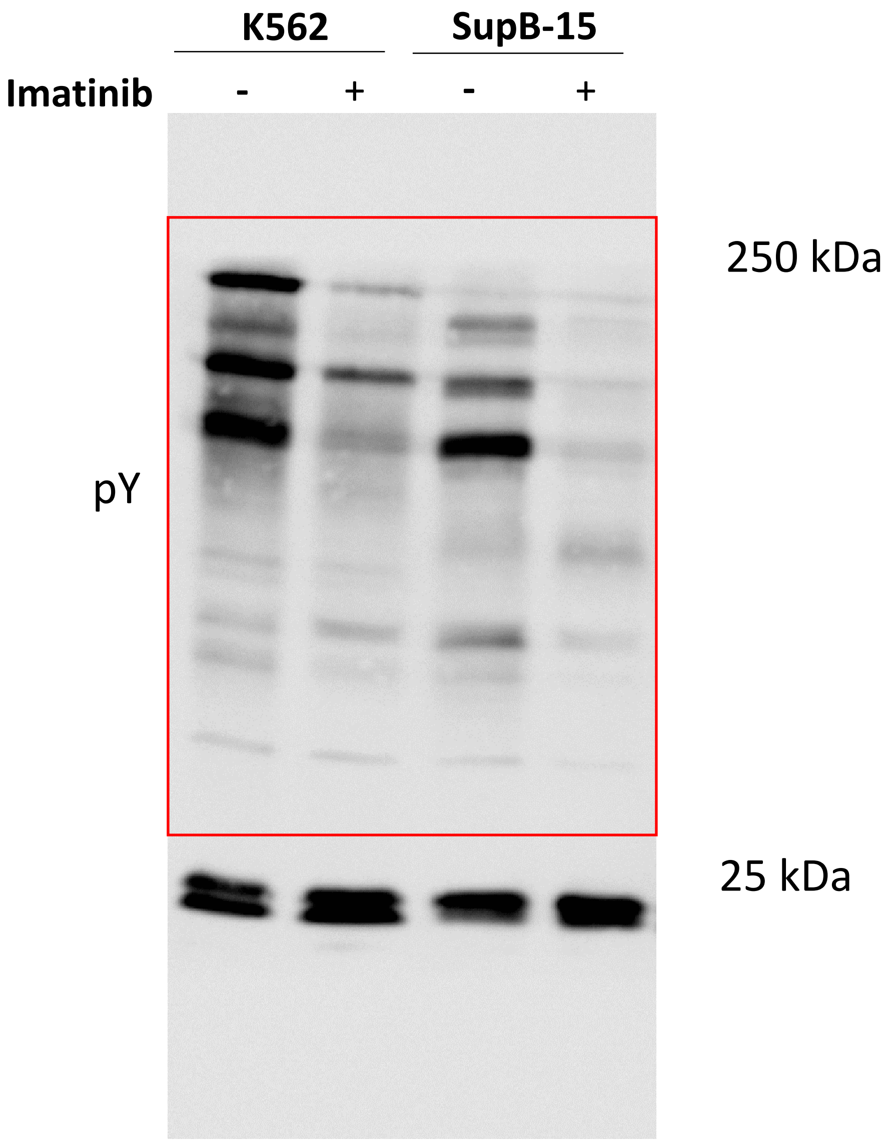

K562
SupB-15
Imatinib
-
+
-
+
pY
250 kDa
25 kDa

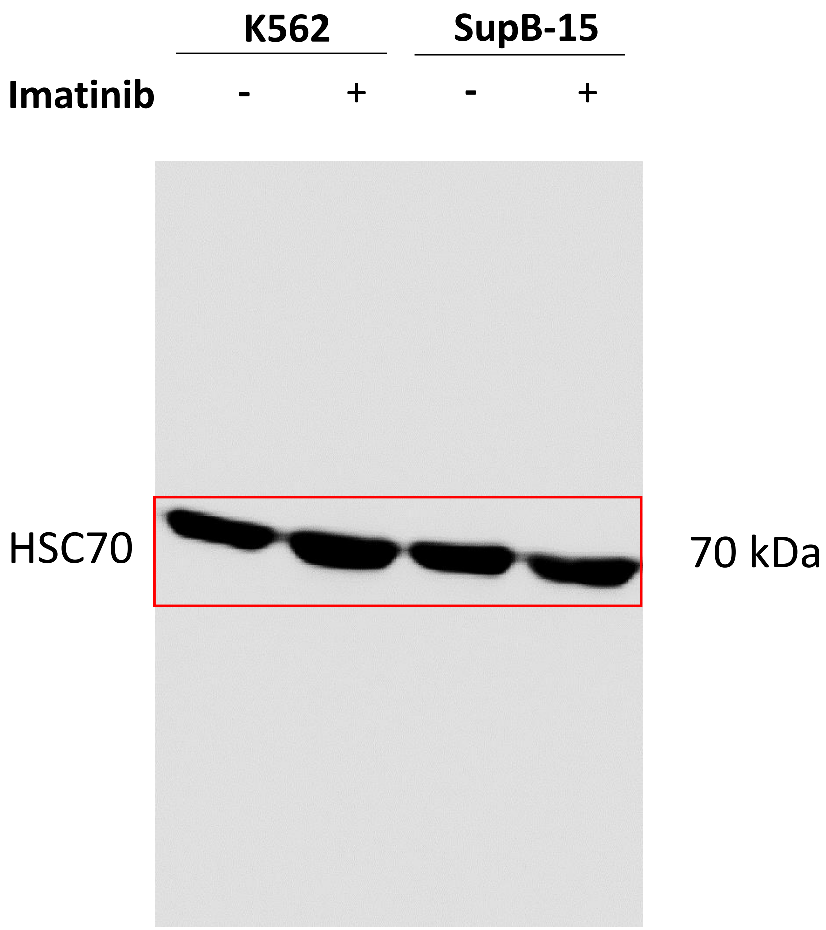

K562
SupB-15
Imatinib
-
+
-
+
HSC70
70 kDa

Figure 2D

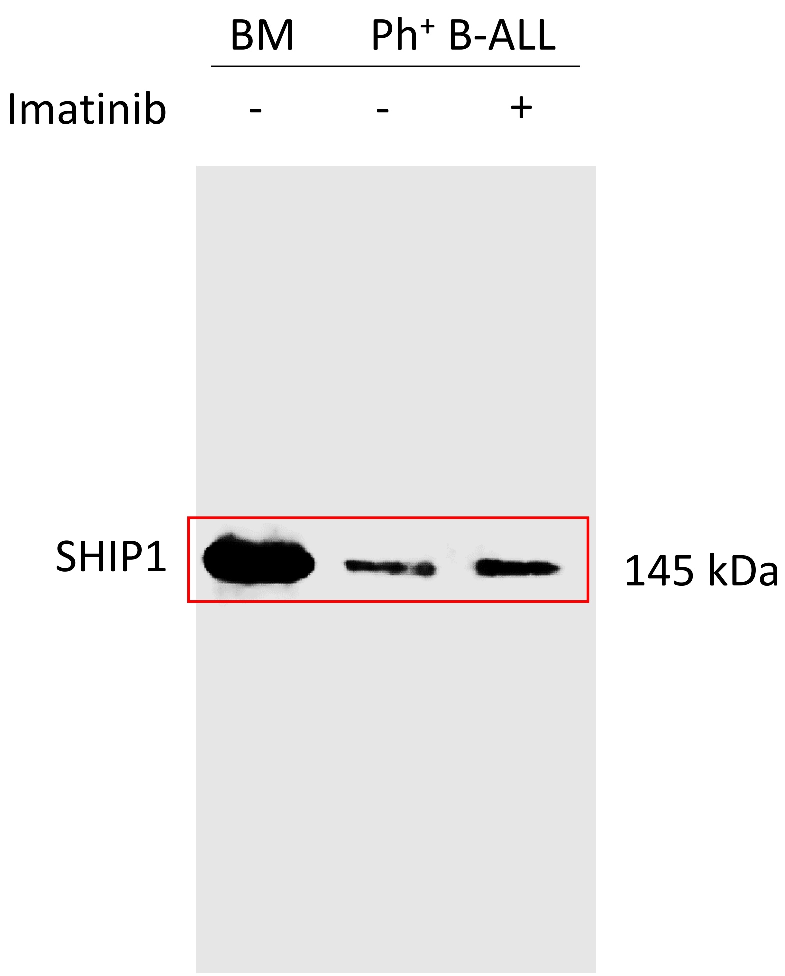

BM
Ph+ B-ALL
Imatinib
-
-
+
SHIP1
145 kDa

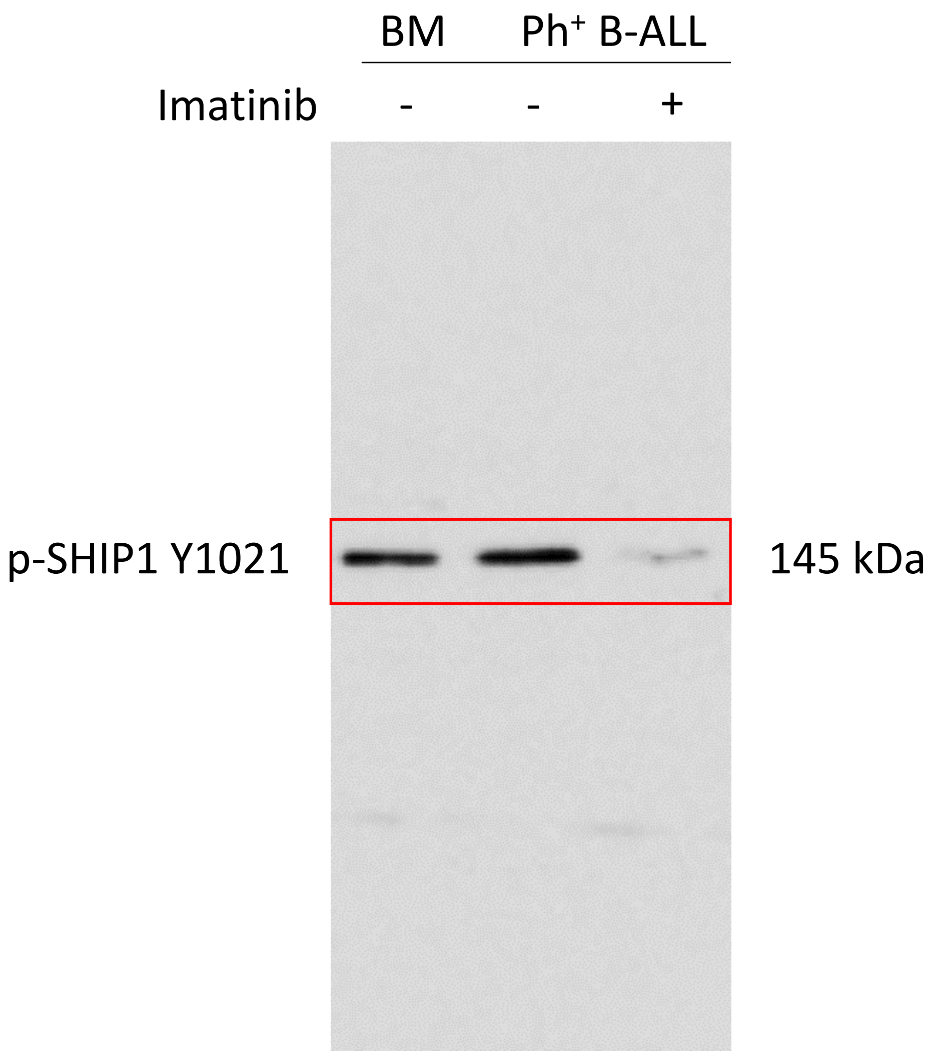
BM
Ph+ B-ALL
Imatinib
-
-
+
p-SHIP1 Y1021
145 kDa

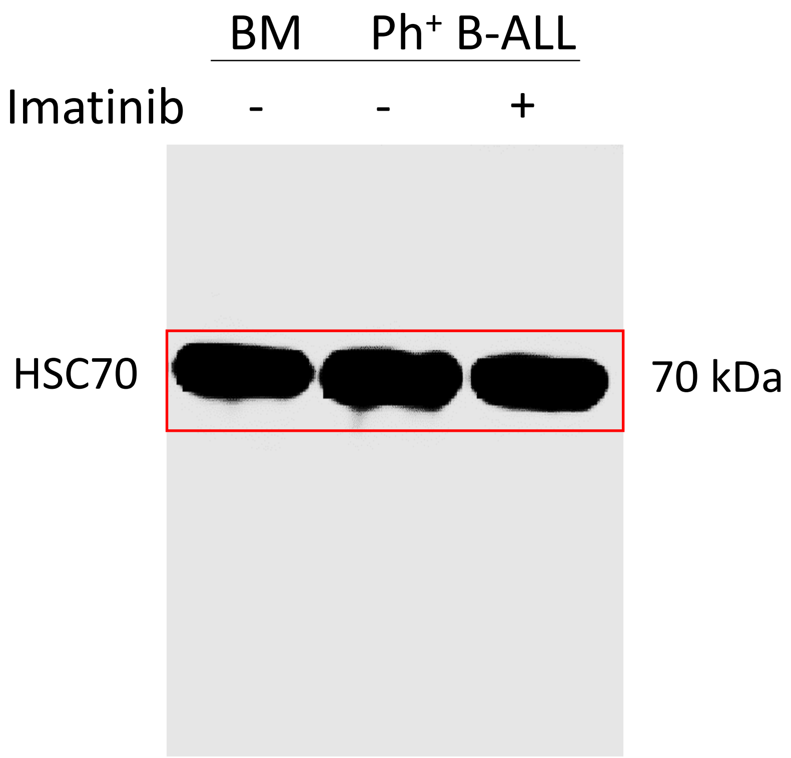
BM
Ph+ B-ALL
Imatinib
-
-
+
HSC70
70 kDa

Figure 3A

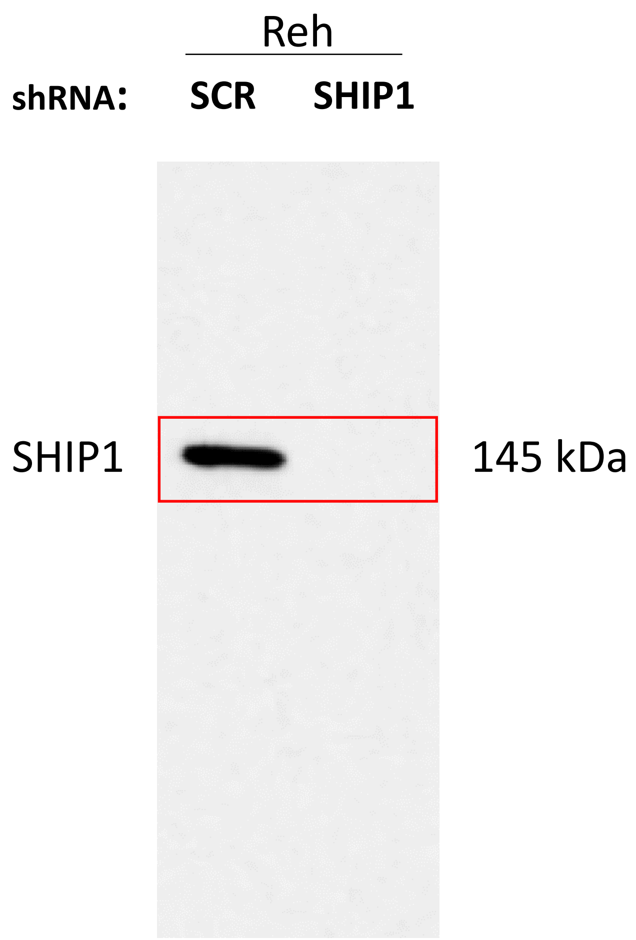
Reh
shRNA:
SCR
SHIP1
SHIP1
145 kDa

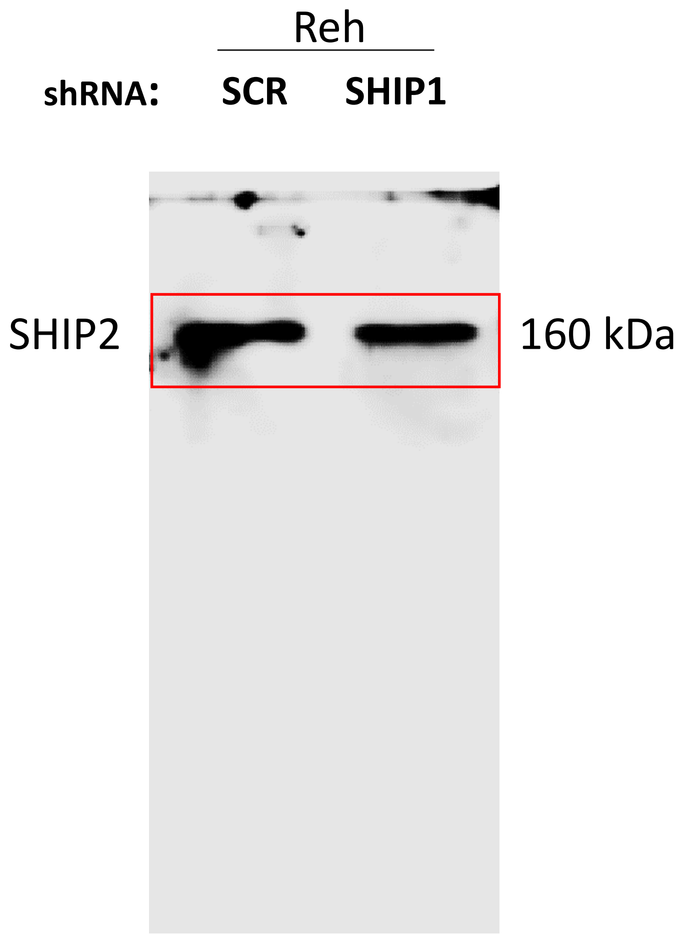
Reh
shRNA:
SCR
SHIP1
SHIP2
160 kDa

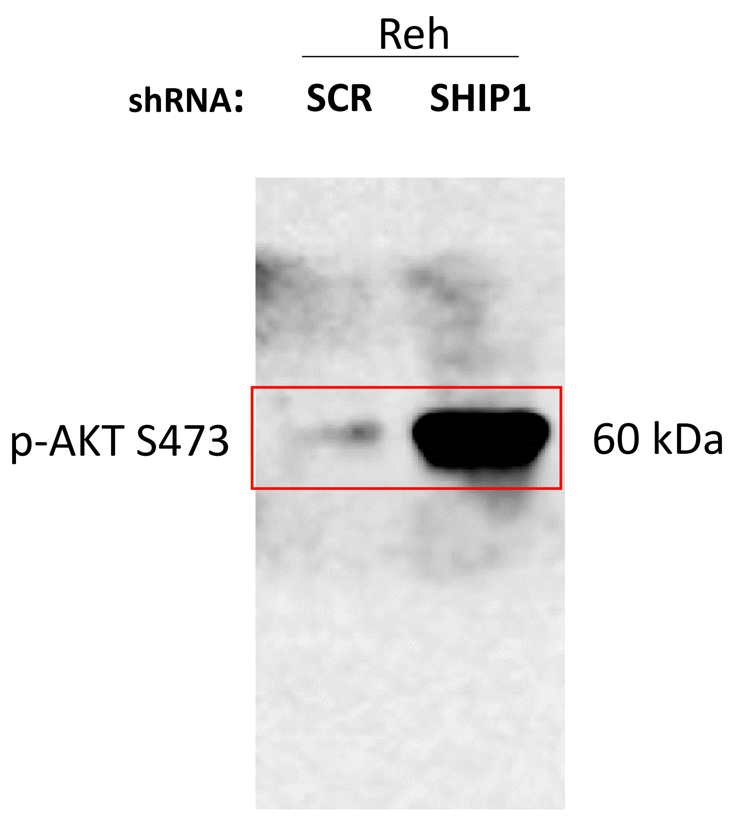
Reh
shRNA:
SCR
SHIP1
p-AKT S473
60 kDa

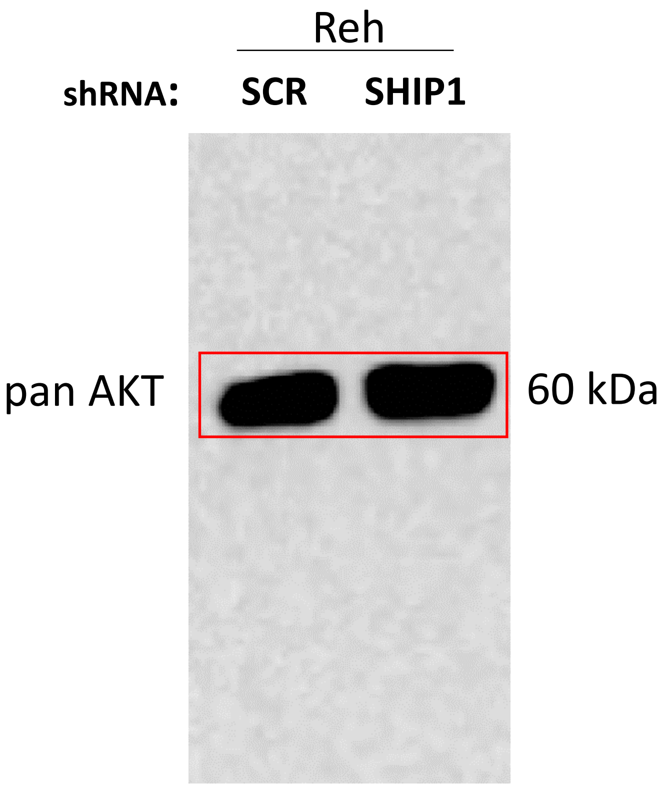
Reh
shRNA:
SCR
SHIP1
pan AKT
60 kDa

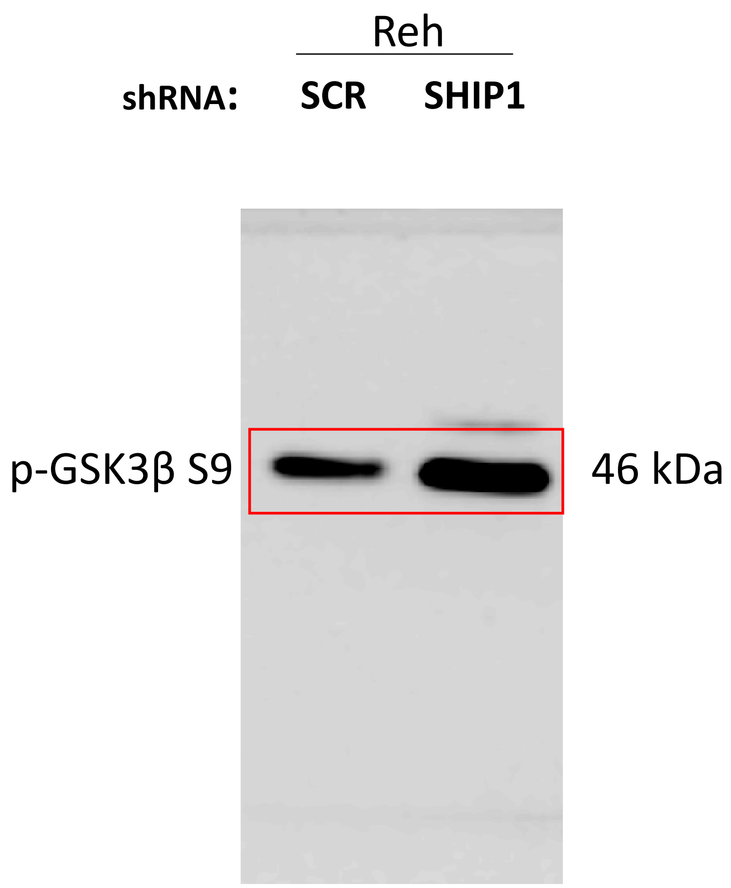
Reh
shRNA:
SCR
SHIP1
p-GSK3β S9
46 kDa

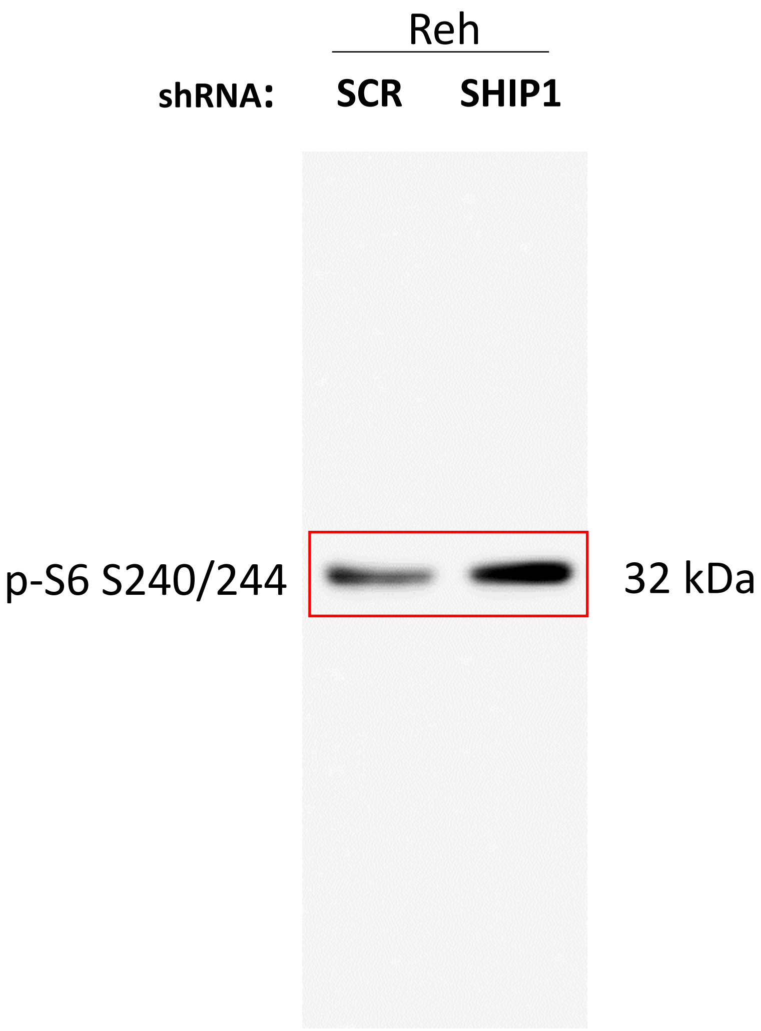
Reh
shRNA:
SCR
SHIP1
p-S6 S240/244
32 kDa

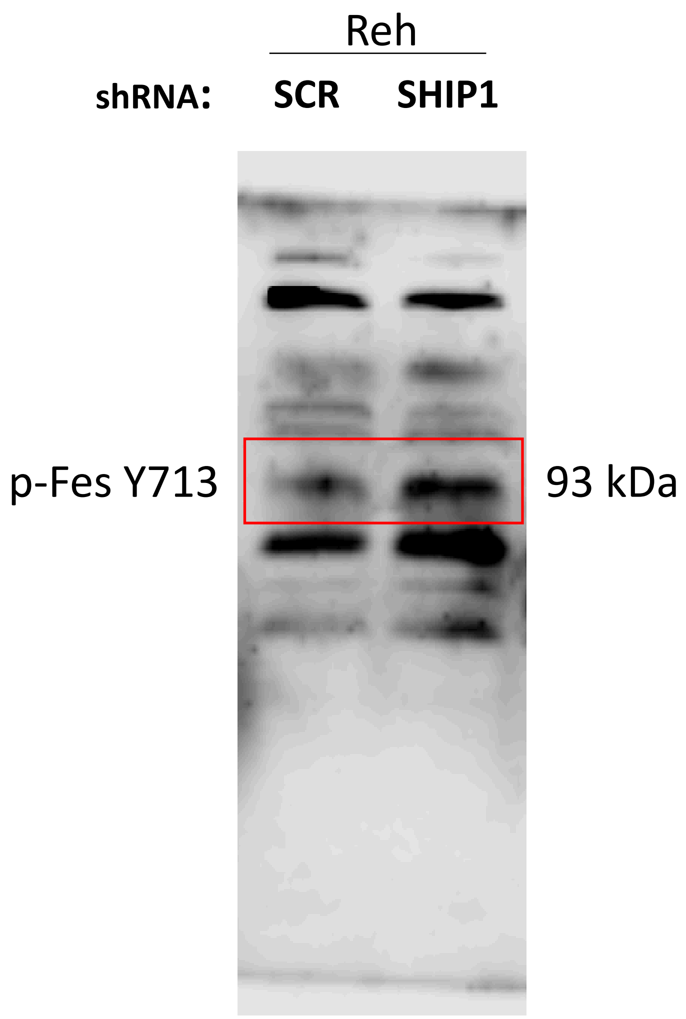
Reh
shRNA:
SCR
SHIP1
p-Fes Y713
93 kDa

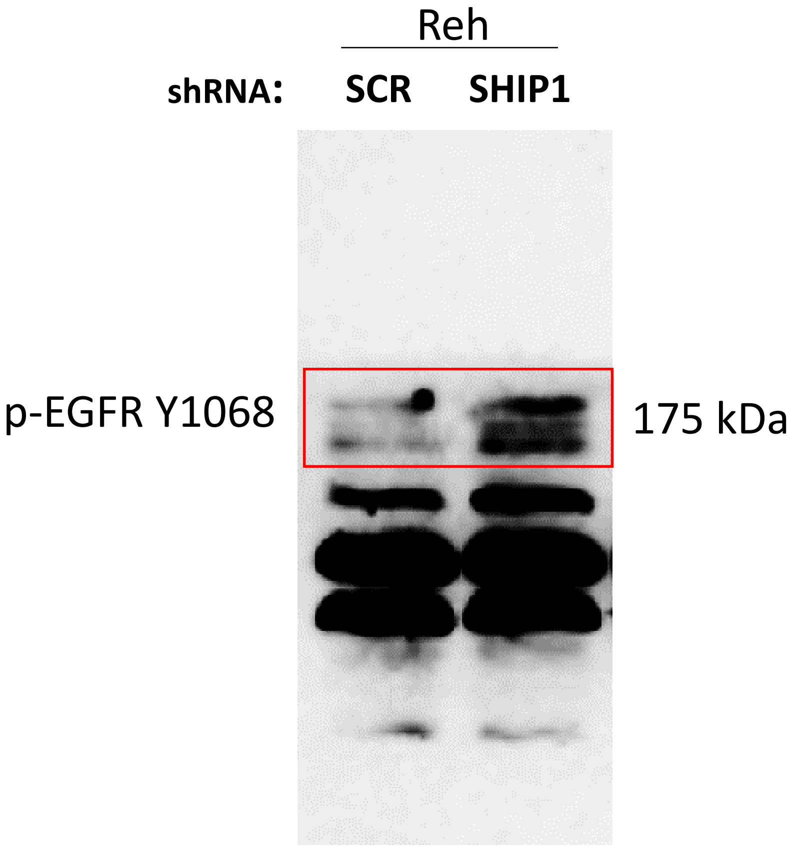
Reh
shRNA:
SCR
SHIP1
p-EGFR Y1068
175 kDa

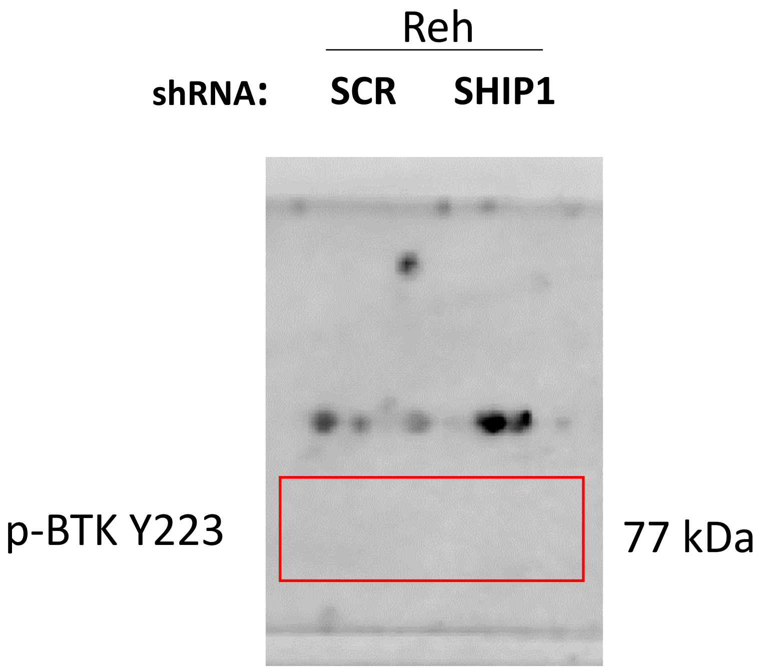
Reh
shRNA:
SCR
SHIP1
p-BTK Y223
77 kDa

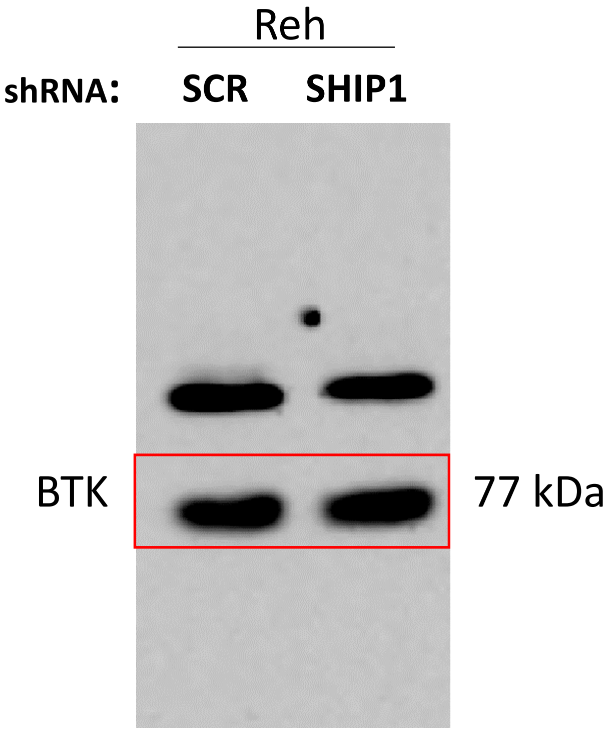
Reh
shRNA:
SCR
SHIP1
BTK
77 kDa

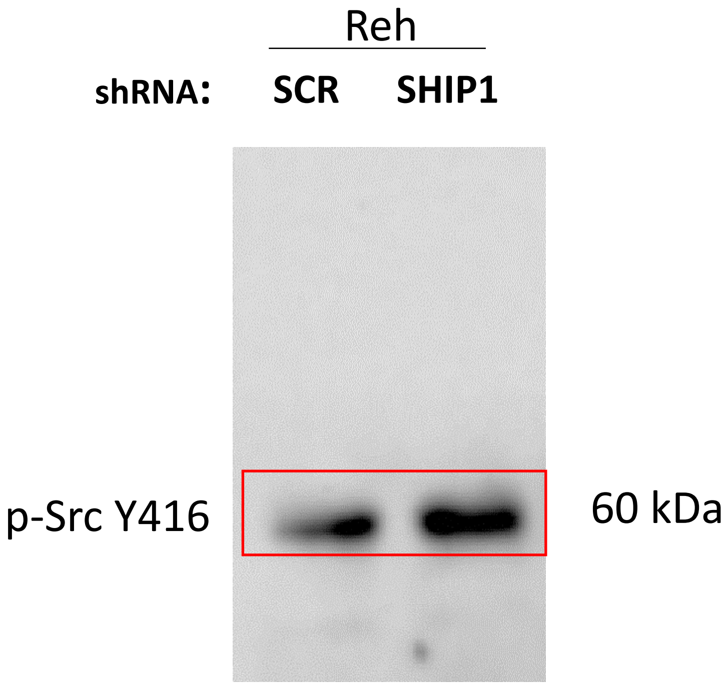

Reh
shRNA:
SCR
SHIP1
p-Src Y416
60 kDa

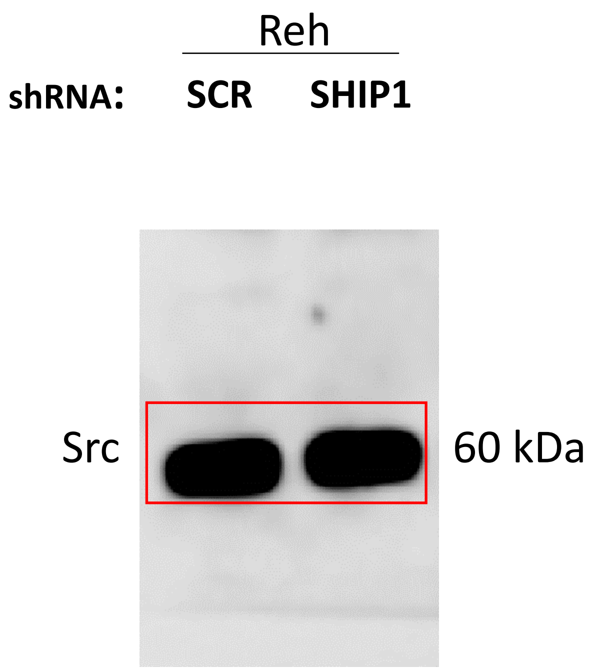

Reh
shRNA:
SCR
SHIP1
Src
60 kDa

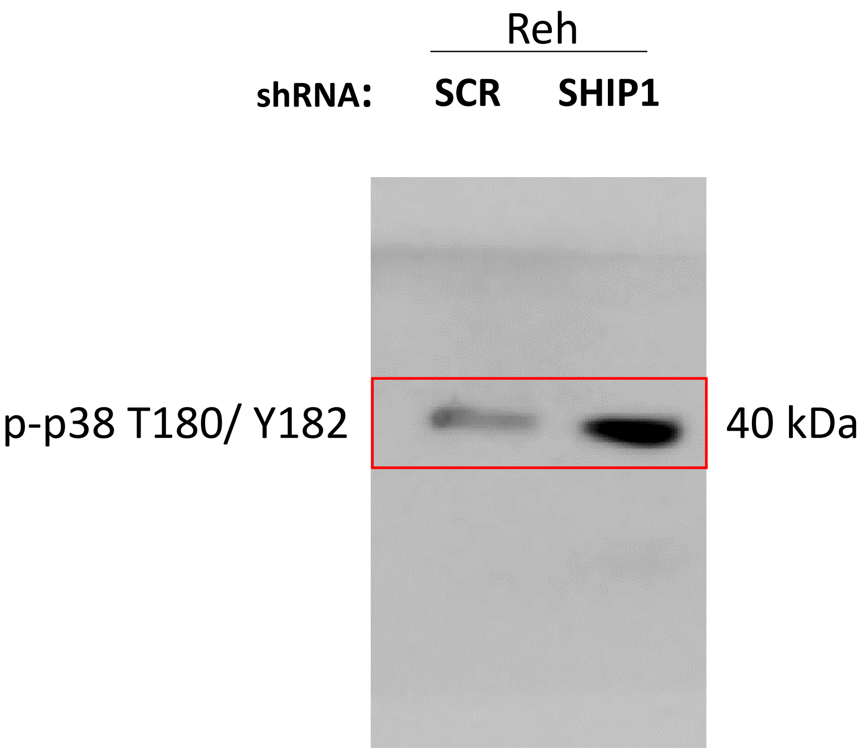
Reh
shRNA:
SCR
SHIP1
p-p38 T180/ Y182
40 kDa

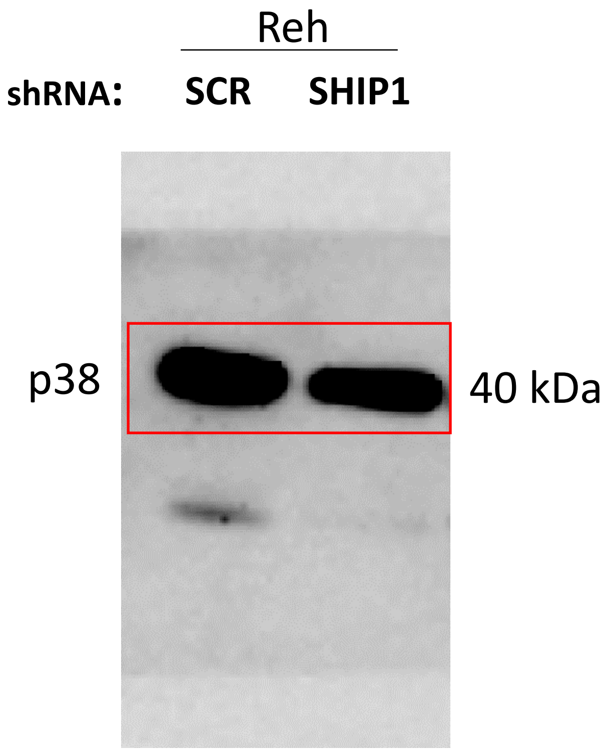
Reh
shRNA:
SCR
SHIP1
p38
40 kDa

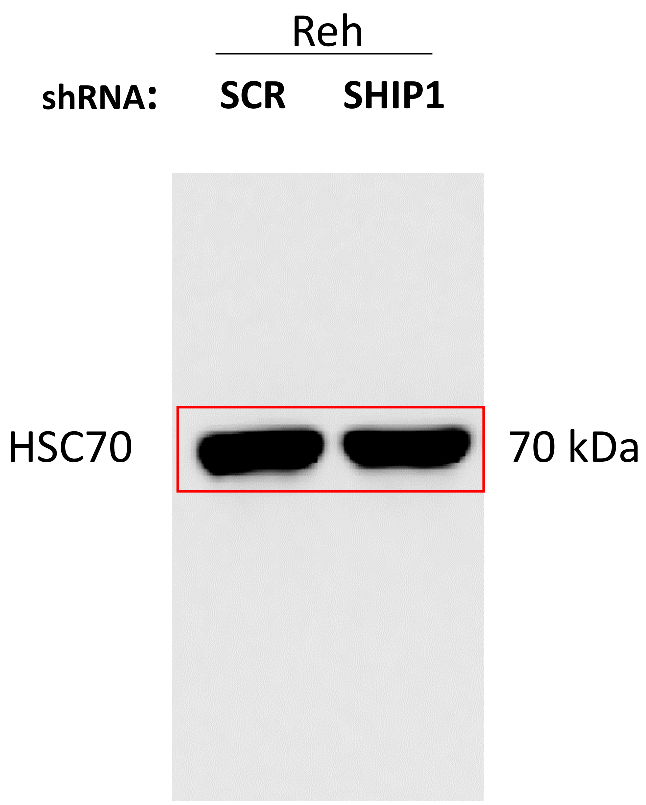
Reh
shRNA:
SCR
SHIP1
HSC70
70 kDa

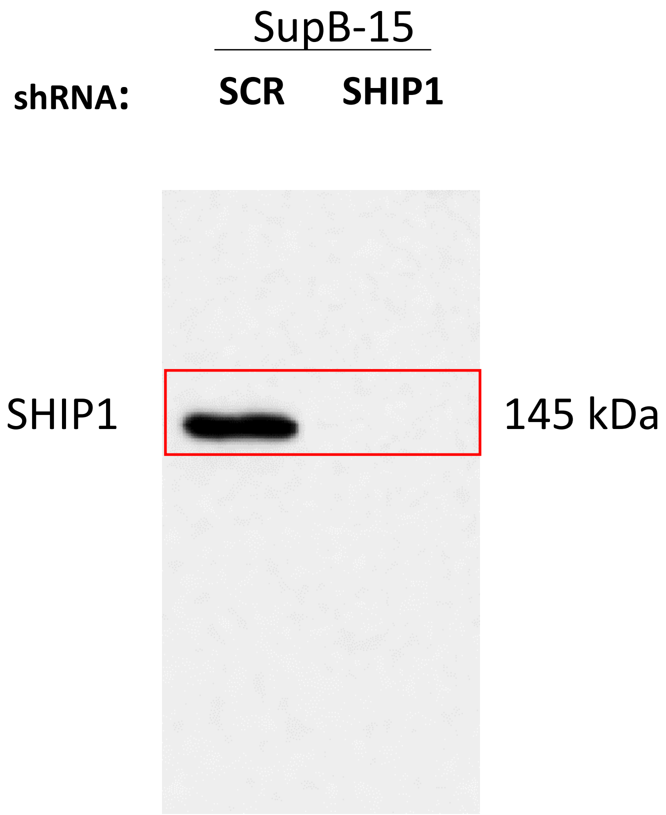
SupB-15
shRNA:
SCR
SHIP1
SHIP1
145 kDa

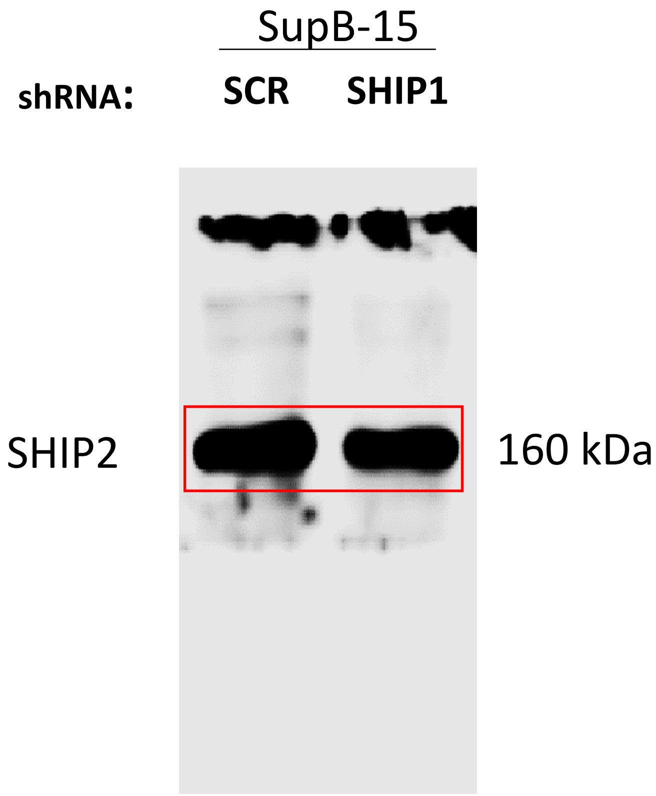

SupB-15
shRNA:
SCR
SHIP1
SHIP2
160 kDa

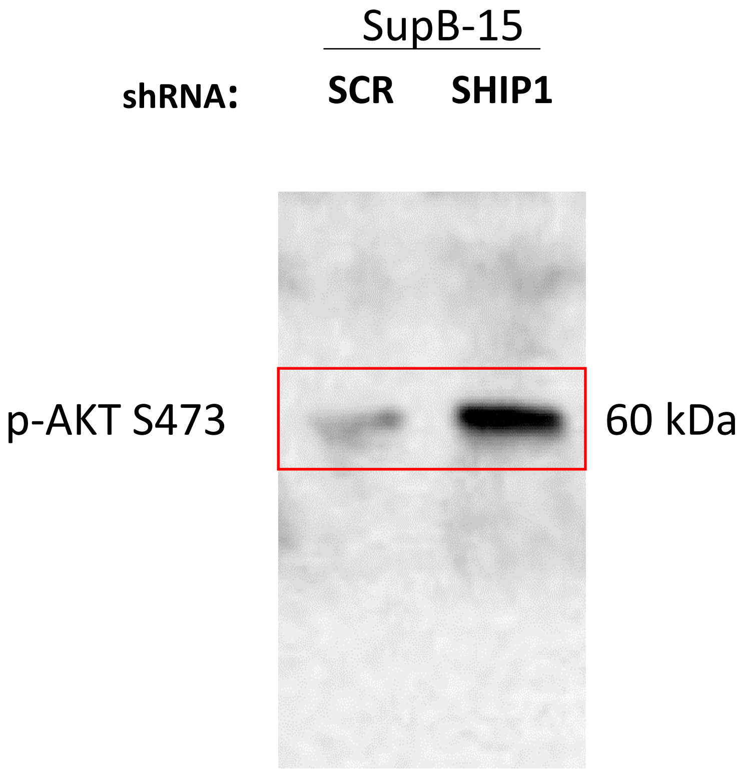
SupB-15
shRNA:
SCR
SHIP1
p-AKT S473
60 kDa

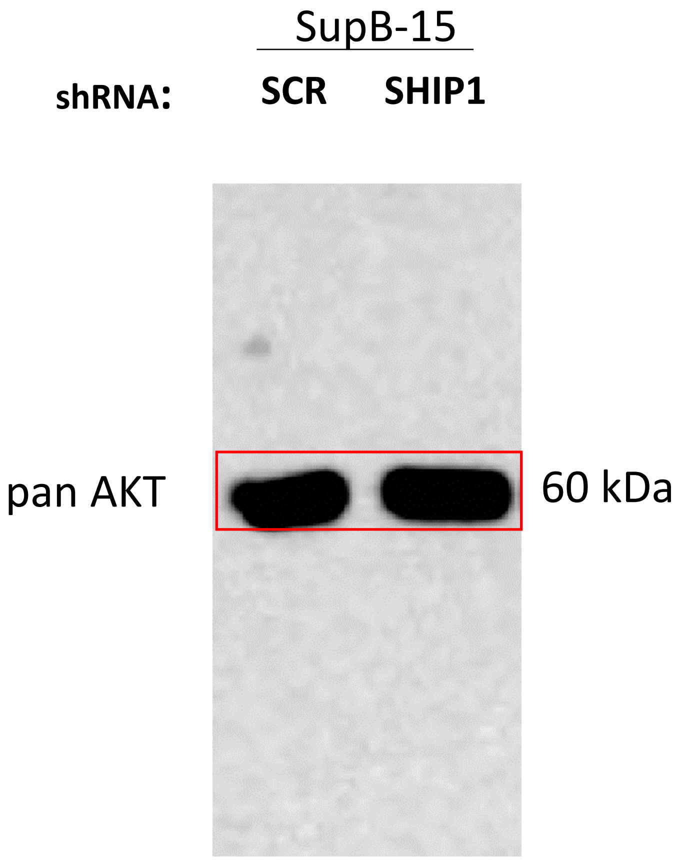
SupB-15
shRNA:
SCR
SHIP1
pan AKT
60 kDa

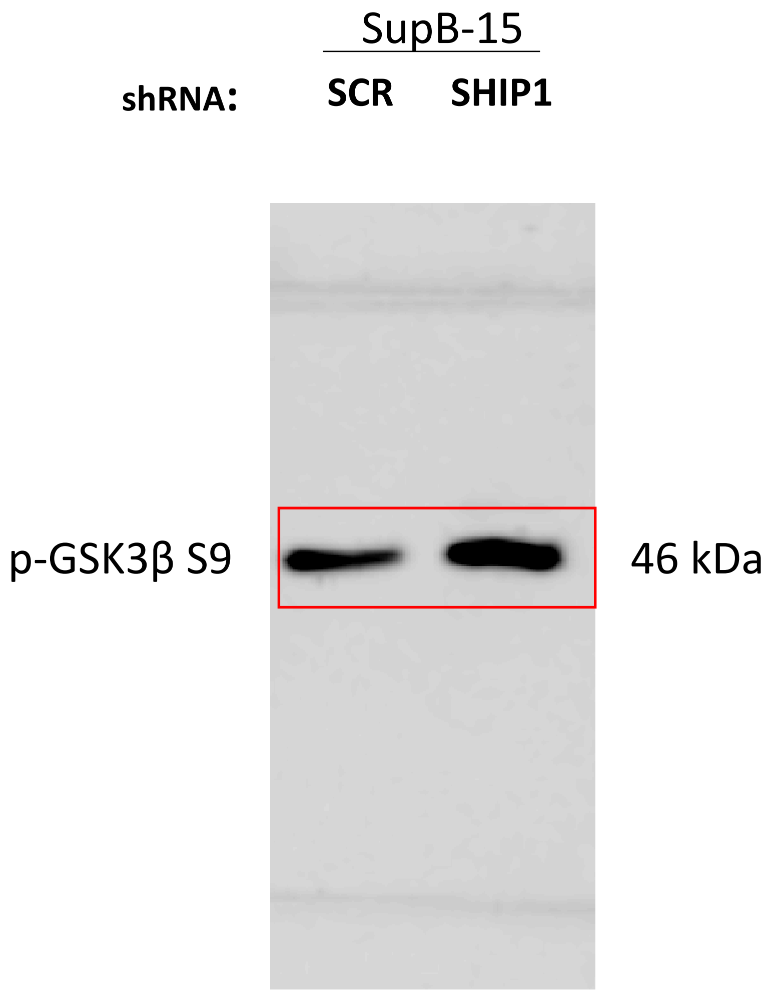
SupB-15
shRNA:
SCR
SHIP1
p-GSK3β S9
46 kDa

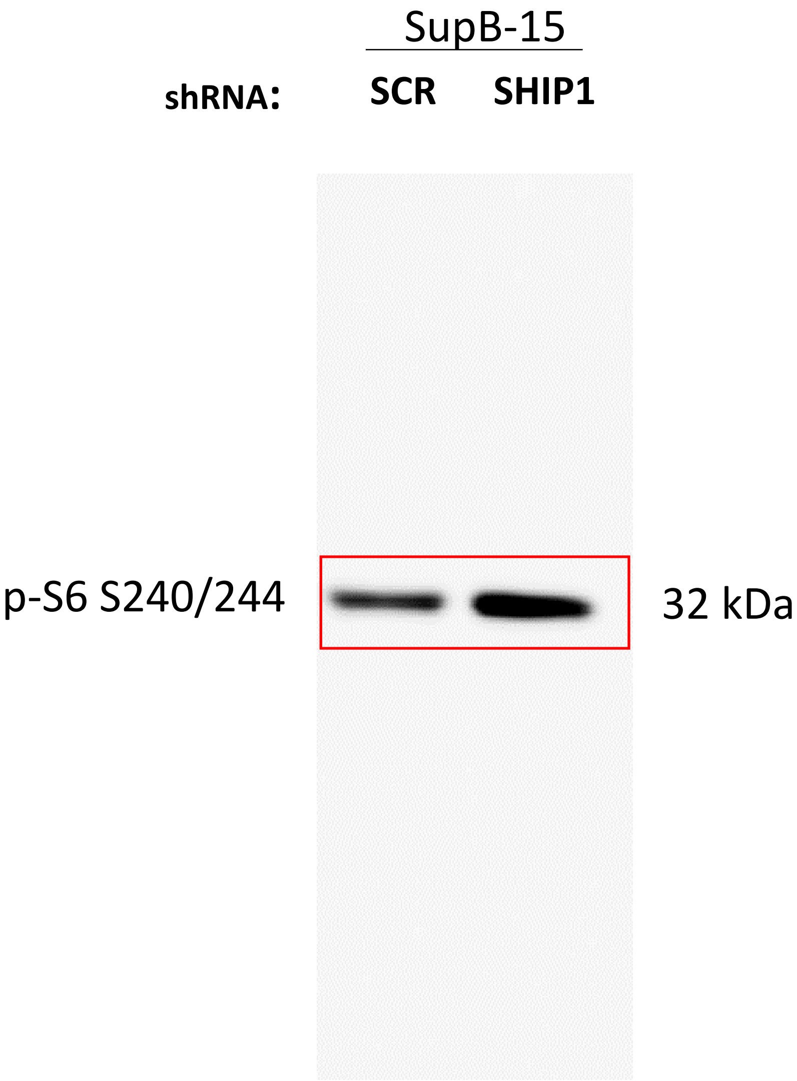
SupB-15
shRNA:
SCR
SHIP1
p-S6 S240/244
32 kDa

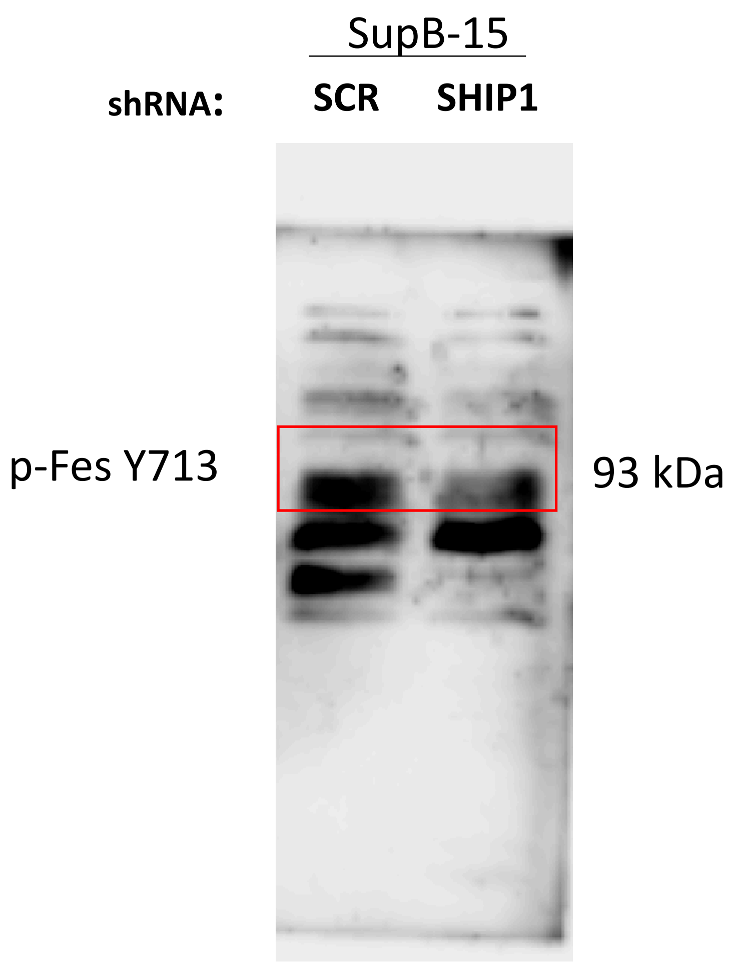
SupB-15
shRNA:
SCR
SHIP1
p-Fes Y713
93 kDa

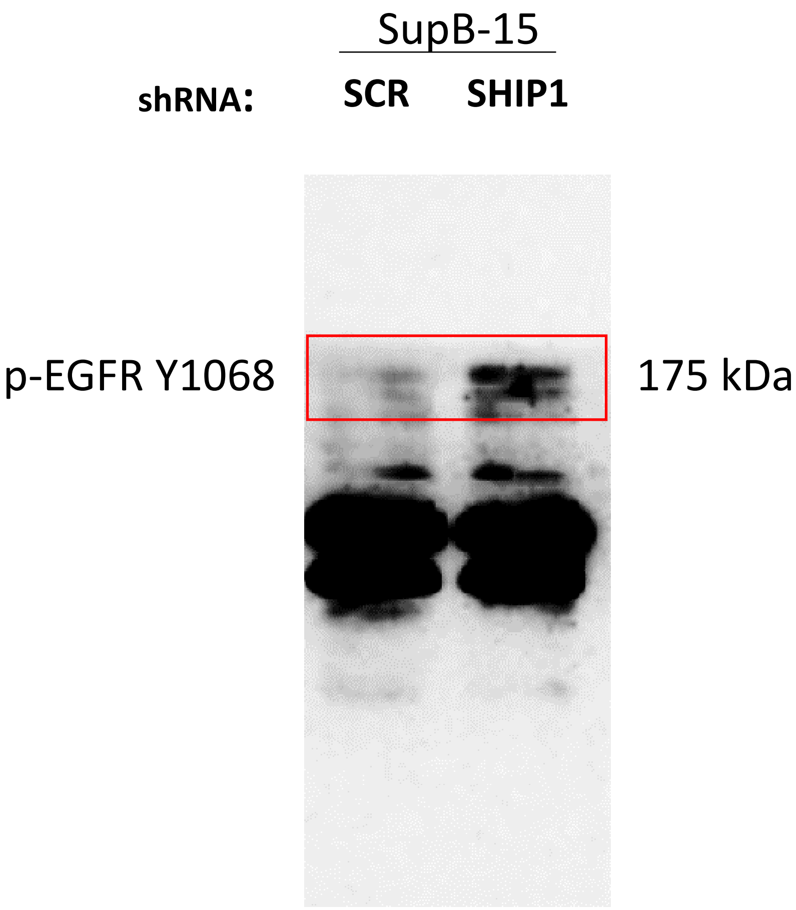
SupB-15
shRNA:
SCR
SHIP1
p-EGFR Y1068
175 kDa

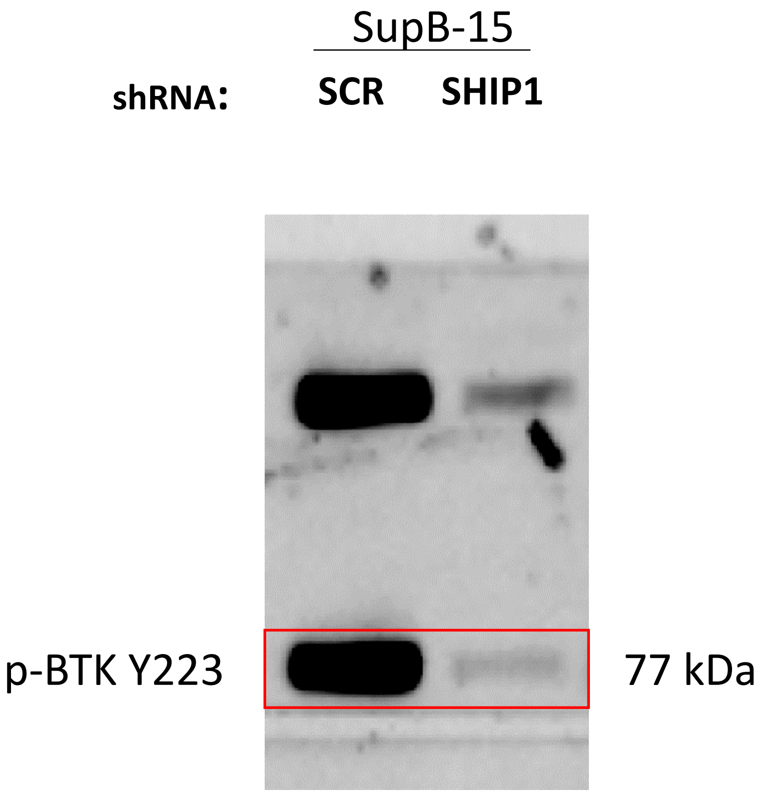
SupB-15
shRNA:
SCR
SHIP1
p-BTK Y223
77 kDa

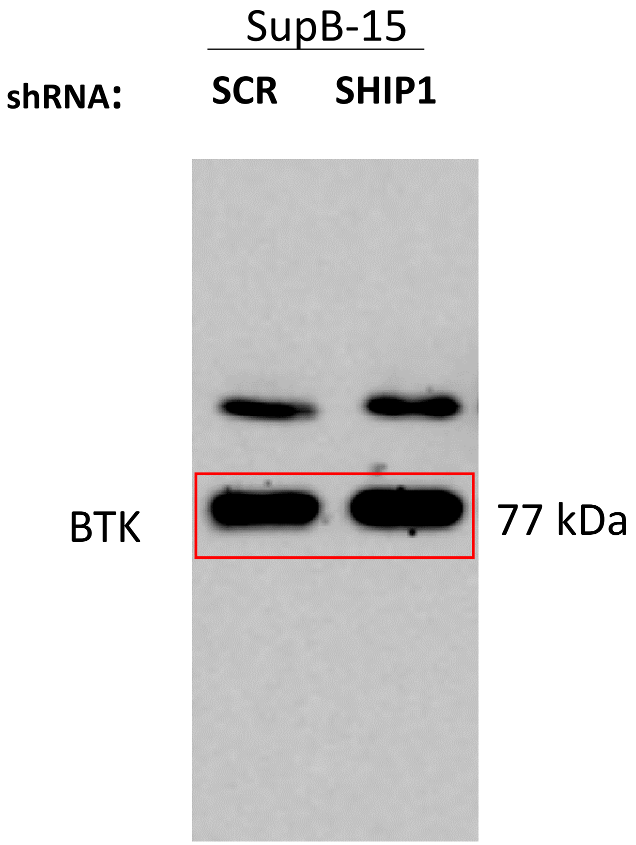
SupB-15
shRNA:
SCR
SHIP1
BTK
77 kDa

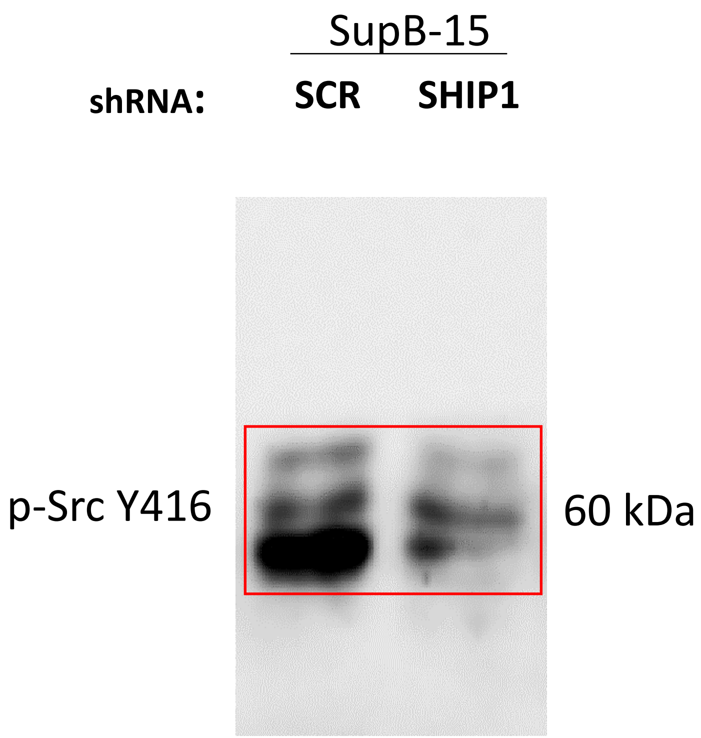
SupB-15
shRNA:
SCR
SHIP1
p-Src Y416
60 kDa

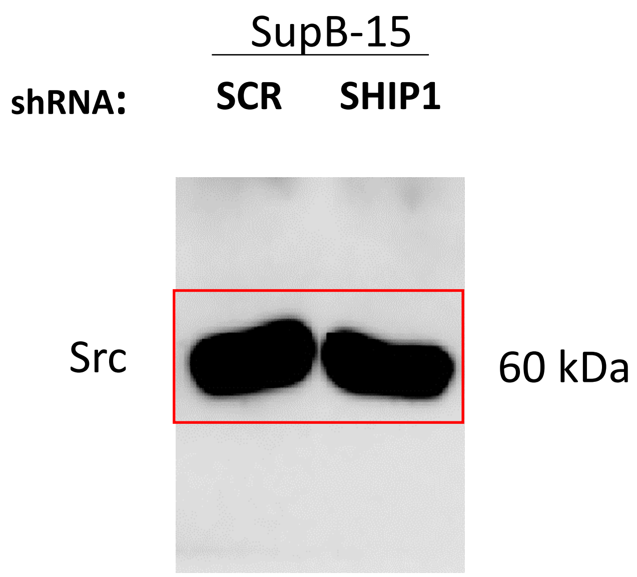
SupB-15
shRNA:
SCR
SHIP1
Src
60 kDa

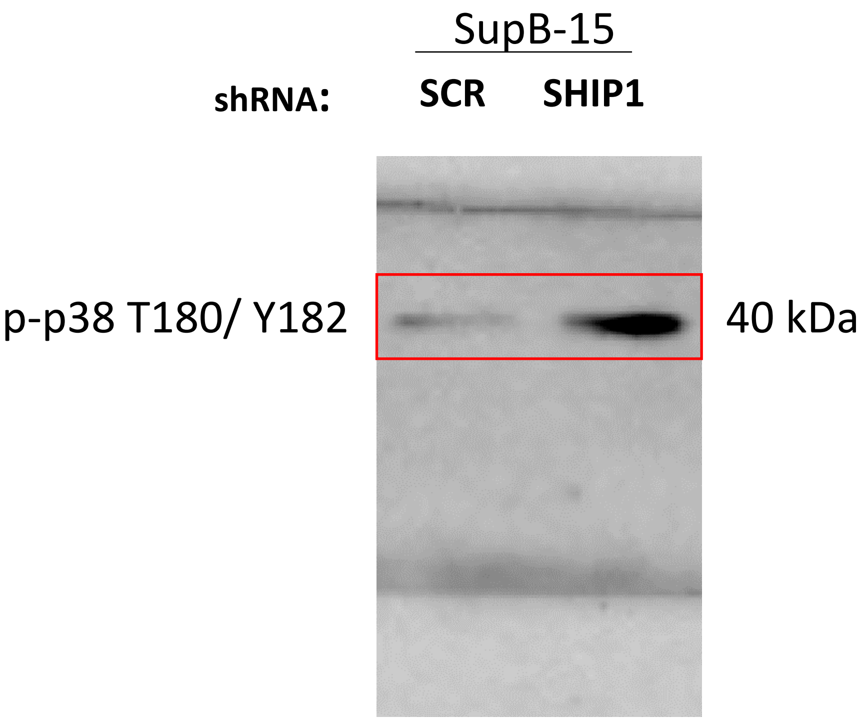
SupB-15
shRNA:
SCR
SHIP1
p-p38 T180/ Y182
40 kDa

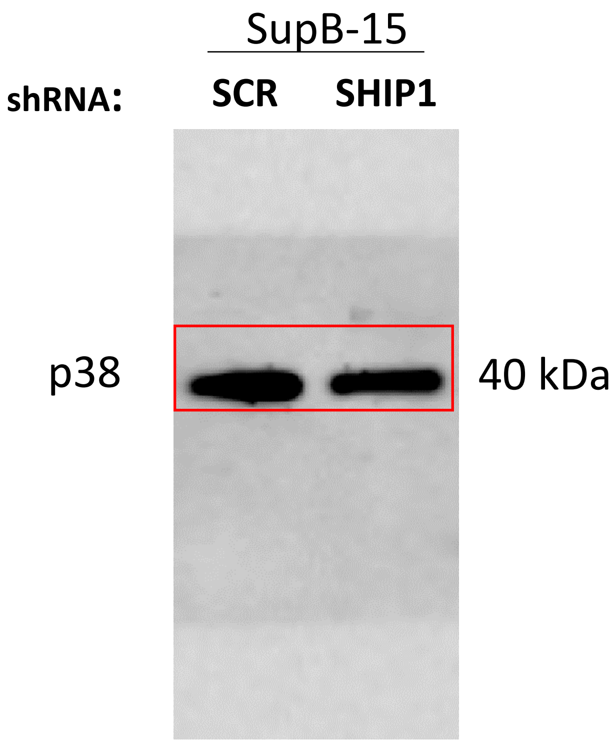
SupB-15
shRNA:
SCR
SHIP1
p38
40 kDa

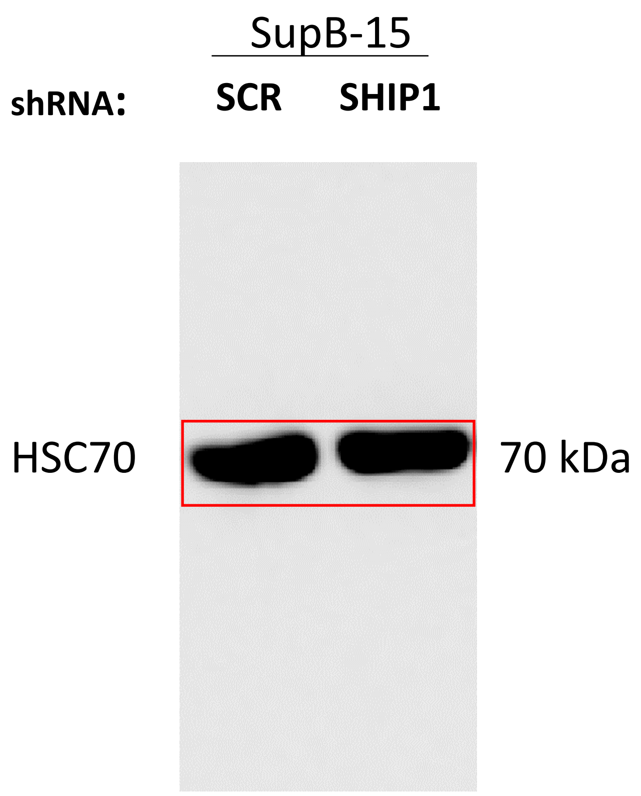

SupB-15
shRNA:
SCR
SHIP1
HSC70
70 kDa

Figure 5B

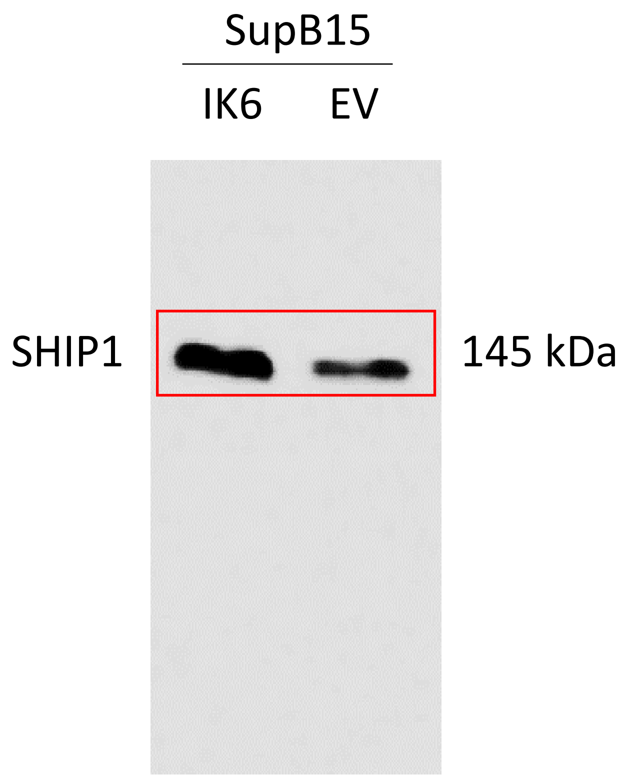
SupB15
IK6
EV
SHIP1
145 kDa

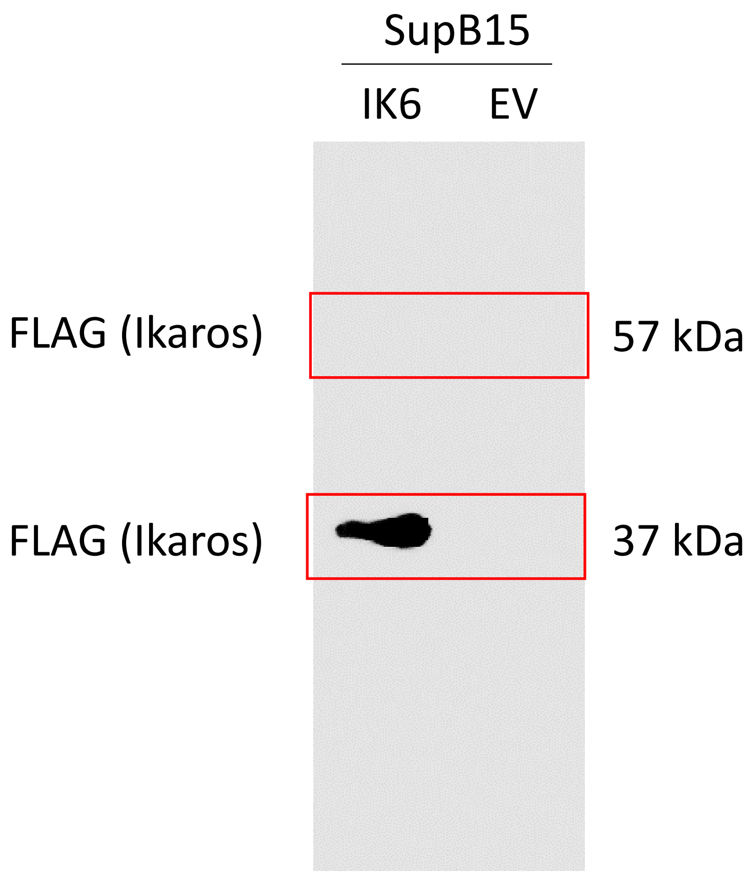
SupB15
IK6
EV
FLAG (Ikaros)
57 kDa
FLAG (Ikaros)
37 kDa

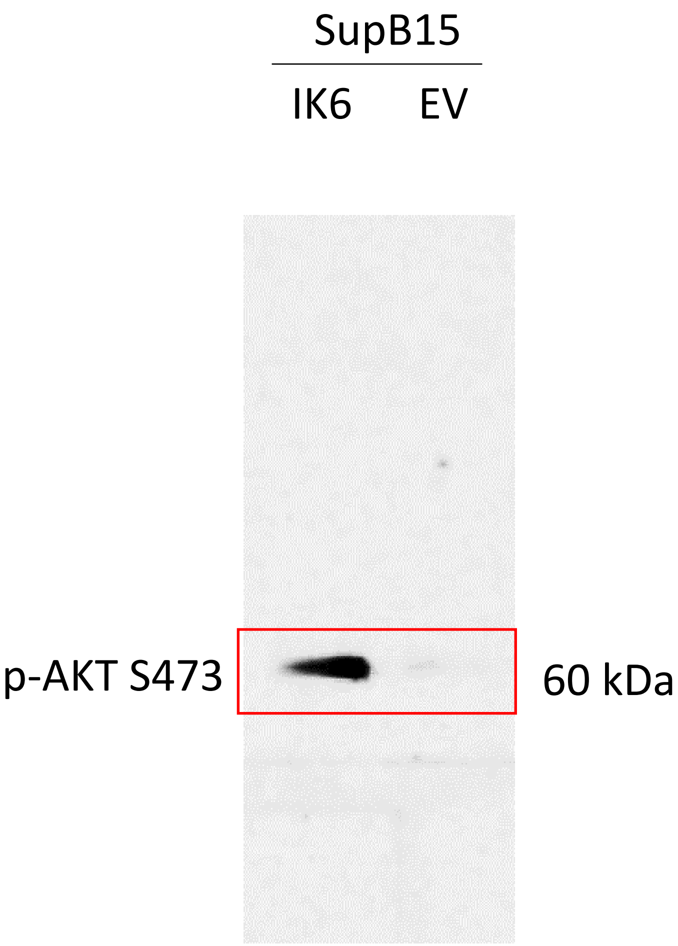
SupB15
IK6
EV
p-AKT S473
60 kDa

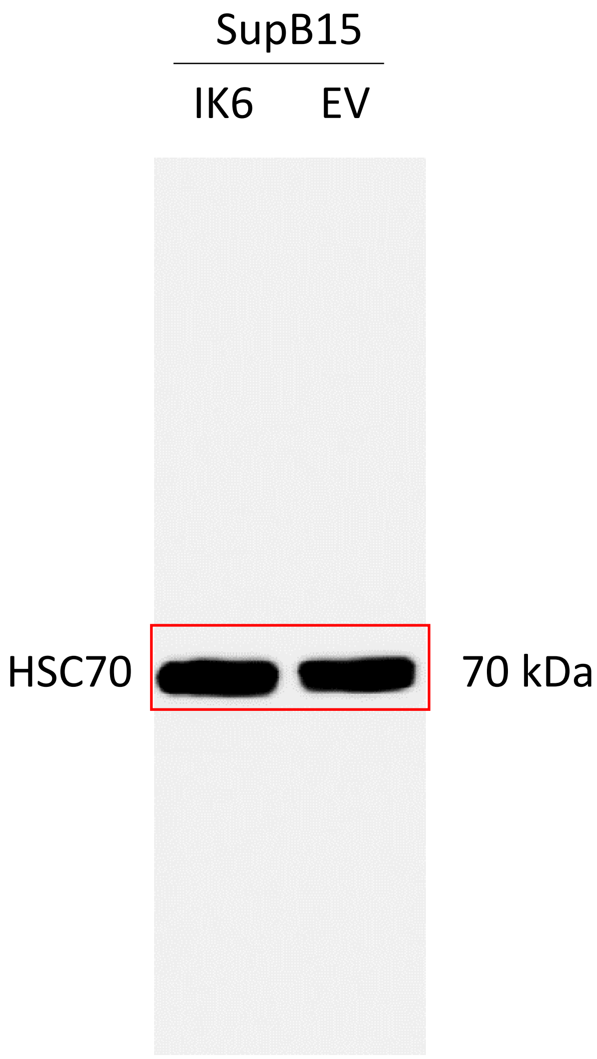
SupB15
IK6
EV
HSC70
70 kDa

Figure 5C

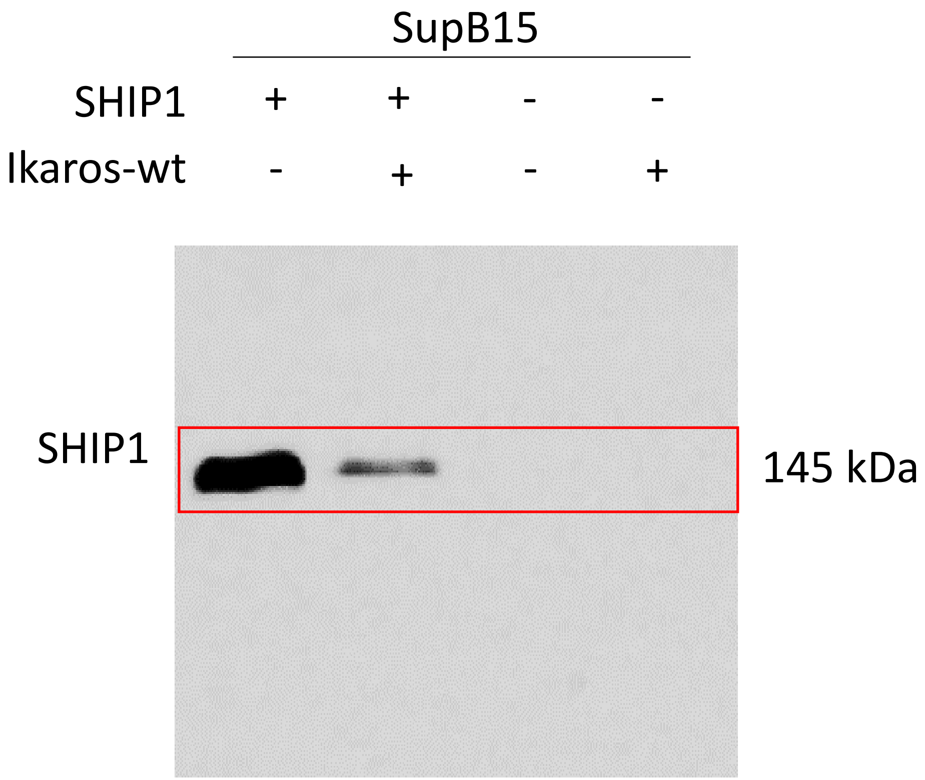
SupB15
SHIP1 + + - -
Ikaros-wt - + - +
SHIP1
145 kDa

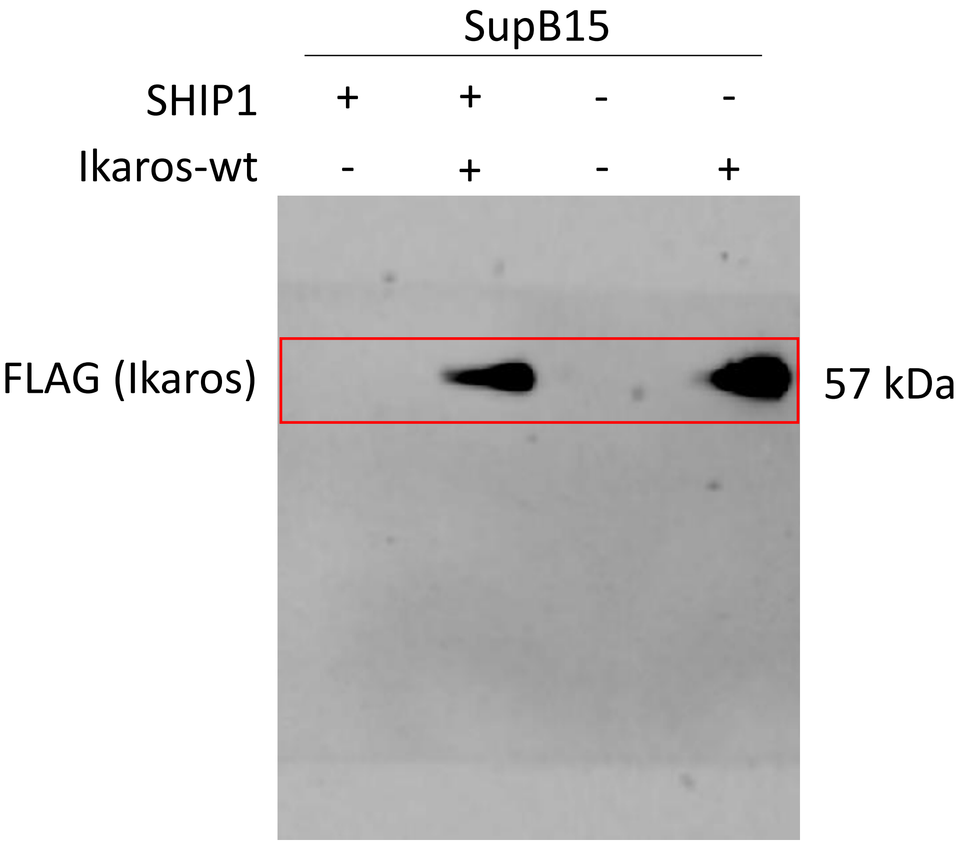
SupB15
SHIP1 + + - -
Ikaros-wt - + - +
FLAG (Ikaros)
57 kDa

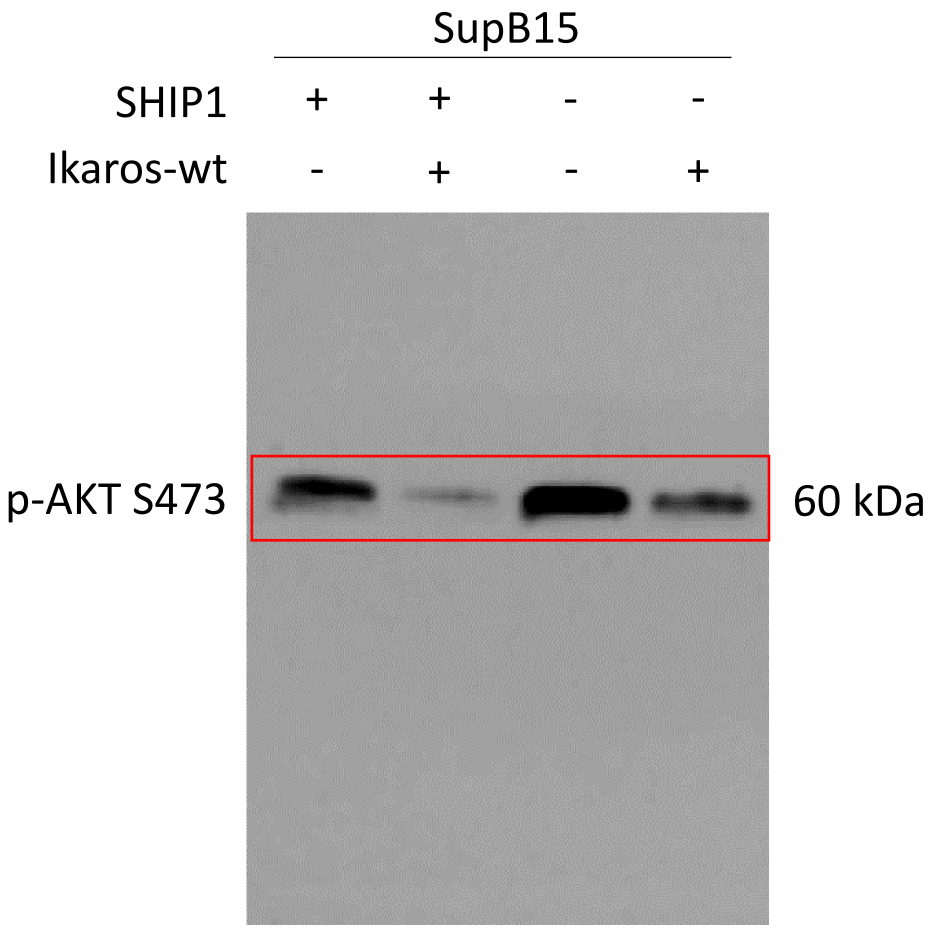
SupB15
SHIP1 + + - -
Ikaros-wt - + - +
p-AKT S473
60 kDa

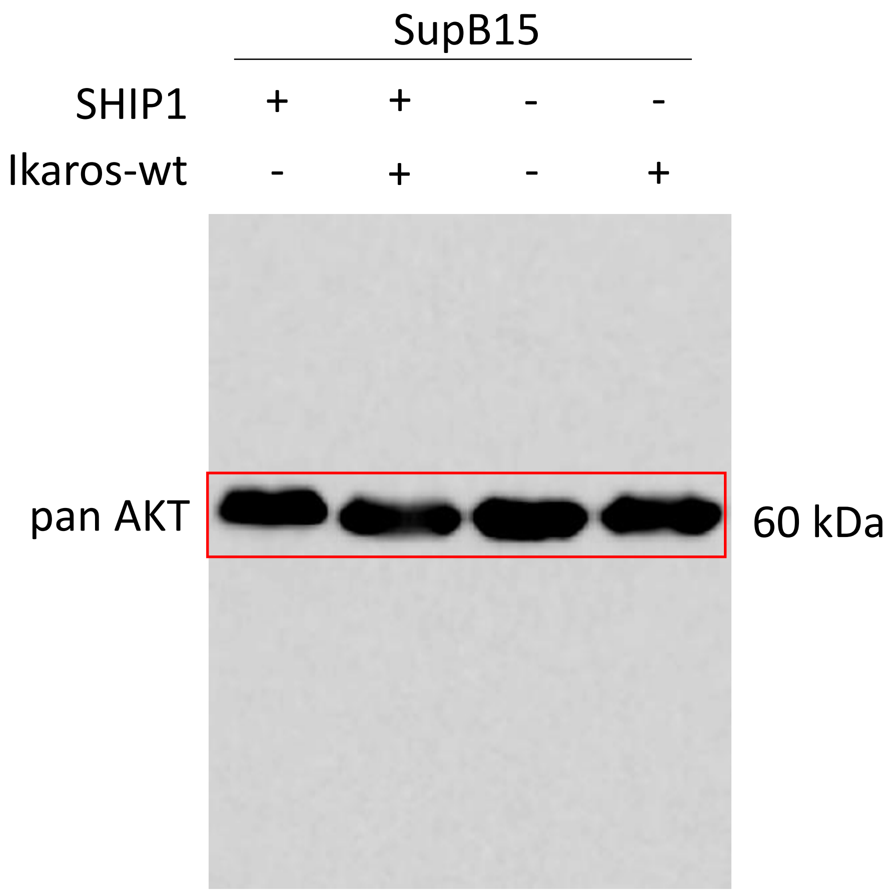
SupB15
SHIP1 + + - -
Ikaros-wt - + - +
pan AKT
60 kDa

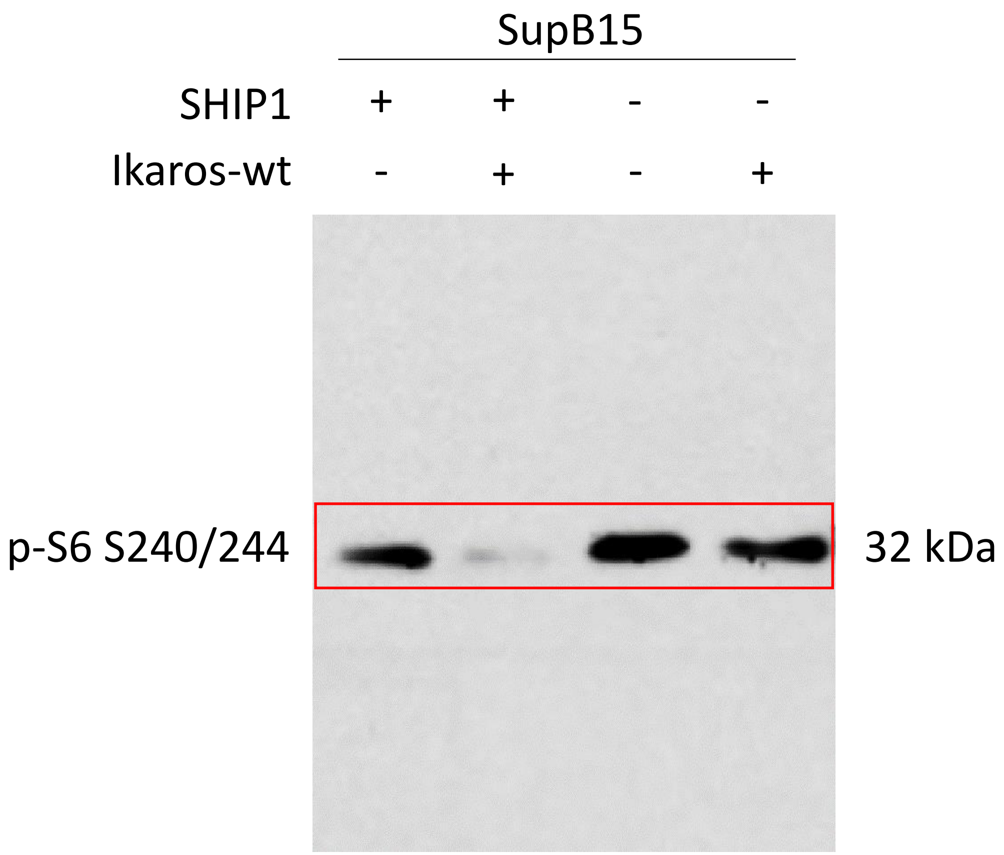
SupB15
SHIP1 + + - -
Ikaros-wt - + - +
p-S6 S240/244
32 kDa

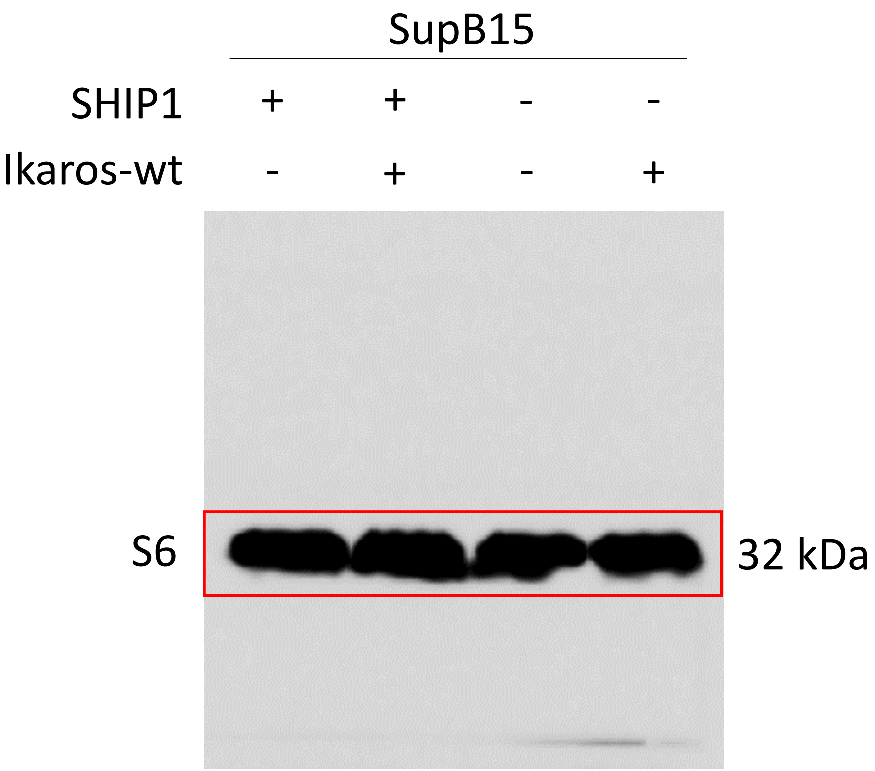
SupB15
SHIP1 + + - -
Ikaros-wt - + - +
S6
32 kDa

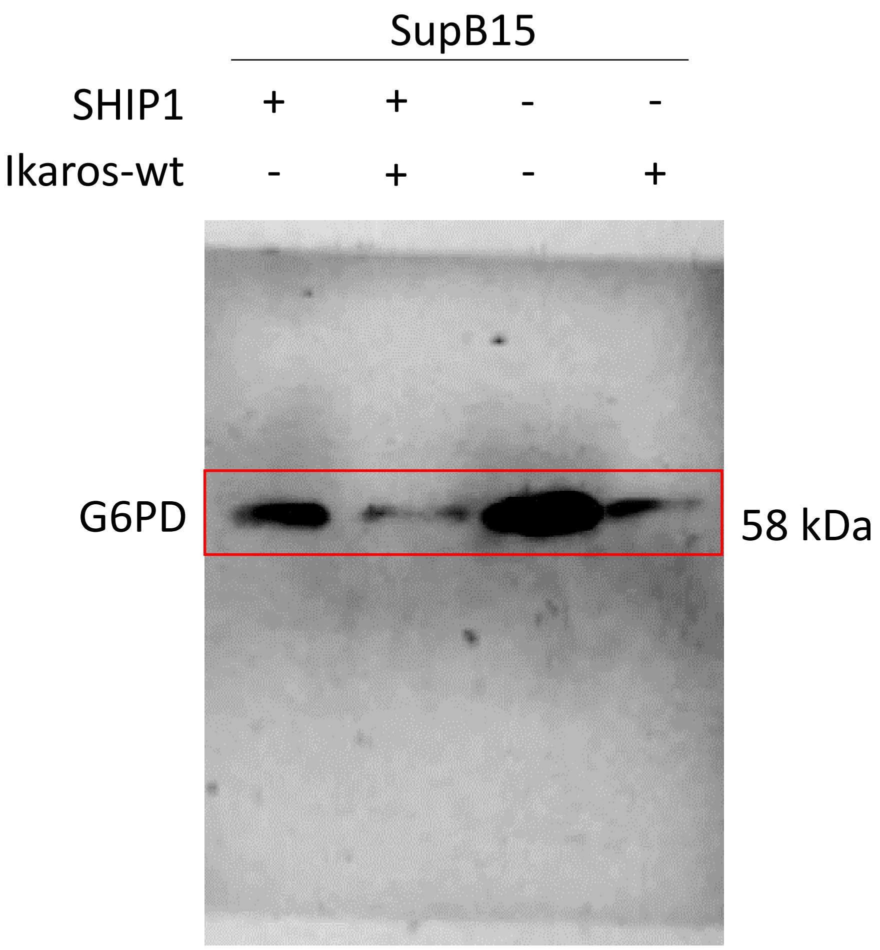
SupB15
SHIP1 + + - -
Ikaros-wt - + - +
G6PD
58 kDa

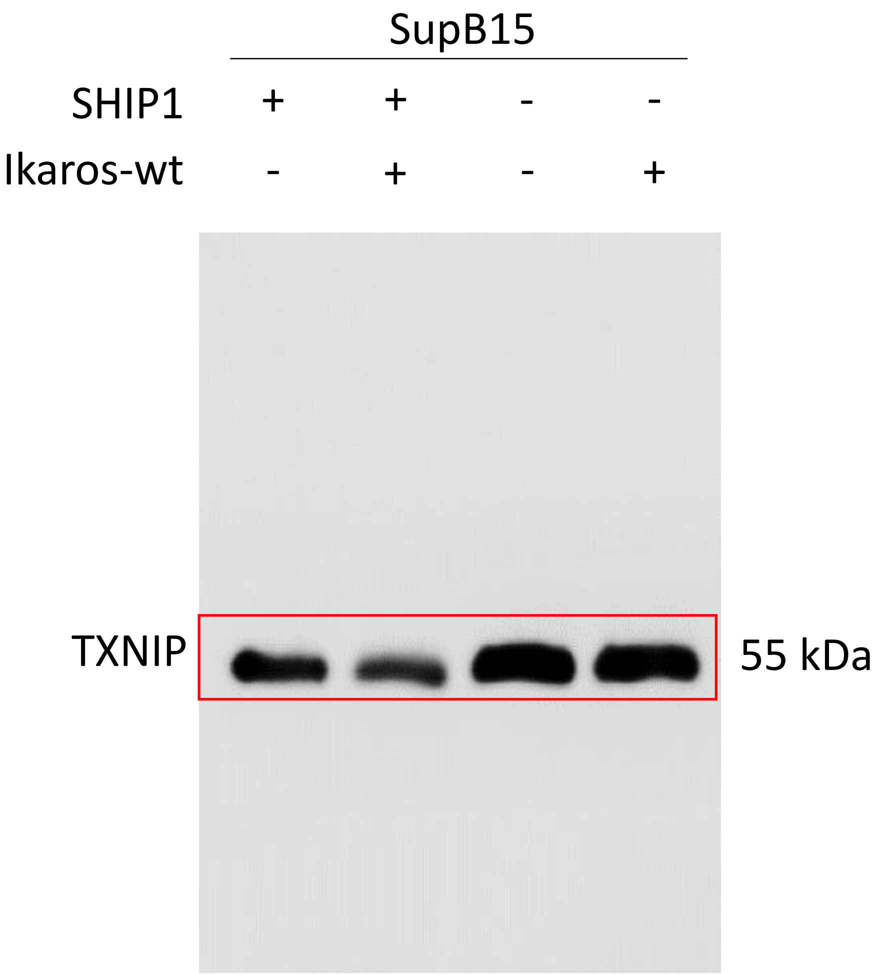
SupB15
SHIP1 + + - -
Ikaros-wt - + - +
TXNIP
55 kDa

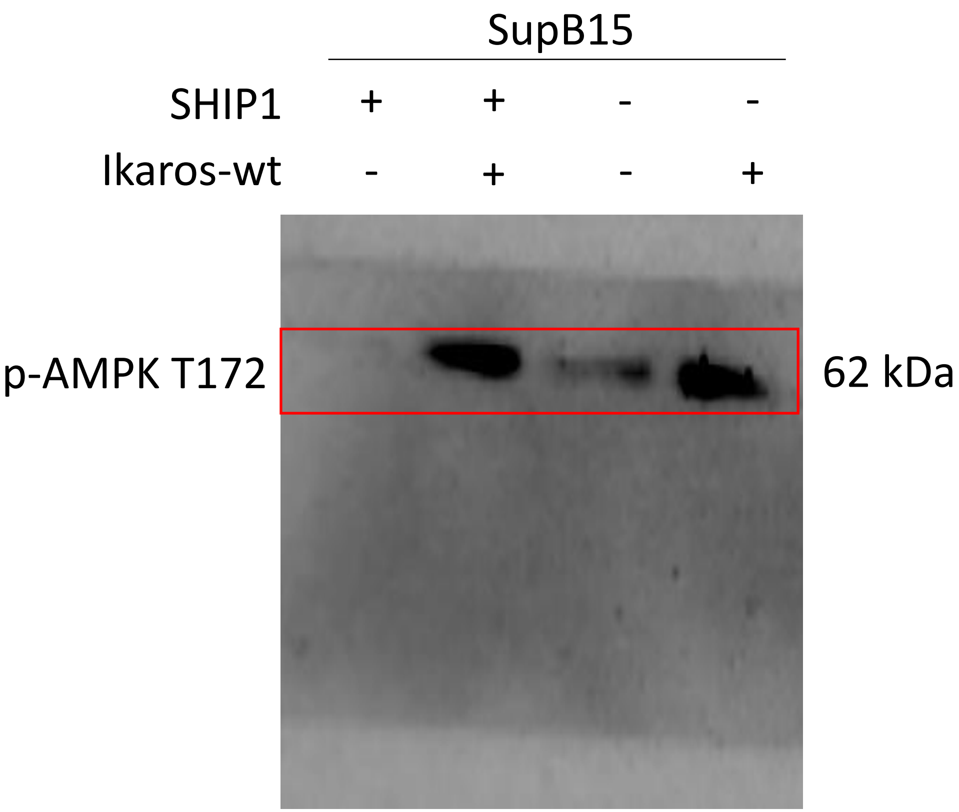
SupB15
SHIP1 + + - -
Ikaros-wt - + - +
p-AMPK T172
62 kDa

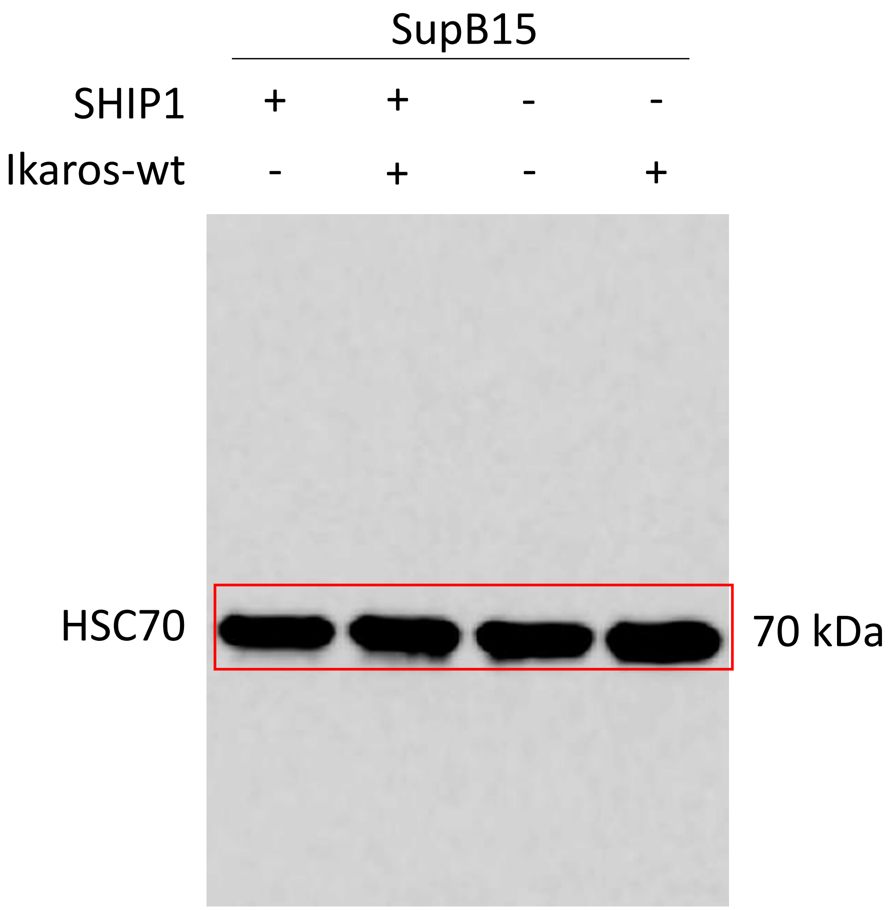
SupB15
SHIP1 + + - -
Ikaros-wt - + - +
HSC70
70 kDa

Figure 6B

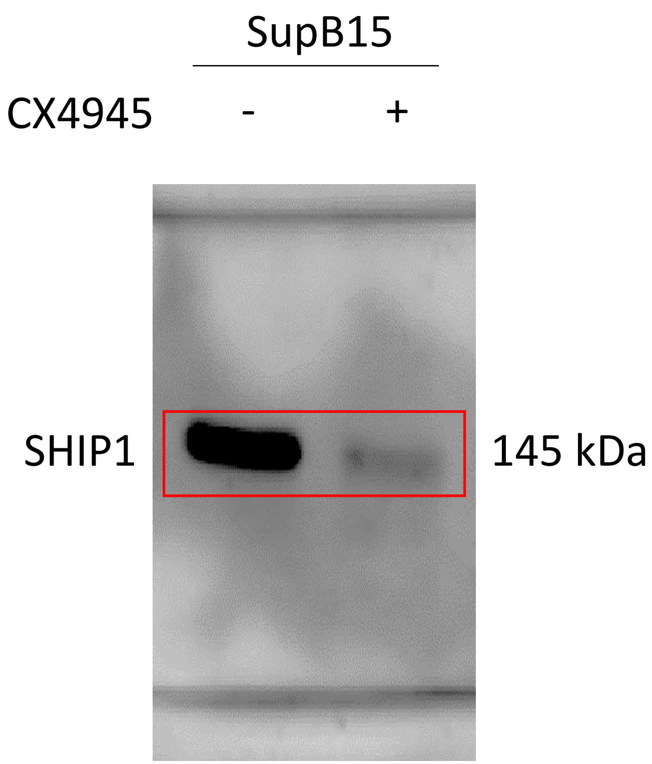
SupB15
CX4945
-
+
SHIP1
145 kDa

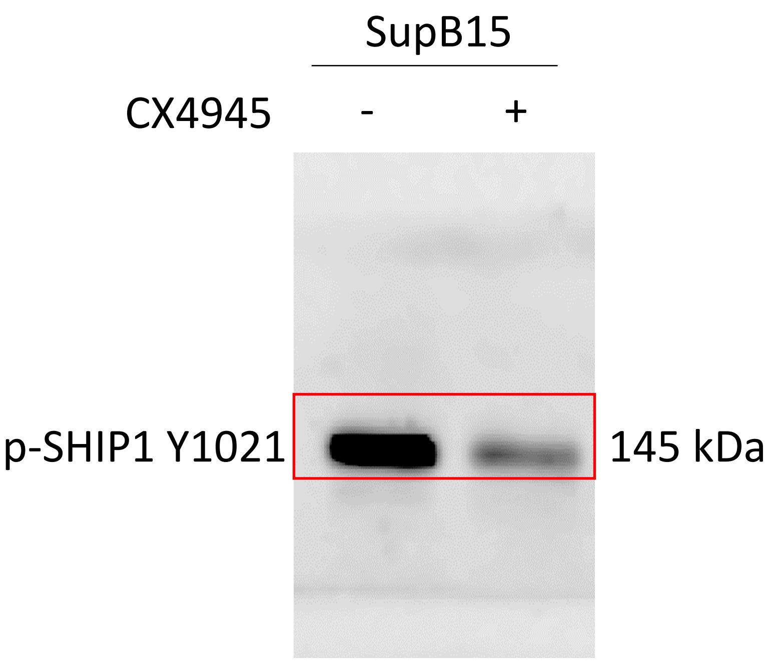
SupB15
CX4945
-
+
p-SHIP1 Y1021
145 kDa

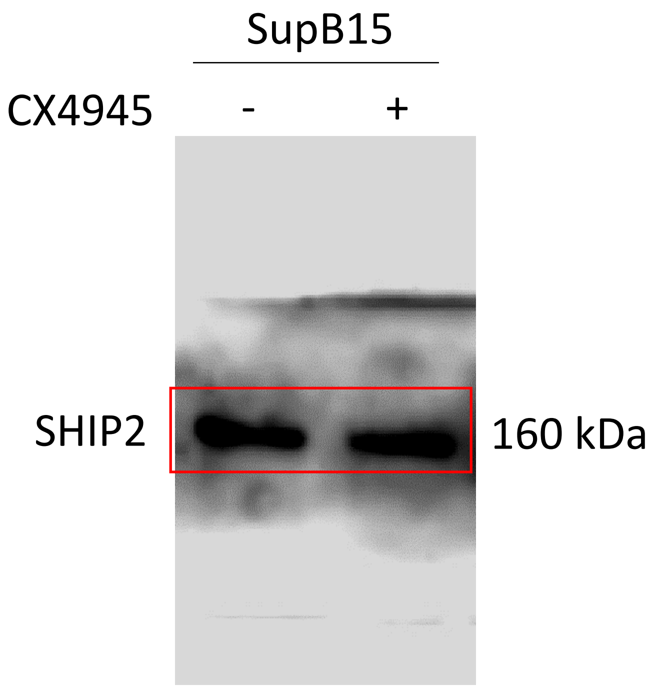
SupB15
CX4945
-
+
SHIP2
160 kDa

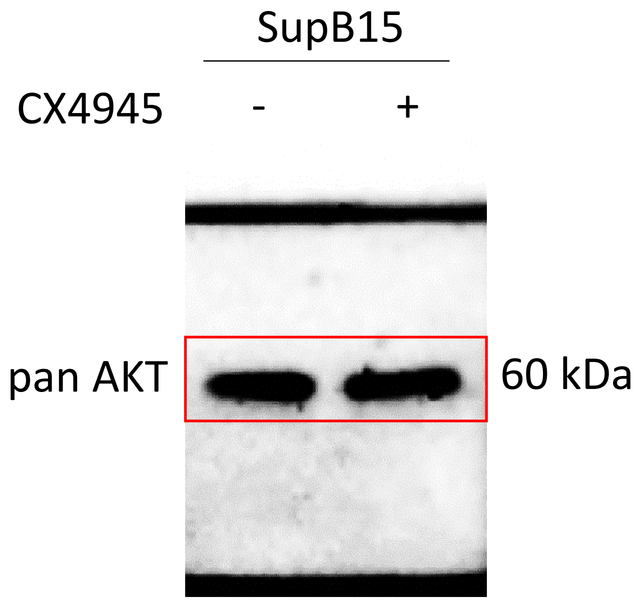
SupB15
CX4945
-
+
pan AKT
60 kDa

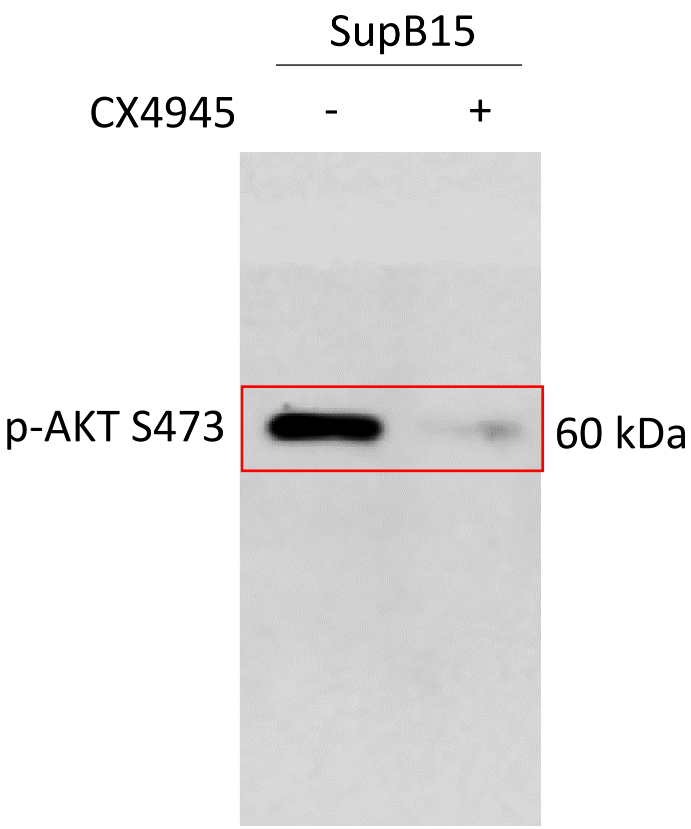
SupB15
CX4945
-
+
p-AKT S473
60 kDa

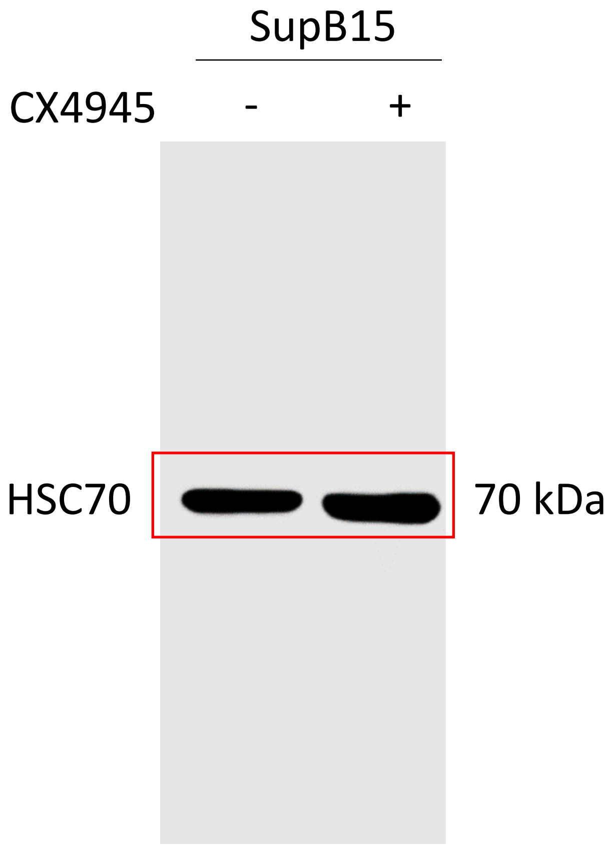
SupB15
CX4945
-
+
HSC70
70 kDa

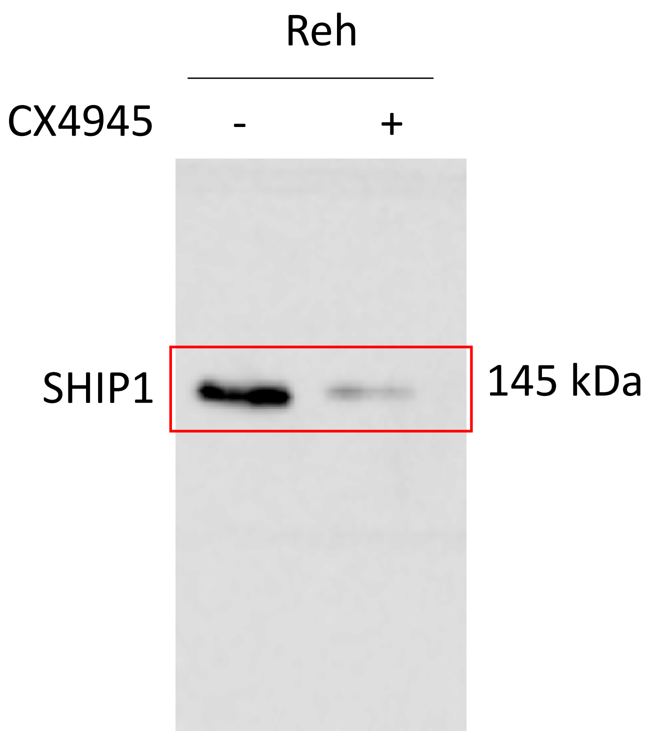
Reh
CX4945
-
+
SHIP1
145 kDa

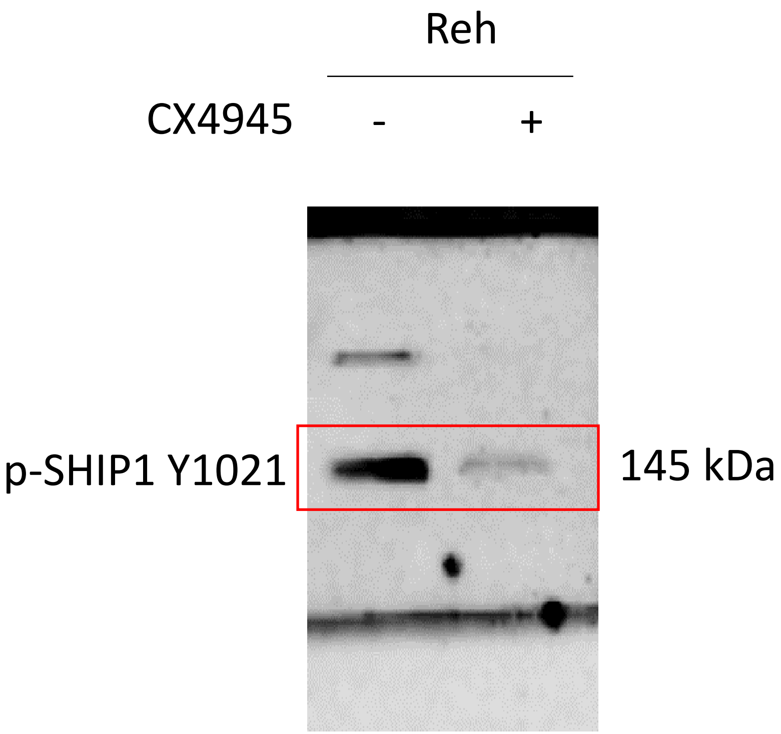
Reh
CX4945
-
+
p-SHIP1 Y1021
145 kDa

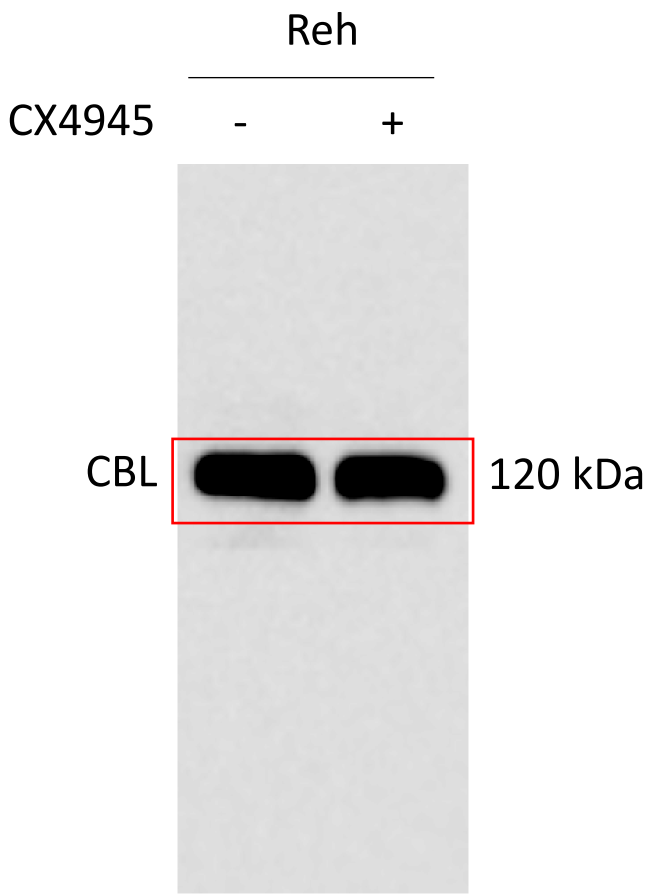
Reh
CX4945
-
+
CBL
120 kDa

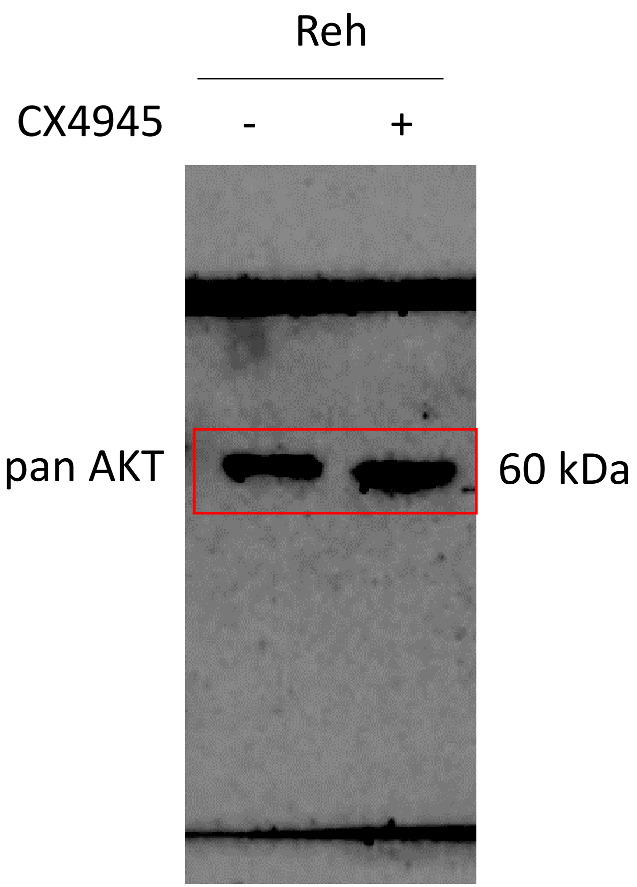

Reh
CX4945
-
+
pan AKT
60 kDa

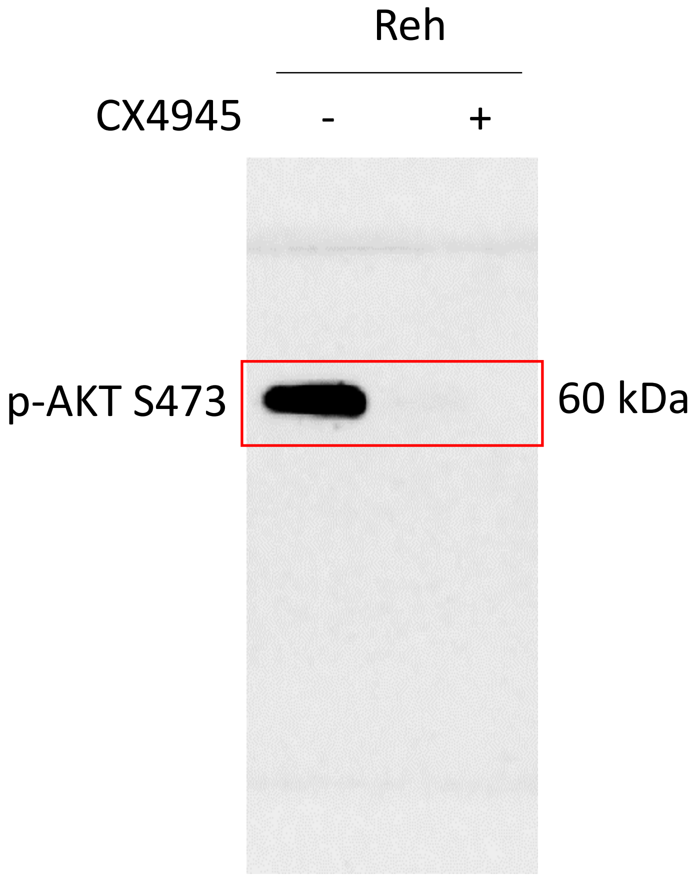
Reh
CX4945
-
+
p-AKT S473
60 kDa

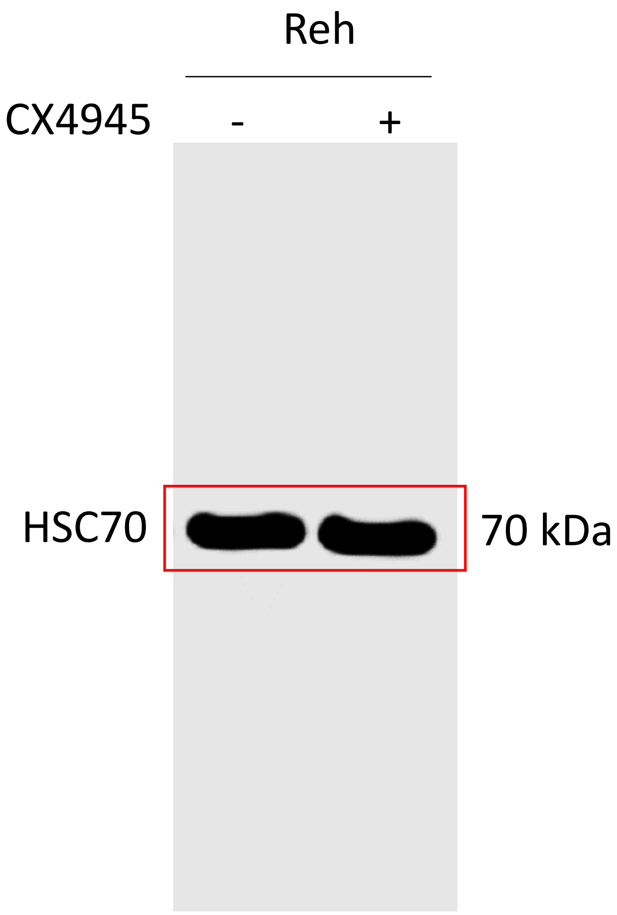
Reh
CX4945
-
+
HSC70
70 kDa

Figure 6E

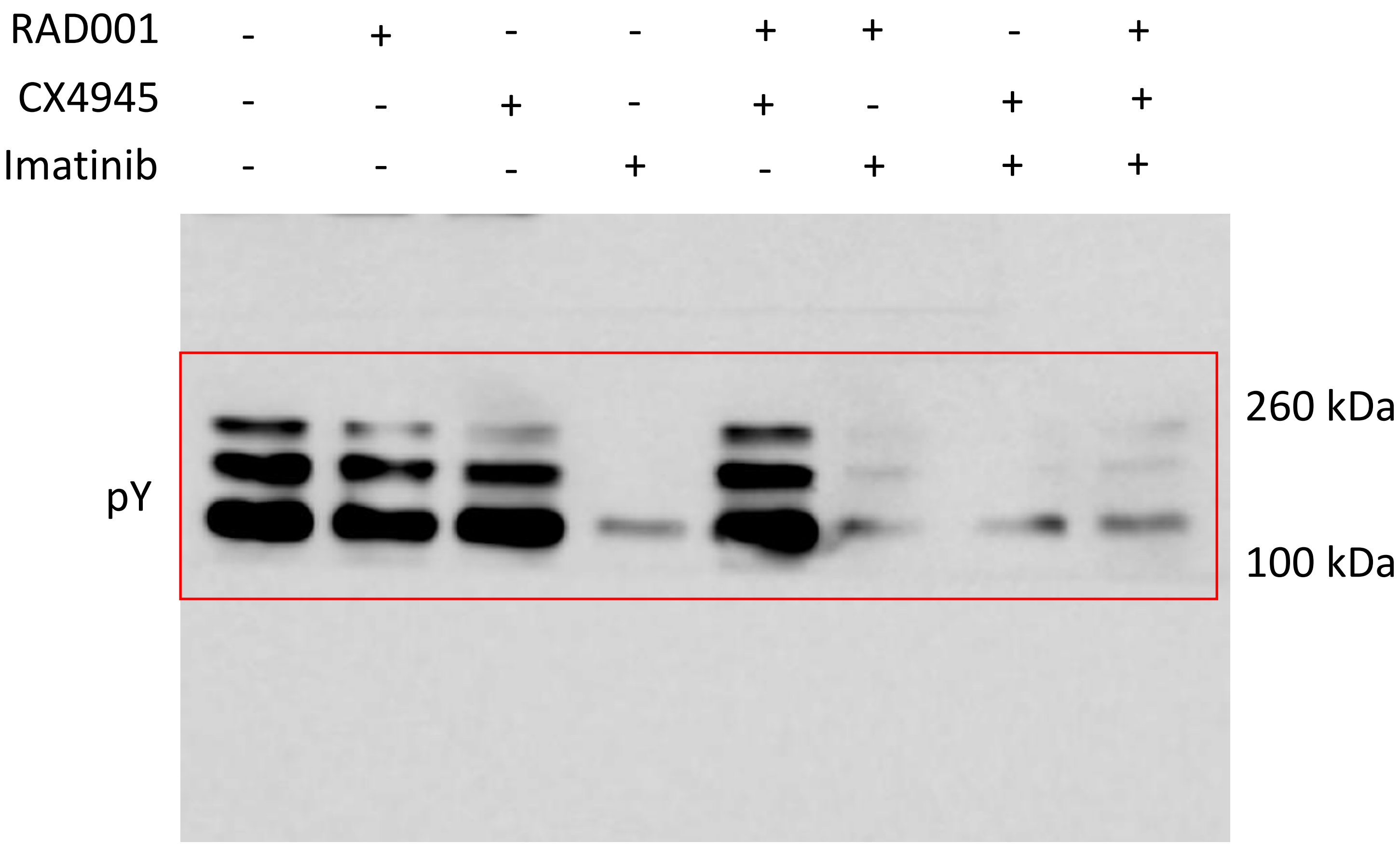
RAD001 - + - - + + - +
CX4945 - - + - + - + +
Imatinib - - - + - + + +
pY
260 kDa
100 kDa

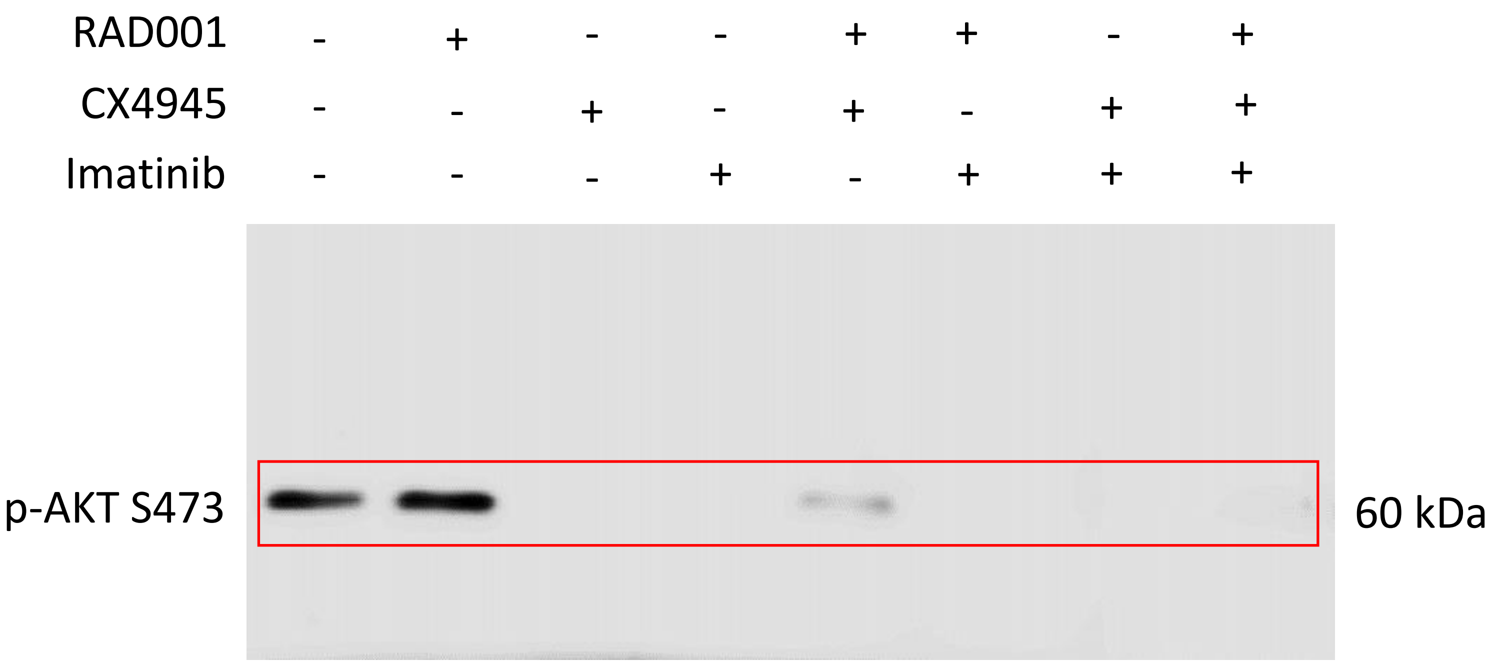

RAD001 - + - - + + - +
CX4945 - - + - + - + +
Imatinib - - - + - + + +
p-AKT S473
60 kDa

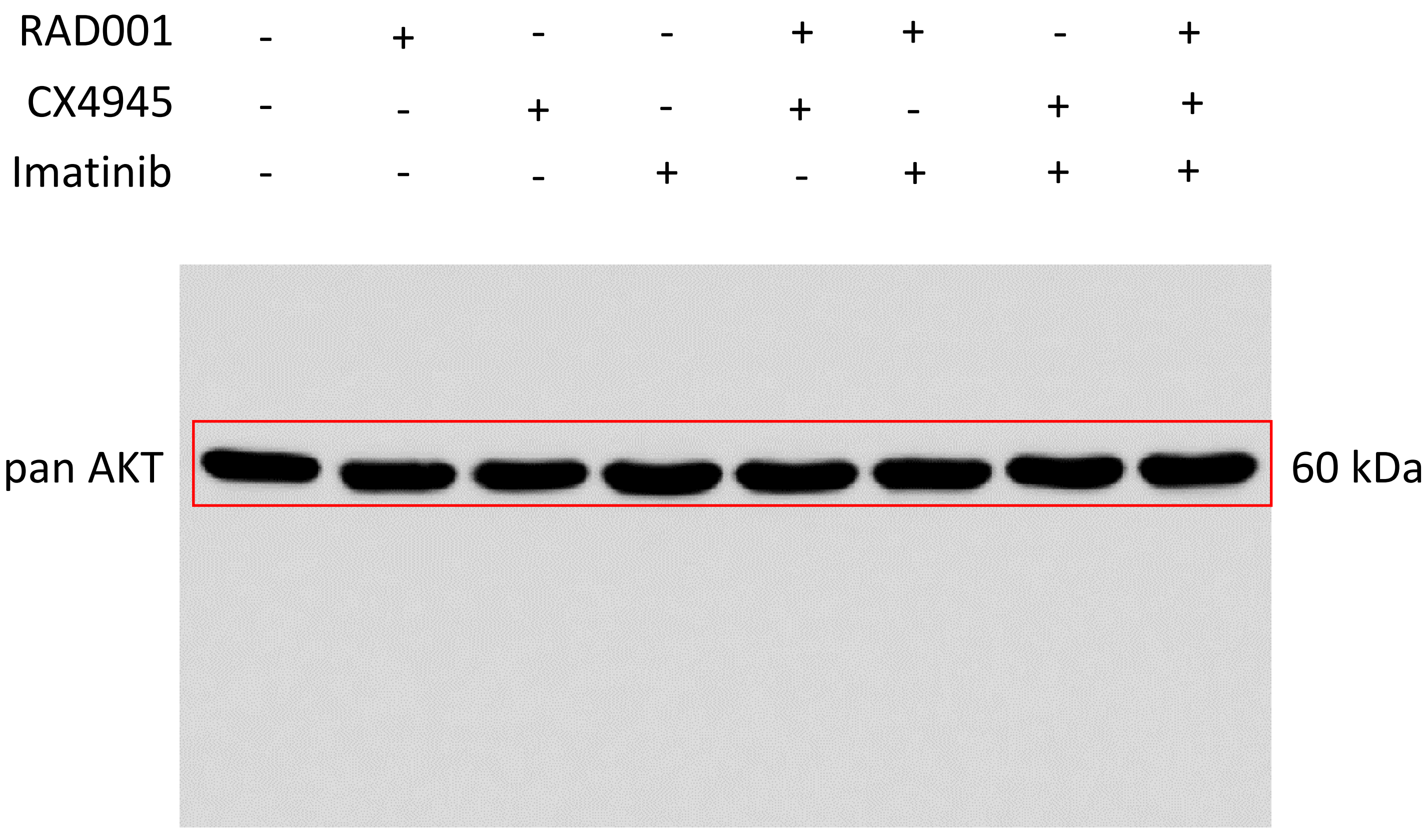
RAD001 - + - - + + - +
CX4945 - - + - + - + +
Imatinib - - - + - + + +
pan AKT
60 kDa

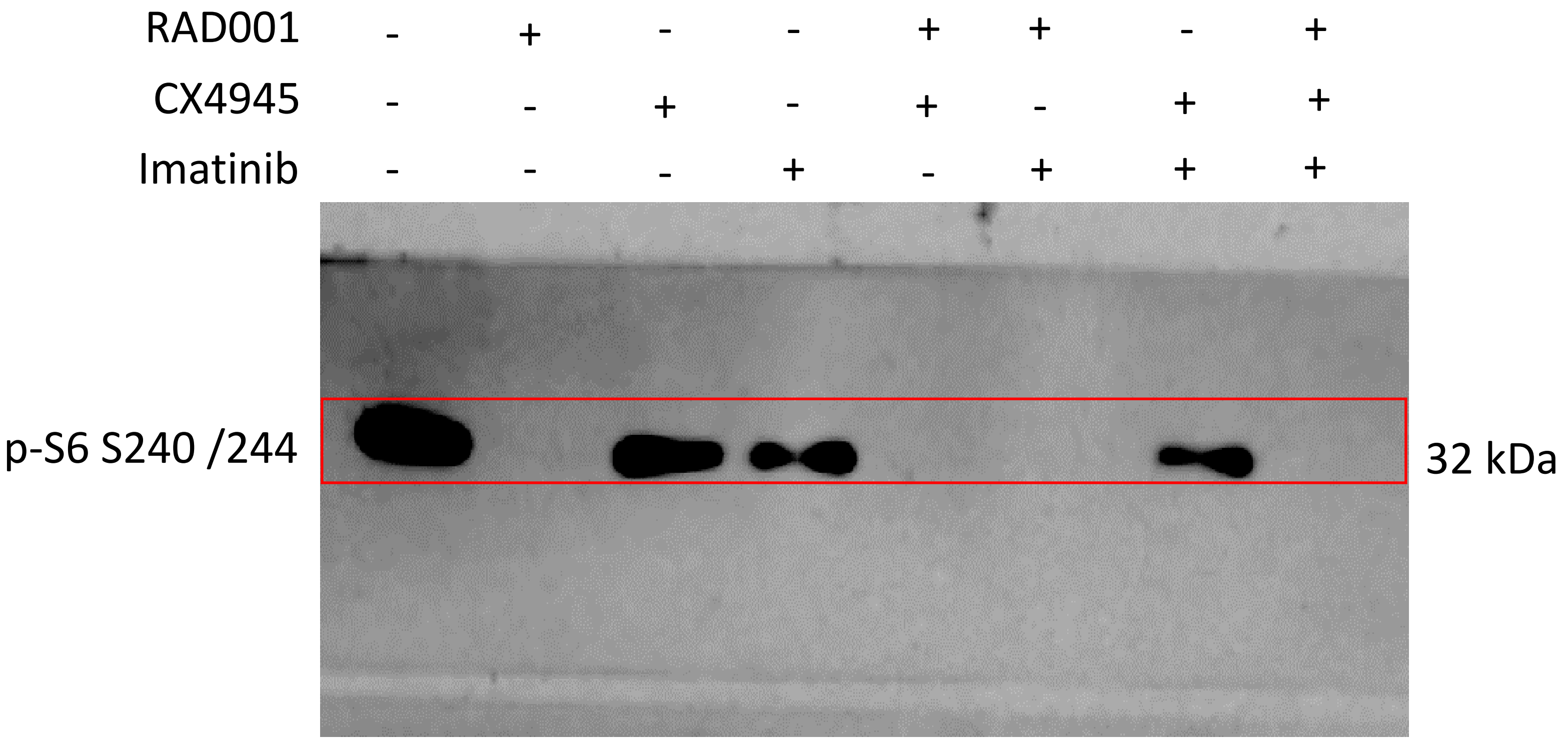
RAD001 - + - - + + - +
CX4945 - - + - + - + +
Imatinib - - - + - + + +
p-S6 S240 /244
32 kDa

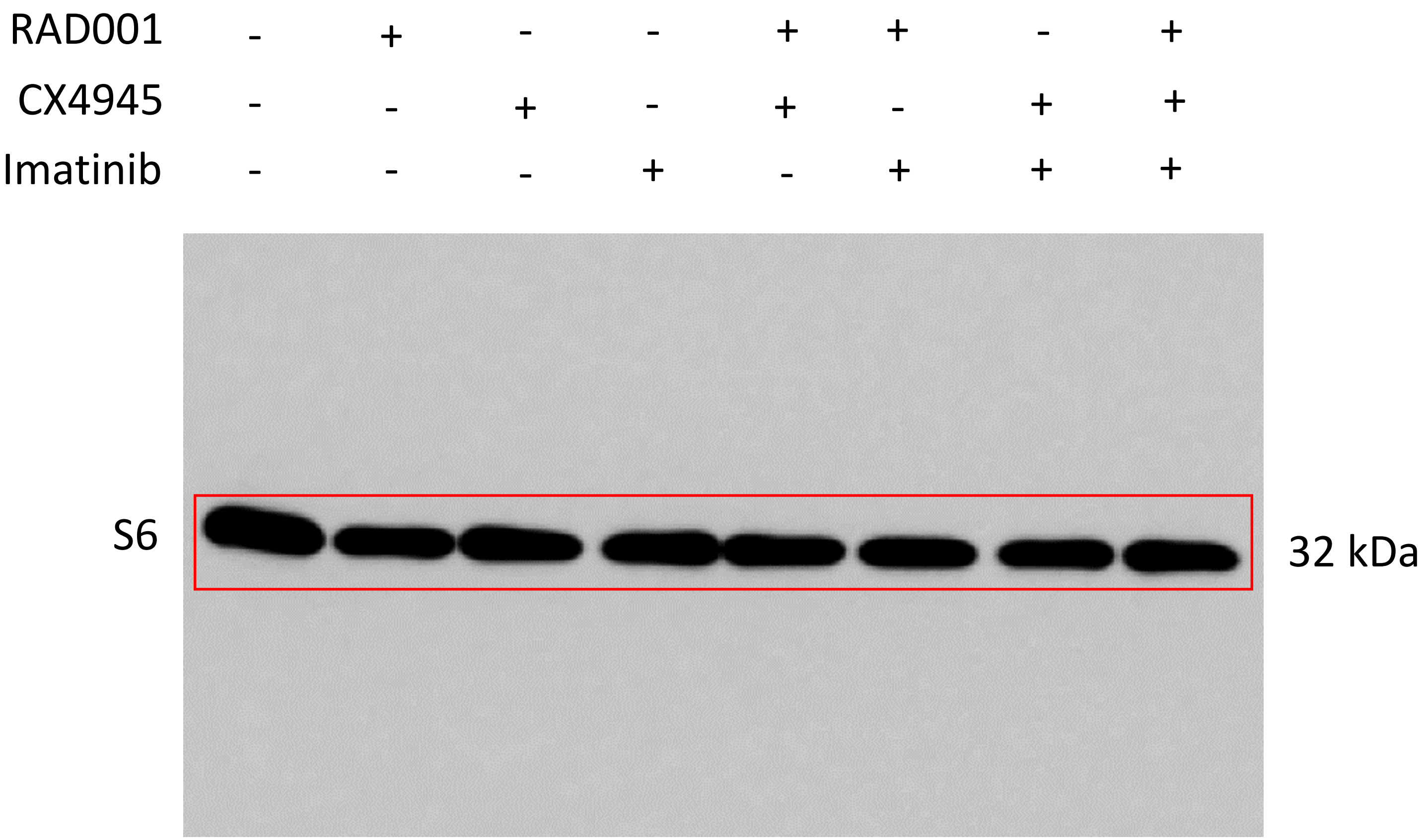
RAD001 - + - - + + - +
CX4945 - - + - + - + +
Imatinib - - - + - + + +
S6
32 kDa

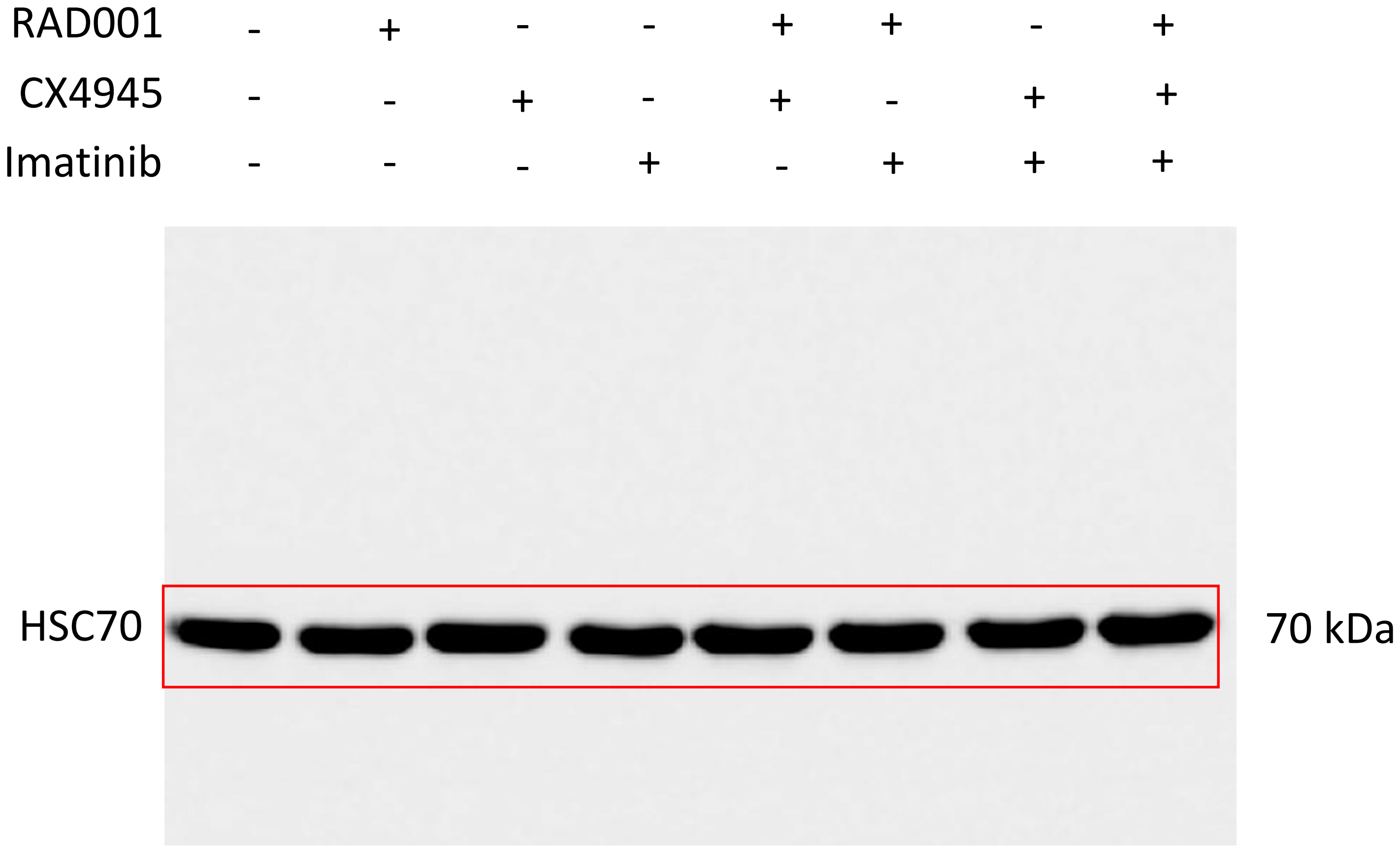
RAD001 - + - - + + - +
CX4945 - - + - + - + +
Imatinib - - - + - + + +
HSC70
70 kDa

Figure 7A

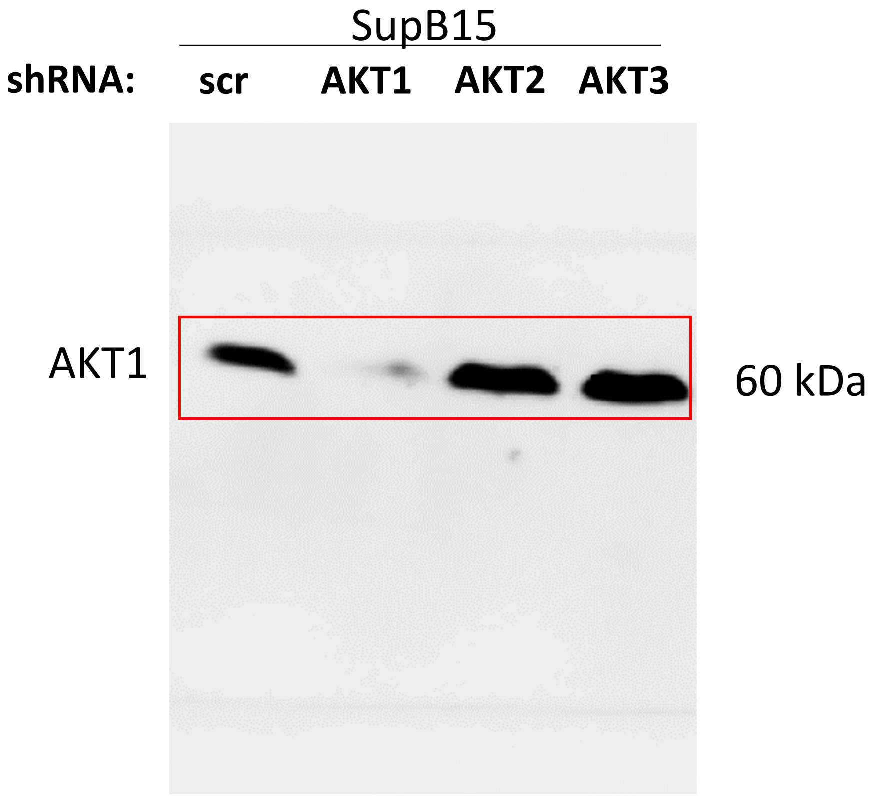
SupB15
shRNA:
scr
AKT1
AKT2
AKT3
AKT1
60 kDa

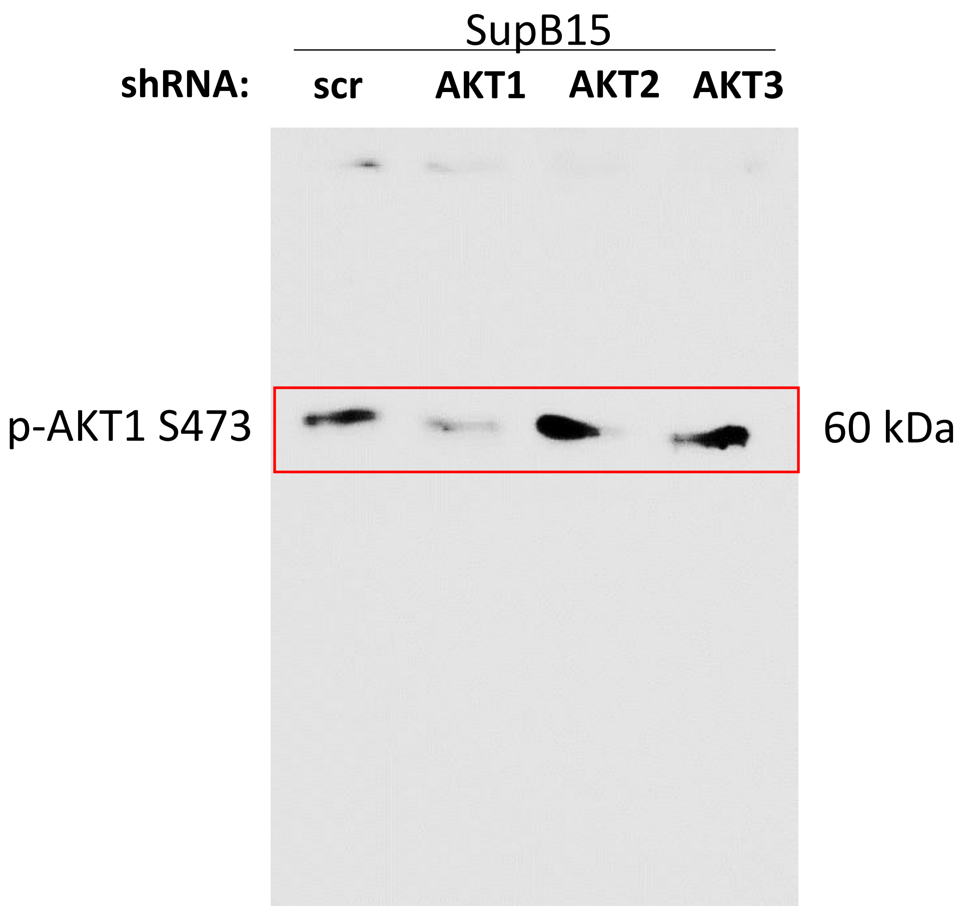
SupB15
shRNA:
scr
AKT1
AKT2
AKT3
p-AKT1 S473
60 kDa

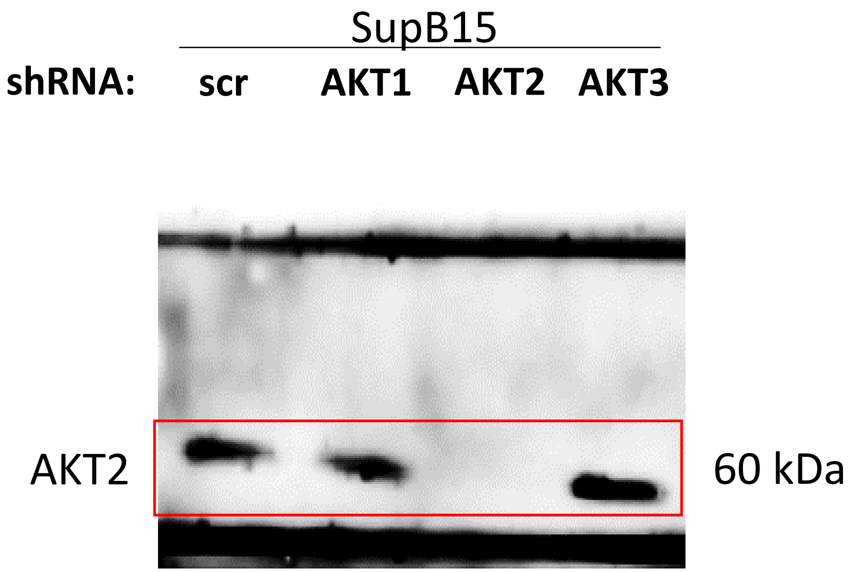
SupB15
shRNA:
scr
AKT1
AKT2
AKT3
AKT2
60 kDa

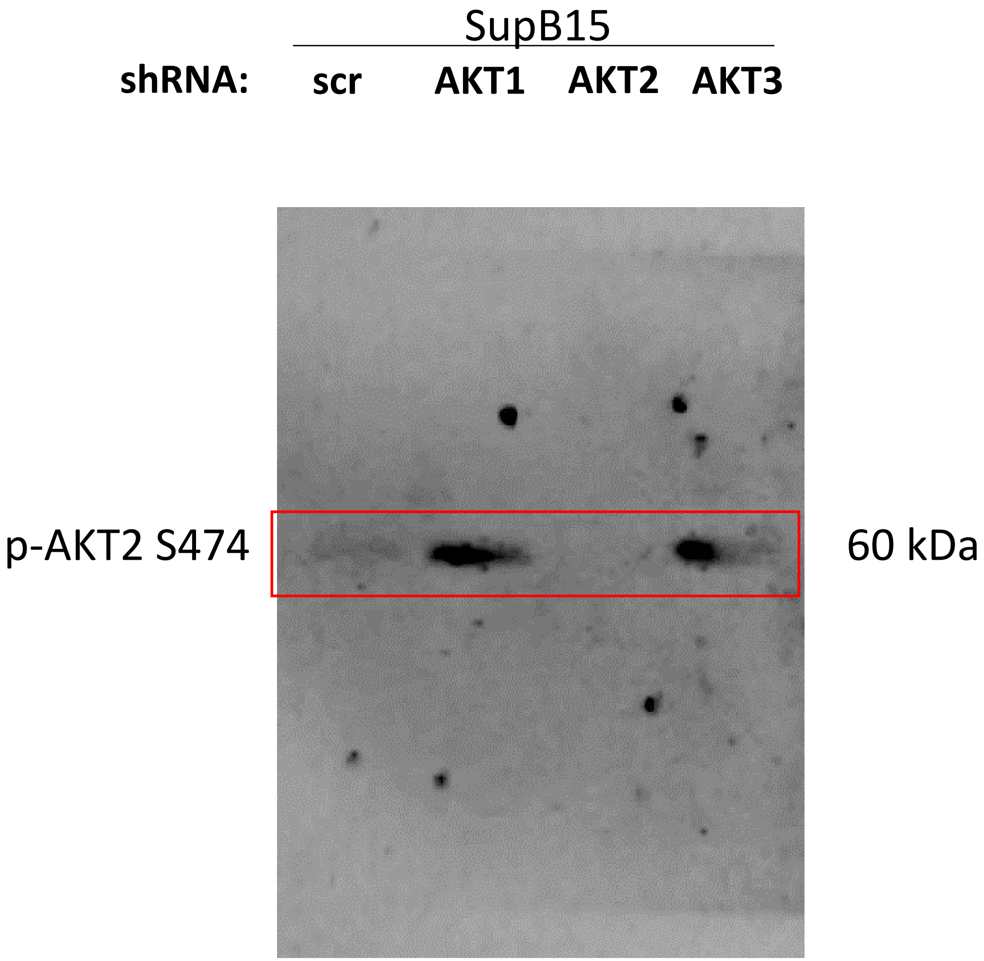
SupB15
shRNA:
scr
AKT1
AKT2
AKT3
p-AKT2 S474
60 kDa

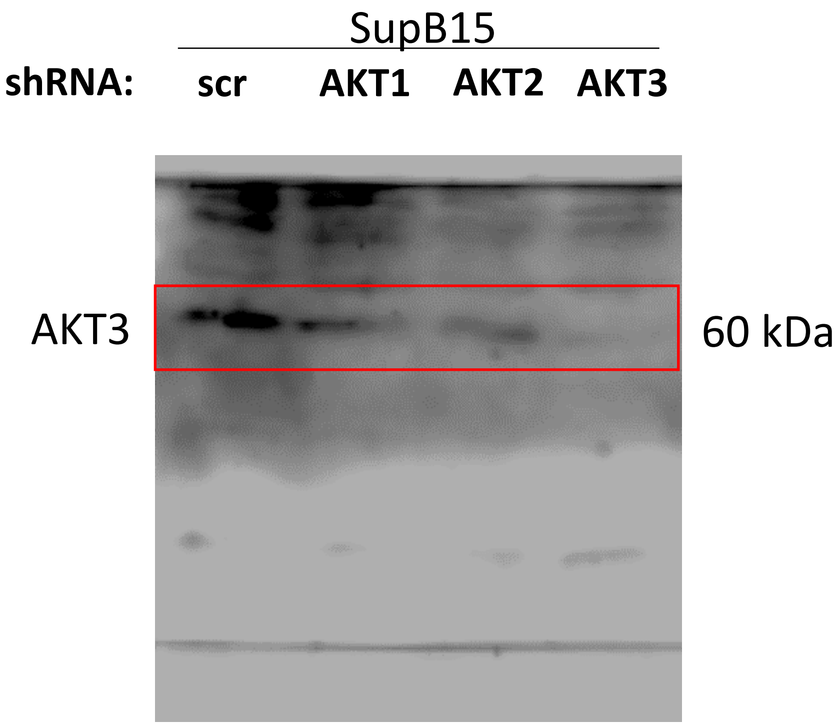

SupB15
shRNA: scr AKT1 AKT2 AKT3
AKT3
60 kDa

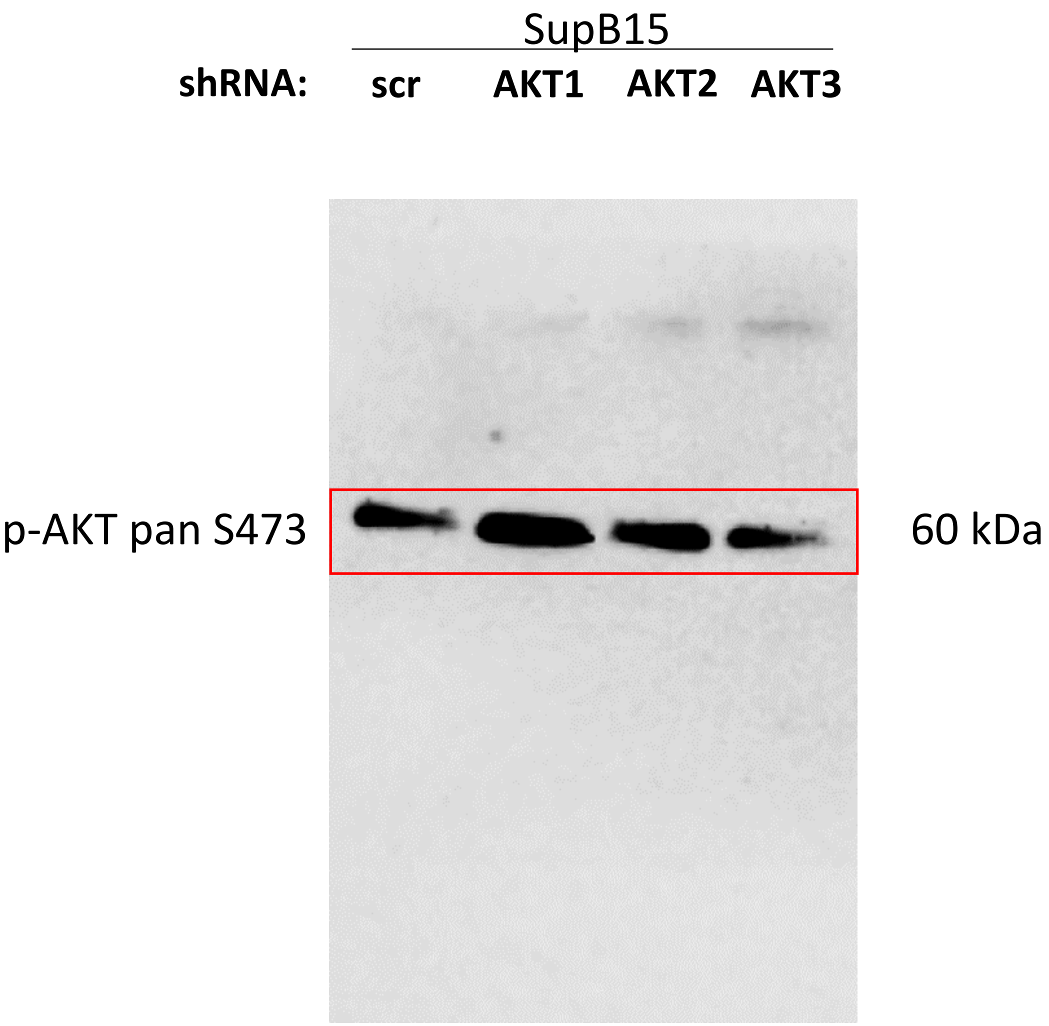
SupB15
shRNA:
scr
AKT1
AKT2
AKT3
p-AKT pan S473
60 kDa

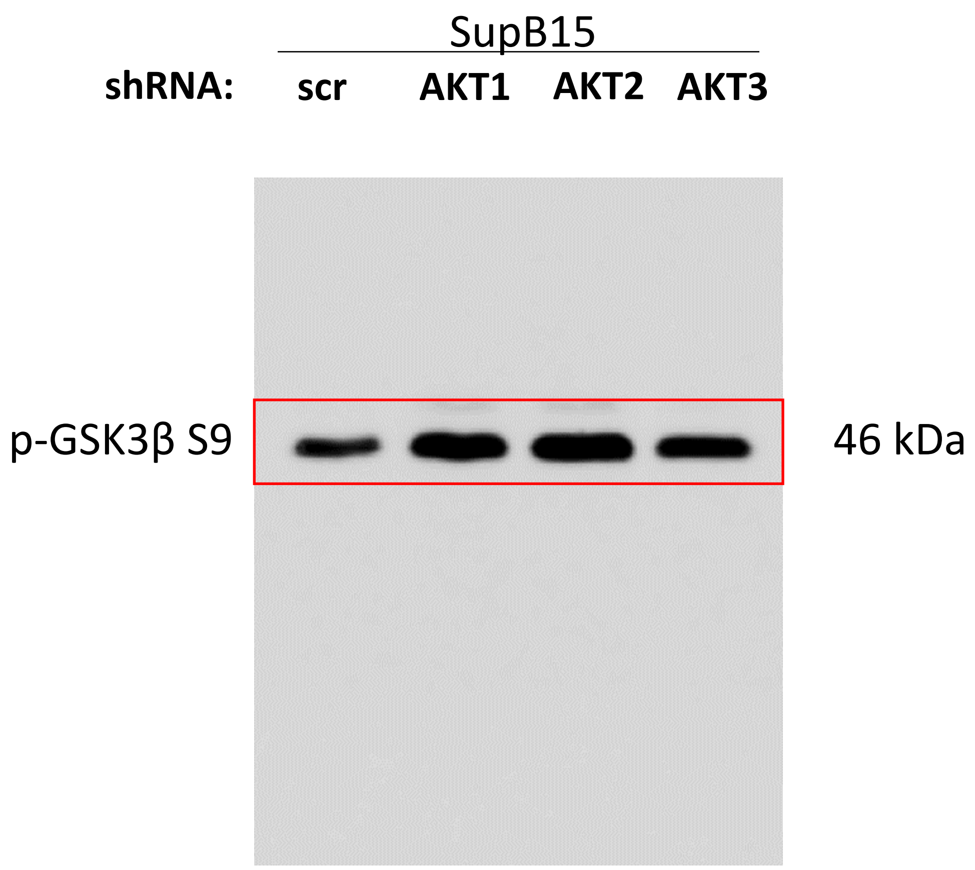
SupB15
shRNA:
scr
AKT1
AKT2
AKT3
p-GSK3β S9
46 kDa

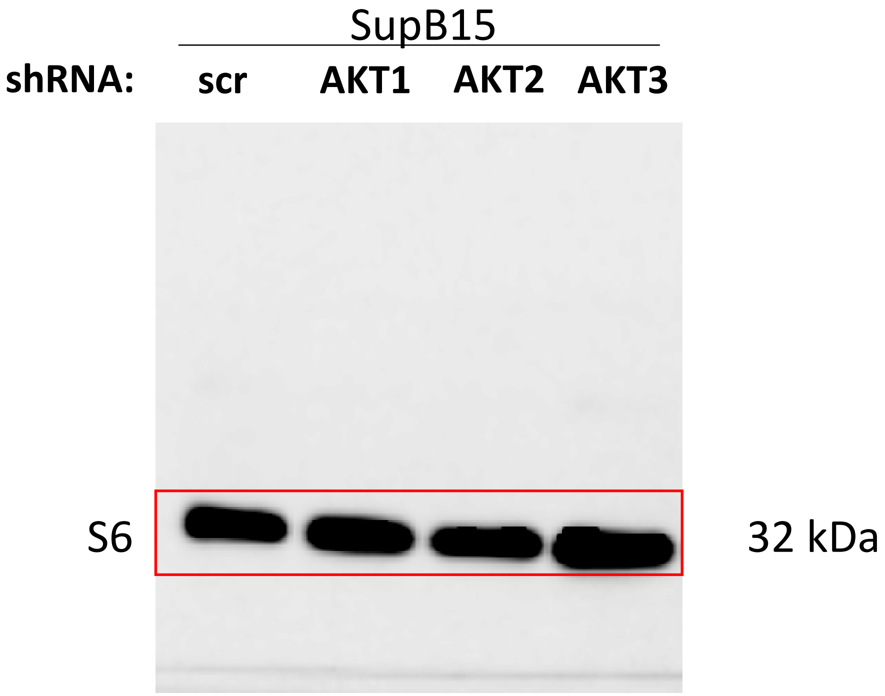
SupB15
shRNA:
scr
AKT1
AKT2
AKT3
S6
32 kDa

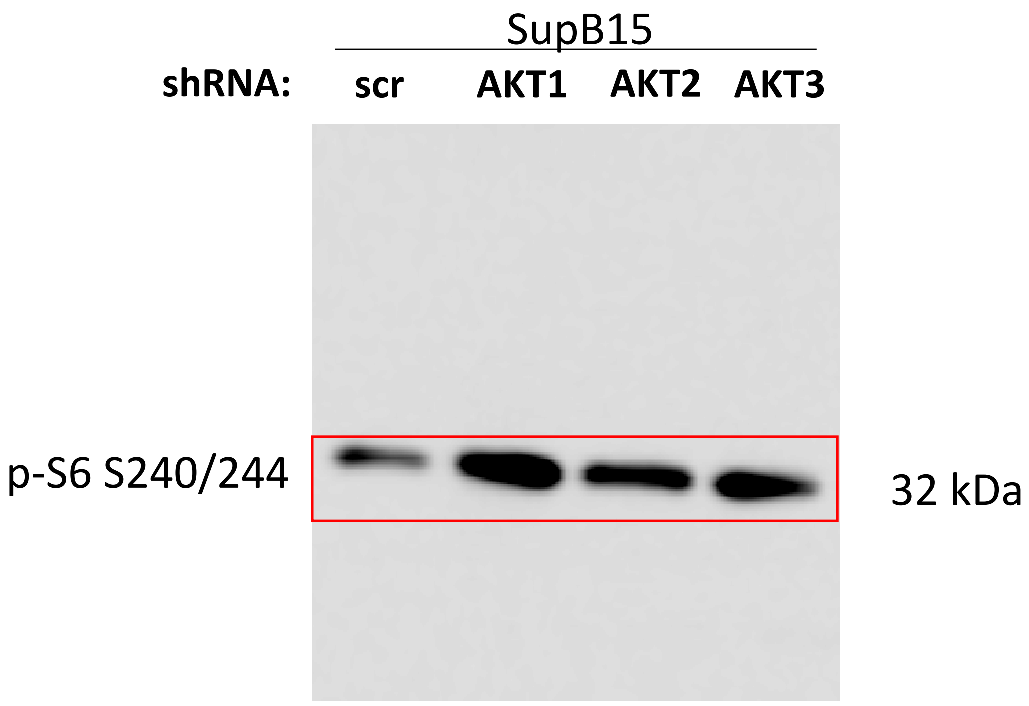
SupB15
shRNA:
scr
AKT1
AKT2
AKT3
p-S6 S240/244
32 kDa

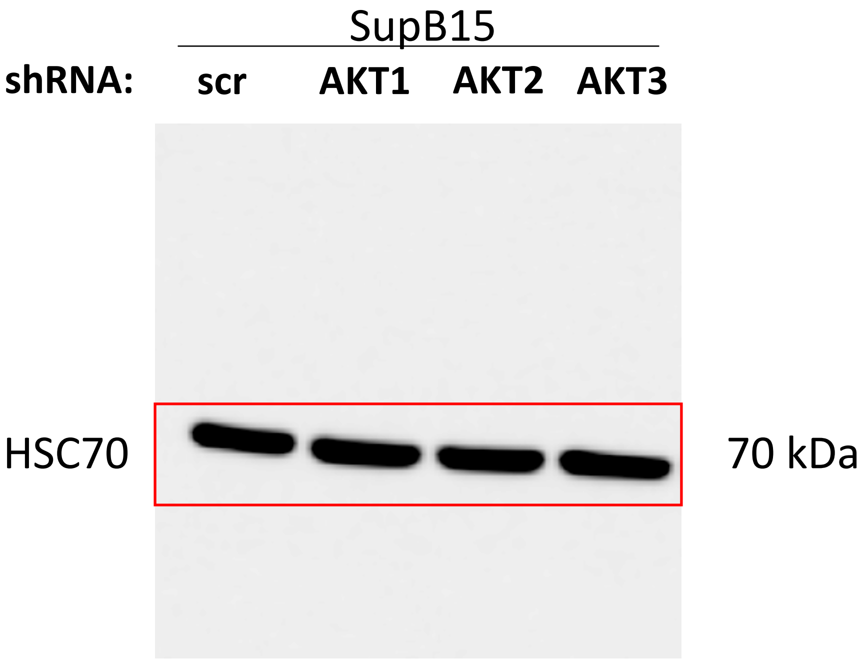
SupB15
shRNA:
scr
AKT1
AKT2
AKT3
HSC70
70 kDa

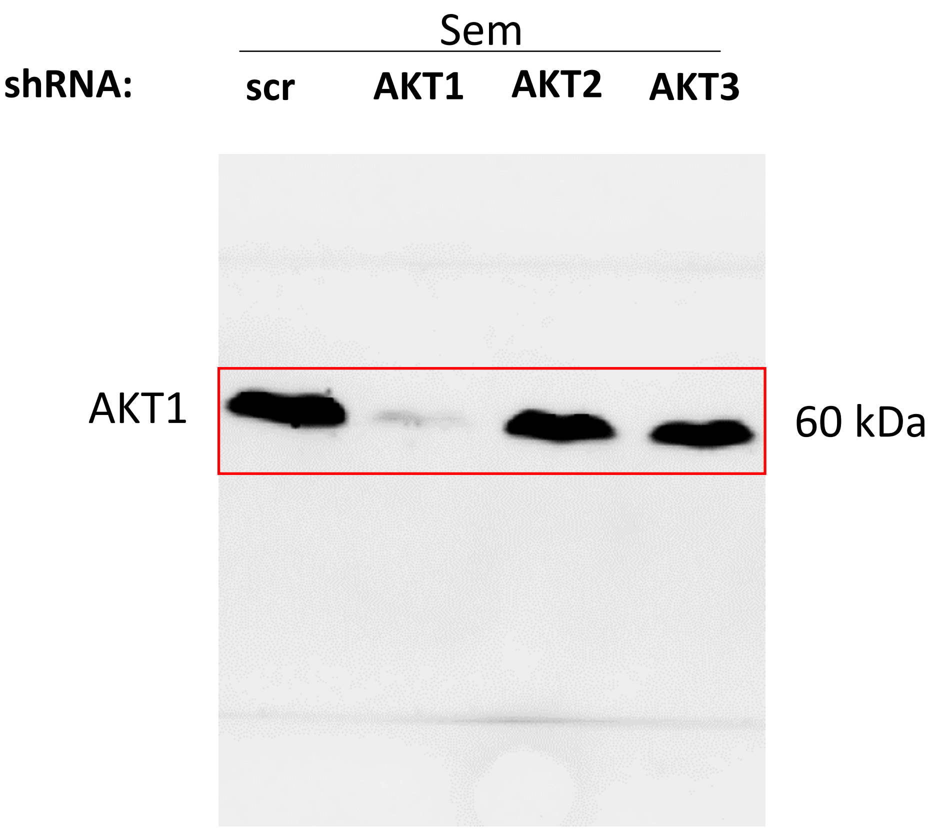
Sem
shRNA:
scr
AKT1
AKT2
AKT3
AKT1
60 kDa

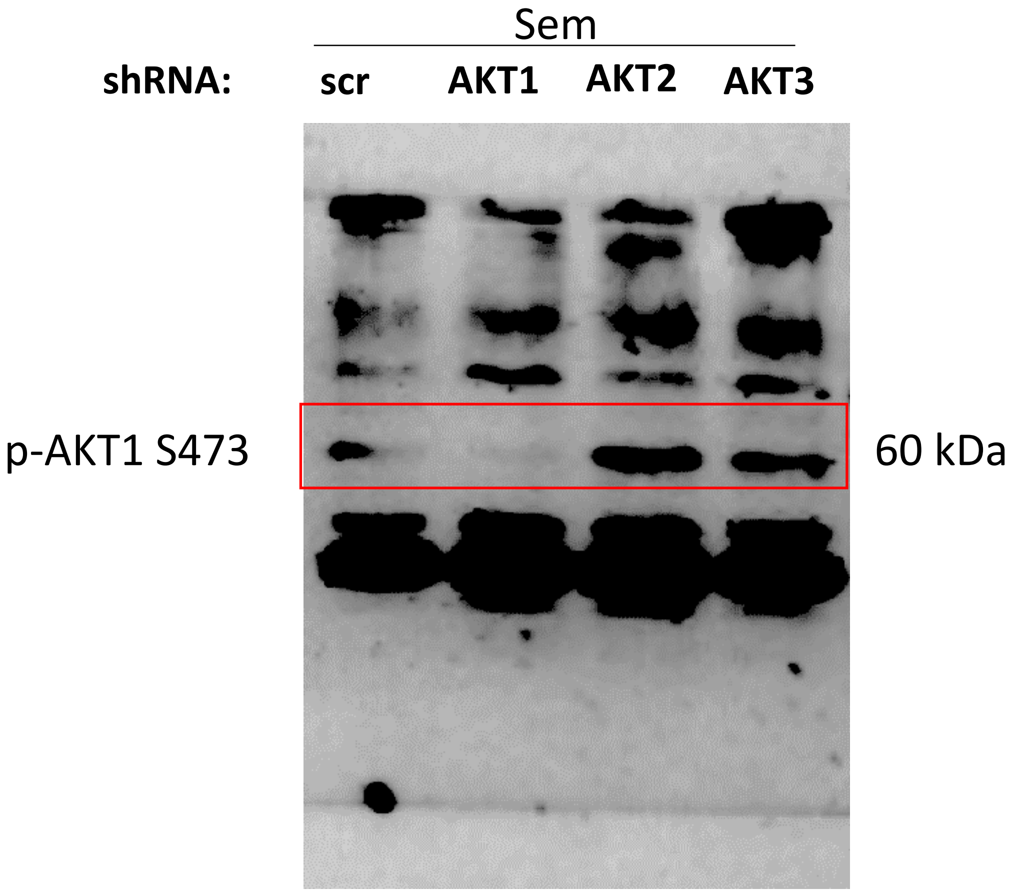
Sem
shRNA:
scr
AKT1
AKT2
AKT3
p-AKT1 S473
60 kDa

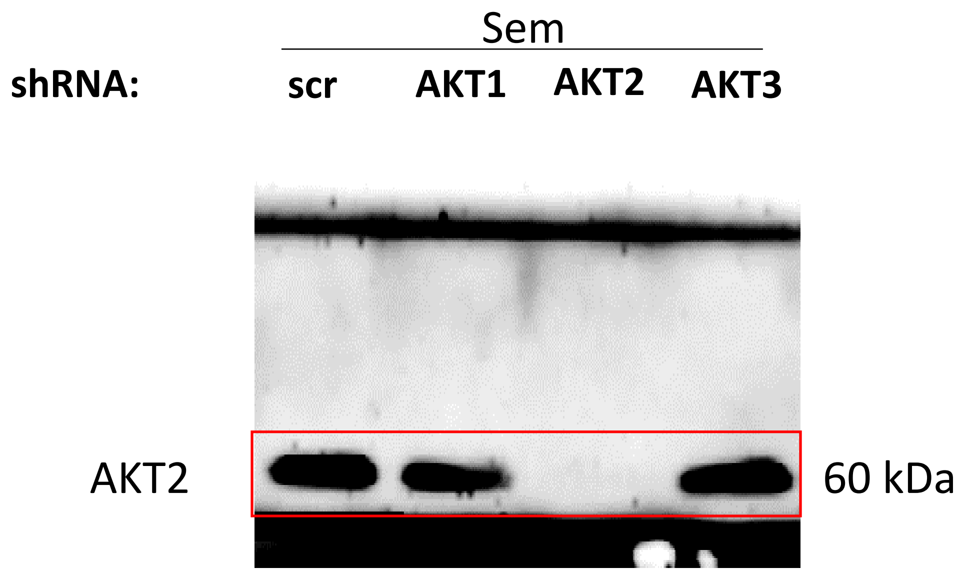
Sem
shRNA:
scr
AKT1
AKT2
AKT3
AKT2
60 kDa

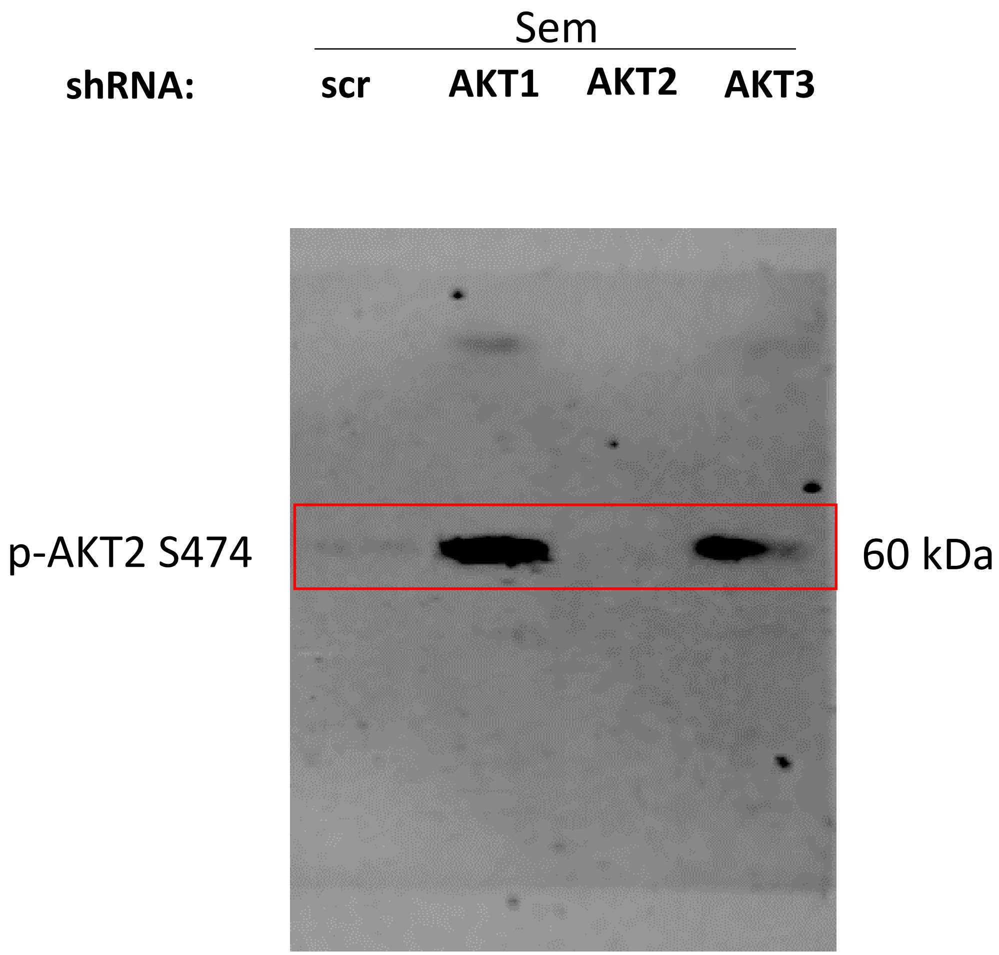
Sem
shRNA:
scr
AKT1
AKT2
AKT3
p-AKT2 S474
60 kDa

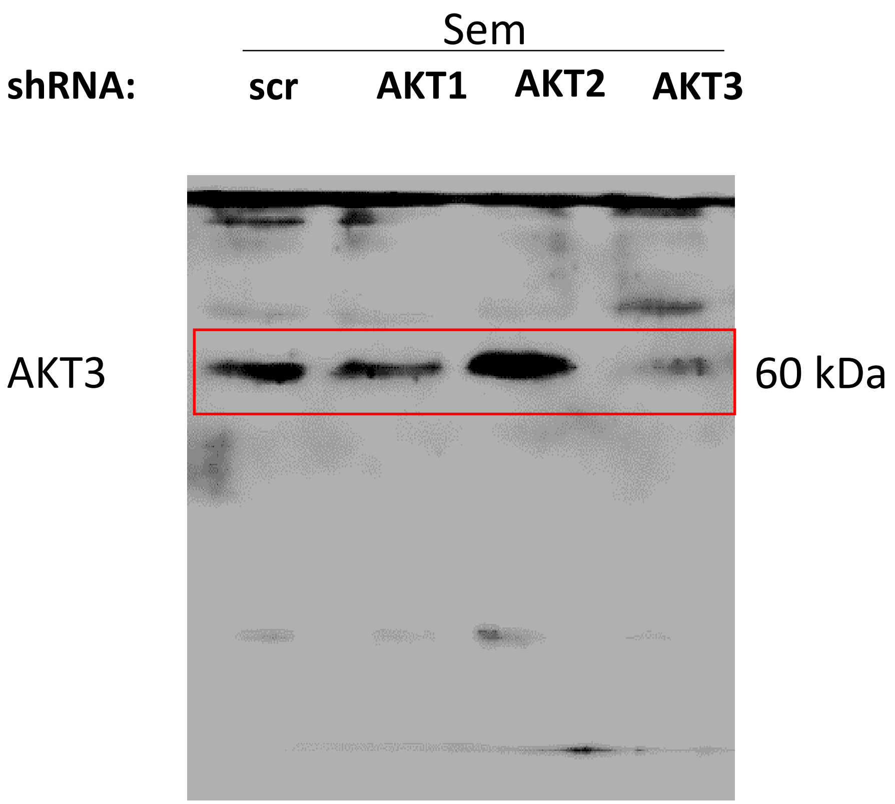
Sem
shRNA:
scr
AKT1
AKT2
AKT3
AKT3
60 kDa

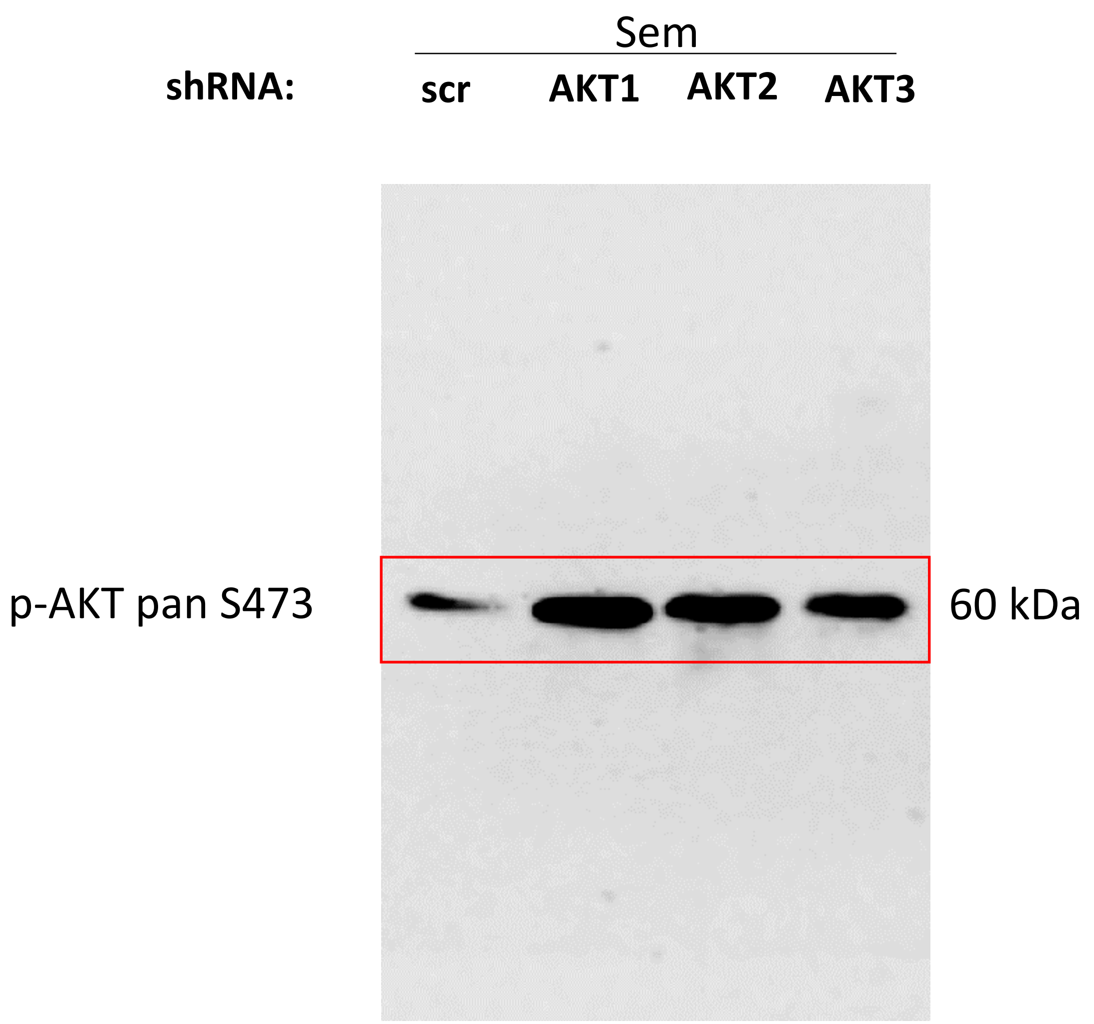
Sem
shRNA: scr AKT1 AKT2 AKT3
p-AKT pan S473
60 kDa

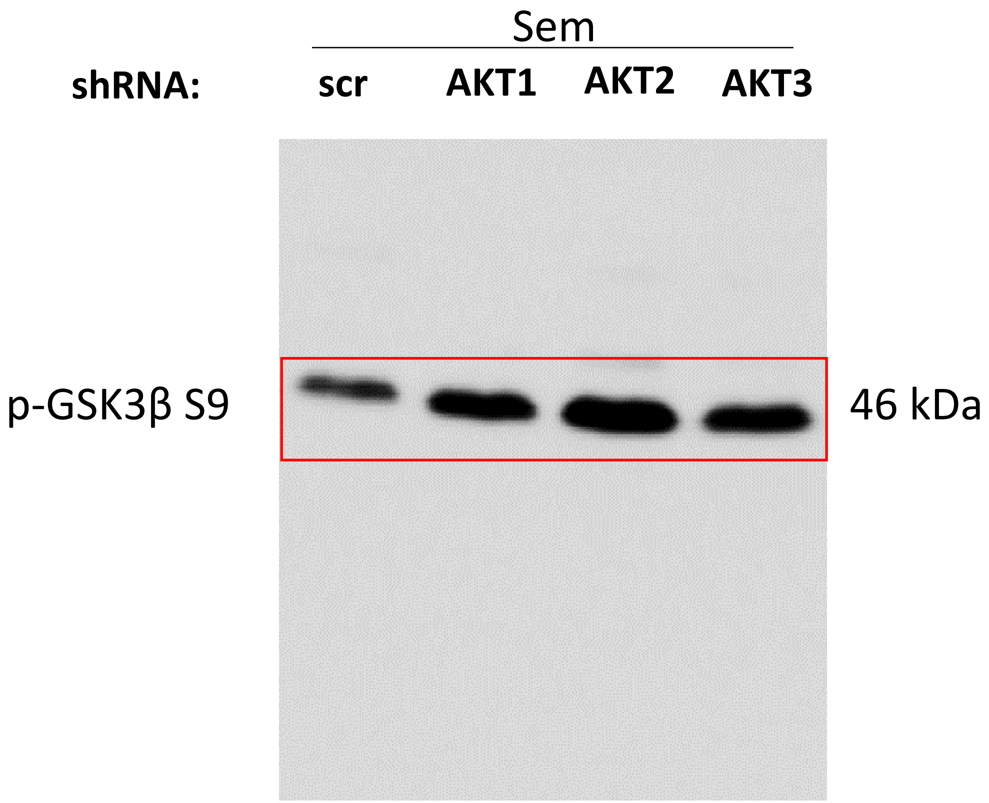
Sem
shRNA:
scr
AKT1
AKT2
AKT3
p-GSK3β S9
46 kDa

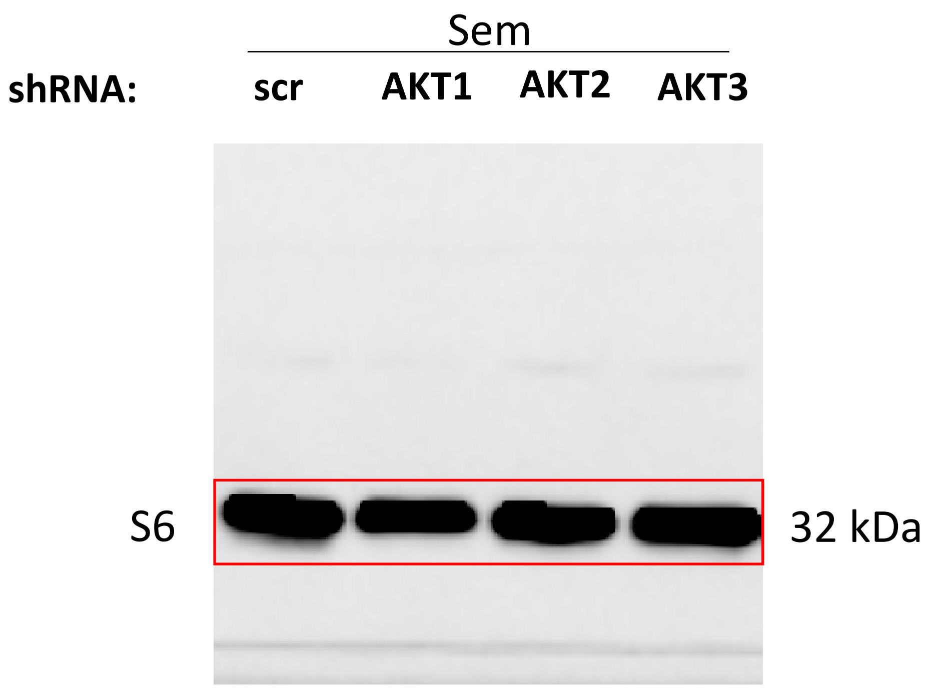
Sem
shRNA:
scr
AKT1
AKT2
AKT3
S6
32 kDa

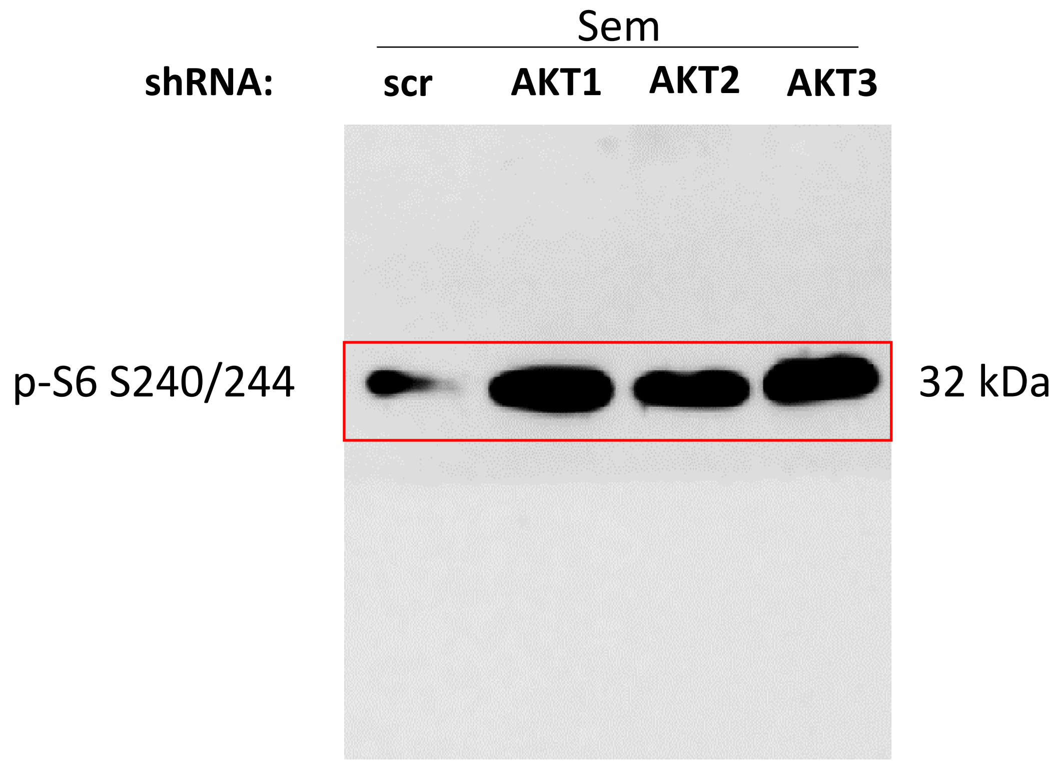
Sem
shRNA:
scr
AKT1
AKT2
AKT3
p-S6 S240/244
32 kDa

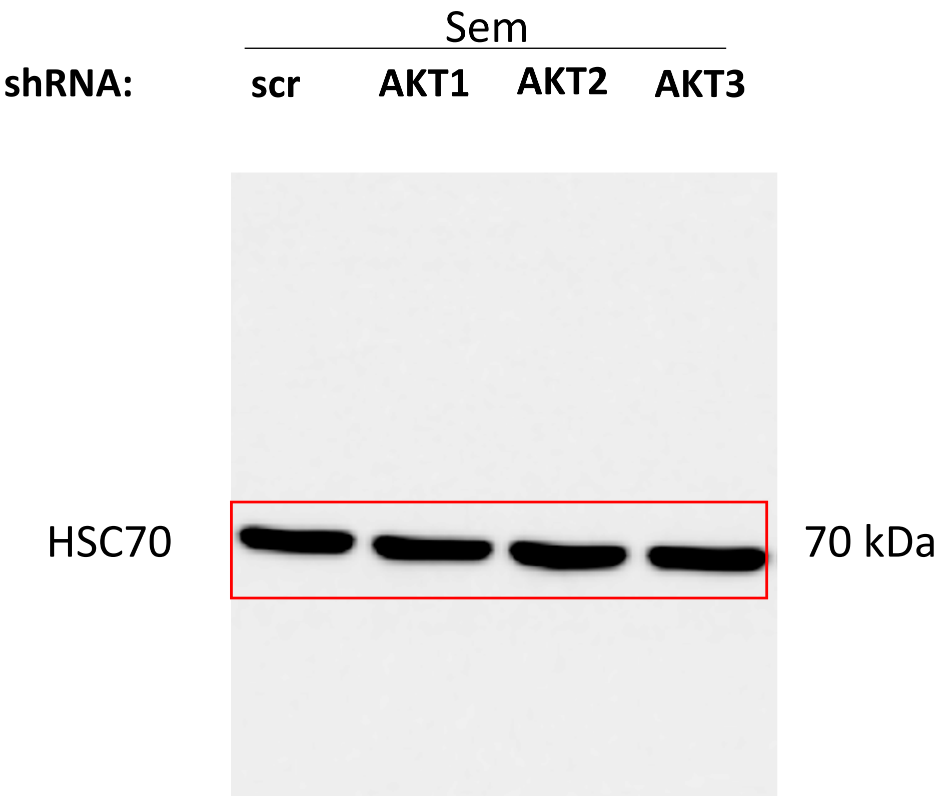
Sem
shRNA:
scr
AKT1
AKT2
AKT3
HSC70
70 kDa

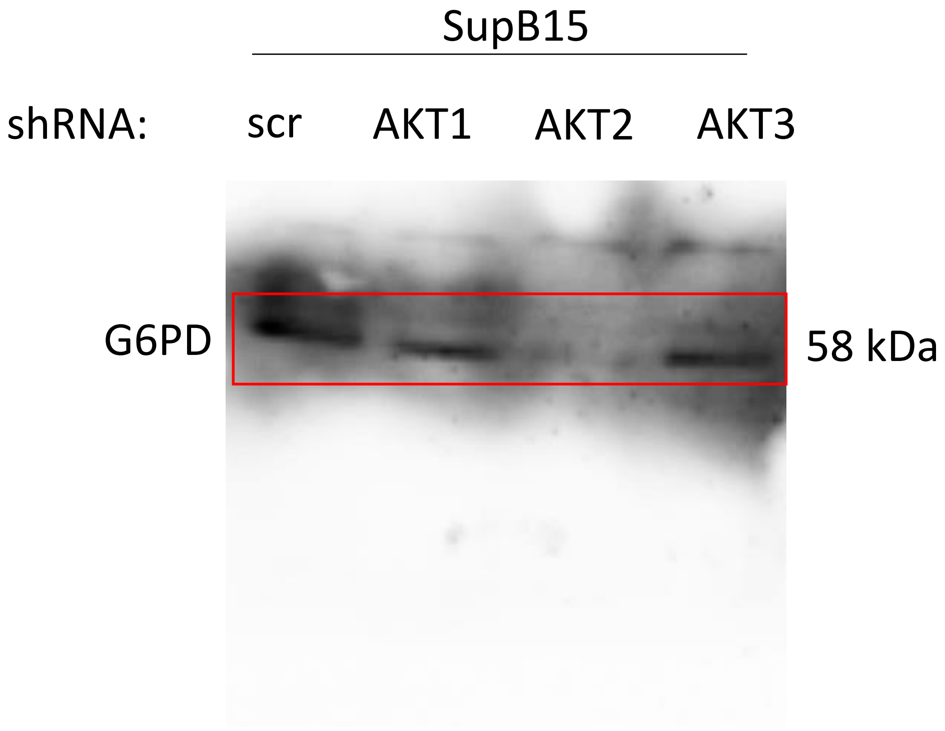
SupB15
shRNA:
scr
AKT1
AKT2
AKT3
G6PD
58 kDa

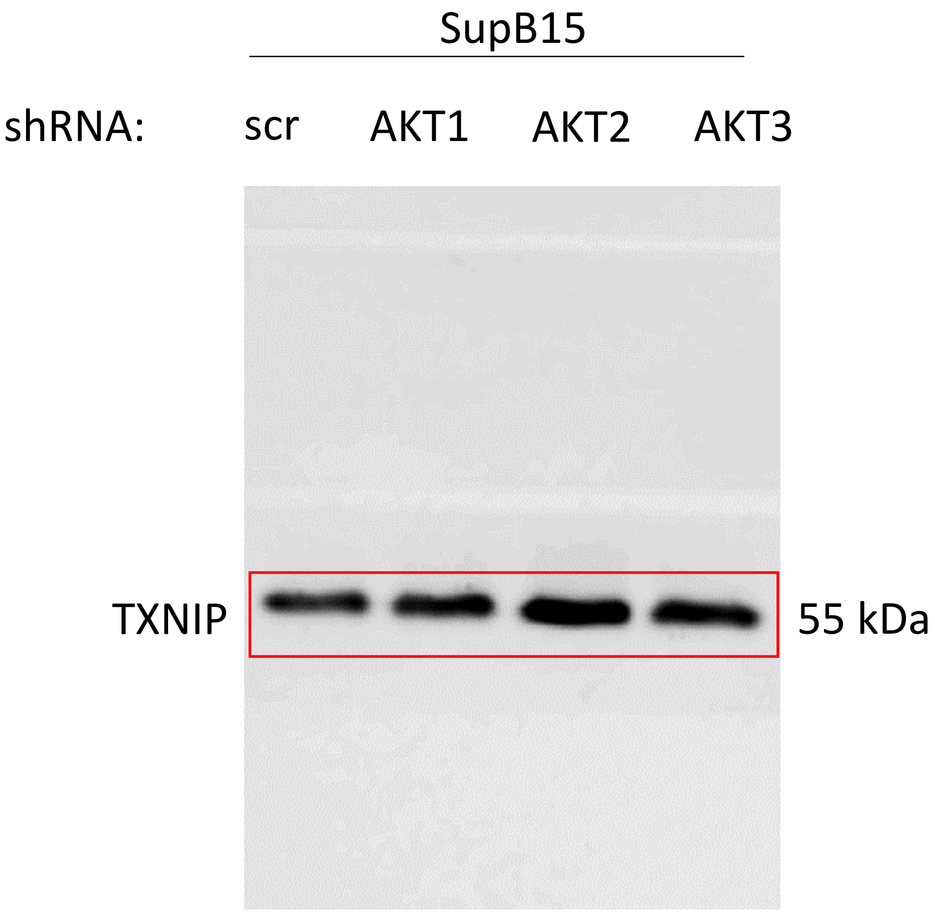
SupB15
shRNA:
scr
AKT1
AKT2
AKT3
TXNIP
55 kDa

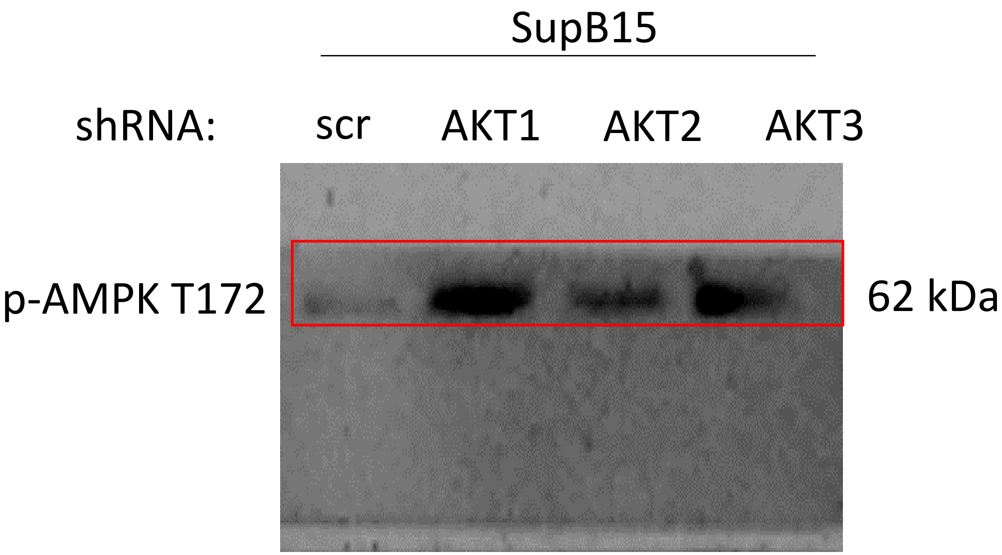
SupB15
shRNA:
scr
AKT1
AKT2
AKT3
p-AMPK T172
62 kDa

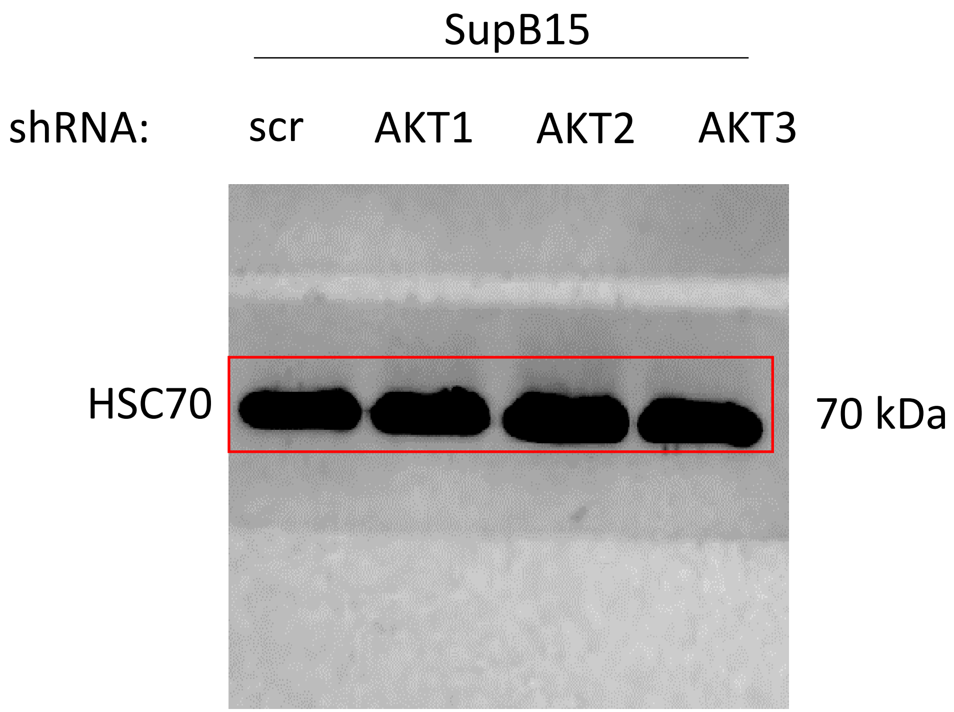
SupB15
shRNA:
scr
AKT1
AKT2
AKT3
HSC70
70 kDa

Figure 7C

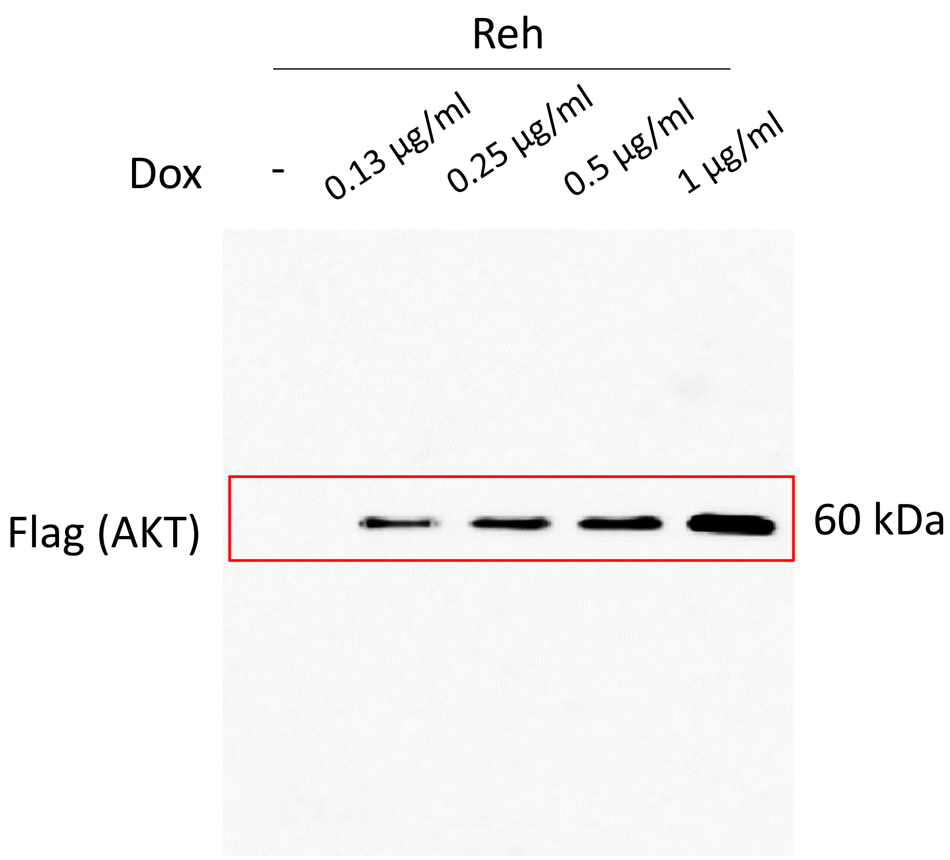
Reh
Dox
-
0.13 µg/ml
0.25 µg/ml
0.5 µg/ml
1 µg/ml
Flag (AKT)
60 kDa

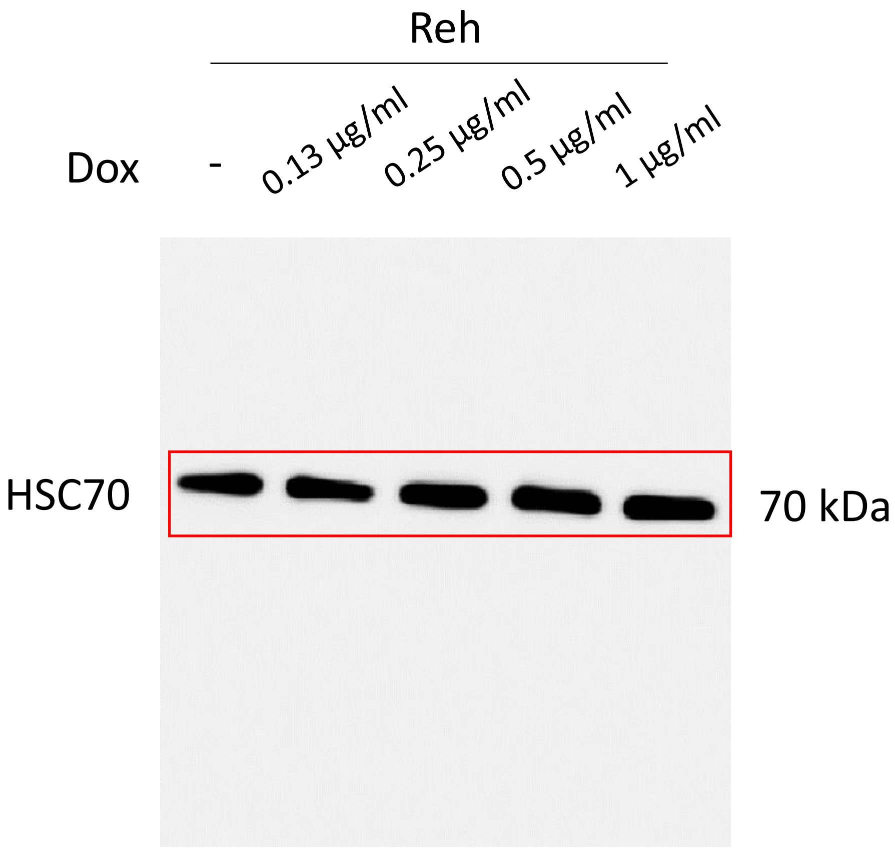
Reh
Dox
-
0.13 µg/ml
0.25 µg/ml
0.5 µg/ml
1 µg/ml
HSC70
70 kDa

Figure 7D

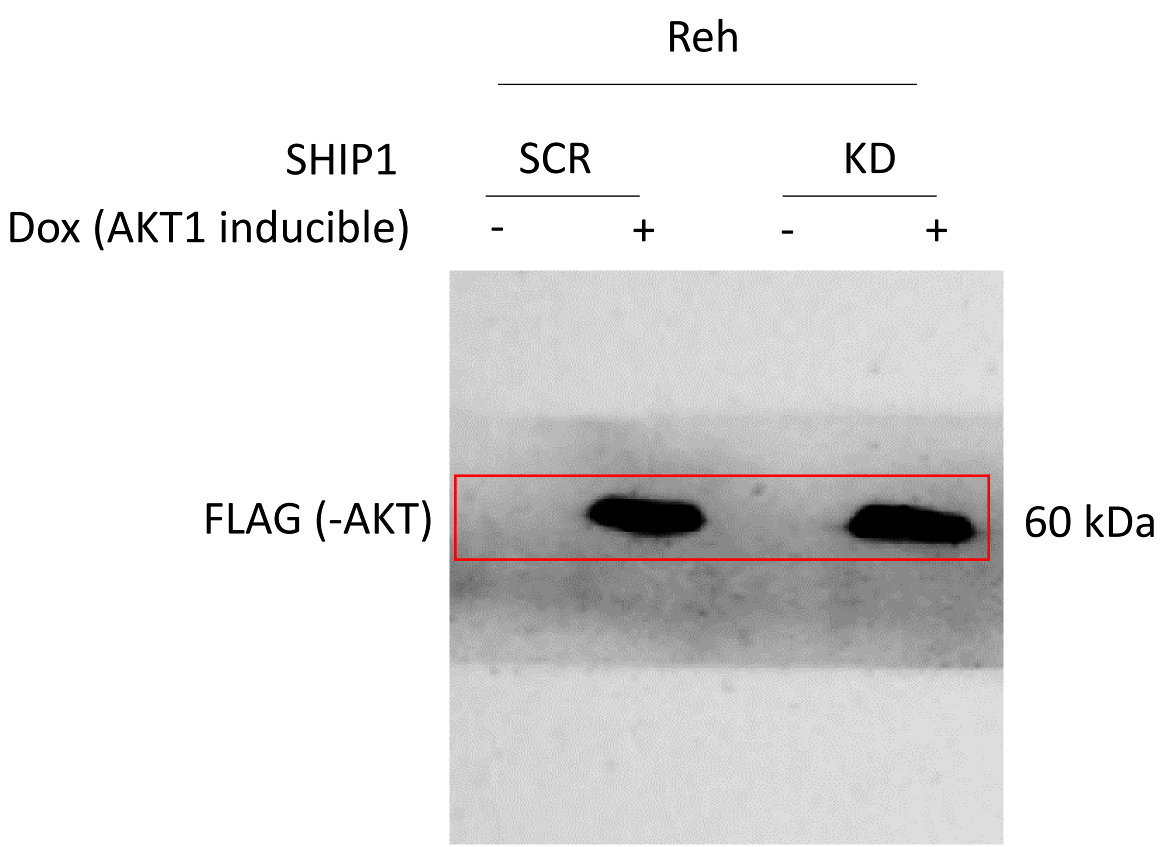
Reh
SHIP1
SCR
KD
Dox (AKT1 inducible)
-
+
-
+
FLAG (-AKT)
60 kDa

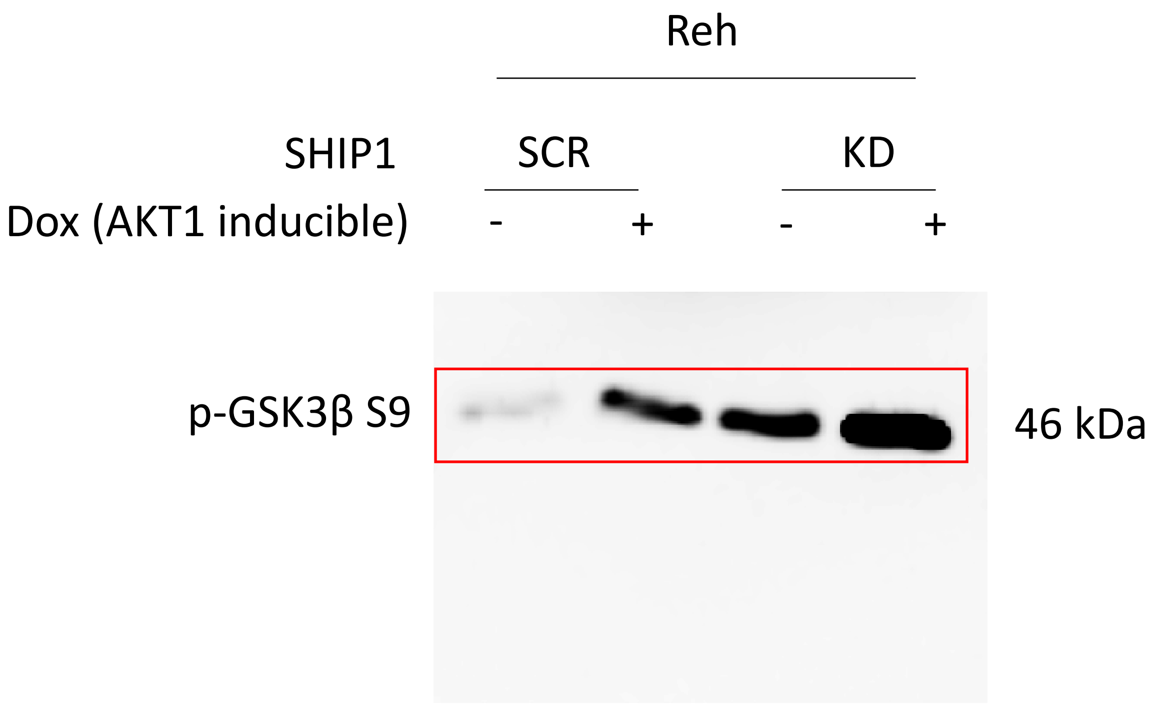
Reh
SHIP1
SCR
KD
Dox (AKT1 inducible)
-
+
-
+
p-GSK3β S9
46 kDa

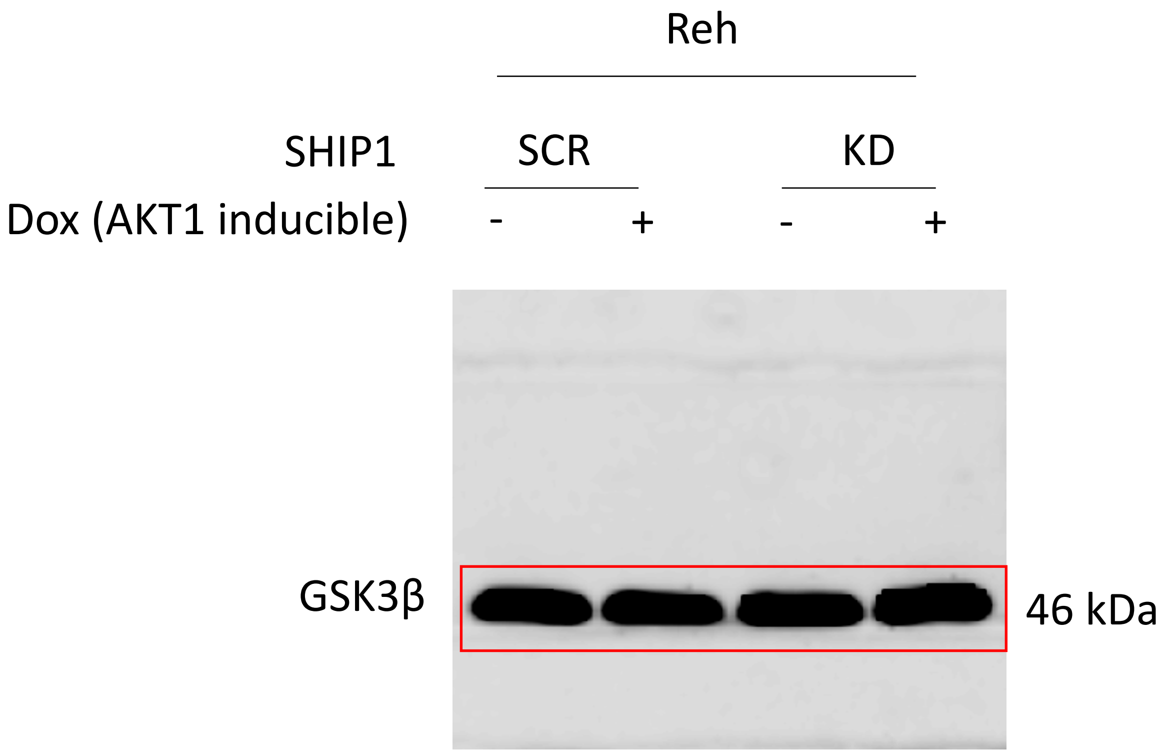
Reh
SHIP1
SCR
KD
Dox (AKT1 inducible)
- + - +
GSK3β
46 kDa

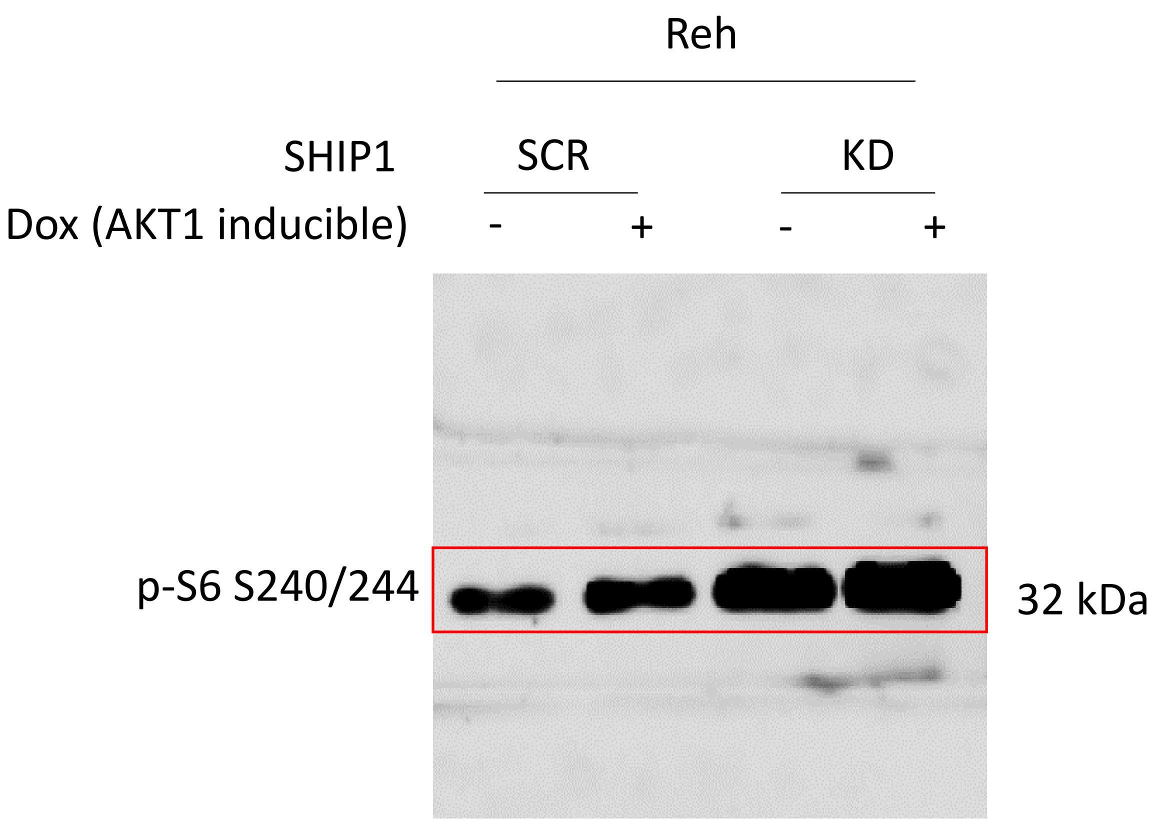
Reh
SHIP1
SCR
KD
Dox (AKT1 inducible)
-
+
-
+
p-S6 S240/244
32 kDa

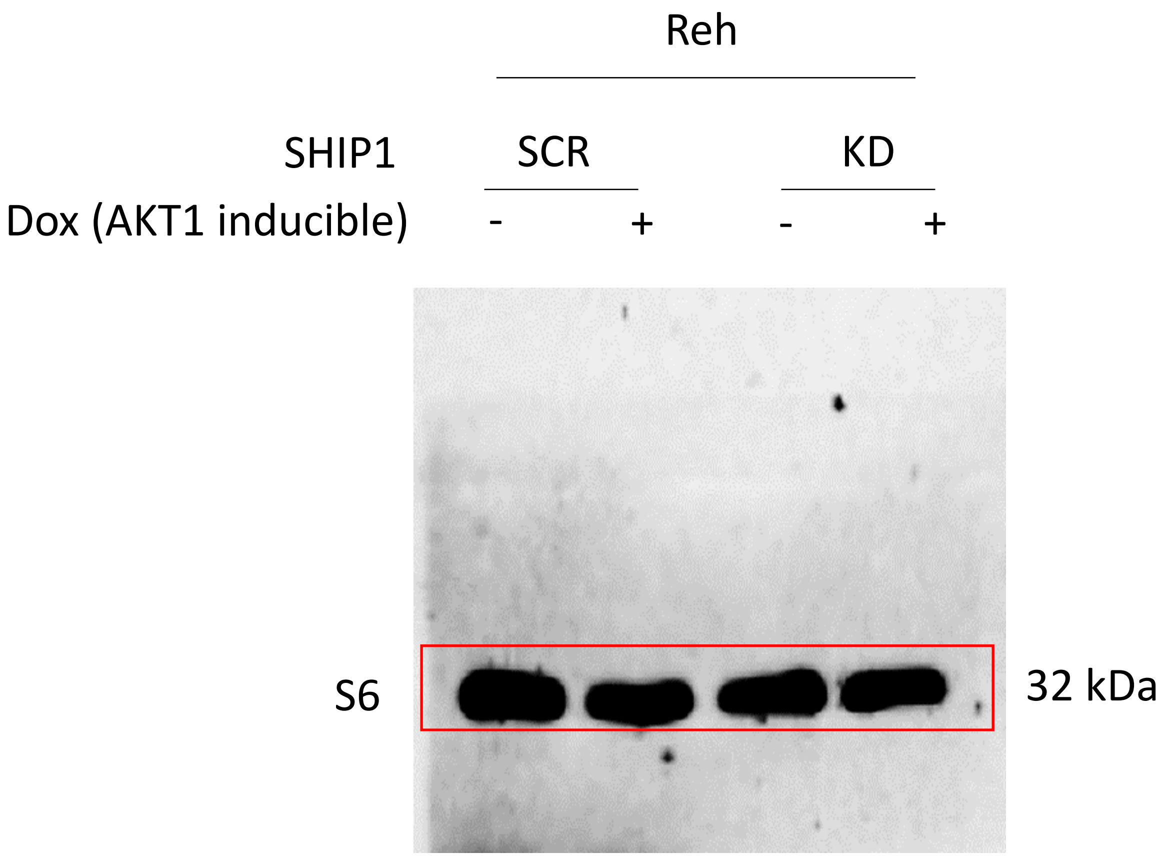
Reh
SHIP1
SCR
KD
Dox (AKT1 inducible)
-
+
-
+
S6
32 kDa

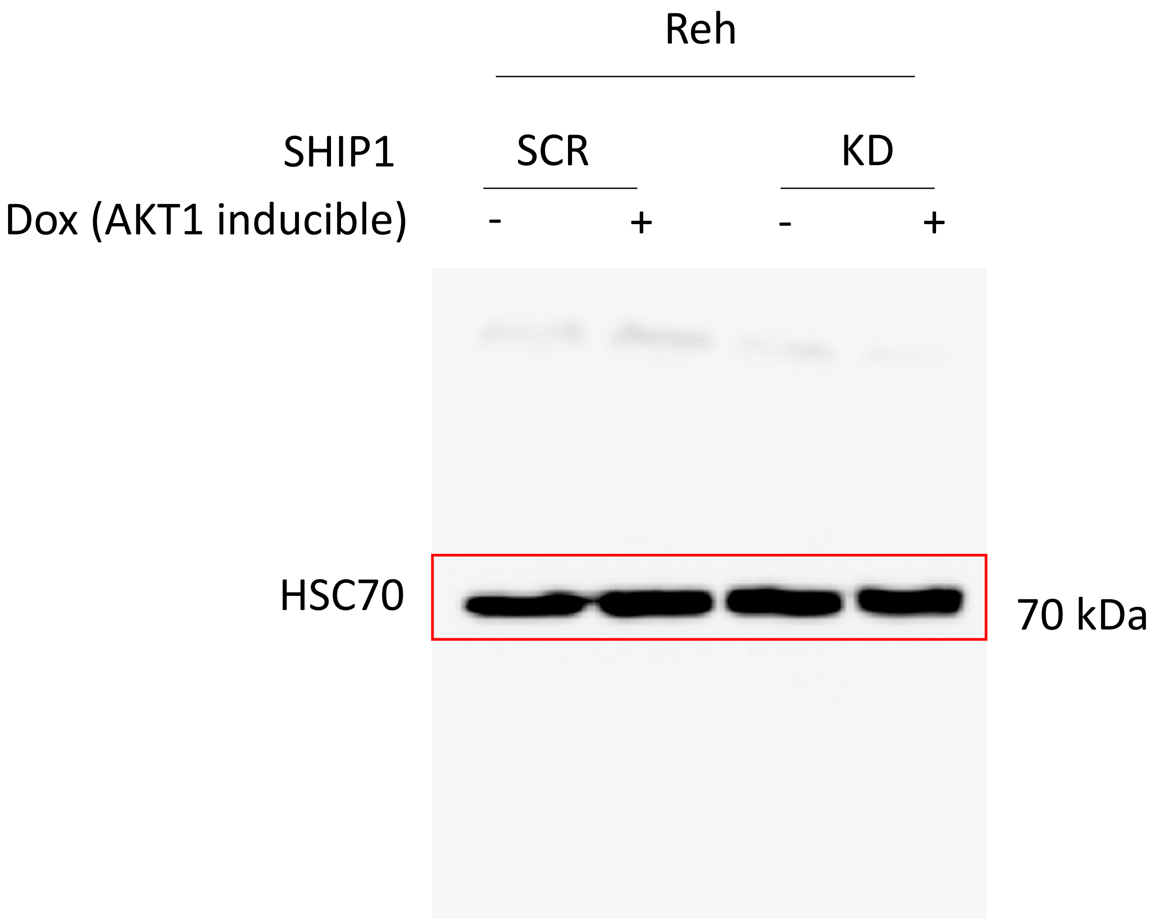
Reh
SHIP1
SCR
KD
Dox (AKT1 inducible)
-
+
-
+
HSC70
70 kDa

Figure 7G

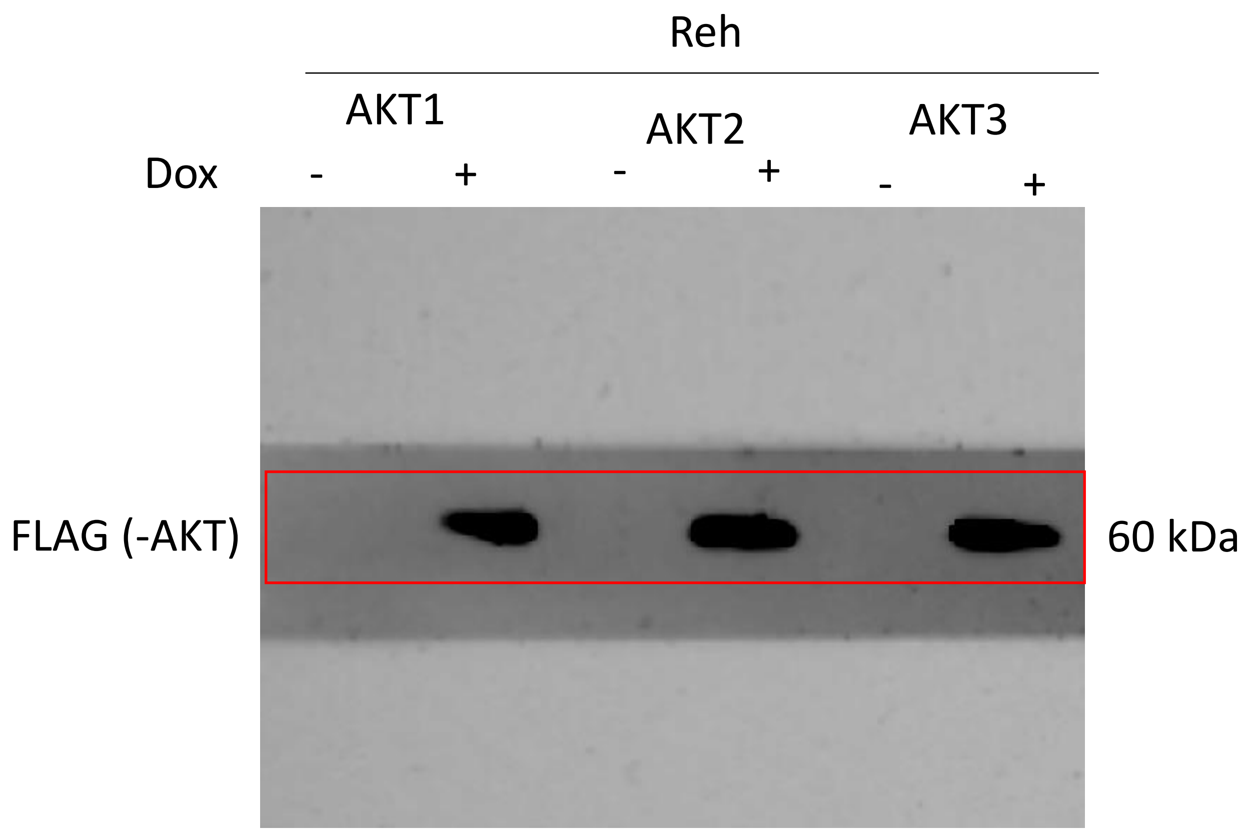
Reh
AKT1
AKT2
AKT3
Dox
-
+
-
+
-
+
FLAG (-AKT)
60 kDa

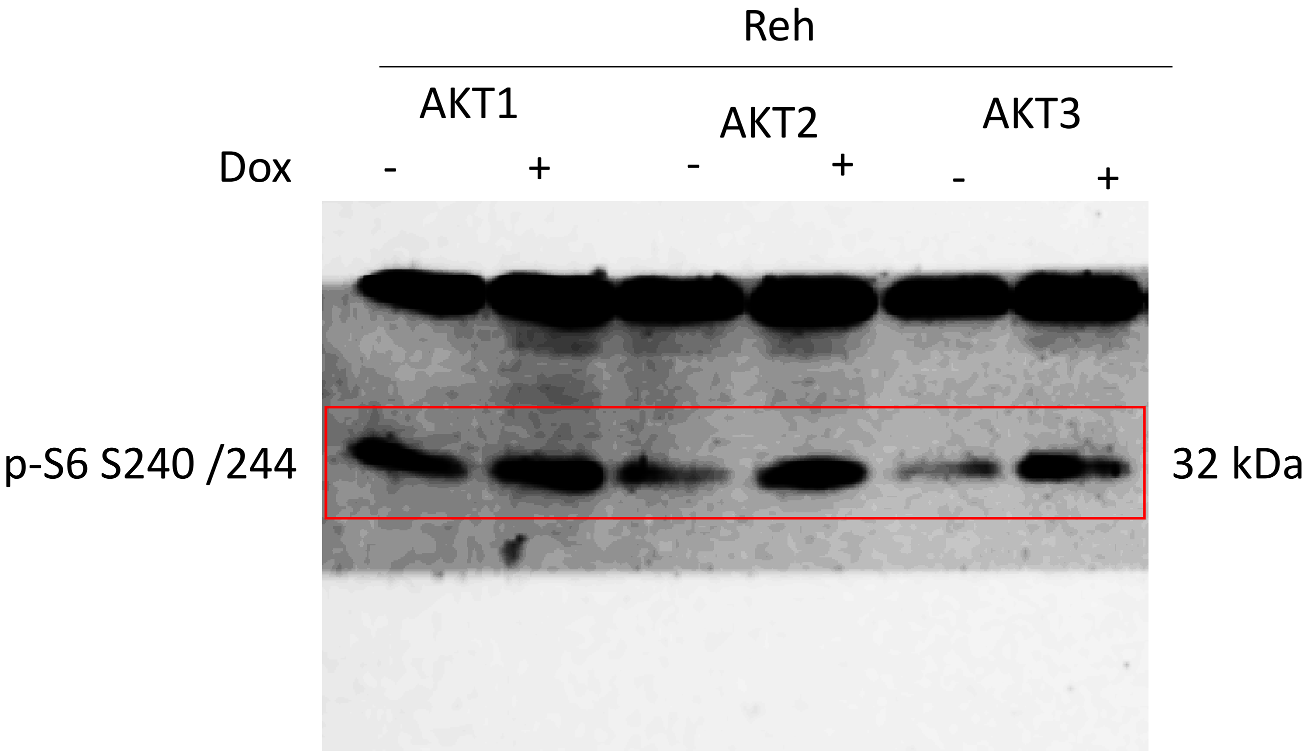
Reh
AKT1
AKT2
AKT3
Dox
-
+
-
+
-
+
p-S6 S240 /244
32 kDa

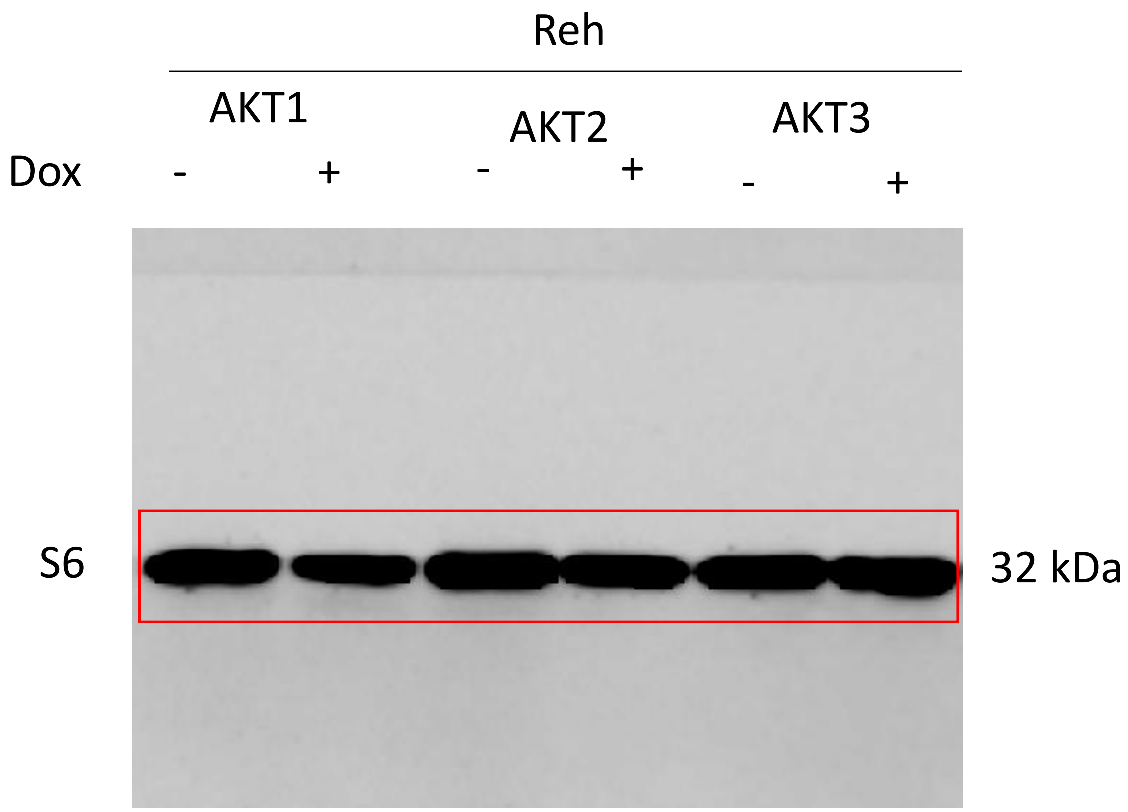
Reh
AKT1
AKT2
AKT3
Dox
-
+
-
+
-
+
S6
32 kDa

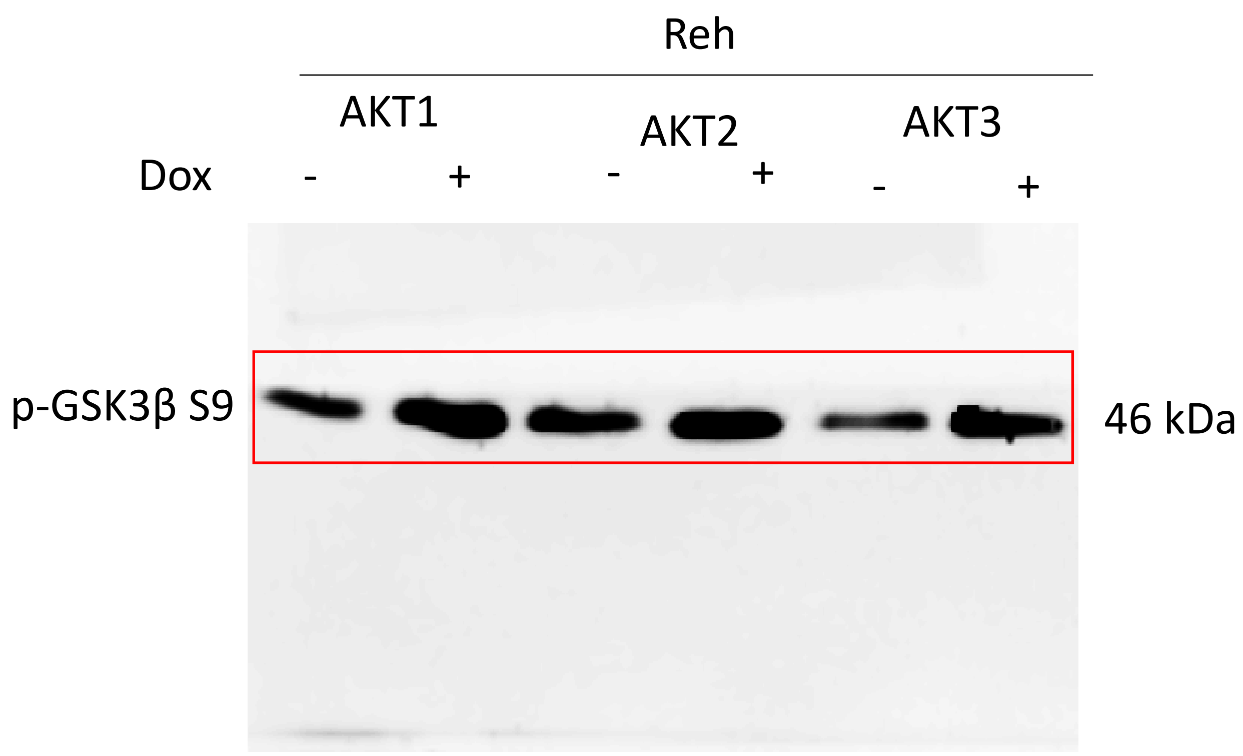
Reh
AKT1
AKT2
AKT3
Dox
-
+
-
+
-
+
p-GSK3β S9
46 kDa

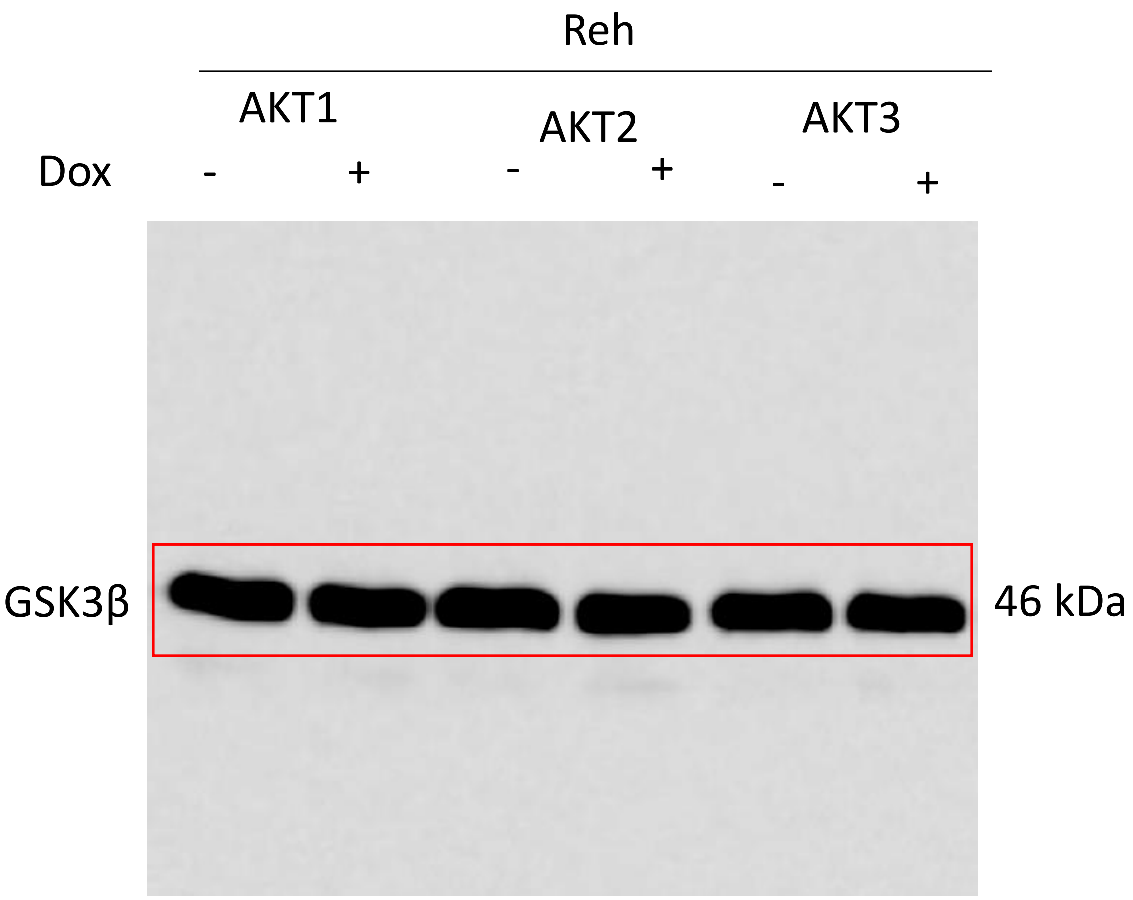
Reh
AKT1
AKT2
AKT3
Dox
-
+
-
+
-
+
GSK3β
46 kDa

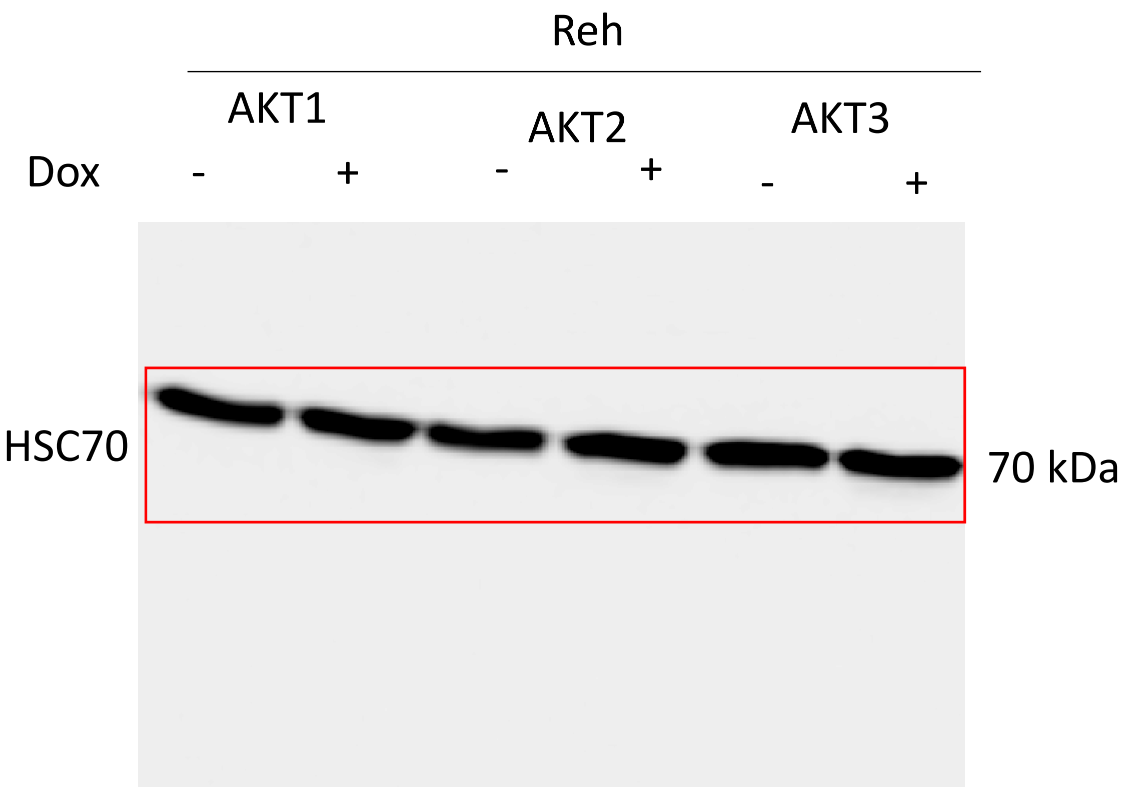
Reh
AKT1
AKT2
AKT3
Dox
-
+
-
+
-
+
HSC70
70 kDa

Figure S1C

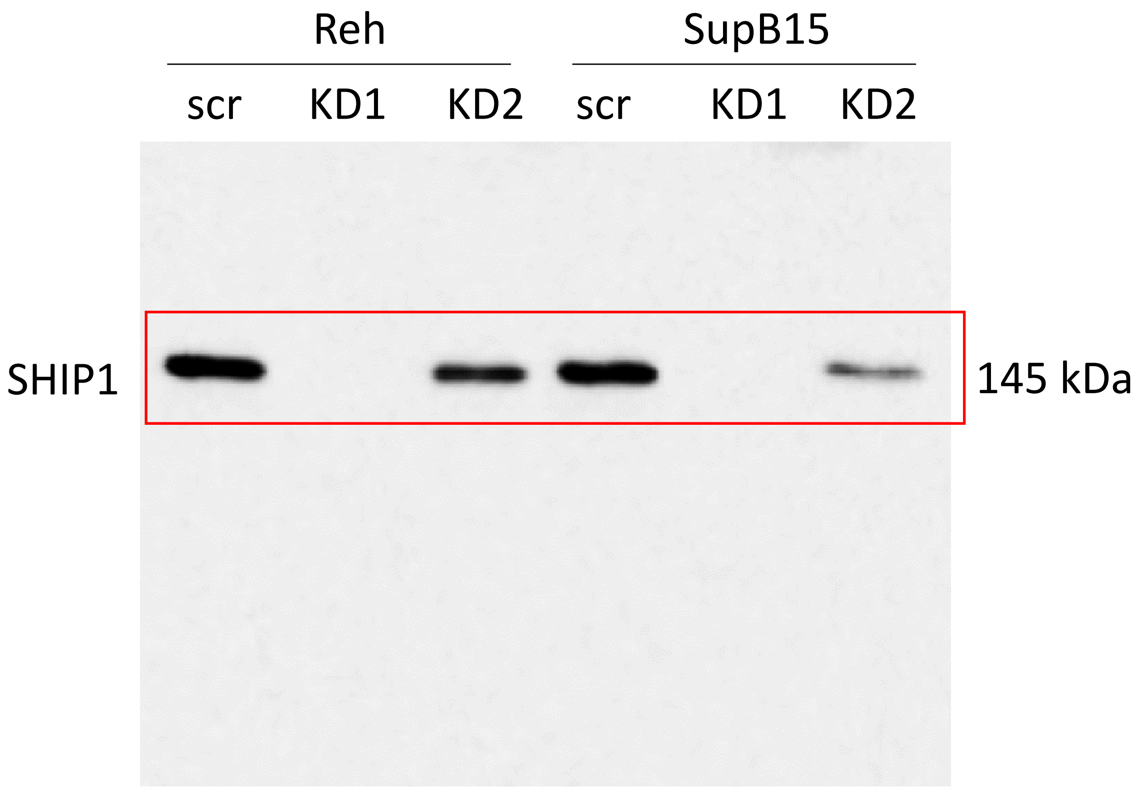
Reh
SupB15
scr
KD1
KD2
scr
KD1
KD2
SHIP1
145 kDa

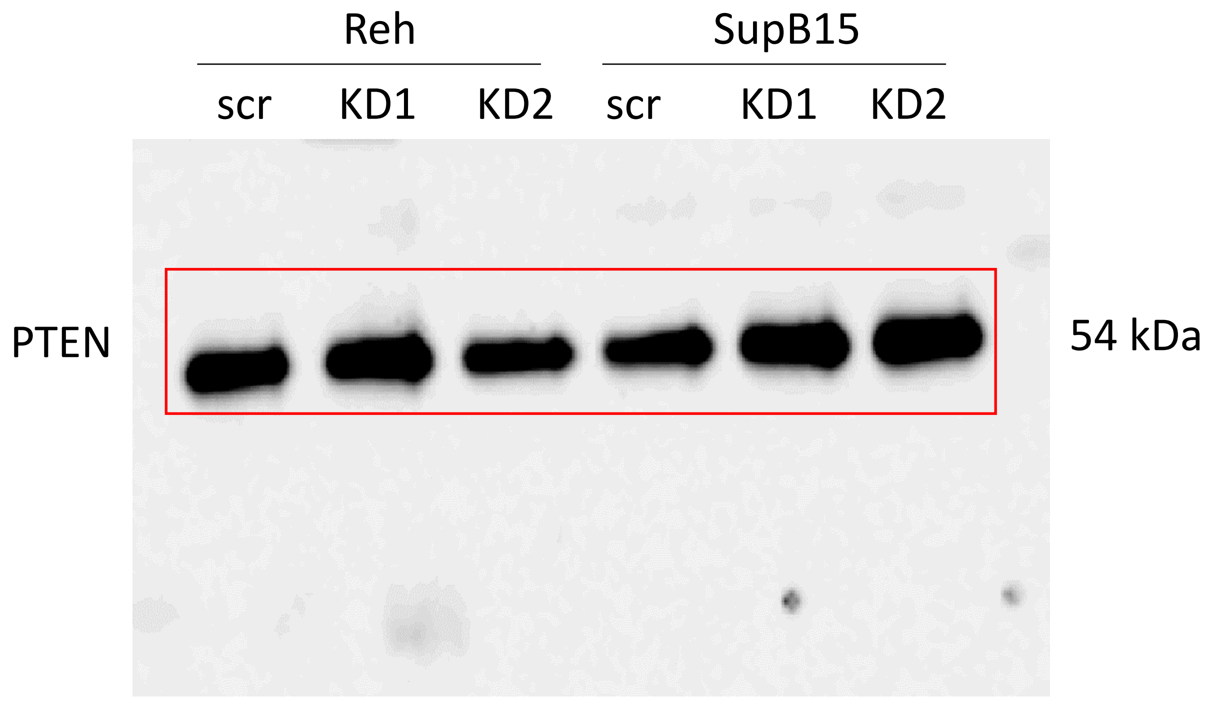

Reh
SupB15
scr
KD1
KD2
scr
KD1
KD2
PTEN
54 kDa

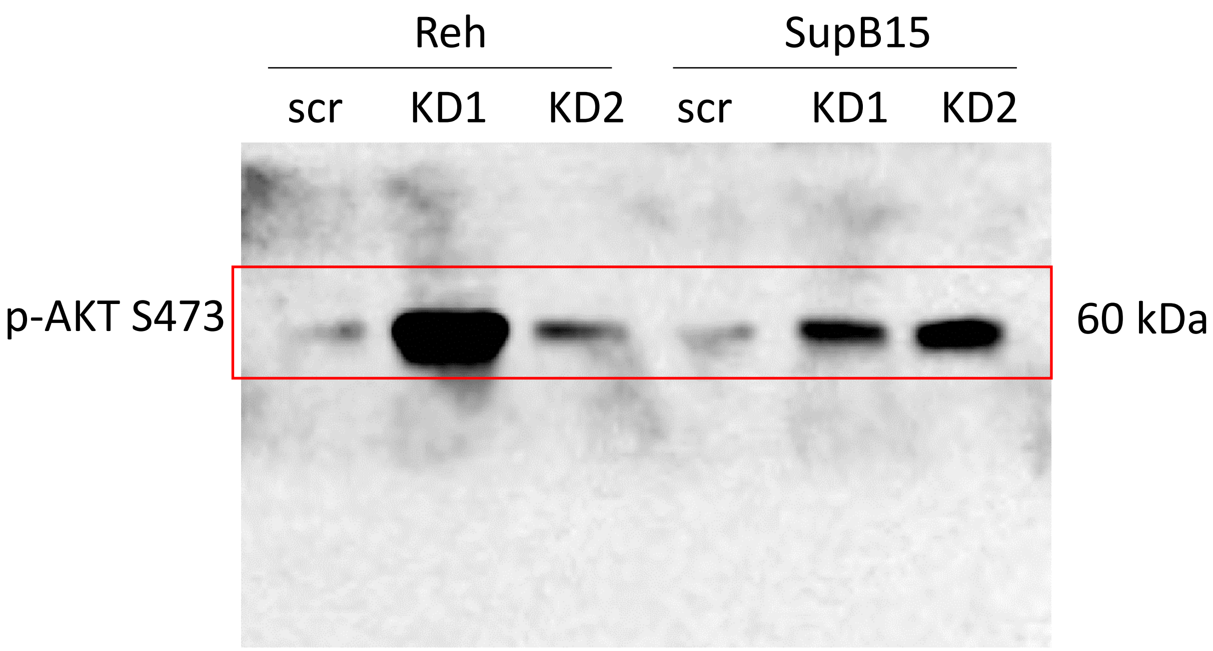
Reh
SupB15
scr
KD1
KD2
scr
KD1
KD2
p-AKT S473
60 kDa

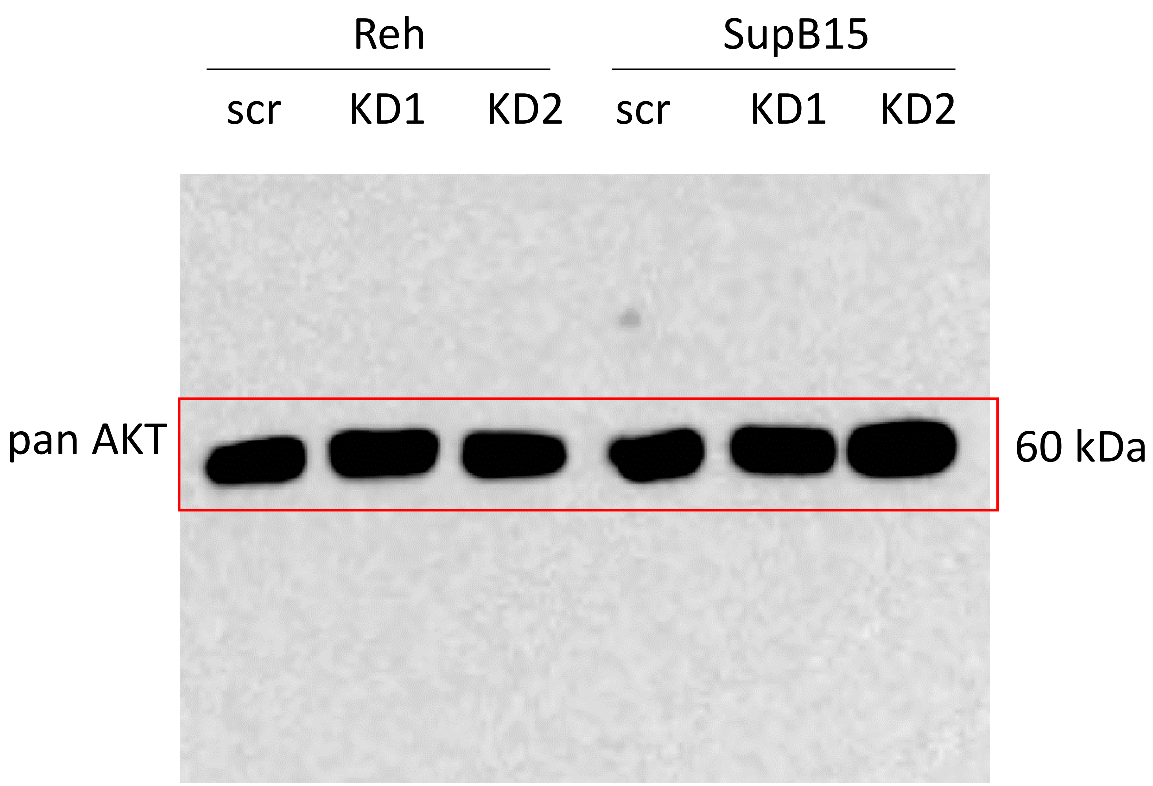
Reh
SupB15
scr
KD1
KD2
scr
KD1
KD2
pan AKT
60 kDa

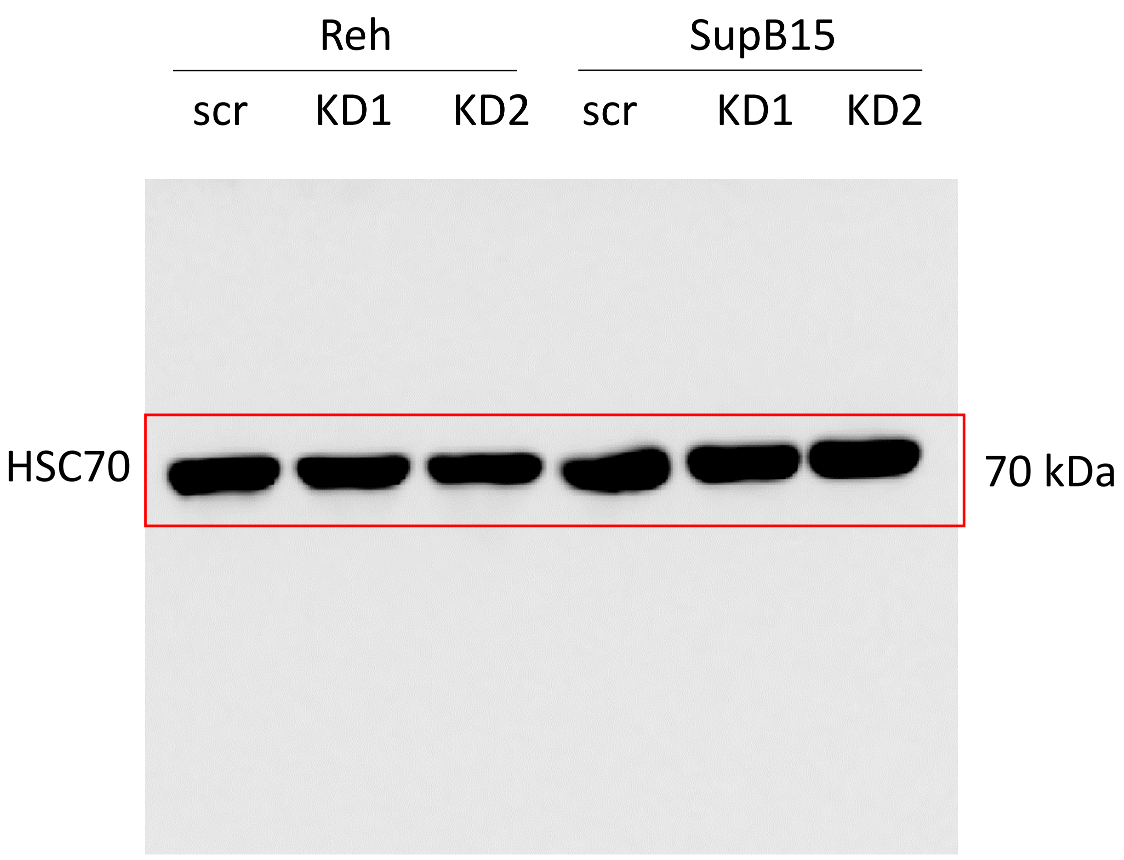
Reh
SupB15
scr
KD1
KD2
scr
KD1
KD2
HSC70
70 kDa

Figure S7A

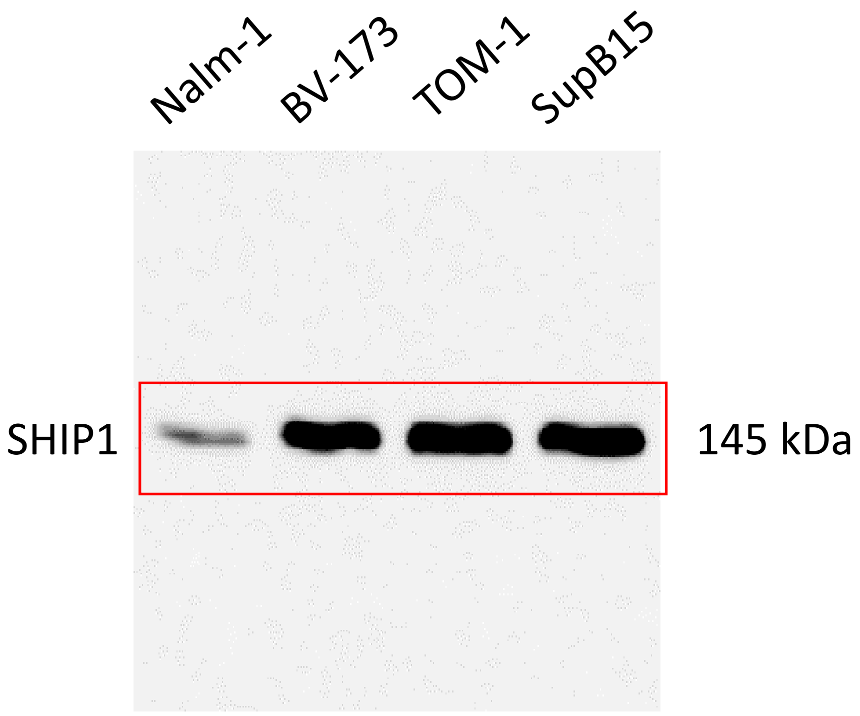
Nalm-1
BV-173
TOM-1
SupB15
SHIP1
145 kDa

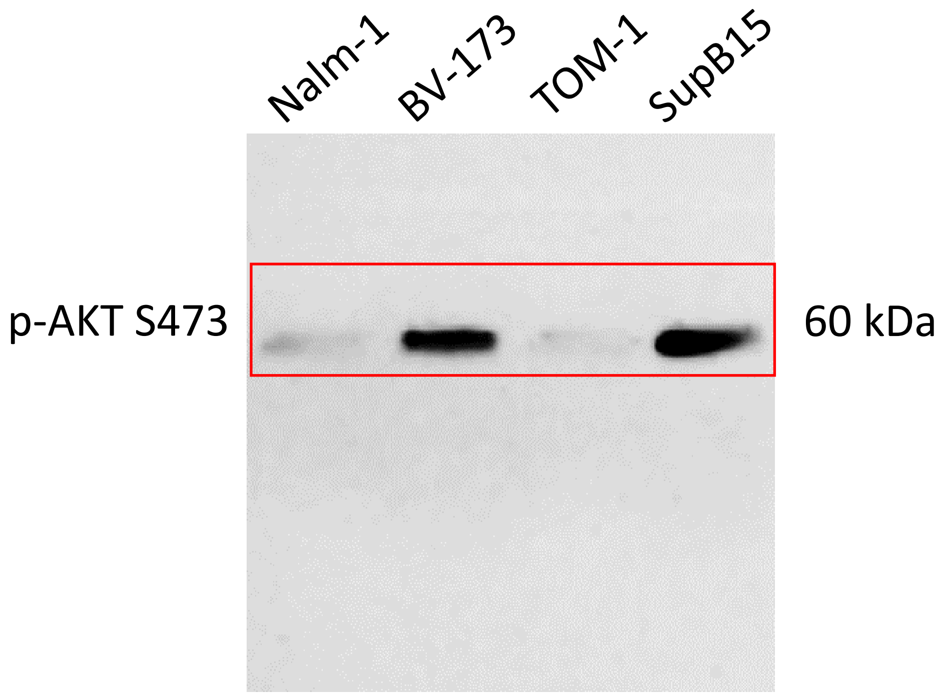
Nalm-1
BV-173
TOM-1
SupB15
p-AKT S473
60 kDa

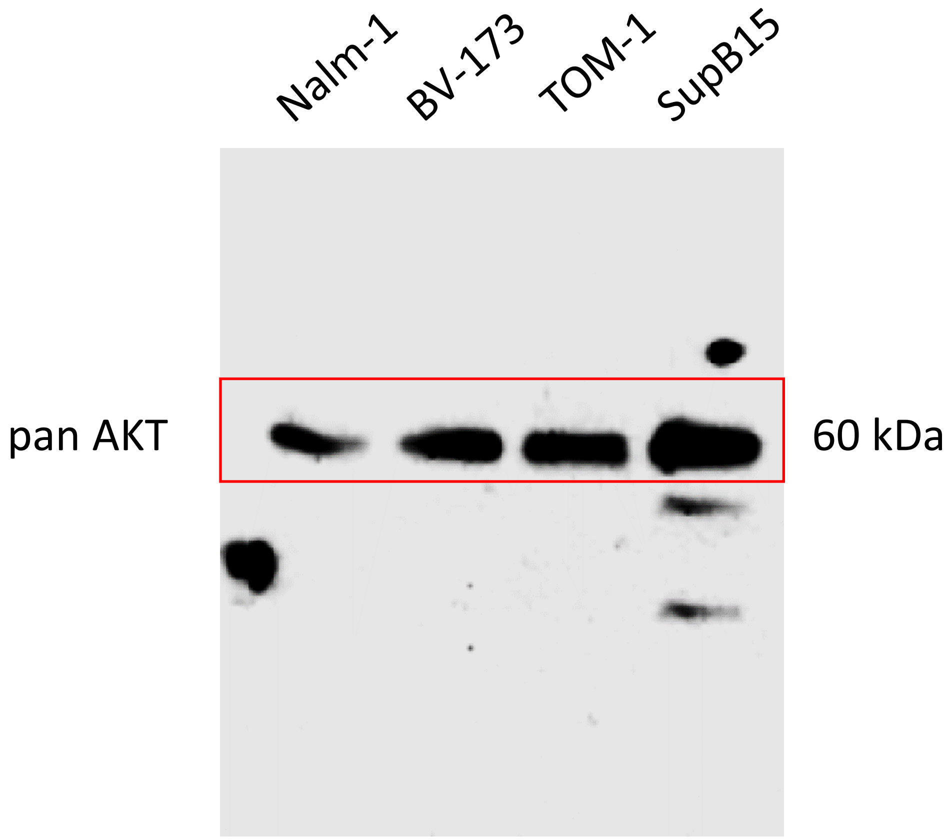
Nalm-1
BV-173
TOM-1
SupB15
pan AKT
60 kDa

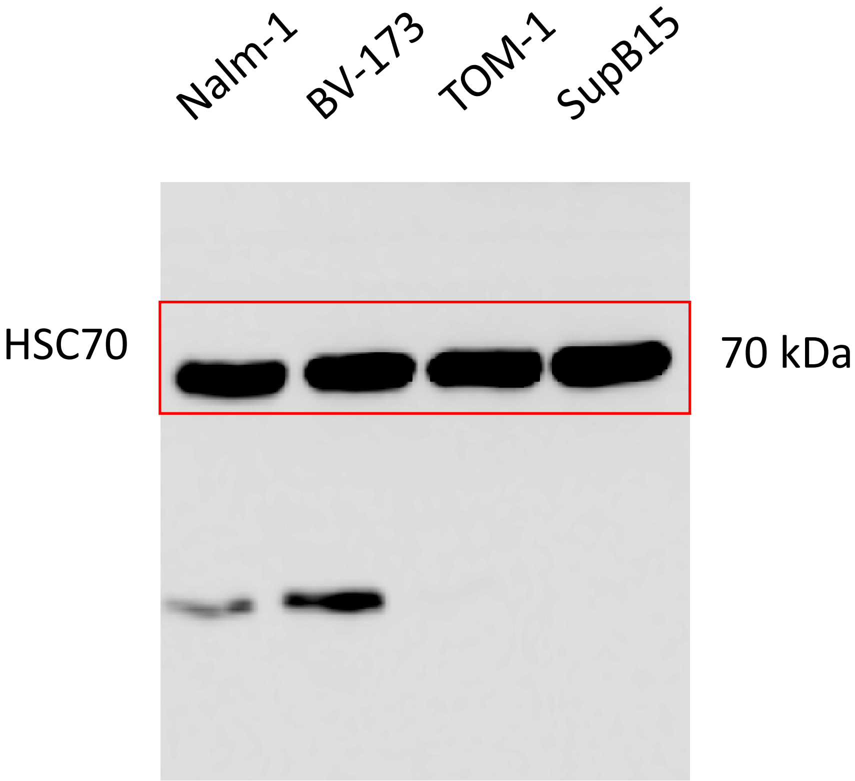
Nalm-1
BV-173
TOM-1
SupB15
HSC70
70 kDa

Figure S7B

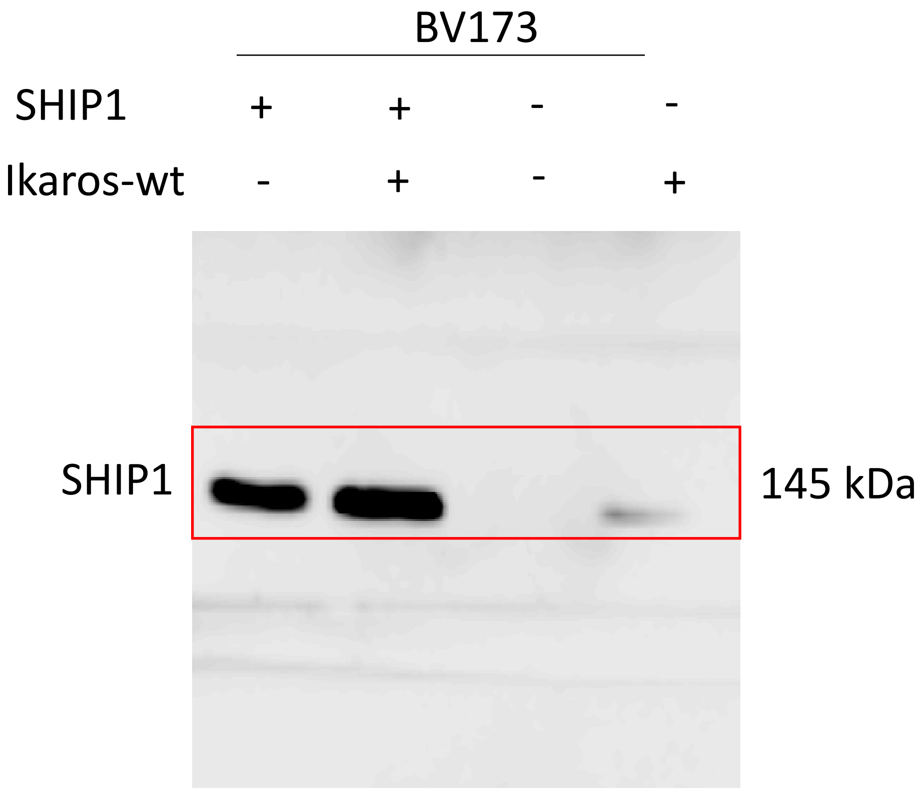
BV173
SHIP1 + + - -
Ikaros-wt - + - +
SHIP1
145 kDa

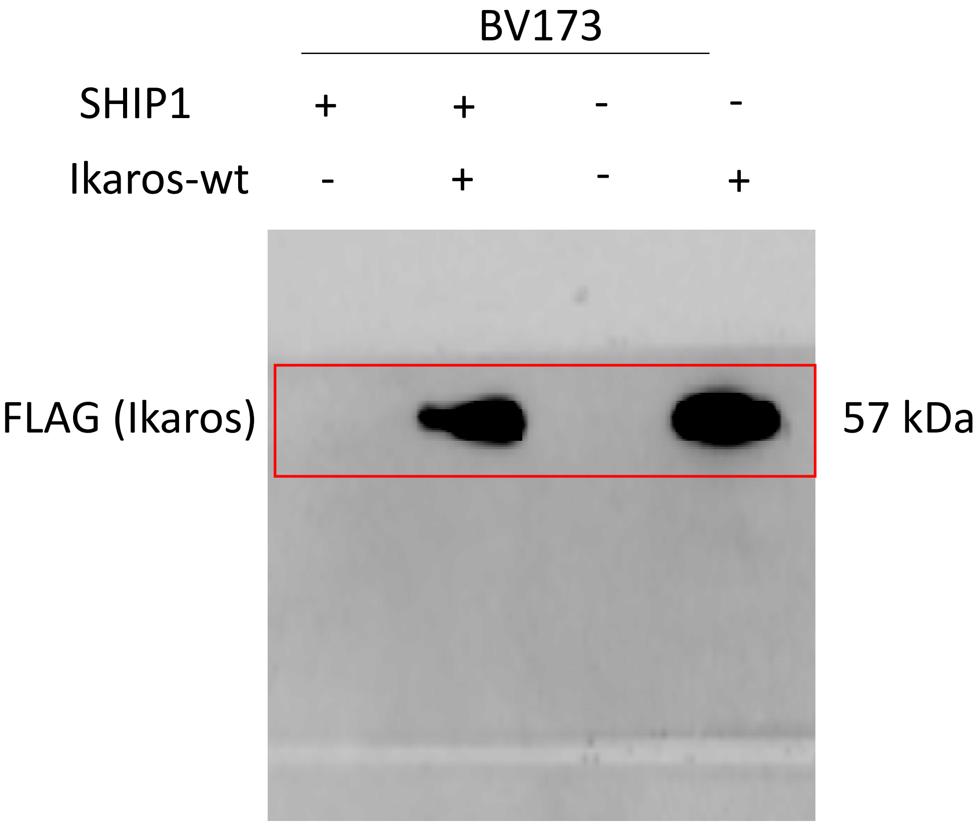
BV173
SHIP1 + + - -
Ikaros-wt - + - +
FLAG (Ikaros)
57 kDa

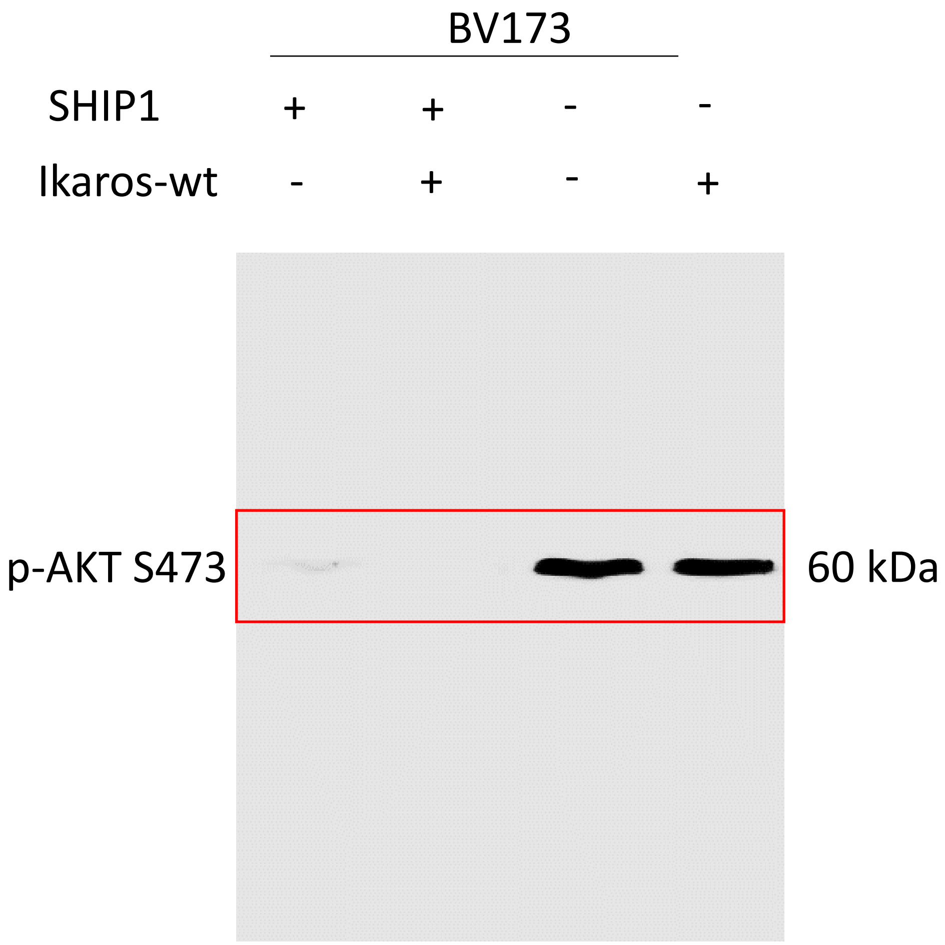
BV173
SHIP1 + + - -
Ikaros-wt - + - +
p-AKT S473
60 kDa

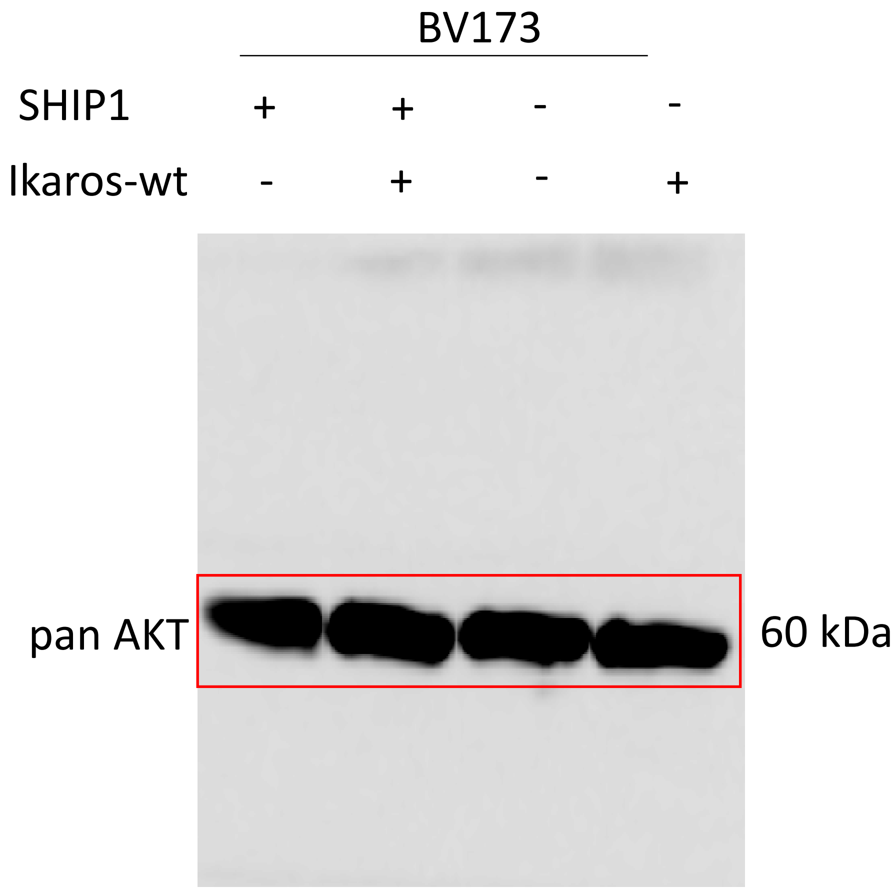
BV173
SHIP1 + + - -
Ikaros-wt - + - +
pan AKT
60 kDa

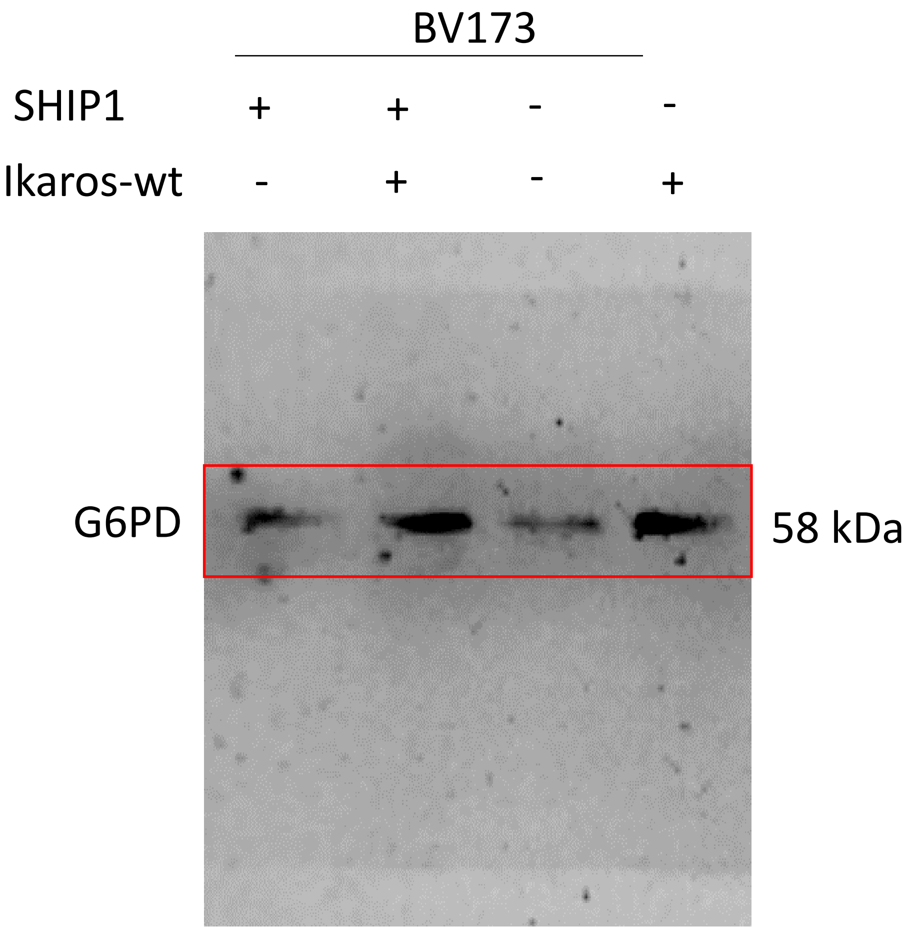
BV173
SHIP1 + + - -
Ikaros-wt - + - +
G6PD
58 kDa

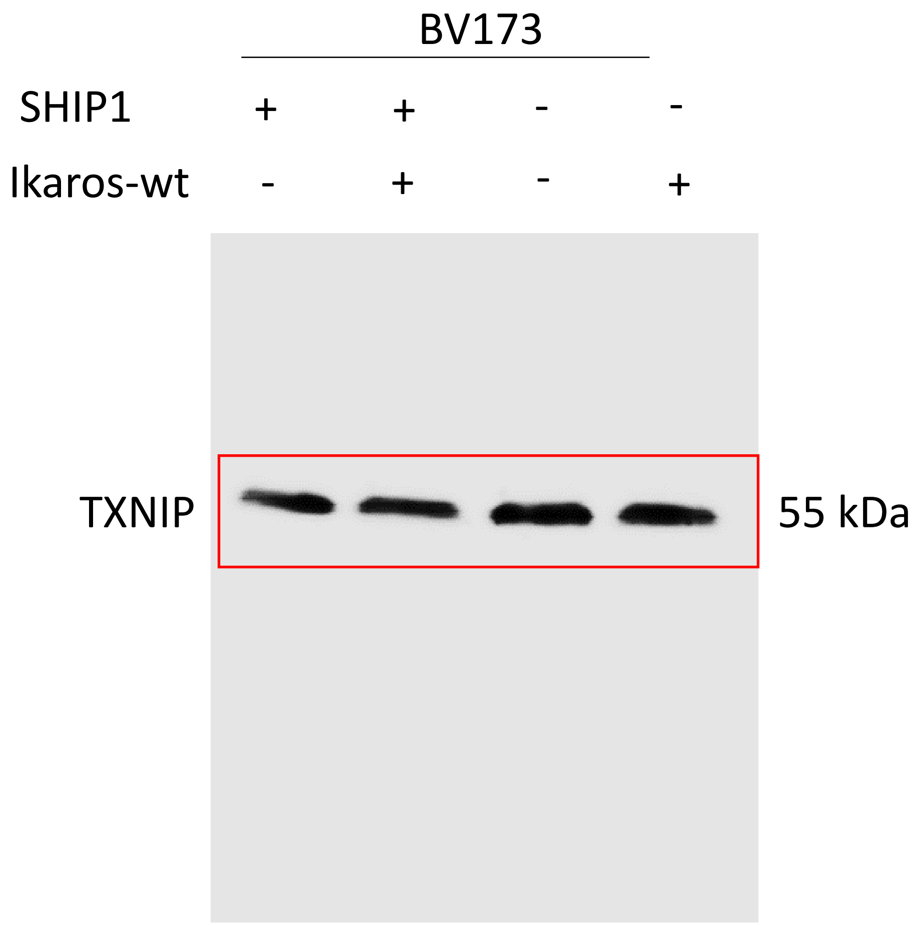
BV173
SHIP1 + + - -
Ikaros-wt - + - +
TXNIP
55 kDa

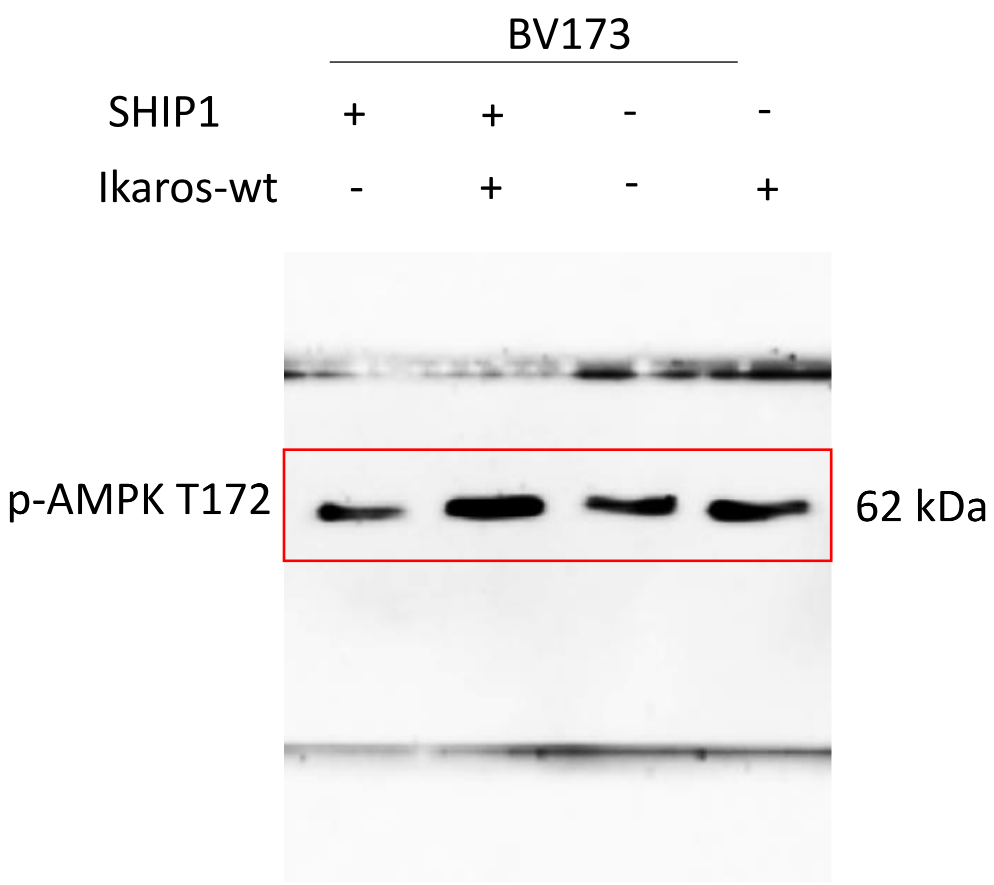

BV173
SHIP1 + + - -
Ikaros-wt - + - +
p-AMPK T172
62 kDa

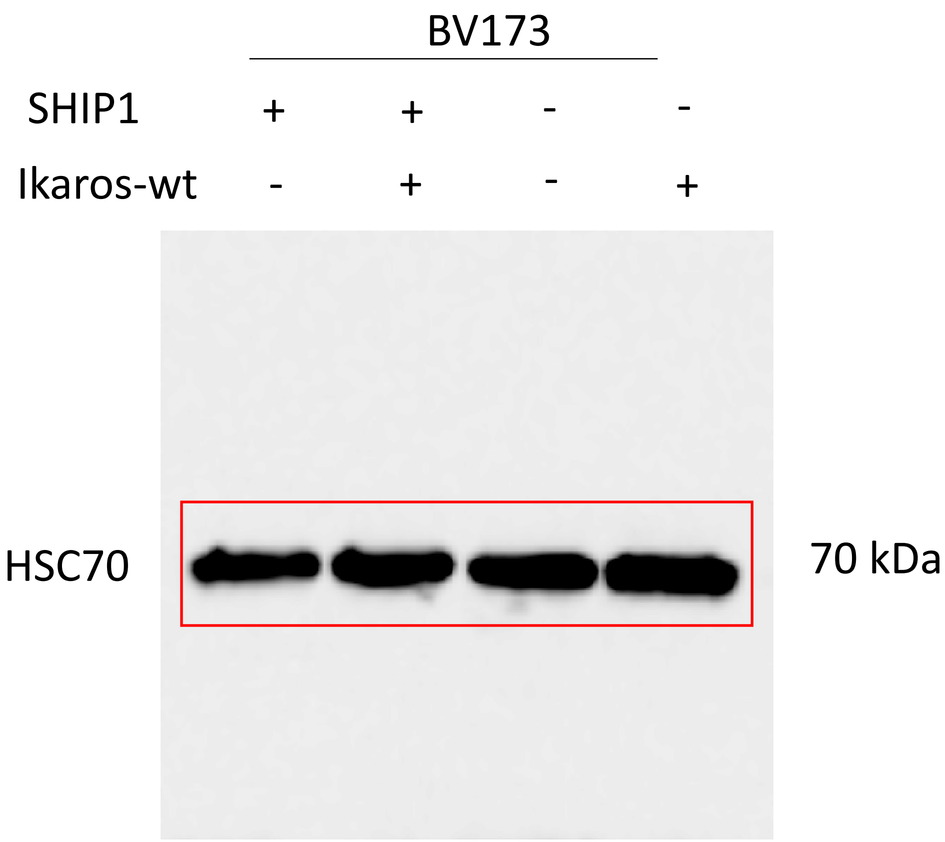
BV173
SHIP1 + + - -
Ikaros-wt - + - +
HSC70
70 kDa
